# Supplementary material for: Analysis of protrusion dynamics in amoeboid cell motility by means of regularized contour flows
Source: PLoS Comput Biol. 2021 Aug 23;17(8):e1009268. doi: 10.1371/journal.pcbi.1009268 (PMC8412247; doi:10.1371/journal.pcbi.1009268)
Supplement: S1 Text — (PDF) [file pcbi.1009268.s001.pdf]

Supporting Information S1

Daniel Schindler Ted Moldenhawer Maike Stange
Valentino Lepro Carsten Beta Matthias Holschneider
Wilhelm Huisinga

August 2, 2021

Contents

1 Gaussian process regression (GPR) 2
1.1 General definitions and theory 2
1.2 Interpolation model of cell outlines 4
1.3 Computation of curvature using GPR 5
1.4 Parameter estimation 6

2 Regularized contour flow method (RCFM) 9
2.1 Implementation of regularized flows 9
2.2 Supporting computations 13

1 Gaussian process regression (GPR)

1.1 General definitions and theory

In this section, we characterize briefly the main aspects of the Gaussian process regression which we used to determine smooth two-dimensional representations of cell membranes. In this context, we introduce common notations and formulas which are necessary for the following sections. In general, the Gaussian process regression (GPR), also called kriging, offers a Bayesian framework for regression analysis. It does not only provide a single regression function but also quantifies the associated uncertainty. Detailed studies of Gaussian processes in machine learning and especially in regression analysis can be found in many textbooks such as [1, 2]. In [3, 4] short summaries of GPR is presented; details on the implementation of GPR can be found in [5].

First, we assume a training data set $\mathcal{D} = \{(\mathbf{x}_i, y_i) \mid i = 1, \ldots, n\}$ consisting of n observations. The input vectors are denoted as $\mathbf{x}_i \in \mathbb{R}^d$, $d \in \mathbb{N}$ and the corresponding scalar outputs are denoted as $y_i \in \mathbb{R}$. Now, we are interested in a distribution of regression functions $f : \mathbb{R}^d \to \mathbb{R}$ which are described by Gaussian processes. Due to the fact that Gaussian processes are uniquely defined by its mean function $\mathbf{x} \mapsto \mu(\mathbf{x})$ and its covariance function $(\mathbf{x}, \mathbf{x}') \mapsto \Sigma(\mathbf{x}, \mathbf{x}')$, we introduce the following notation for Gaussian processes:

$$f(\mathbf{x}) \sim \mathcal{GP}\left(\mu(\mathbf{x}),\ \Sigma(\mathbf{x}, \mathbf{x}')\right),$$

where the mean function and the covariance function are given by

$$\begin{aligned}\mu(\mathbf{x}) &= \mathbb{E}\left[f(\mathbf{x})\right],\\ \Sigma(\mathbf{x}, \mathbf{x}') &= \mathbb{E}\left[(f(\mathbf{x}) - \mu(\mathbf{x}))(f(\mathbf{x}') - \mu(\mathbf{x}'))\right].\end{aligned}$$

Since we assumed n observations obtained by d-dimensional input vectors, we define the training input as $X \in \mathbb{R}^{d\times n}$, which is also called design matrix. The training output for all n observations are denoted as $Y \in \mathbb{R}^n$. Similarly, we denote test input and output for $n_* \in \mathbb{N}$ evaluations as $X_* \in \mathbb{R}^{d\times n_*}$ and $Y_* \in \mathbb{R}^{n_*}$, respectively.

Prediction model with noise. For the expected values of Y and Y_* we introduce the following notation:

$$\mu := \mathbb{E}[Y] \quad \text{and} \quad \mu_* := \mathbb{E}[Y_*].$$

We assume noisy observations with noise variance σ_n^2 such that the covariance of Y_1 is given as

$$\mathrm{Cov}(y_i, y_j) = k(\mathbf{x}_i, \mathbf{x}_j) + \sigma_n^2 \delta_{ij} \qquad \text{and} \qquad \mathrm{Cov}(Y, Y) = K(X, X) + \sigma_n^2 I, \tag{1.1}$$

where the kernel matrix K is defined as

$$K(X, X') := \big(k(\mathbf{x}_i, \mathbf{x}'_j)\big)_{i,j},$$

for a given kernel function $k : \mathbb{R}^d \times \mathbb{R}^d \to \mathbb{R}$, e.g. the Gaussian kernel function $k_\sigma(\mathbf{x}, \mathbf{x}') = \exp\left(-\frac{\|\mathbf{x}-\mathbf{x}'\|^2}{2\sigma}\right)$ with $\sigma > 0$.

Likewise, we define the following covariance matrices:

$$\begin{aligned} \mathrm{Cov}(Y, Y_*) &= \mathrm{Cov}(Y_*, Y)^\mathsf{T} = K(X, X_*), \qquad (1.2)\\ \mathrm{Cov}(Y_*, Y_*) &= K(X_*, X_*). \end{aligned}$$

Finally, we assume that the training outputs Y and test outputs Y_* are jointly normally distributed:

$$\begin{pmatrix} Y \\ Y_* \end{pmatrix} \sim \mathcal{N}_{n+n_*}\left(\begin{pmatrix} \mu \\ \mu_* \end{pmatrix}, \begin{bmatrix} K(X,X) + \sigma_n^2 I & K(X, X_*) \\ K(X_*, X) & K(X_*, X_*) \end{bmatrix}\right).$$

By using the abbreviated notation Σ, Σ_* and Σ_{**}, we obtain:

$$\begin{pmatrix} Y \\ Y_* \end{pmatrix} \sim \mathcal{N}_{n+n_*}\left(\begin{pmatrix} \mu \\ \mu_* \end{pmatrix}, \begin{bmatrix} \Sigma & \Sigma_* \\ \Sigma_*^\mathsf{T} & \Sigma_{**} \end{bmatrix}\right).$$

Since we are interested in inferring the relationship between inputs and function evaluations, the conditional distribution of the test output Y_* given the training output Y and inputs X and X_* is of relevance. This conditional distribution is also normally distributed

$$Y_* \mid Y \sim \mathcal{N}_{n_*}\left(\tilde{\mu}, \tilde{\Sigma}\right),$$

with mean and covariance defined as

$$\tilde{\mu} = \mathbb{E}(Y_* \mid Y) = \mu_* + \Sigma_*^\mathsf{T} \Sigma^{-1} (Y - \mu) \qquad (1.3)$$

$$\tilde{\Sigma} = \mathbb{V}(Y_* \mid Y) = \Sigma_{**} - \Sigma_*^\mathsf{T} \Sigma^{-1} \Sigma_*. \qquad (1.4)$$

Choice of hyperparameters via maximized likelihood. Consider the normally distributed observations $Y \sim \mathcal{N}_n(\mu(\vartheta), \Sigma(\vartheta))$ depending on parameters $\vartheta \in \Theta \subset \mathbb{R}^{n_\theta}, n_\theta \in \mathbb{N}$. To estimate the parameters, we maximize the likelihood function $L_\mathbf{y} : \Theta \to \mathbb{R}_0^+$, $L_\mathbf{y}(\vartheta) := p_\vartheta(\mathbf{y})$. The density function of the normally distributed random variable Y is given by:

$$p_\vartheta(\mathbf{y}) := \frac{1}{\sqrt{(2\pi)^n |\Sigma|}} \exp\left(-\frac{1}{2}(\mathbf{y} - \mu)^\mathsf{T} \Sigma^{-1} (\mathbf{y} - \mu)\right),$$

where $\mu := \mu(\vartheta)$ and $\Sigma := \Sigma(\vartheta)$. The log likelihood is then given by

$$\ell_\mathbf{y}(\vartheta) := \log L_\mathbf{y}(\vartheta) = -\frac{1}{2}\log|\Sigma| - \frac{1}{2}(\mathbf{y} - \mu)^\mathsf{T}\Sigma^{-1}(\mathbf{y} - \mu) - \frac{n}{2}\log(2\pi). \qquad (1.5)$$

Assuming separate parameters ϑ_μ and ϑ_Σ impacting the expectation $\mu := \mu(\vartheta_\mu)$ and the covariance $\Sigma := \Sigma(\vartheta_\Sigma)$, the following derivatives are of relevance for maximizing the likelihood

$$\frac{\partial \ell_{\mathbf{y}}}{\partial \vartheta_\mu} = (\mathbf{y} - \mu)^{\mathsf{T}} \Sigma^{-1} \frac{\partial \mu}{\partial \vartheta_\mu} \quad \text{and} \tag{1.6}$$

$$\frac{\partial \ell_{\mathbf{y}}}{\partial \vartheta_\Sigma} = -\frac{1}{2} \mathrm{tr}\left(\Sigma^{-1} \frac{\partial \Sigma}{\partial \vartheta_\Sigma}\right) + \frac{1}{2} (\mathbf{y} - \mu)^{\mathsf{T}} \Sigma^{-1} \frac{\partial \Sigma}{\partial \vartheta_\Sigma} \Sigma^{-1} (\mathbf{y} - \mu). \tag{1.7}$$

1.2 Interpolation model of cell outlines

In this section, we describe how to use the Gaussian process regression to interpolate cell outlines based on a discrete set of segmentation points. Here, we consider only a single contour Γ_k; for ease of notation, we omit the subscript k in the sequel. Let $(x_0, y_0), \ldots, (x_{M-1}, y_{M-1})$ denote $M \in \mathbb{N}$ segmentation points with periodic boundaries $x_M := x_0$ and $y_M := y_0$ since we describe cell contours by closed curves. The idea is to use the arc length of these curves as parametrization to obtain corresponding coordinates in $\mathbb{R}^2$. This parametrization of the contour Γ will be denoted as

$$\Phi : [0, 2\pi) \ni \theta \mapsto (x(\theta), y(\theta)) \in \mathbb{R}^2 \tag{1.8}$$

First, we define support points $\theta_i,\ i \in \{0, 1, \ldots, M-1\}$

$$\theta_m = \frac{2\pi \sum_{i=0}^{m} d_i}{\sum_{i=0}^{M-1} d_i} \quad \text{and} \quad d_0 = 0 \tag{1.9}$$

according to the normalized secant length along the contour

$$d_i = \left((x_i - x_{i-1})^2 + (y_i - y_{i-1})^2\right)^{1/2}$$

It is easy to see that the sequence of support points obtained by Eq. (1.9) is striclty increasing, i.e. $\theta_0 < \theta_1 < \cdots < \theta_{M-1}$.

Now, we apply Gaussian process regression to this sequence of support points in order to obtain a smoothing spline representing the cell contour. The underlying kernel function, necessary to model the correlation between the data points, is defined by

$$k_r(\theta, \theta') := \frac{1 - r^2}{1 - 2r\ \cos(\theta - \theta') + r^2}, \qquad \theta, \theta' \in [0, 2\pi), \tag{1.10}$$

with radius parameter $r \in (0, 1)$. This kernel function, which is closely related to the wrapped Cauchy distribution, is often called **Poisson kernel function**. We have chosen the Poisson kernel due to its periodicity property, i.e. $k_r(\theta, \theta') = k_r(\theta + 2z\pi, \theta')$ for all $z \in \mathbb{Z}$. Alternatively, one could periodize

a non-periodic kernel function $k(\cdot,\cdot)$, e.g. the Gaussian kernel function, by applying the following formula:

$$k_{\text{per}}(\theta,\theta') := \sum_{z\in\mathbb{Z}} k(\theta + 2z\pi, \theta').$$

We simplify the regression model by assuming that x and y coordinates are independent from each other. For this reason, we perform the GPR two times in order to obtain two regression functions f_x and f_y satisfying $f_x(\theta_i) \approx x_i$ and $f_y(\theta_i) \approx y_i$ for all $i \in \{0,\dots,M-1\}$. By deducing these regression functions, we can compute the two-dimensional coordinates for any test input $\tilde{\theta}_j \in [0,2\pi)$ with $j \in \{0,\dots,M_*-1\}$ and $M_* \in \mathbb{N}$ representing the number of function evaluations.

In the following, we describe how to obtain the x coordinates $\tilde{\mathbf{x}} := (\tilde{x}_0,\dots,\tilde{x}_{M_*-1})$ given the training input $\{\theta_j \mid j = 0,\dots,M-1\}$, the training output $\mathbf{x} := (x_0,\dots,x_{M-1})$ and the testing input $\{\tilde{\theta}_j \mid j = 0,\dots,M_*-1\}$. The GPR can be similarly used to obtain the y coordinates. First, we consider a prediction model with noisy observations and no drift, i.e. $\mathbb{E}[\mathbf{x}] = \mathbb{E}[\tilde{\mathbf{x}}] = 0$. The covariances are given by the Eq. (1.1) and (1.2), where the underlying kernel matrix $K(\boldsymbol{\theta},\boldsymbol{\theta'}) := \left(k(\theta_i,\theta'_j)\right)_{i,j}$ is given by the Poisson kernel from Eq. (1.10). Since the Gaussian processes is centered, the GPR prediction equation is simply given by:

$$\begin{aligned} \mathbb{E}(\tilde{\mathbf{x}} \mid \mathbf{x}) &= \Sigma_*^{\mathsf{T}}\Sigma^{-1}\mathbf{x} \qquad \text{and} \\ \mathbb{V}(\tilde{\mathbf{x}} \mid \mathbf{x}) &= \Sigma_{**} - \Sigma_*^{\mathsf{T}}\Sigma^{-1}\Sigma_*. \end{aligned} \tag{1.11}$$

Since the covariance matrices depends on the input data consisting of the normalized arc length parametrization, we introduce the following notation:

$$\begin{aligned} f_x(\tilde{\boldsymbol{\theta}}) &:= \mathbb{E}(\tilde{\mathbf{x}} \mid \mathbf{x}; \tilde{\boldsymbol{\theta}}, \boldsymbol{\theta}) = \Sigma_*^{\mathsf{T}}(\boldsymbol{\theta},\tilde{\boldsymbol{\theta}}) \cdot \Sigma^{-1}(\boldsymbol{\theta}) \cdot \mathbf{x}, \\ f_y(\tilde{\boldsymbol{\theta}}) &:= \mathbb{E}(\tilde{\mathbf{y}} \mid \mathbf{y}; \tilde{\boldsymbol{\theta}}, \boldsymbol{\theta}) = \Sigma_*^{\mathsf{T}}(\boldsymbol{\theta},\tilde{\boldsymbol{\theta}}) \cdot \Sigma^{-1}(\boldsymbol{\theta}) \cdot \mathbf{y}. \end{aligned} \tag{1.12}$$

Although we obtained a space of functions with a probability distribution based on $\mathbb{E}(\tilde{\mathbf{x}} \mid \mathbf{x})$ and $\mathbb{V}(\tilde{\mathbf{x}} \mid \mathbf{x})$, the regression functions $f_x : [0,2\pi)^{M_*} \to \mathbb{R}^{M_*}$ and $f_y : [0,2\pi)^{M_*} \to \mathbb{R}^{M_*}$ are defined only by the expectation.

1.3 Computation of curvature using GPR

In this section, we highlight the benefits of using the GPR to obtain smooth cell outlines given a discrete data set. Given a two dimensional curve

$$\begin{aligned} \gamma &: [0,2\pi) \to \mathbb{R}^2, \\ &\theta \mapsto \gamma(\theta) := \begin{pmatrix} f_x(\theta) \\ f_y(\theta) \end{pmatrix} \end{aligned}$$

with corresponding coordinate functions $f_x : [0, 2\pi) \to \mathbb{R}$ and $f_y : [0, 2\pi) \to \mathbb{R}$, the curvature at $\gamma(\theta)$ is denoted as $\kappa(\theta)$ and defined by

$$\kappa(\theta) := \frac{f_x' f_y'' - f_y' f_x''}{\left({f_x'}^2 + {f_y'}^2\right)^{\frac{3}{2}}}, \qquad \theta \in [0, 2\pi). \tag{1.13}$$

Since the curve coordinates are given by the GPR prediction equations in Eq. (1.12), we can easily compute the first and second derivative of f_x:

$$f_x'(\tilde{\boldsymbol{\theta}}) = \frac{\partial}{\partial \tilde{\boldsymbol{\theta}}} \Sigma_*^{\mathsf{T}}(\boldsymbol{\theta}, \tilde{\boldsymbol{\theta}}) \cdot \Sigma^{-1}(\boldsymbol{\theta}) \cdot \mathbf{x} \quad \text{and} \quad f_x''(\tilde{\boldsymbol{\theta}}) = \frac{\partial^2}{\partial \tilde{\boldsymbol{\theta}}^2} \Sigma_*^{\mathsf{T}}(\boldsymbol{\theta}, \tilde{\boldsymbol{\theta}}) \cdot \Sigma^{-1}(\boldsymbol{\theta}) \cdot \mathbf{x}, \tag{1.14}$$

and similarly for f_y:

$$f_y'(\tilde{\boldsymbol{\theta}}) = \frac{\partial}{\partial \tilde{\boldsymbol{\theta}}} \Sigma_*^{\mathsf{T}}(\boldsymbol{\theta}, \tilde{\boldsymbol{\theta}}) \cdot \Sigma^{-1}(\boldsymbol{\theta}) \cdot \mathbf{y} \quad \text{and} \quad f_y''(\tilde{\boldsymbol{\theta}}) = \frac{\partial^2}{\partial \tilde{\boldsymbol{\theta}}^2} \Sigma_*^{\mathsf{T}}(\boldsymbol{\theta}, \tilde{\boldsymbol{\theta}}) \cdot \Sigma^{-1}(\boldsymbol{\theta}) \cdot \mathbf{y}. \tag{1.15}$$

The partial derivative $\frac{\partial}{\partial \tilde{\boldsymbol{\theta}}} \Sigma_*^{\mathsf{T}}(\boldsymbol{\theta}, \tilde{\boldsymbol{\theta}})$ is given by the partial derivative of the Poisson kernel function k_r. More precisely, we obtain the following equations:

$$\begin{aligned} \frac{\partial}{\partial \tilde{\boldsymbol{\theta}}} \Sigma_*^{\mathsf{T}}(\boldsymbol{\theta}, \tilde{\boldsymbol{\theta}}) &= \frac{\partial}{\partial \tilde{\boldsymbol{\theta}}} K(\tilde{\boldsymbol{\theta}}, \boldsymbol{\theta}) = \left(\frac{\partial}{\partial \tilde{\theta}_i} k_r(\tilde{\theta}_i, \theta_j) \right)_{i,j} \qquad \text{and} \\ \frac{\partial^2}{\partial \tilde{\boldsymbol{\theta}}^2} \Sigma_*^{\mathsf{T}}(\boldsymbol{\theta}, \tilde{\boldsymbol{\theta}}) &= \frac{\partial^2}{\partial \tilde{\boldsymbol{\theta}}^2} K(\tilde{\boldsymbol{\theta}}, \boldsymbol{\theta}) = \left(\frac{\partial^2}{\partial \tilde{\theta}_i^2} k_r(\tilde{\theta}_i, \theta_j) \right)_{i,j} . \end{aligned}$$

For the Poisson kernel function in Eq. (1.10), the first and second partial derivative are given by:

$$\begin{aligned} \frac{\partial}{\partial \tilde{\theta}} k_r(\tilde{\theta}, \theta) &= \frac{2r\left(r^2 - 1\right) \sin(\tilde{\theta} - \theta)}{\left(r^2 - 2r \ \cos(\tilde{\theta} - \theta) + 1\right)^2} \qquad \text{and} \\ \frac{\partial^2}{\partial \tilde{\theta}^2} k_r(\tilde{\theta}, \theta) &= \frac{2r(r^2 - 1)\Big((r^2 + 1)\cos(\tilde{\theta} - \theta) - 2r \ \cos^2(\tilde{\theta} - \theta) - 4r \ \sin^2(\tilde{\theta} - \theta)\Big)}{\left(r^2 - 2r \ \cos(\tilde{\theta} - \theta) + 1\right)^3}. \end{aligned}$$

In order to compute f_x' and f_x'', we have to substitute the first covariance matrix in Eq. (1.12) with the covariance matrix generated by the first and second derivative of the kernel function, respectively. Likewise to f_x, we can evaluate the derivatives f_x' and f_x'' at every position $\tilde{\theta}$. The same holds true for f_y. Thereby we found an analytic way to compute the curvature κ via Eq. (1.13).

1.4 Parameter estimation

In this section, we present a short overview how to choose the hyperparameters r and σ_n used to obtain smooth contours based on GPR. In this context, we

maximize the log likelihood function presented in Section 1.1

$$\ell_{\mathbf{y}}(\vartheta) := \log L_{\mathbf{y}}(\vartheta) = -\frac{1}{2}\log|\Sigma| - \frac{1}{2}(\mathbf{y}-\mu)^{\mathsf{T}}\Sigma^{-1}(\mathbf{y}-\mu) - \frac{n}{2}\log(2\pi). \quad (1.16)$$

Recall that the GPR model is based on centered Gaussian processes (i.e. $\mu = 0$) and that both hyperparameters influence the covariance matrix Σ only. Hence, we obtain the following formula of the partial derivative of the log likelihood function

$$\frac{\partial \ell_{\mathbf{y}}}{\partial \vartheta_\Sigma} = -\frac{1}{2}\mathrm{tr}\left(\Sigma^{-1}\frac{\partial \Sigma}{\partial \vartheta_\Sigma}\right) + \frac{1}{2}\mathbf{y}^{\mathsf{T}}\Sigma^{-1}\frac{\partial \Sigma}{\partial \vartheta_\Sigma}\Sigma^{-1}\mathbf{y} \quad (1.17)$$

with $\vartheta_\Sigma \in \{r, \sigma_n\}$. Furthermore, $\partial\Sigma/\partial\vartheta_\Sigma$ is given by the following formulas

$$\frac{\partial \Sigma}{\partial r} = \frac{\partial}{\partial r}\left(K(\boldsymbol{\theta},\boldsymbol{\theta}) + \sigma_n^2 I\right) = \left(\frac{\partial}{\partial r}k_r(\theta_i,\theta_j)\right)_{i,j} \quad \text{and}$$

$$\frac{\partial \Sigma}{\partial \sigma_n} = \frac{\partial}{\partial \sigma_n}\left(K(\boldsymbol{\theta},\boldsymbol{\theta}) + \sigma_n^2 I\right) = 2\sigma_n I$$

with corresponding derivative of the Poisson kernel function

$$\frac{\partial k_r(\theta,\theta')}{\partial r} = \frac{2(r^2+1)\cos(\theta-\theta') - 4r}{(1-2r\,cos(\theta-\theta')+r^2)^2}.$$

Now, we can determine the maximum log likelihood estimate using gradient descent with the above derivatives in order to estimate the hyperparameters. In Fig 1, we present estimated parameters based on the persistently motile cell example as in the main article. In panel (A), pairs of estimated hyperparameters (r, σ_n) are shown for different contours ($K = 500$). The gradient descent method was applied to each contour separately in order to obtain one pair of hyperparameters.

Two clusters can be observed, which corresponds to the bimodal distributions of r and σ_n shown in panel (B) and (C). Furthermore, the total log-likelihood, i.e., the sum of the log-likelihood functions of all contours, is presented in panel (D). The maximum at $r = 0.80$ and $\sigma_n = 0.017$ is depicted as black dot. These parameters were used in all computations in the main article.

Partial derivatives of $\ell_{\mathbf{y}}(\vartheta)$. Let $Y \sim \mathcal{N}_k(\mu, \Sigma)$ be normally distributed with parametric dependencies $\mu = \mu(\vartheta_\mu)$ and covariance matrix $\Sigma = \Sigma(\vartheta_\Sigma)$. Then, the partial derivatives of the log likelihood function are described by:

$$\frac{\partial \ell_{\mathbf{y}}}{\partial \vartheta_\mu} = (\mathbf{y}-\mu)^{\mathsf{T}}\Sigma^{-1}\frac{\partial \mu}{\partial \vartheta_\mu}, \quad (1.18)$$

$$\frac{\partial \ell_{\mathbf{y}}}{\partial \vartheta_\Sigma} = -\frac{1}{2}\mathrm{tr}\left(\Sigma^{-1}\frac{\partial \Sigma}{\partial \vartheta_\Sigma}\right) + \frac{1}{2}(\mathbf{y}-\mu)^{\mathsf{T}}\Sigma^{-1}\frac{\partial \Sigma}{\partial \vartheta_\Sigma}\Sigma^{-1}(\mathbf{y}-\mu). \quad (1.19)$$

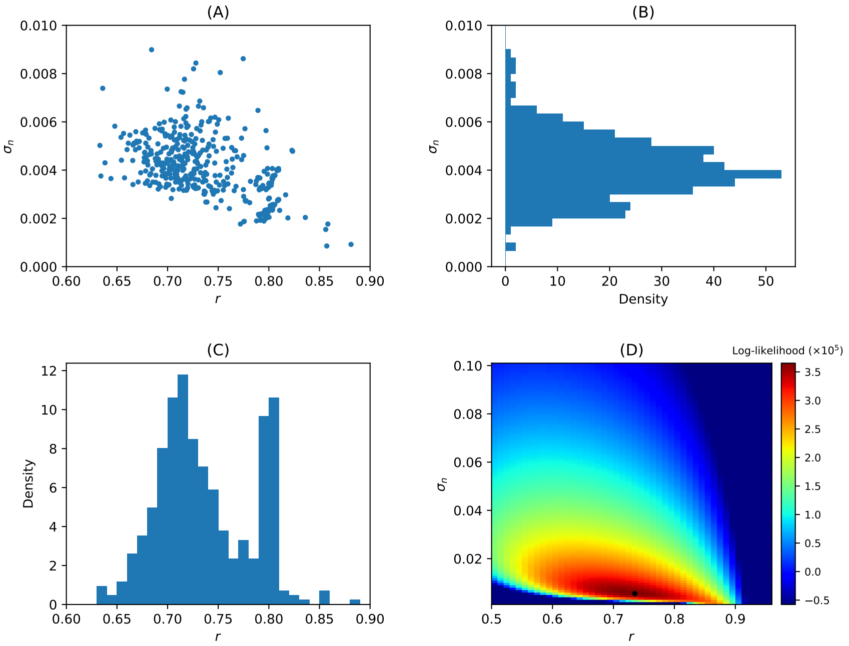

Figure 1. Estimation of hyperparameters for example cell track. **(A)** $K = 500$ Pairs of parameters (r, σ_n) estimated by maximum likelihood for each contour. **(B, C)** Corresponding histograms for σ_n and r, respectively. **(D)** Total log-likelihood of all $K = 500$ contours as a function of the hyperparameters. Maximum is displayed as black dot.

This can be seen as follows: At first, by using basic mathematical operations, we can show that:

$$
\begin{aligned}
\frac{\partial \ell_{\mathbf{y}}}{\partial \vartheta_\mu} &= 0 + \frac{\partial}{\partial \vartheta_\mu}\left(-\frac{1}{2}(\mathbf{y}-\mu)^\mathsf{T}\Sigma^{-1}(\mathbf{y}-\mu)\right) + 0 \\
&= \frac{1}{2}\frac{\partial \mu}{\partial \vartheta_\mu}^\mathsf{T}\Sigma^{-1}(\mathbf{y}-\mu) + \frac{1}{2}(\mathbf{y}-\mu)^\mathsf{T}\Sigma^{-1}\frac{\partial \mu}{\partial \vartheta_\mu} \\
&= \frac{1}{2}\left(\frac{\partial \mu}{\partial \vartheta_\mu}^\mathsf{T}\Sigma^{-1}(\mathbf{y}-\mu)\right)^\mathsf{T} + \frac{1}{2}(\mathbf{y}-\mu)^\mathsf{T}\Sigma^{-1}\frac{\partial \mu}{\partial \vartheta_\mu} \\
&= \frac{1}{2}(\mathbf{y}-\mu)^\mathsf{T}\left(\Sigma^{-1}\right)^\mathsf{T}\frac{\partial \mu}{\partial \vartheta_\mu} + \frac{1}{2}(\mathbf{y}-\mu)^\mathsf{T}\Sigma^{-1}\frac{\partial \mu}{\partial \vartheta_\mu} \\
&\overset{1}{=} (\mathbf{y}-\mu)^\mathsf{T}\Sigma^{-1}\frac{\partial \mu}{\partial \vartheta_\mu}.
\end{aligned}
$$

Now, we consider the following identities of matrix derivatives:

$$\frac{dA^{-1}}{d\vartheta} = -A^{-1}\frac{dA}{d\vartheta}A^{-1} \qquad \text{and} \qquad \frac{d\,|A|}{d\vartheta} = |A|\ \mathrm{tr}\left(A^{-1}\frac{dA}{d\vartheta}\right), \tag{1.20}$$

where $A = A(\vartheta)$ is an arbitrary matrix depending on a parameter ϑ. By using these identities, it is easy to show that:

$$\begin{aligned}\frac{\partial \ell_{\mathbf{y}}}{\partial \vartheta_\Sigma} &= -\frac{1}{2}\frac{\partial}{\partial \vartheta_\Sigma}\left(\log|\Sigma|\right) - \frac{1}{2}\frac{\partial}{\partial \vartheta_\Sigma}\left((\mathbf{y}-\mu)^\mathsf{T}\Sigma^{-1}(\mathbf{y}-\mu)\right)\\ &= -\frac{1}{2\,|\Sigma|}|\Sigma|\ \mathrm{tr}\left(\Sigma^{-1}\frac{\partial\Sigma}{\partial\vartheta_\Sigma}\right) - \frac{1}{2}\left(-(\mathbf{y}-\mu)^\mathsf{T}\Sigma^{-1}\frac{\partial\Sigma}{\partial\vartheta_\Sigma}\Sigma^{-1}(\mathbf{y}-\mu)\right)\\ &= -\frac{1}{2}\mathrm{tr}\left(\Sigma^{-1}\frac{\partial\Sigma}{\partial\vartheta_\Sigma}\right) + \frac{1}{2}(\mathbf{y}-\mu)^\mathsf{T}\Sigma^{-1}\frac{\partial\Sigma}{\partial\vartheta_\Sigma}\Sigma^{-1}(\mathbf{y}-\mu).\end{aligned}$$

2 Regularized contour flow method (RCFM)

In this section, we present further details of the regularized contour flow method in addition to the main article. First, we provide information how the RCFM is implemented in `AmoePy`, our Python-based toolbox for analyzing and simulating amoeboid cell motility [6]. Subsequently, we describe the gradient descent method which is used to solve the minimization problem from Eq. (22) of the main article. Here, we describe how the Gaussian processes regression can also be used to obtain the gradient of the functional w.r.t. to its arguments (virtual markers) analytically. This will reduce the number of iterations of the gradient descent method and therefore reduce the computational cost of the algorithm substantially.

Furthermore, we present test cases which were used to validate the RCFM. These test cases as well as multiple experimental cell tracks are fully accessible in our `AmoePy` software package. In Fig 2, a current version (v1.1) of `AmoePy` is shown.

Finally, in section 2.2, we provide the step-by-step derivations for some of the statements presented in the method section of the main article.

2.1 Implementation of regularized flows

Objective function. Note that the optimal flow is defined as the flow that solves the following minimization problem:

$$\phi_{k,\lambda} = \underset{\phi_k}{\mathrm{argmin}}\ F_k[\phi_k] + \lambda U_k[\phi_k], \quad \lambda > 0. \tag{2.1}$$

where the two functionals are given by:

$$F_k[\phi_k] \simeq F_k\left[\theta_{k+1}|\theta_k\right] = \frac{1}{N\delta t^2}\sum_{i=0}^{N-1}\left\|\Phi_{k+1}(\theta_{k+1,i}) - \Phi_k(\theta_{k,i})\right\|^2 \tag{2.2}$$

[1] $\left(\Sigma^{-1}\right)^\mathsf{T} = \left(\Sigma^\mathsf{T}\right)^{-1} = \Sigma^{-1}$ due to the symmetry of Σ.

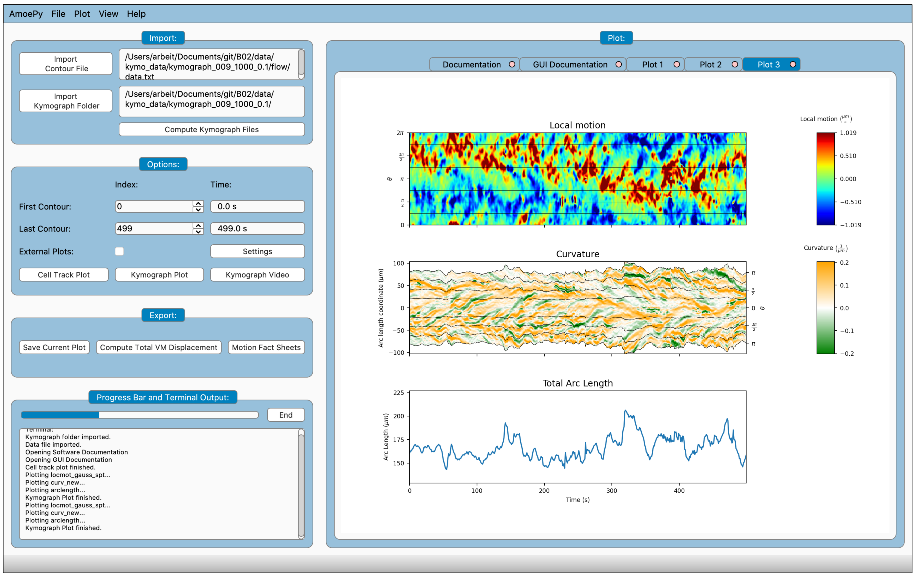

Figure 2. Graphical user interface of `AmoePy` providing multiple analysis routines as described in the main article. Additionally, `AmoePy` contains several plotting tools in order to generate graphics and videos from cell tracks and their kymographs. Finally, a detailed documentation of the underlying source codes is accessible via the GUI.

$$U_k[\phi_k] \simeq U_k\left[\theta_{k+1}|\theta_k\right] = N \sum_{i=0}^{N-1} \left|\theta_{k+1,i+1} - \theta_{k+1,i}\right|^2. \tag{2.3}$$

where $\theta_{k+1},\ \theta_k \in [0, 2\pi)^N$ denote the normalized arc length coordinates for N segmentation points at time t_k and t_{k+1}. For two consecutive parametrizations θ_{k+1} and θ_k and regularization parameter $\lambda \in \mathbb{R}^+$, the objective function $H_k[\phi_k] \coloneqq F_k[\phi_k] + \lambda U_k[\phi_k]$ is given by

$$\begin{aligned} H_k[\phi_k] \simeq H_k\left[\theta_{k+1}|\theta_k\right] = \frac{1}{N\delta t^2} \sum_{i=0}^{N-1} \left\|\Phi_{k+1}(\theta_{k+1,i}) - \Phi_k(\theta_{k,i})\right\|^2 \\ + \lambda N \sum_{i=0}^{N-1} \left|\theta_{k+1,i+1} - \theta_{k+1,i}\right|^2. \end{aligned} \tag{2.4}$$

From the following optimization problem

$$\theta_{k+1} \coloneqq \underset{\tilde{\theta}_{k+1} \in [0,2\pi)^M}{\operatorname{argmin}} \ H_k\left[\tilde{\theta}_{k+1}|\theta_k\right], \tag{2.5}$$

we obtain the arc length parametrization of the next contour.

Derivatives of objective function. In order to solve the optimization problem, we used built-in gradient descent methods from the Python package `SciPy`. In this context, we have chosen `'L-BFGS-B'` as minimizer due to its short running time and usage of bound constraints. Alternatively, `'trust-constr'` offers constrained minimization with acceptable running times as well. See [7, 8] and [9, 10] for more details about both minimizers. The Jacobian and Hessian of H_k, necessary for some custom minimizers, are given by the following formulas:

$$\frac{\partial H_k}{\partial \theta_{k+1,i}} = 2a \cdot \left\langle \Phi_{k+1}\left(\theta_{k+1,i}\right) - \Phi_k\left(\theta_{k,i}\right),\, \frac{\partial \Phi_{k+1}}{\partial \theta_{k+1,i}} \right\rangle$$

$$- 2b \cdot \left(\theta_{k+1,i-1} + \theta_{k+1,i+1}\right) + 4b \cdot \theta_{k+1,i}$$

$$\frac{\partial^2 H_k}{\partial \theta_{k+1,i}^2} = 2a \cdot \left\langle \Phi_{k+1}\left(\theta_{k+1,i}\right) - \Phi_k\left(\theta_{k,i}\right),\, \frac{\partial^2 \Phi_{k+1}}{\partial \theta_{k+1,i}^2} \right\rangle$$

$$2a \cdot \left\| \frac{\partial \Phi_{k+1}}{\partial \theta_{k+1,i}} \right\|^2 + 4b,$$

$$\frac{\partial^2 H_k}{\partial \theta_{k+1,i}\,\partial \theta_{k+1,j}} = \begin{cases} -2b & |i-j| = 1, \\ 0 & 1 < |i-j| < N-1, \\ -2b & |i-j| = N-1, \end{cases}$$

where $a := 1/(N\delta t^2)$ and $b := \lambda N$ and $i, j \in \{0,\ \ldots,\ N-1\}$. Conveniently, the derivatives $\partial \Phi_{k+1}/\partial \theta_{k+1,i}$ and $\partial^2 \Phi_{k+1}/\partial \theta_{k+1,i}^2$ are a by-product from the Gaussian process regression obtained by Eq. (1.14) and (1.15). See also

Linear constraints. In order to avoid mapping violations, one can choose a gradient descent method with following constraints

$$\begin{aligned} I:&\quad 0 \leq \theta_{k,j+1} - \theta_{k,j} \leq 2\pi \qquad \text{and} \\ II:&\quad 0 \leq \theta_{k,N-1} - \theta_{k,0} \leq 2\pi. \end{aligned}$$

Both inequalities can be summarized by the following linear constraint:

$$\begin{pmatrix} 0 \\ \vdots \\ 0 \\ -2\pi \end{pmatrix} \leq \begin{pmatrix} -1 & 1 & & \\ & \ddots & \ddots & \\ & & \ddots & 1 \\ 1 & & & -1 \end{pmatrix} \cdot \begin{pmatrix} \theta_{k,0} \\ \theta_{k,1} \\ \vdots \\ \theta_{k,N-1} \end{pmatrix} \leq \begin{pmatrix} 2\pi \\ \vdots \\ 2\pi \\ 0 \end{pmatrix}.$$

Since the second functional in Eq. (2.1) already penalizes mapping violations, i.e. $\theta_{k,i+1} - \theta_{k,i} \leq 0$ for $i \in \{0,\ \ldots,\ N-1\}$, we used a gradient descent method with smaller computation time where the check of linear constraint violations is skipped.

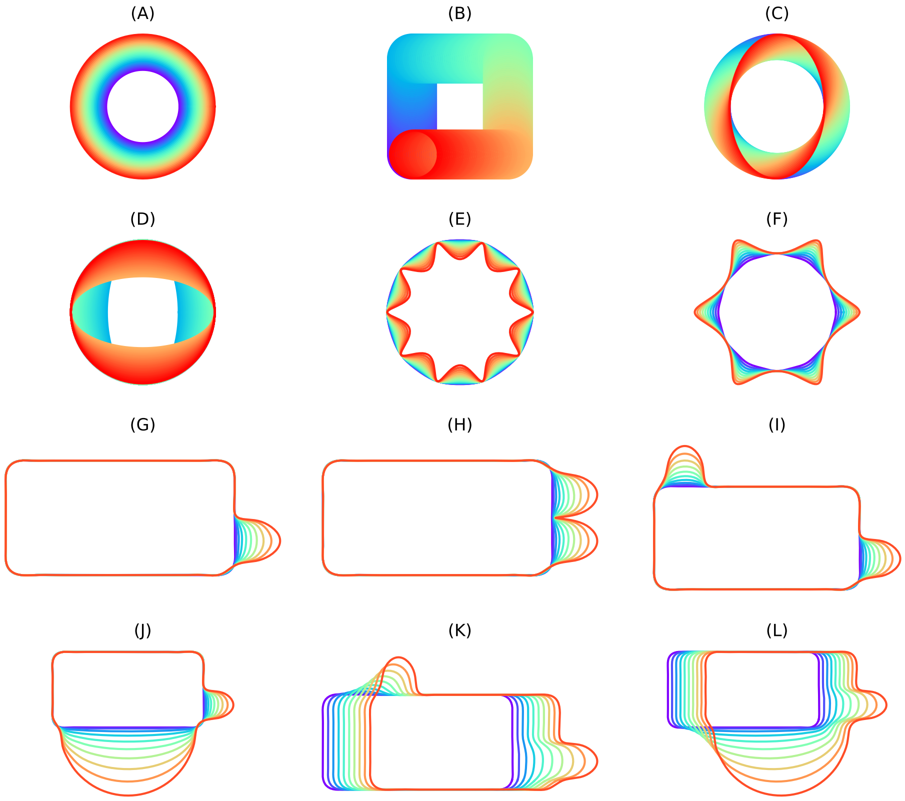

Figure 3. Selection of test cases accessible in `AmoePy`: Pulsating circle **(A)**, translation of circle **(B)**, rotating ellipse **(C)**, circle to ellipse transformation **(D)**, inward membrane changes **(E)**, outward membrane changes **(F)**, single protrusion **(G)**, neighboring protrusions **(H)**, distant protrusions **(I)**, different sized protrusions and different protrusions during translation **(K, L)**. The period of time of each excerpt is color-coded from purple to red.

Method validation on test cases. Our software package `AmoePy` provides several simple contour tracks as displayed in Fig 3. These test cases consist of basic geometric transformations (A-F) and different protrusion patterns (G-L). Many tracks are periodic to further challenge the algorithm; for periodic tracks, we only show one period of the track in 2d in Fig 3, while the kymographs are shown for all period in S4 Fig. The starting contour is highlighted in purple, whereas the last contour is highlighted in red. The exact time intervals of each track can be extracted from S4 Fig.

The kymographs of the test cases nicely illustrates special features of the local dispersion. In the example case of the pulsating circle (A), the local dispersion is equal to zero for the entire contour and time interval, since the

local dispersion was defined as a concentration/stretching rate of the marker arc length coordinates on the unit circle $\mathcal{S}^1$. Moreover, cases (G-L) also illustrate a non-local feature of the marker dispersion: Strong protrusions at one part of the contour may also affect the dispersion of markers at the rest of the contour. In `AmoePy`, a detailed documentation is included which can be used to replicate the corresponding kymographs as well as animated videos of these test cases.

2.2 Supporting computations

(I) Cost functional $\boldsymbol{U_k[\phi_k]}$. Recall that virtual markers along a contour Γ_k are distributed according to a density $\mu_k(\theta)$. The density of virtual markers on the consecutive contour induced by ϕ_k can be described with

$$\mu_{k+1}(\phi_k(\theta))d\theta = \frac{\mu_k(\theta)d\theta}{\partial_\theta \phi_k(\theta)}, \tag{2.6}$$

where the underlying flow is defined by

$$\theta_{k+1} = \phi_k(\theta_k)$$

The second functional, which quantifies the degree of non-uniformity, is defined by

$$U[\mu] := \int_0^{2\pi} \frac{\mathrm{d}\theta}{\mu(\theta)}. \tag{2.7}$$

By applying the substitution rule for integrals to $\mu_{k+1}(\phi_k(\theta))$, we obtain the following expression

$$U_k[\phi_k] = \int_0^{2\pi} \frac{\partial_\theta \phi_k(\theta)}{\mu_{k+1}(\phi_k(\theta))} \mathrm{d}\theta \overset{(2.6)}{=} \int_0^{2\pi} \frac{\partial_\theta \phi_k(\theta)^2}{\mu_k(\theta)} \mathrm{d}\theta. \tag{2.8}$$

(II) Cost functional $\boldsymbol{U_k[\phi_k]}$. Note that the mapping from Γ_0 to Γ_k is denoted as $\chi_k(\theta)$ and defined by

$$\chi_{k+1}(\theta) = \phi_k(\chi_k(\theta)), \quad \chi_0(\theta_0) = \theta_0.$$

Furthermore, the density of virtual markers along the cell contour Γ_k is defined by

$$\mu_k(\chi_k(\theta_0)) = \frac{1}{2\pi \cdot \partial_{\theta_0} \chi_k(\theta_0)}. \tag{2.9}$$

Then, we obtain the following expression

$$\begin{aligned} U_k[\mu] &:= \int_0^{2\pi} \frac{\mathrm{d}\theta}{\mu(\theta)} \\ &= \int_0^{2\pi} \frac{1}{\mu_k\left(\chi_k(\theta_0)\right)} \cdot \partial_{\theta_0} \chi_k(\theta_0) \mathrm{d}\theta_0 \end{aligned}$$

$$\overset{(2.9)}{=} 2\pi \int_0^{2\pi} |\partial_{\theta_0} \chi_k(\theta_0)|^2 \, \mathrm{d}\theta_0.$$

From there on, we can deduce the formula mentioned in the main article

$$\begin{aligned} U_k[\phi_k] &= 2\pi \int_0^{2\pi} |\partial_{\theta_0} \phi_k\left(\chi_k(\theta_0)\right)|^2 \, \mathrm{d}\theta_0 \\ &= 2\pi \int_0^{2\pi} |\partial_{\theta_0} \chi_{k+1}(\theta_0)|^2 \, \mathrm{d}\theta_0. \end{aligned}$$

Cost functional $F_k[\phi_k]$. By noticing that the square mean velocity of the flow is defined by

$$F_k[\phi_k] = \int_0^{2\pi} \|V_k(\theta)\|^2 \, \mu_k(\theta) \mathrm{d}\theta$$

and the translation vectors Φ, V, and W are linked by the following formula

$$\frac{\Phi_{k+1}(\phi_k(\chi_k(\theta_0))) - \Phi_k(\chi_k(\theta_0))}{\delta t} = V_k(\chi_k(\theta_0)) = W_k(\theta_0). \tag{2.10}$$

Again, by using the substitution formula for integral, we obtain the following expression of the first cost functional

$$\begin{aligned} F_k[\phi_k] &:= \int_0^{2\pi} \|V_k(\theta)\|^2 \, \mu_k(\theta) \mathrm{d}\theta \\ &= \int_0^{2\pi} \|V_k(\chi_k(\theta_0))\|^2 \, \mu_k(\chi_k(\theta_0)) \cdot \partial_{\theta_0} \chi_k(\theta_0) \mathrm{d}\theta_0 \\ &\overset{(2.9)}{=} \frac{1}{2\pi} \int_0^{2\pi} \|V_k(\chi_k(\theta_0))\|^2 \, \mathrm{d}\theta_0 \\ &\overset{(2.10)}{=} \frac{1}{2\pi} \int_0^{2\pi} \left\| \frac{\Phi_{k+1}(\phi_k(\chi_k(\theta_0))) - \Phi_k(\chi_k(\theta_0))}{\delta t} \right\|^2 \mathrm{d}\theta_0. \end{aligned}$$

References

[1] Rasmussen CE, Williams CKI. Gaussian Processes for Machine Learning. Adaptive Computation and Machine Learning. MIT Press; 2006.

[2] Bousquet O, von Luxburg U, Rätsch G. Advanced Lectures on Machine Learning. vol. 3176 of Lecture Notes in Computer Science. Springer, Berlin, Heidelberg; 2004.

[3] Williams CKI, Rasmussen CE. Gaussian processes for regression. Advances in Neural Processing Systems. 1996;8(June):514–520.

[4] Williams CKI. Prediction with Gaussian Processes: From Linear Regression to Linear Prediction and Beyond. In: Learning in Graphical Models. Kluwer; 1998. p. 599–621.

[5] Ambikasaran S, Foreman-Mackey D, Greengard L, Hogg DW, O'Neil M. Fast Direct Methods for Gaussian Processes. IEEE Transactions on Pattern Analysis and Machine Intelligence. 2016;38(2):252–265. doi:10.1109/TPAMI.2015.2448083.

[6] Schindler D, Moldenhawer T, Lindenmeier L, Holschneider M. AmoePy: A Python-based toolbox for analyzing and simulating amoeboid cell motility; 2020. Available from: `https://zenodo.org/record/3982371`.

[7] Byrd RH, Lu P, Nocedal J, Zhu C. A Limited Memory Algorithm for Bound Constrained Optimization. SIAM Journal on Scientific Computing. 1995;16(5):1190–1208. doi:10.1137/0916069.

[8] Zhu C, Byrd RH, Lu P, Nocedal J. Algorithm 778: L-BFGS-B: Fortran Subroutines for Large-Scale Bound-Constrained Optimization. ACM Transactions on Mathematical Software. 1997;23(4):550–560. doi:10.1145/279232.279236.

[9] Lalee M, Nocedal J, Plantenga T. On the implementation of an algorithm for large-scale equality constrained optimization. SIAM Journal on Optimization. 1998;8(3):682–706. doi:10.1137/S1052623493262993.

[10] Byrd RH, Hribar ME, Nocedal J. An interior point algorithm for large-scale nonlinear programming. SIAM Journal on Optimization. 1999;9(4):877–900. doi:10.1137/S1052623497325107.
