# Supplementary material for: Analysis of protrusion dynamics in amoeboid cell motility by means of regularized contour flows
Source: PLoS Comput Biol. 2021 Aug 23;17(8):e1009268. doi: 10.1371/journal.pcbi.1009268 (PMC8412247; doi:10.1371/journal.pcbi.1009268)
Supplement: S1 Fig — (PDF) [file pcbi.1009268.s002.pdf]

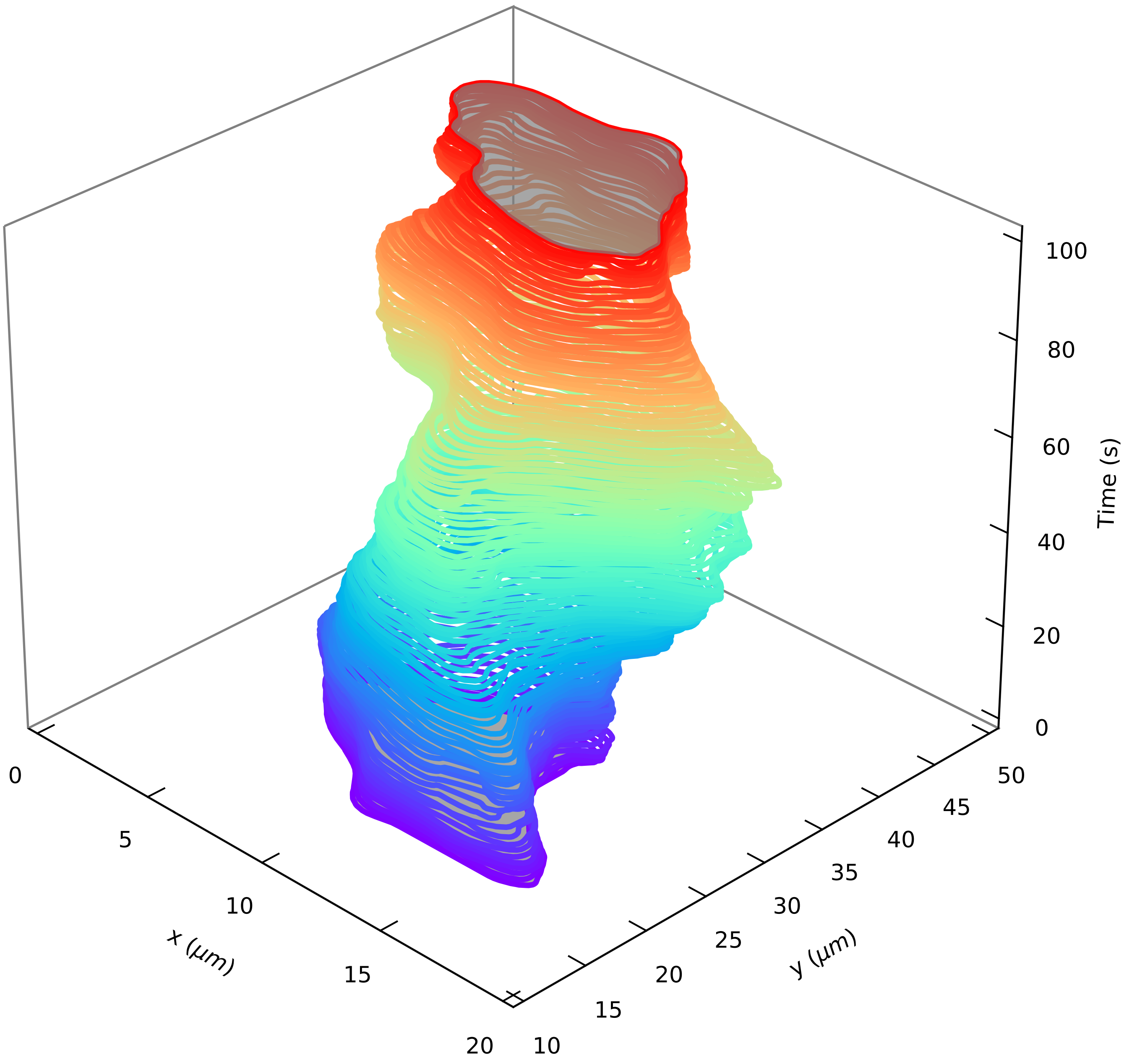

Fig S1. Three-dimensional presentation of cell contours (amoeba tube), where consecutive contours are stacked onto each other. The color of each contour coincides with the time scale.
