# Supplementary material for: Analysis of protrusion dynamics in amoeboid cell motility by means of regularized contour flows
Source: PLoS Comput Biol. 2021 Aug 23;17(8):e1009268. doi: 10.1371/journal.pcbi.1009268 (PMC8412247; doi:10.1371/journal.pcbi.1009268)
Supplement: S2 Fig — (PDF) [file pcbi.1009268.s003.pdf]

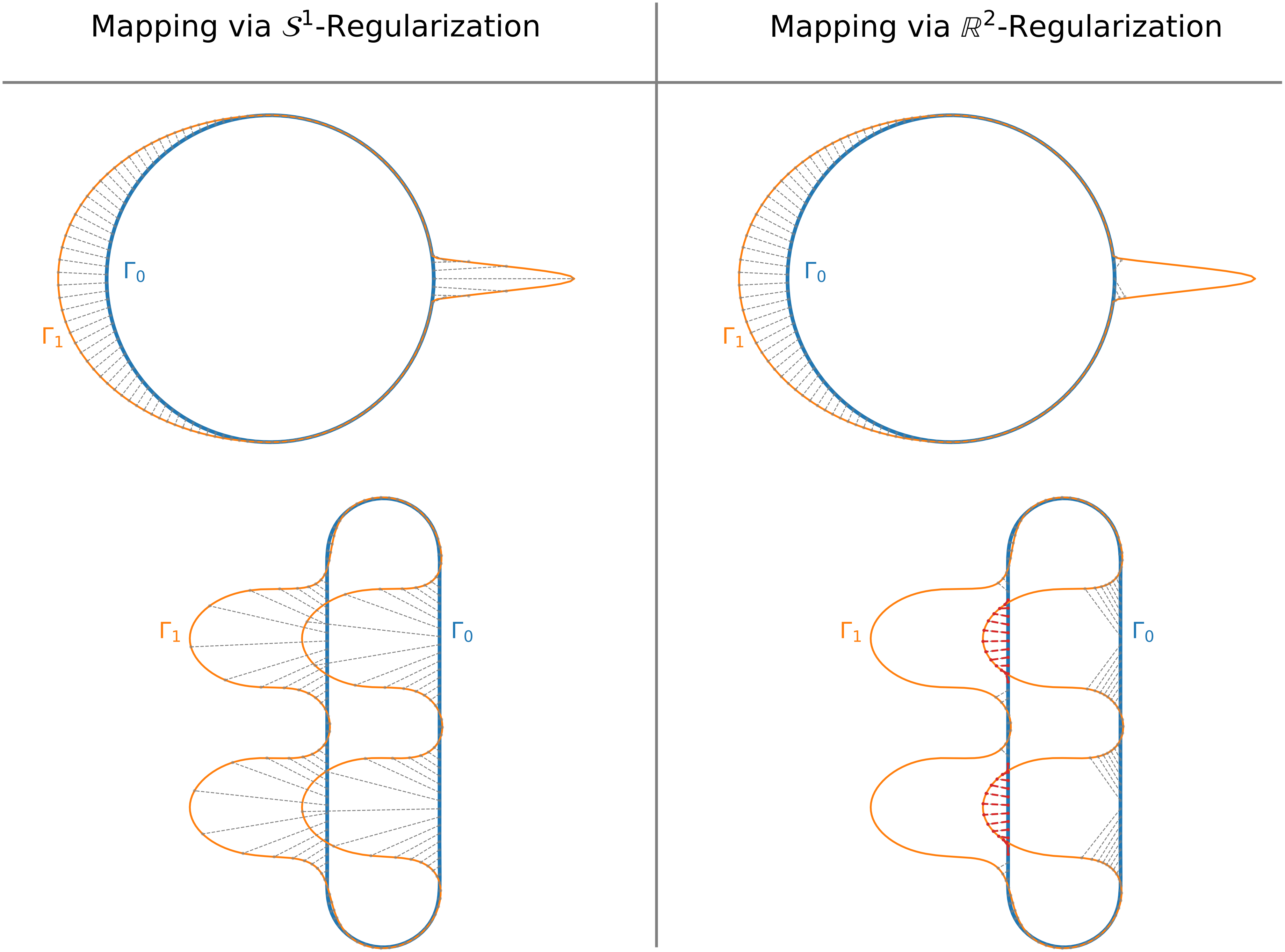

Fig S2. Comparison of virtual marker mappings obtained with different regularization schemes. On the left hand side, $\mathcal{S}^1$-regularization was used in which neighboring virtual markers are regularized w.r.t their normalized arc length coordinates. On the right hand side, the $\mathbb{R}^2$-distances of neighboring virtual markers is used for regularization. While the $\mathbb{R}^2$-regularization fails during large shape deformations, producing wide gaps between virtual markers and topological mapping violations (red lines), satisfying contour mappings are provided by the $\mathcal{S}^1$-regularization as used by our method.
