# Supplementary material for: Analysis of protrusion dynamics in amoeboid cell motility by means of regularized contour flows
Source: PLoS Comput Biol. 2021 Aug 23;17(8):e1009268. doi: 10.1371/journal.pcbi.1009268 (PMC8412247; doi:10.1371/journal.pcbi.1009268)
Supplement: S3 Fig — The underlying cell track was recorded with an imaging rate of δt ≈ 3.13s. (PDF) [file pcbi.1009268.s004.pdf]

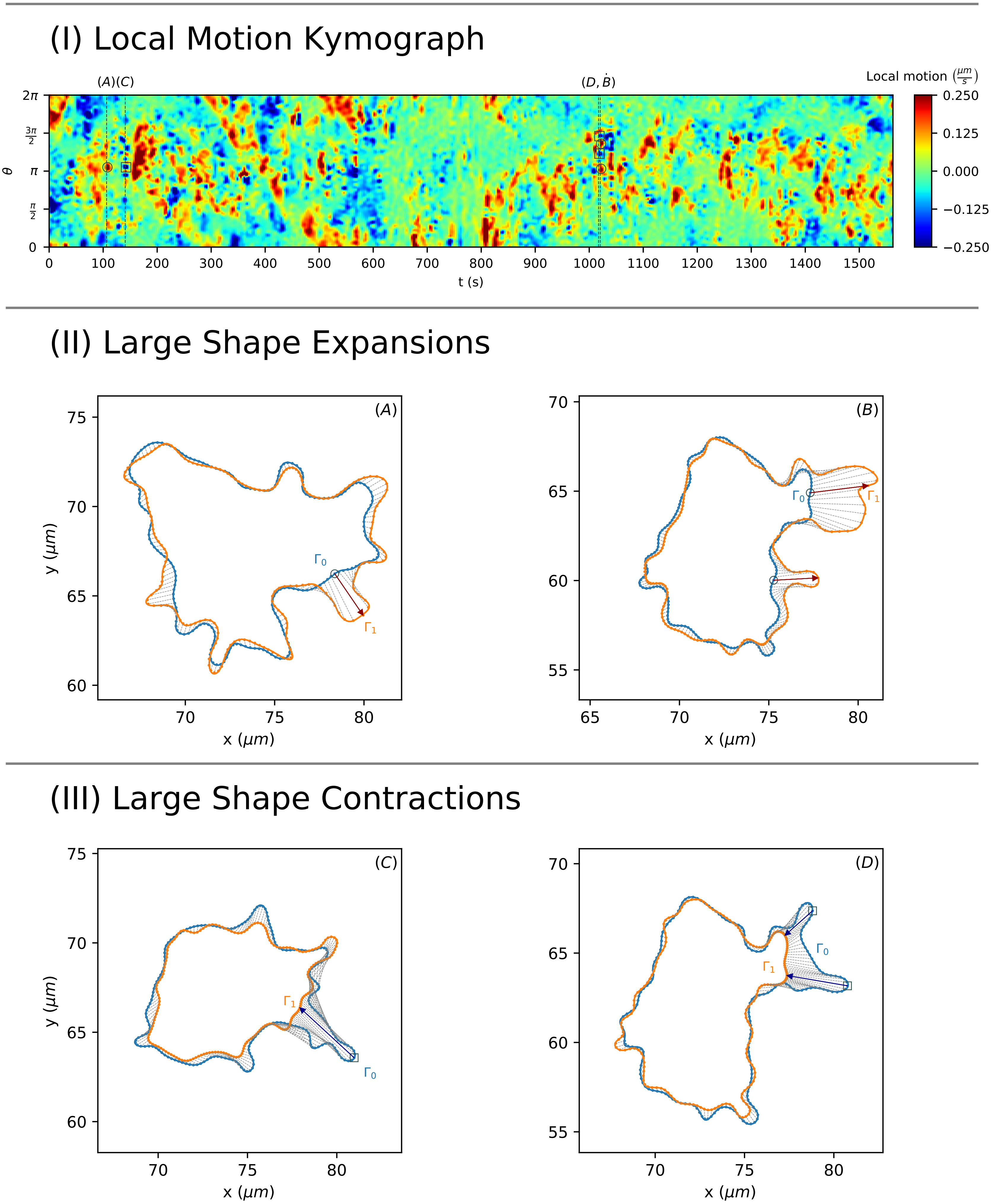

Fig S3. Cell track with large shape deformations. As in previous kymographs, a strongly regularized (global) flow is used to define the underlying coordinate system. Then, based on a weakly regularized (local) flow ($\lambda = 0.01$), the local motion is computed (first row). Compared to previous cell tracks, the imaging frequency of this cell track is roughly three times lower ($\delta t \approx 3.13s$), which results in larger shape deformations. Examples showing the local flow between two consecutive frames are displayed for expansions (second row) and contractions (third row). Expansion and contraction events, indicated by red and blue arrows, respectively, are also displayed in the local motion kymograph (gray circles/squares, respectively).
