# Supplementary material for: Analysis of protrusion dynamics in amoeboid cell motility by means of regularized contour flows
Source: PLoS Comput Biol. 2021 Aug 23;17(8):e1009268. doi: 10.1371/journal.pcbi.1009268 (PMC8412247; doi:10.1371/journal.pcbi.1009268)
Supplement: S4 Fig — In the figure at the top of each page, the period of time of several cell tracks was shortened for illustrative purposes due to overlapping contours. (PDF) [file pcbi.1009268.s005.pdf]

Pulsating Circle

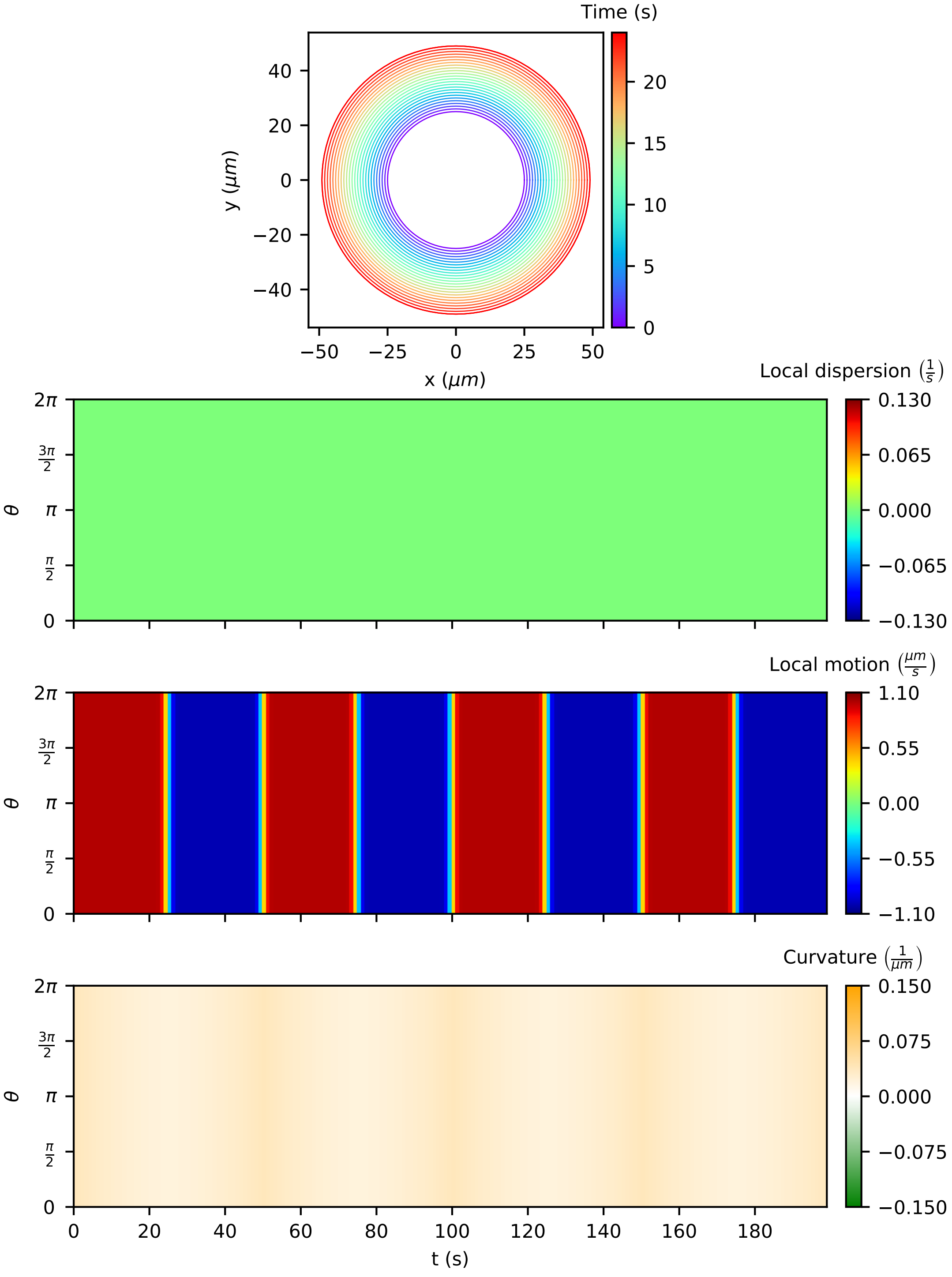

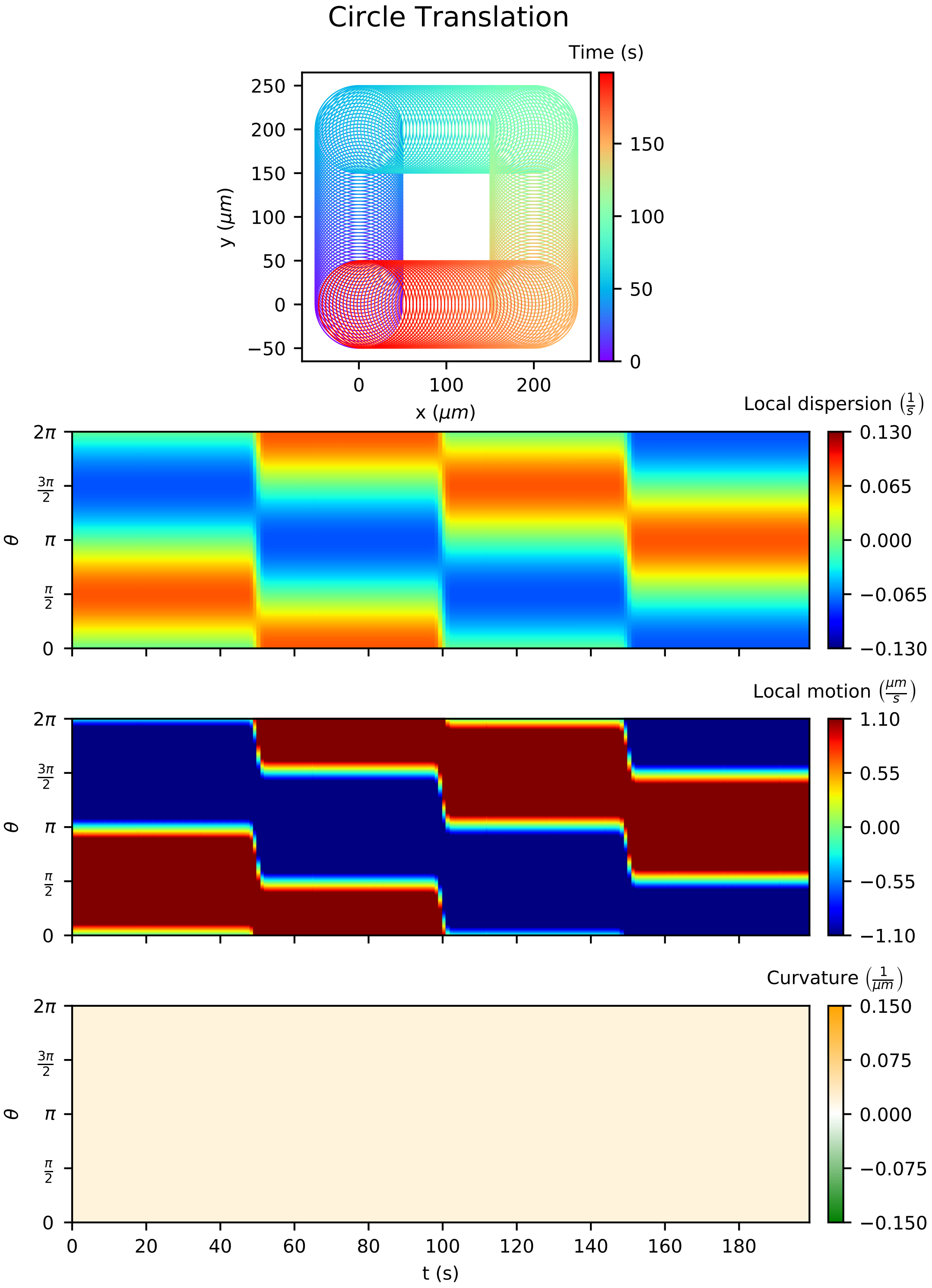

Circle Translation
Time (s)
y (μm)
x (μm)
Local dispersion $\left(\frac{1}{s}\right)$
Local motion $\left(\frac{\mu m}{s}\right)$
Curvature $\left(\frac{1}{\mu m}\right)$
θ
t (s)

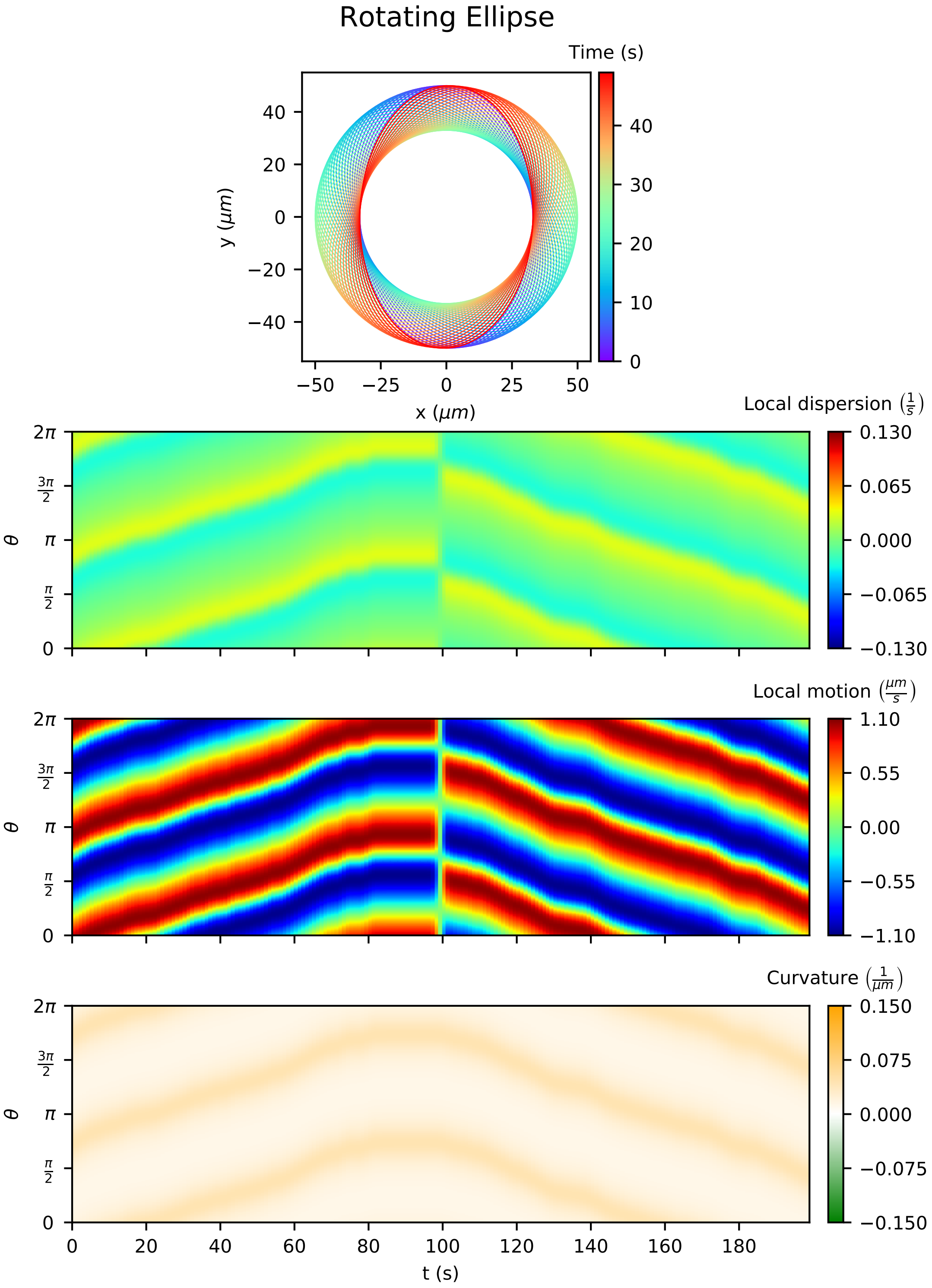

Rotating Ellipse
Time (s)
y (μm)
x (μm)
Local dispersion $\left(\frac{1}{s}\right)$
Local motion $\left(\frac{\mu m}{s}\right)$
Curvature $\left(\frac{1}{\mu m}\right)$
θ
t (s)

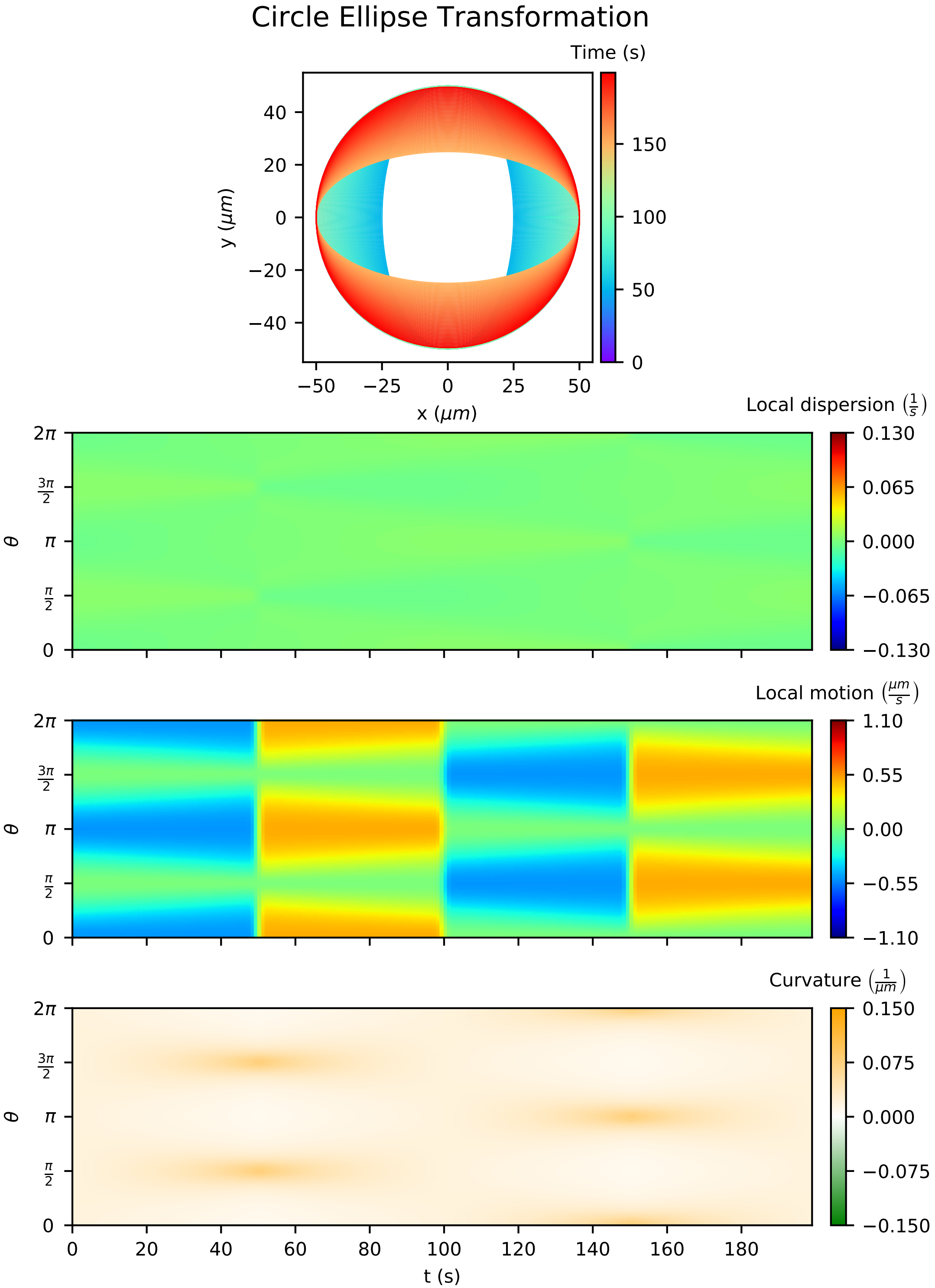

Circle Ellipse Transformation
Time (s)
y (μm)
x (μm)
Local dispersion $\left(\frac{1}{s}\right)$
Local motion $\left(\frac{\mu m}{s}\right)$
Curvature $\left(\frac{1}{\mu m}\right)$
θ
t (s)

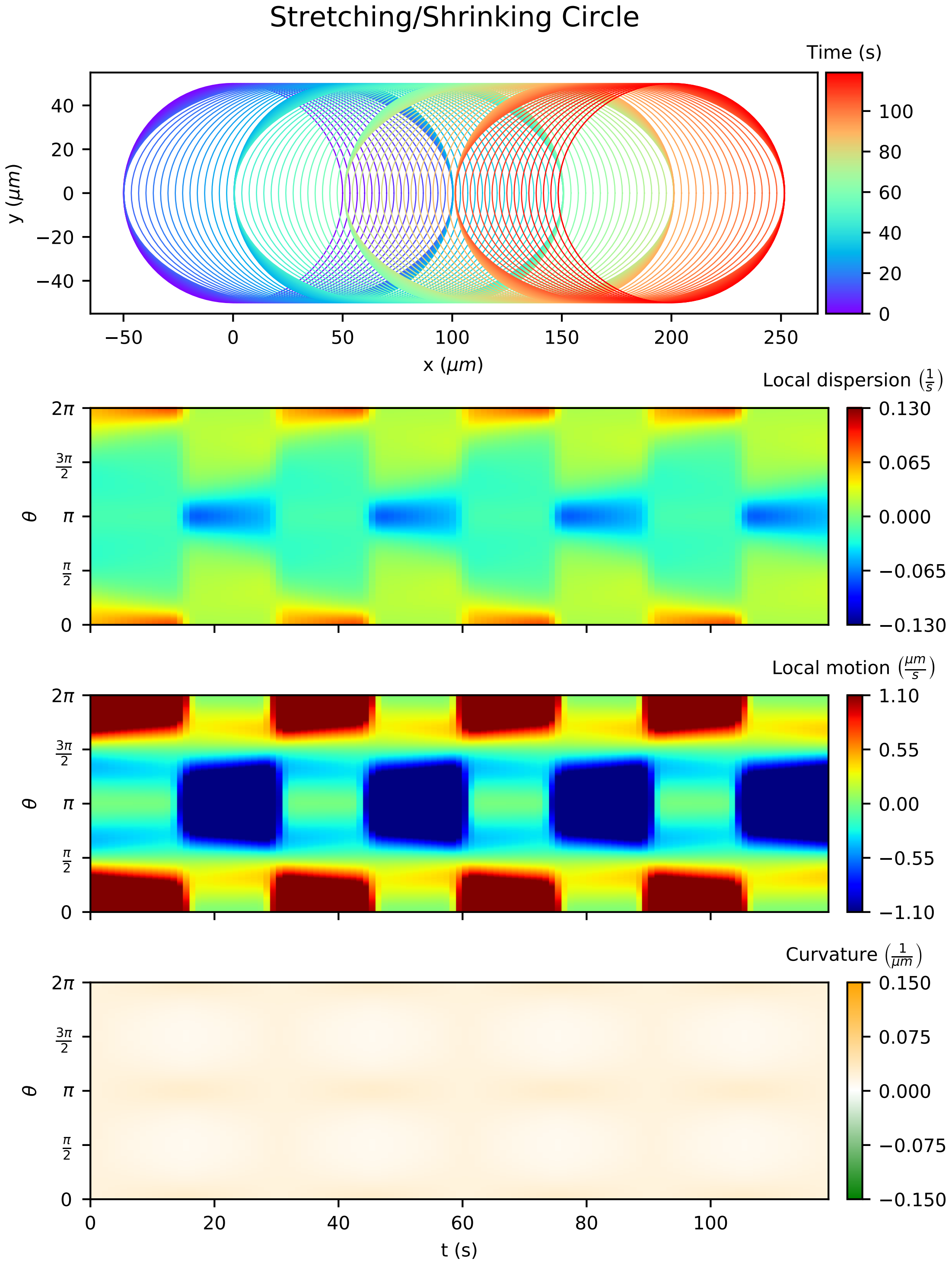

Stretching/Shrinking Circle
Time (s)
y (μm)
x (μm)
Local dispersion (1/s)
Local motion (μm/s)
Curvature (1/μm)
θ
t (s)

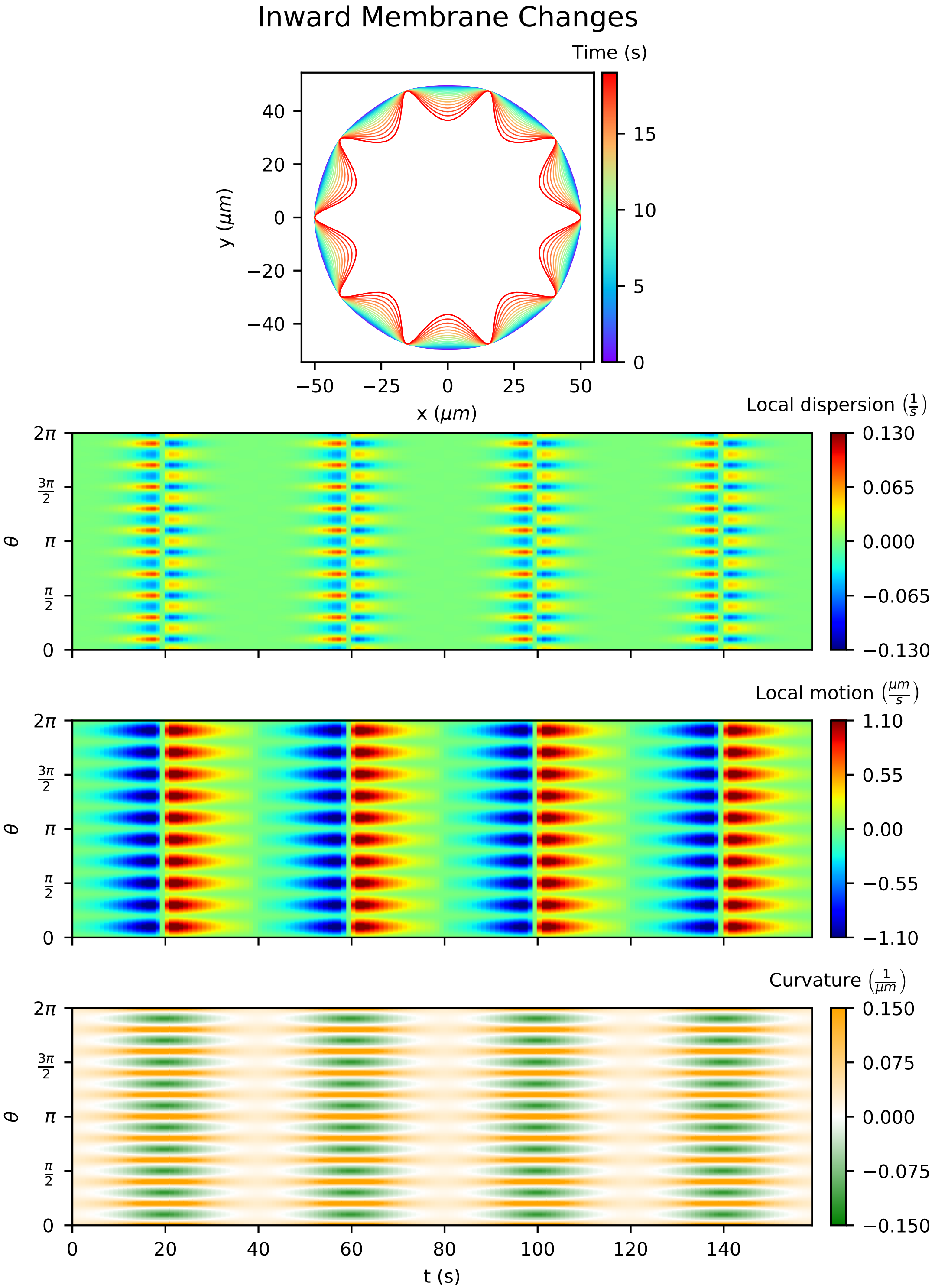

Inward Membrane Changes
Time (s)
y (μm)
x (μm)
Local dispersion $\left(\frac{1}{s}\right)$
Local motion $\left(\frac{\mu m}{s}\right)$
Curvature $\left(\frac{1}{\mu m}\right)$
θ
t (s)

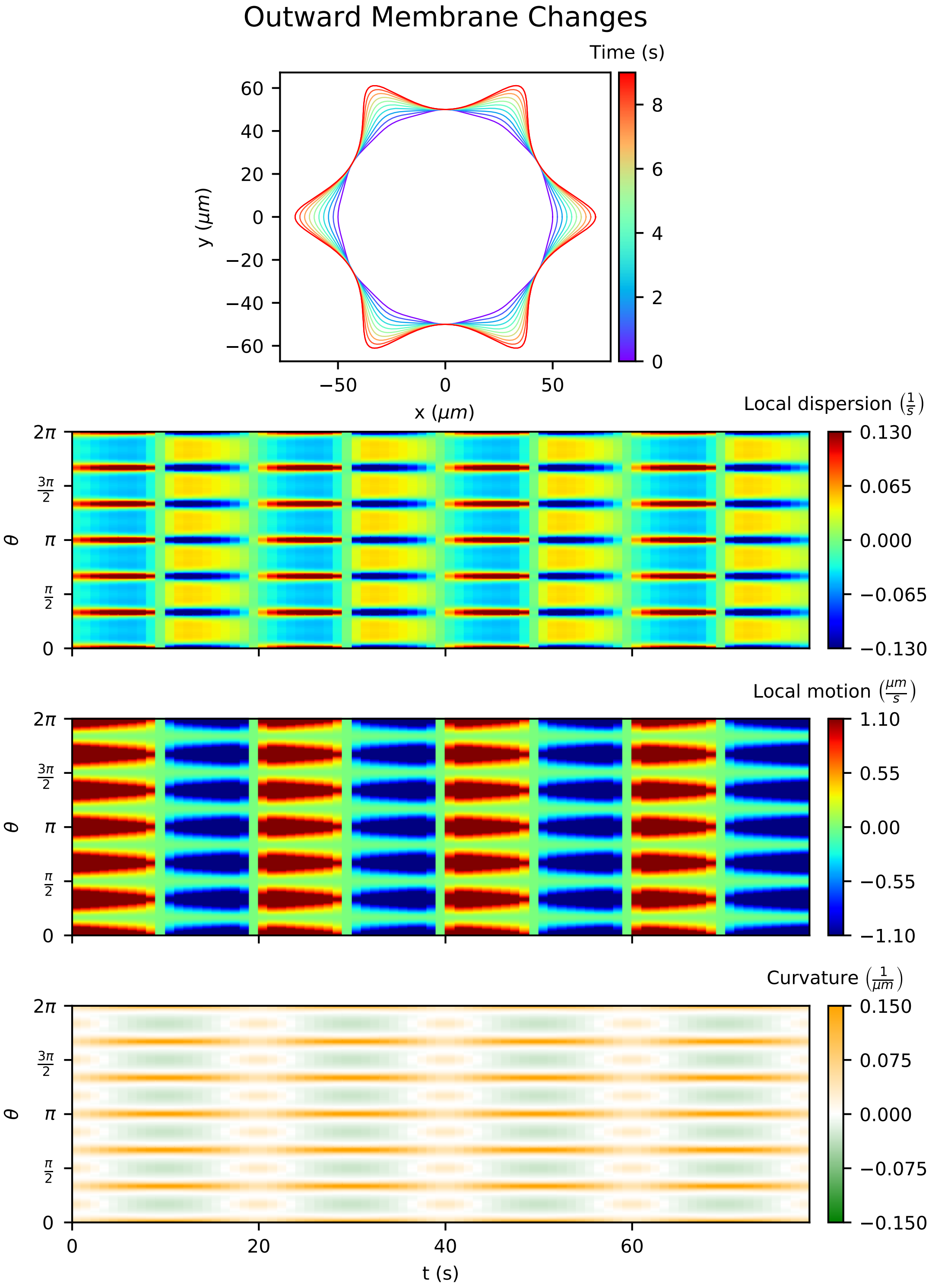

Outward Membrane Changes
Time (s)
y (μm)
x (μm)
Local dispersion $\left(\frac{1}{s}\right)$
Local motion $\left(\frac{\mu m}{s}\right)$
Curvature $\left(\frac{1}{\mu m}\right)$
θ
t (s)

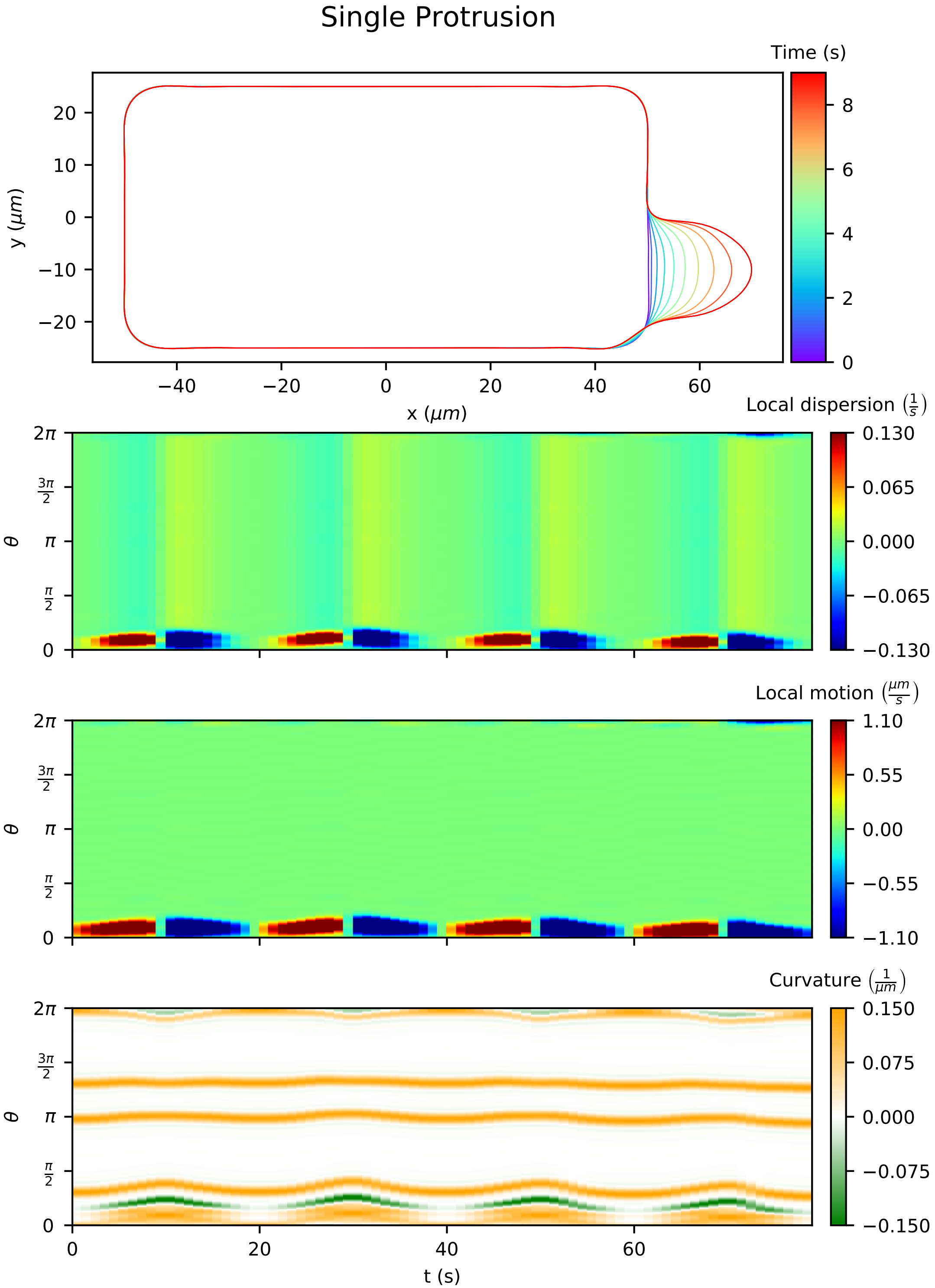

Single Protrusion
Time (s)
y (μm)
x (μm)
Local dispersion (1/s)
Local motion (μm/s)
Curvature (1/μm)
θ
t (s)

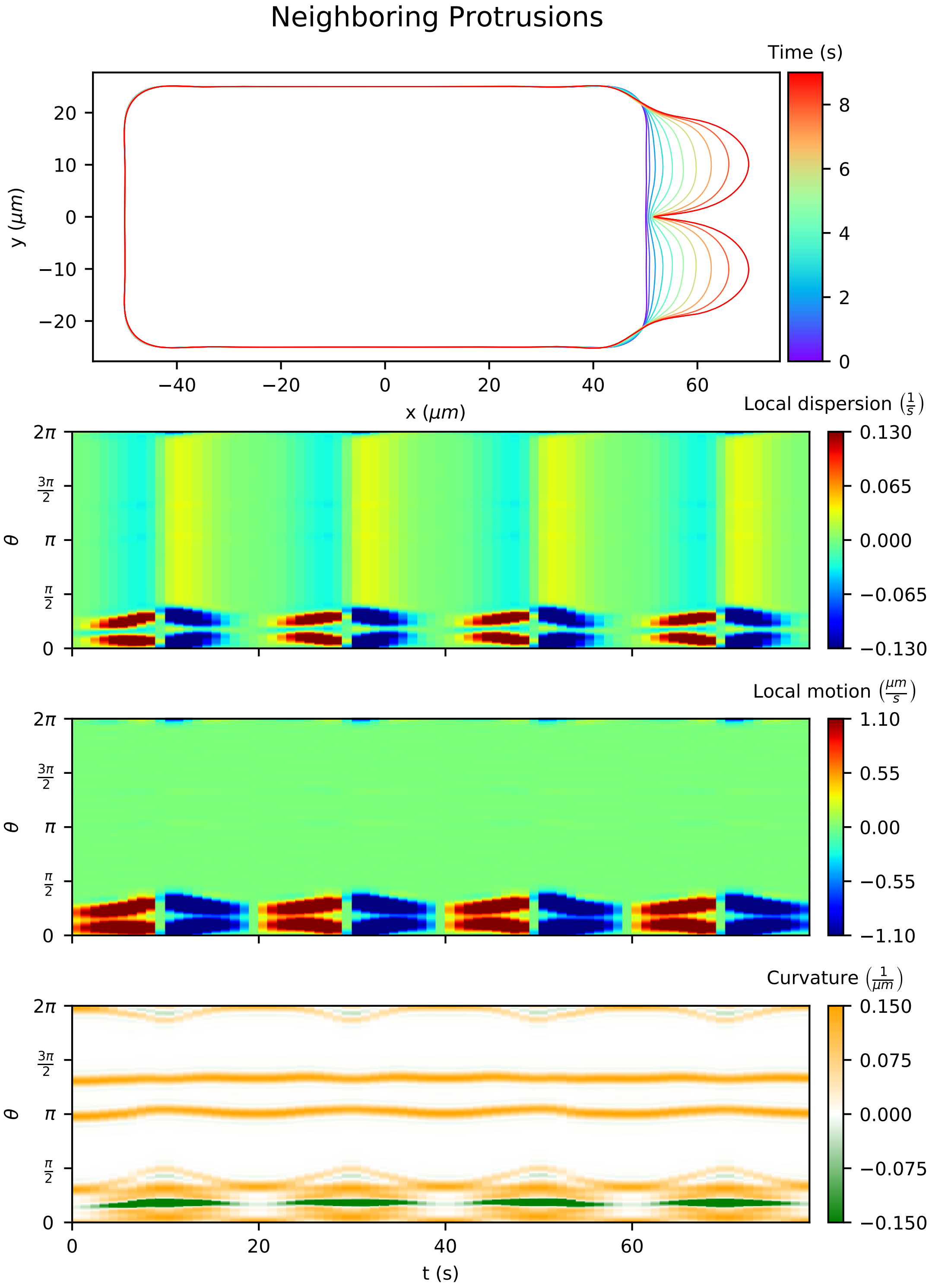

Neighboring Protrusions
Time (s)
y (μm)
x (μm)
Local dispersion (1/s)
Local motion (μm/s)
Curvature (1/μm)
θ
t (s)

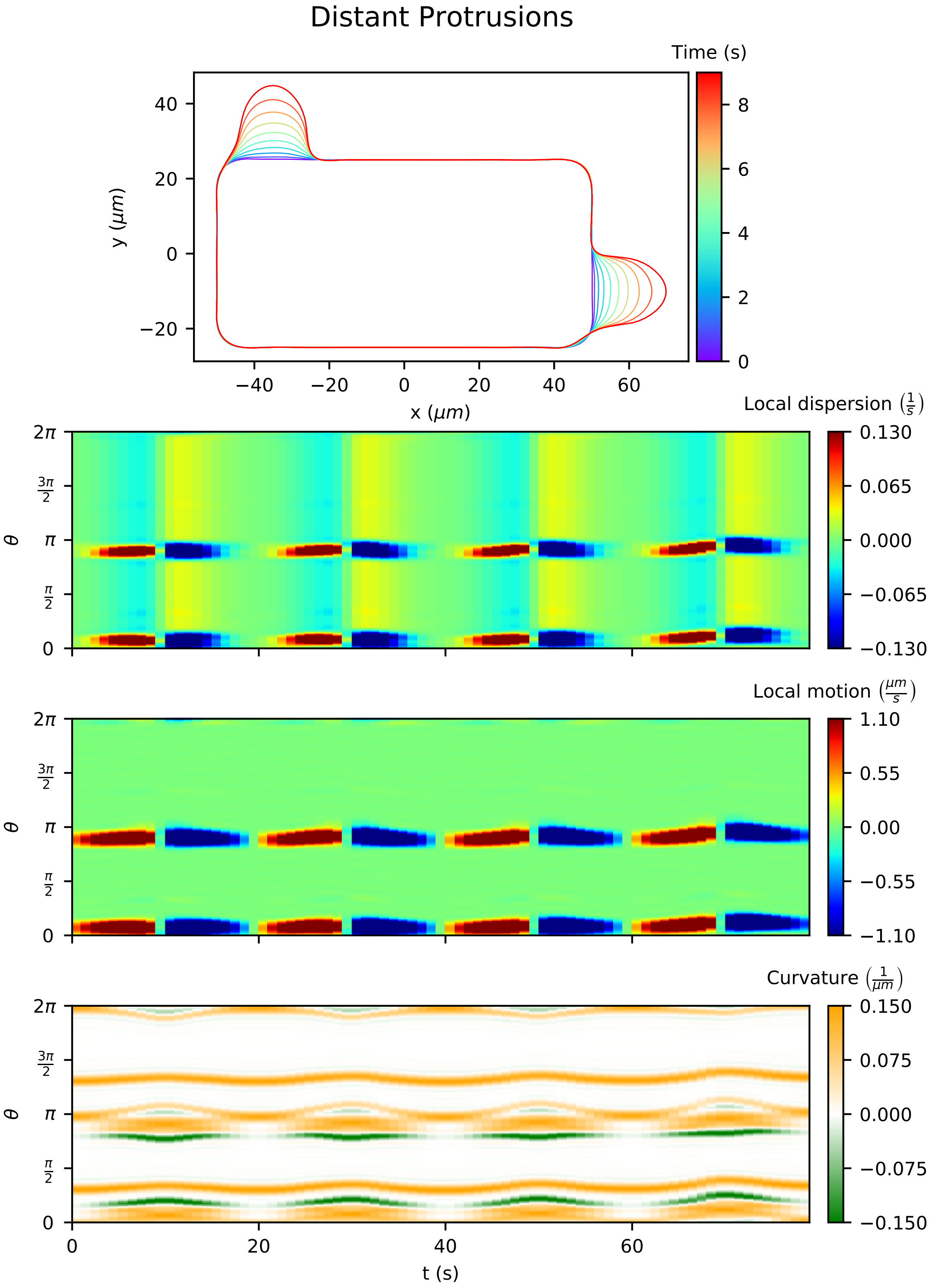

Distant Protrusions
Time (s)
y (μm)
x (μm)
Local dispersion $\left(\frac{1}{s}\right)$
Local motion $\left(\frac{\mu m}{s}\right)$
Curvature $\left(\frac{1}{\mu m}\right)$
θ
t (s)

Different Sized Protrusions

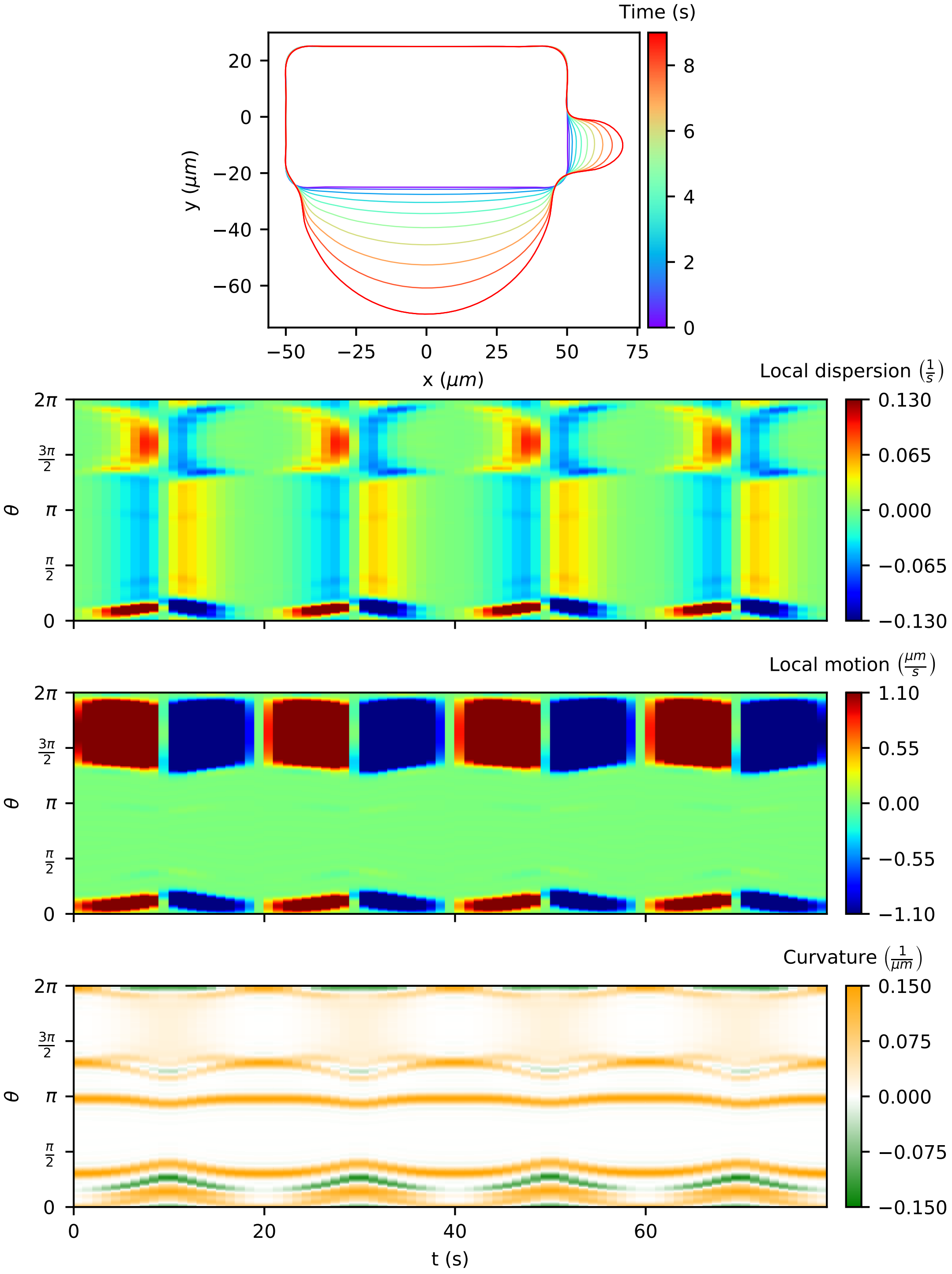

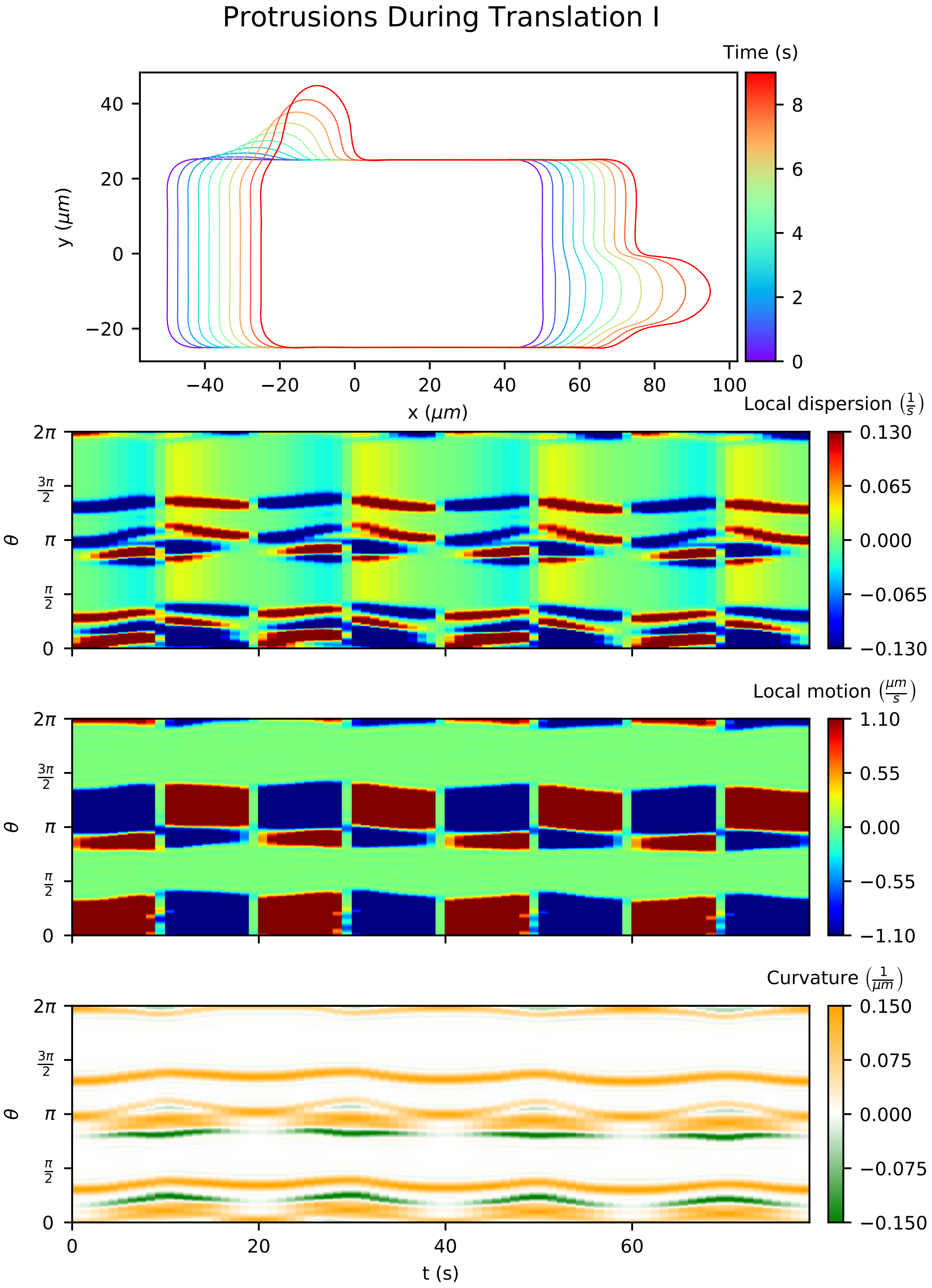

Protrusions During Translation I
Time (s)
y (μm)
x (μm)
Local dispersion (1/s)
Local motion (μm/s)
Curvature (1/μm)
θ
t (s)

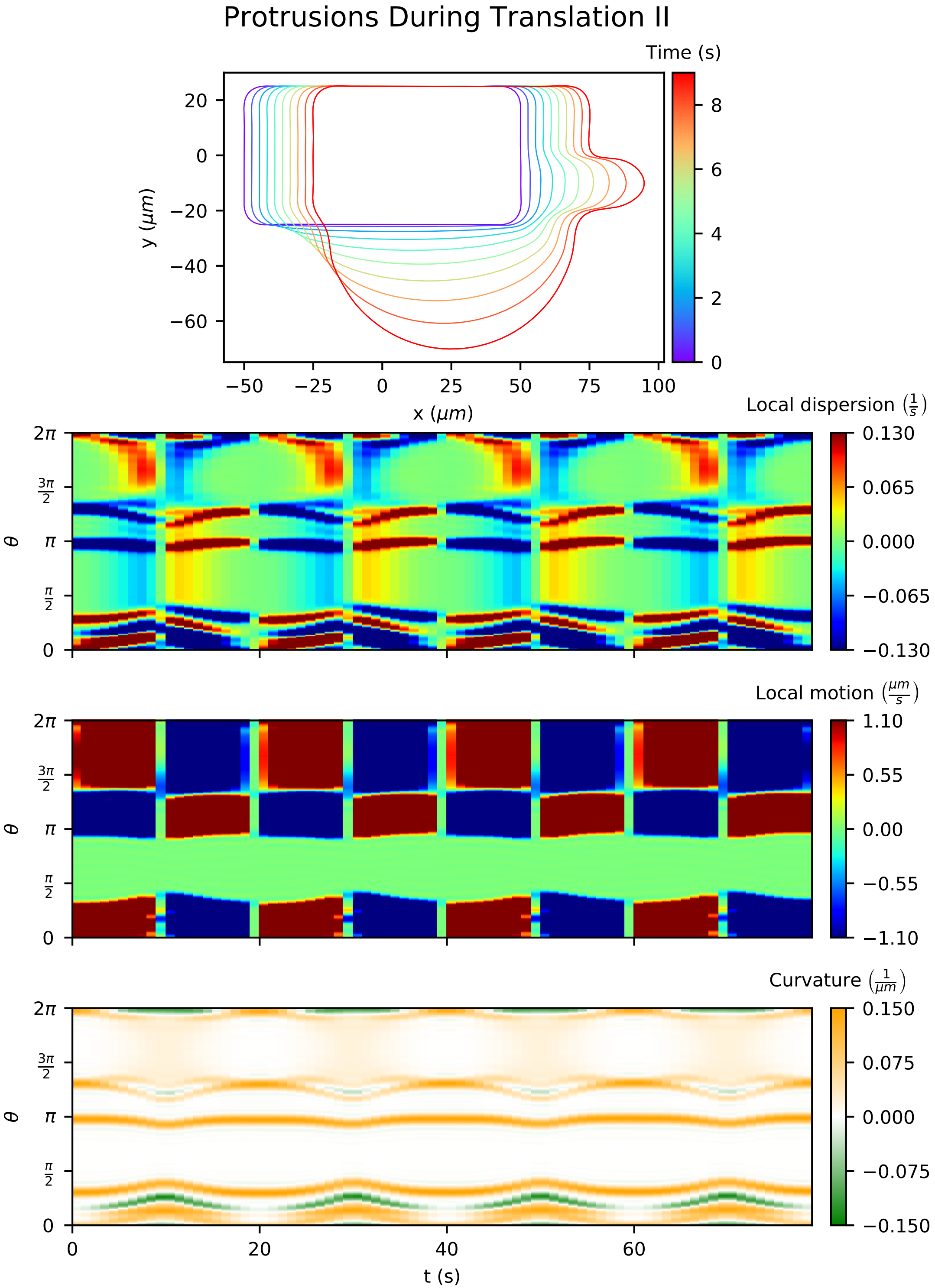
Protrusions During Translation II
Time (s)
y (μm)
x (μm)
Local dispersion $(\frac{1}{s})$
Local motion $(\frac{\mu m}{s})$
Curvature $(\frac{1}{\mu m})$
θ
t (s)
