# Supplementary material for: Analysis of protrusion dynamics in amoeboid cell motility by means of regularized contour flows
Source: PLoS Comput Biol. 2021 Aug 23;17(8):e1009268. doi: 10.1371/journal.pcbi.1009268 (PMC8412247; doi:10.1371/journal.pcbi.1009268)
Supplement: S6 Fig — (PDF) [file pcbi.1009268.s007.pdf]

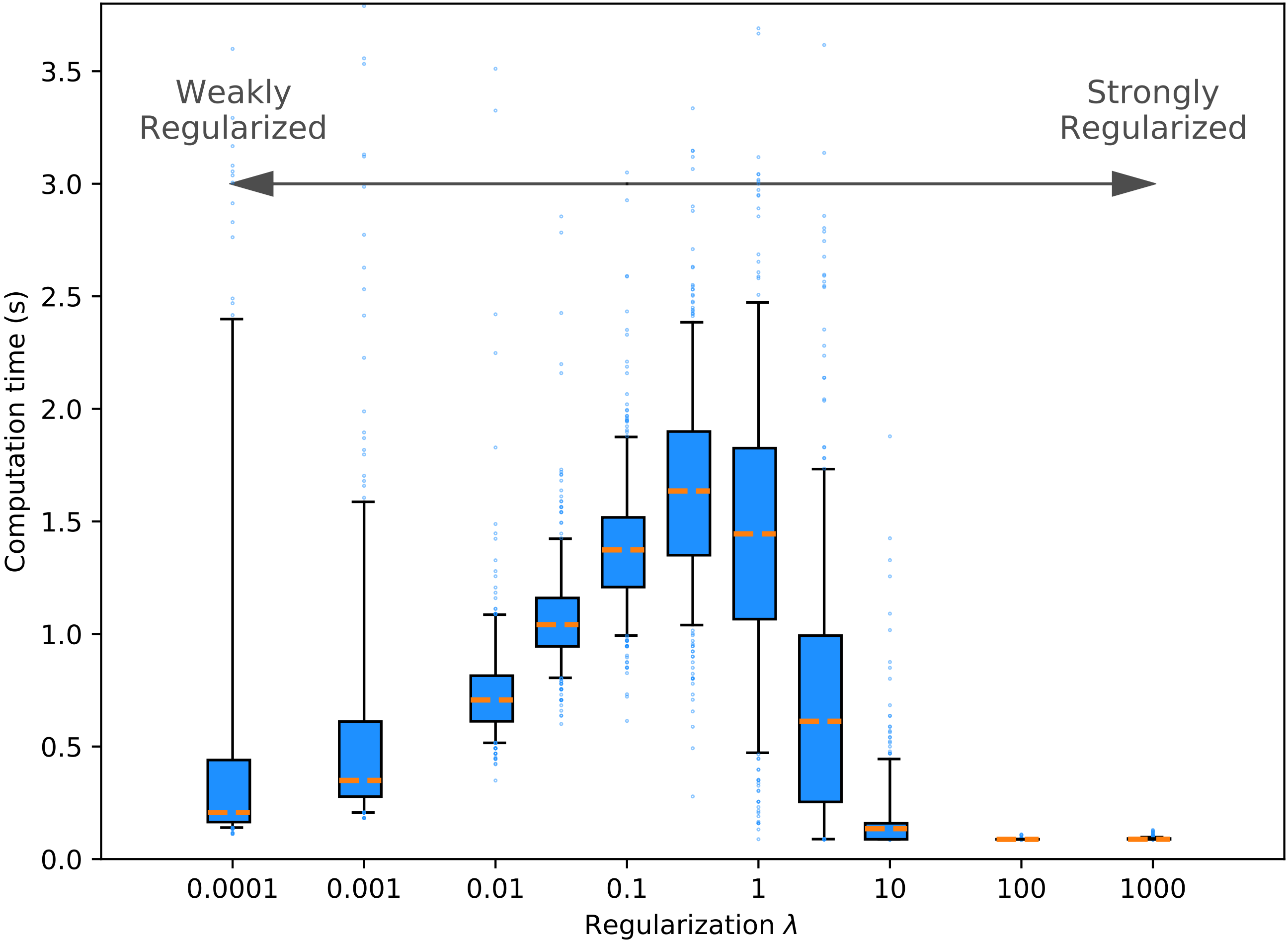

Fig S6. Computation times of the algorithm "Regflow" from Fig 2 for different values of the regularization parameter λ for the mapping between two consecutive contours. The statistic is taken over 500 pairs of consecutive contours of the cell track displayed in Fig 4, each based on 400 virtual markers. For each λ, the median (orange lines), the upper and lower quartile (blue boxes), and the 5th and 95th percentile (black lines) are shown.
