# Supplementary material for: Analysis of protrusion dynamics in amoeboid cell motility by means of regularized contour flows
Source: PLoS Comput Biol. 2021 Aug 23;17(8):e1009268. doi: 10.1371/journal.pcbi.1009268 (PMC8412247; doi:10.1371/journal.pcbi.1009268)
Supplement: S7 Fig — (PDF) [file pcbi.1009268.s008.pdf]

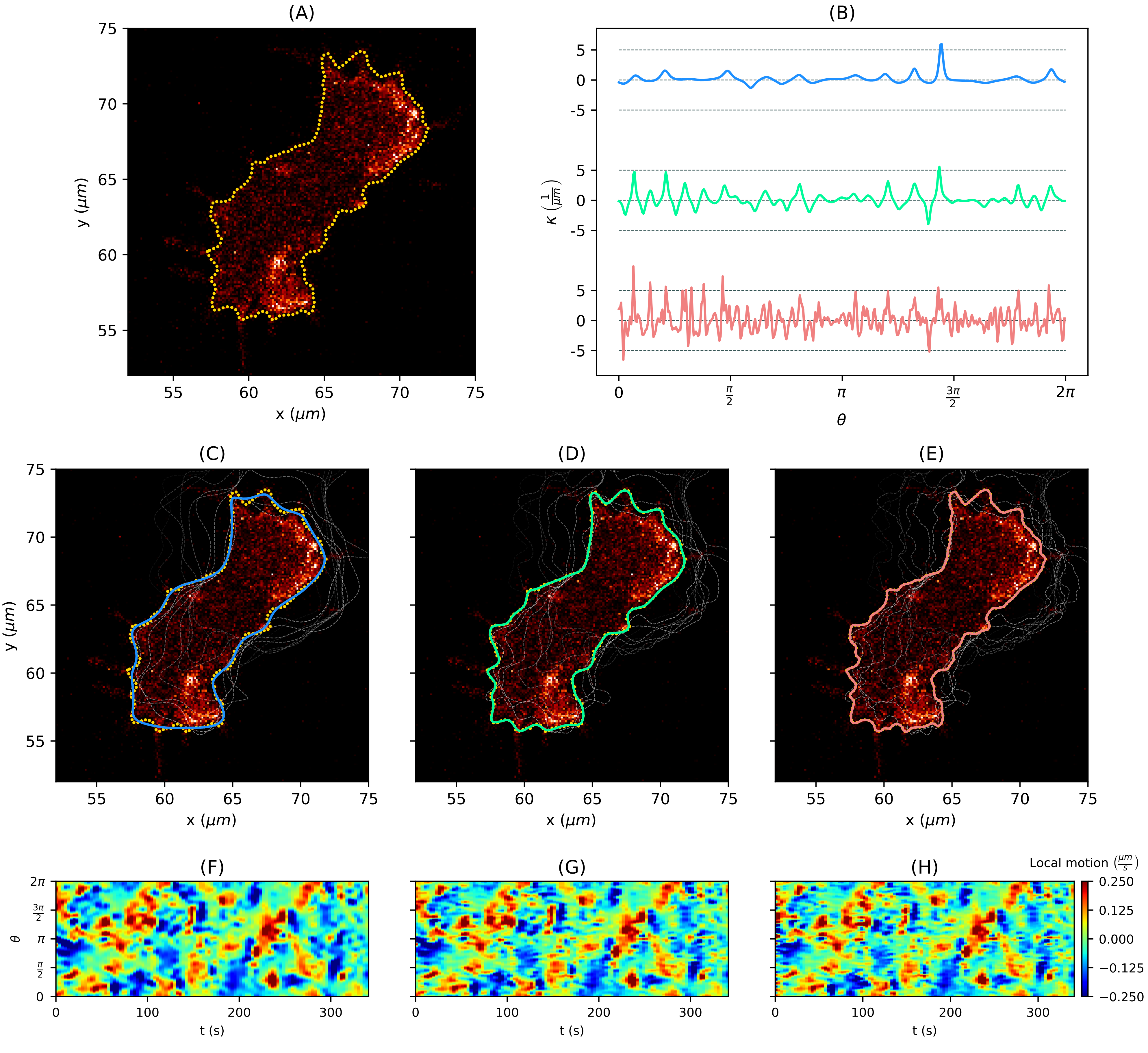

Fig S7. Discrete set of points (yellow) segmented from a noisy fluorescence image **(A)**. By using Gaussian process regression with varying parameters of the underlying Poisson kernel, different estimations of the membrane can be obtained. The curvature of these estimated contours are shown in **(B)**. The parameters were chosen as follows: $r = 0.4$ (blue), $r = 0.65$ (green), and $r = 0.9$ (red). In **(C, r = 0.4)**, the resulting contour is highly underfitted which leads to a strongly regularized curvature. In **(E, r = 0.9)**, an overfitting effect can be observed, resulting in high fluctuations of the curvature. In **(D, r = 0.65)** a more plausible parameter was chosen leading to an accurate approximation while preserving the main characteristics of the curvature. The corresponding local motion kymographs are shown in panel **(F − H)**. For **(F, r = 0.4)**, some details are not resolved. In contrast, only few differences can be observed between **(G, r = 0.65)** and **(H, r = 0.9)**, which shows that the estimate for $r = 0.65$ is already an accurate approximation of the contour.
