# Supplementary material for: Analysis of protrusion dynamics in amoeboid cell motility by means of regularized contour flows
Source: PLoS Comput Biol. 2021 Aug 23;17(8):e1009268. doi: 10.1371/journal.pcbi.1009268 (PMC8412247; doi:10.1371/journal.pcbi.1009268)
Supplement: S8 Fig — (PDF) [file pcbi.1009268.s009.pdf]

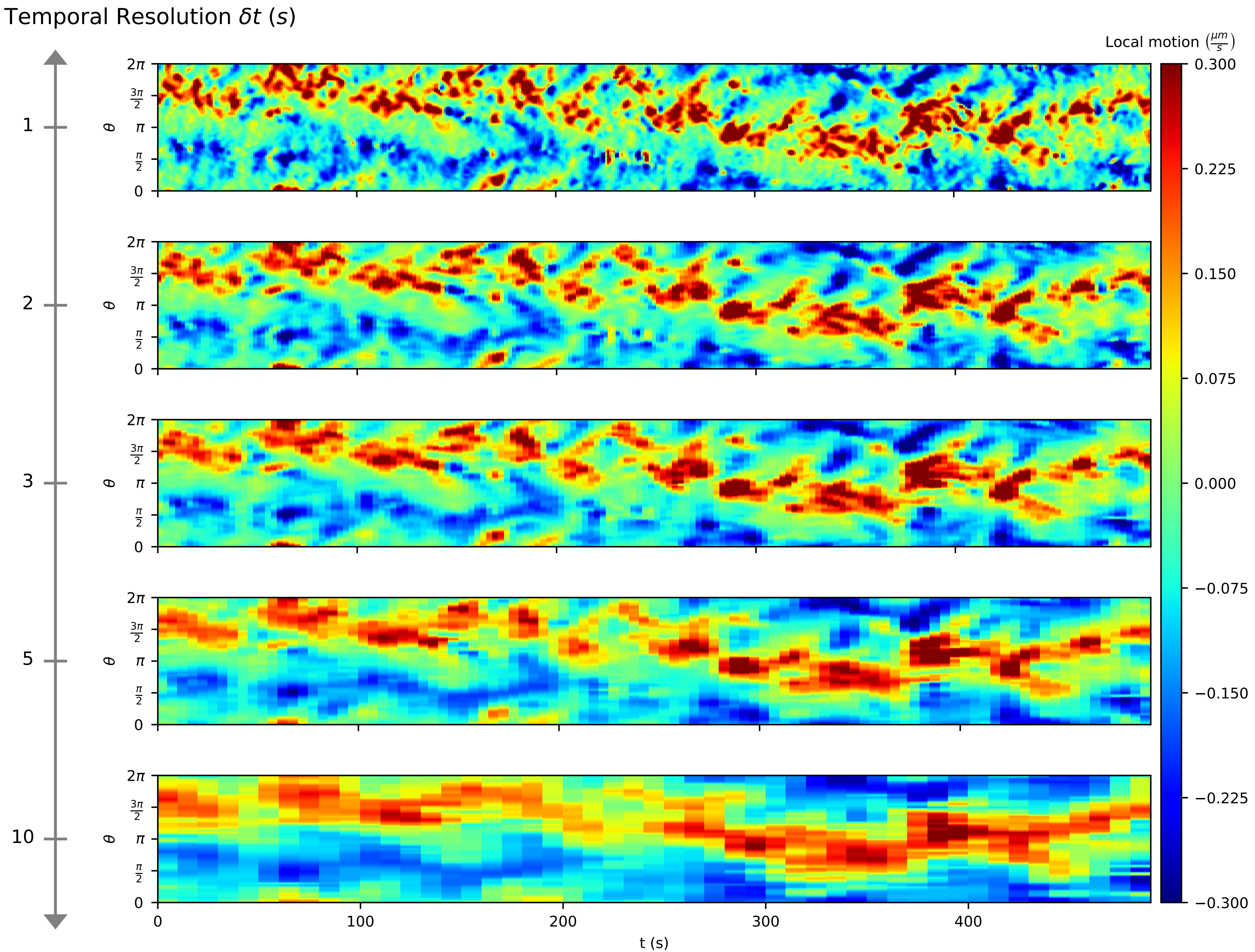

Fig S8. Comparison of local motion kymographs computed for different imaging frequencies. At the top, the kymograph is based on one image/contour per second. In the kymographs below, the underlying contour flows were computed for every 2nd, 3rd, 5th, and 10th image. For decreasing imaging frequencies, the identification of local membrane changes becomes more difficult. However, the algorithm is stable even for a lower temporal resolution ($\delta t > 3s$) producing contour flows without mapping violations while capturing global features of the contour dynamics.
