# Supplementary material for: Analysis of protrusion dynamics in amoeboid cell motility by means of regularized contour flows
Source: PLoS Comput Biol. 2021 Aug 23;17(8):e1009268. doi: 10.1371/journal.pcbi.1009268 (PMC8412247; doi:10.1371/journal.pcbi.1009268)
Supplement: S9 Fig — (PDF) [file pcbi.1009268.s010.pdf]

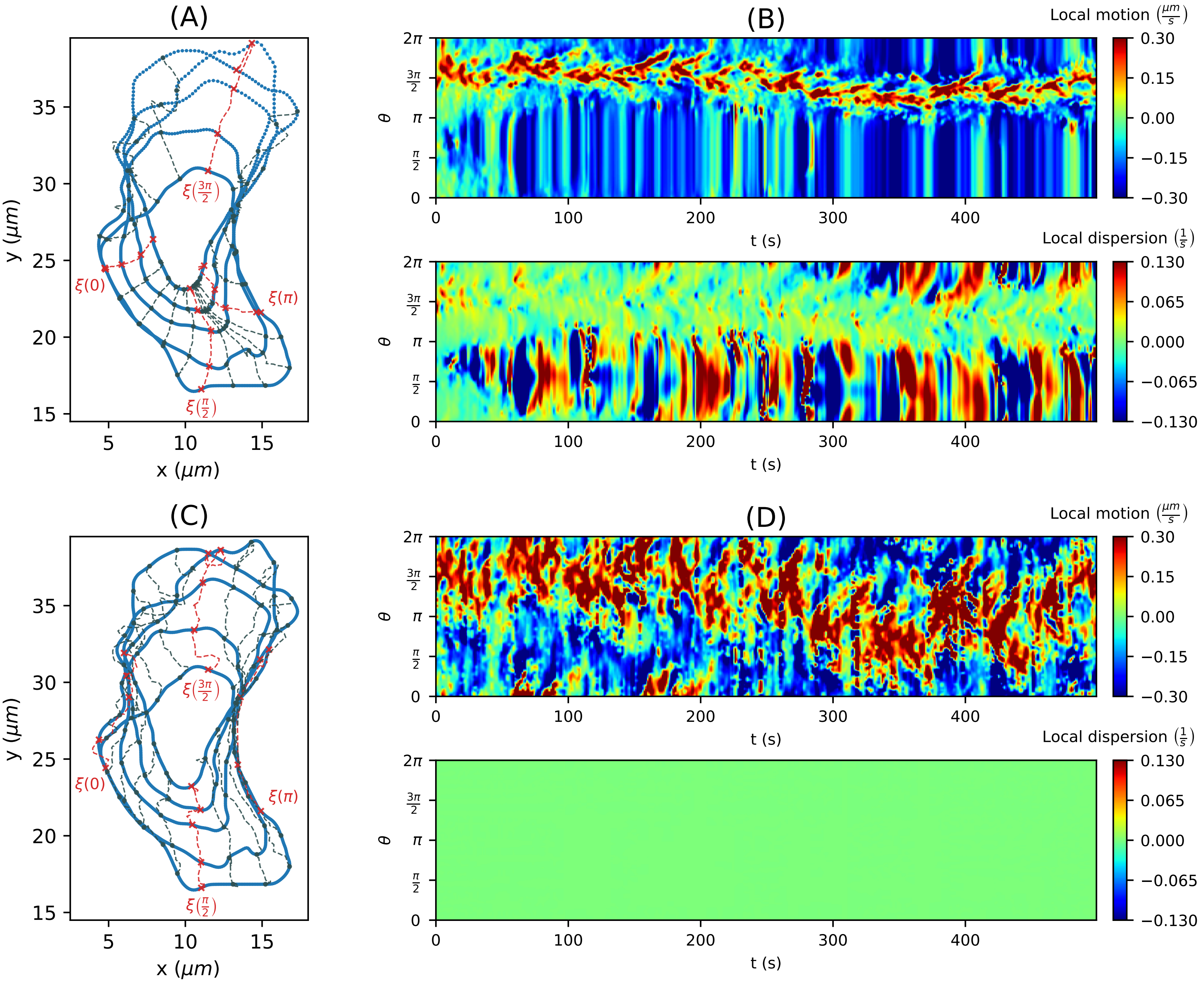

Fig S9. Weakly regularized global flow (**A**, $\lambda = 1$) with corresponding kymographs: Local motion (top) and local dispersion (bottom). Clustering and thinning effects appear at the back side and front side of the cell, respectively, which results in an overproportional display of a short retractive contour segment. For the case of the strongly regularized flow (**C**, $\lambda = 1000$) we observe a more uniform distribution of virtual markers along the cell. Therefore, the corresponding local dispersion kymograph becomes less informative (**D**, bottom).
