# Supplementary material for: Analysis of protrusion dynamics in amoeboid cell motility by means of regularized contour flows
Source: PLoS Comput Biol. 2021 Aug 23;17(8):e1009268. doi: 10.1371/journal.pcbi.1009268 (PMC8412247; doi:10.1371/journal.pcbi.1009268)
Supplement: S10 Fig — (PDF) [file pcbi.1009268.s011.pdf]

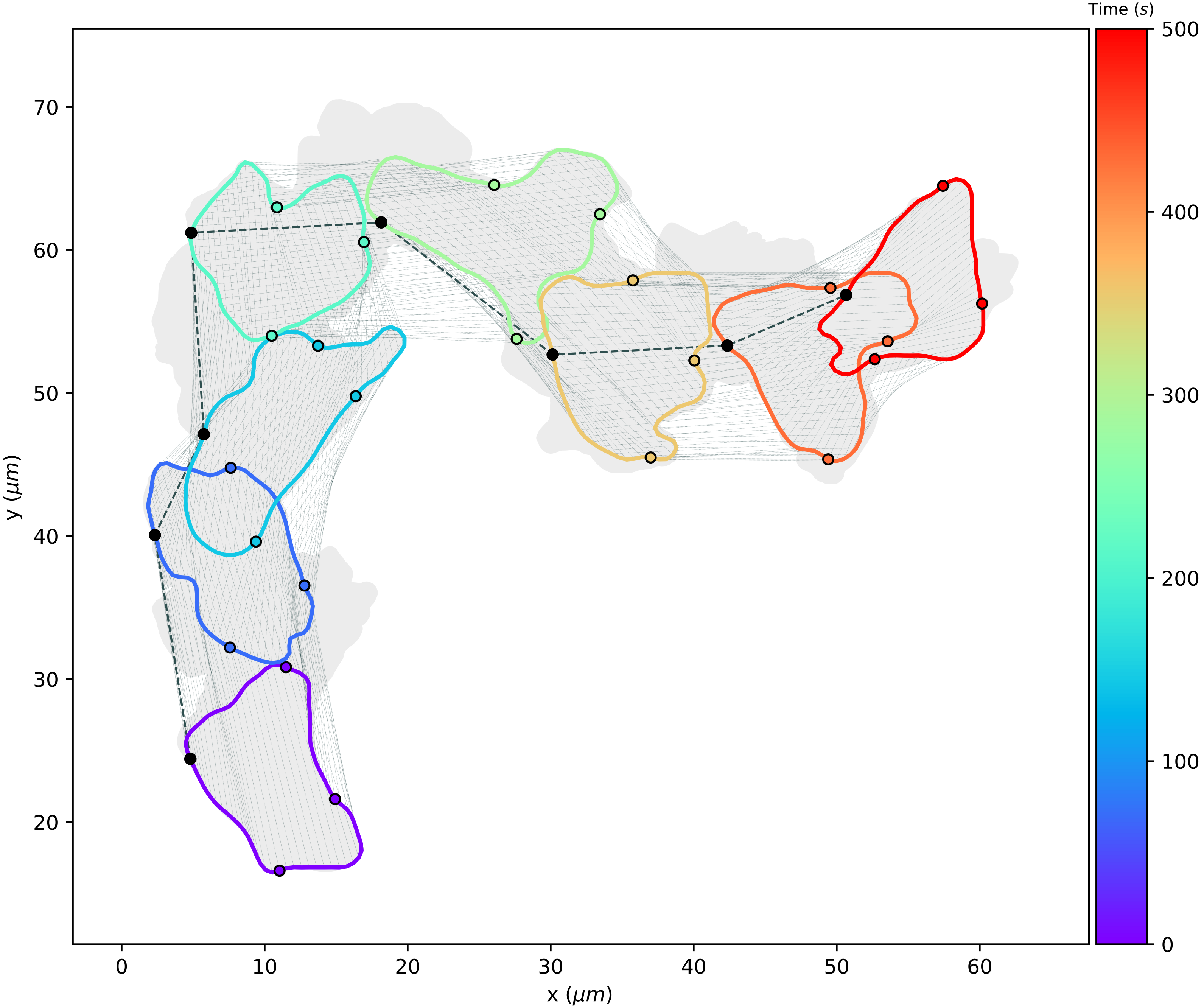

Fig S10. Extreme test scenario to further challenge our algorithm. In this test case, only 8 out of 500 contours are taken into account. The underlying coordinate system is defined by a strongly regularized flow ($\lambda = 1000$) and is depicted as gray lines.For illustration, we have marked four points on the contour with normalized arc length 0, $\frac{\pi}{2}$, π, $\frac{3\pi}{2}$ and highlighted the mapping of one of them. Additionally, the initial cell track is highlighted as gray area. Noticeable, no mapping violations were produced. Moreover, the strongly regularized flow prevents clustering and thinning effects of virtual markers.
