# Supplementary figures and images for: Analysis of protrusion dynamics in amoeboid cell motility by means of regularized contour flows
Source: PLoS Comput Biol. 2021 Aug 23;17(8):e1009268. doi: 10.1371/journal.pcbi.1009268 (PMC8412247; doi:10.1371/journal.pcbi.1009268)

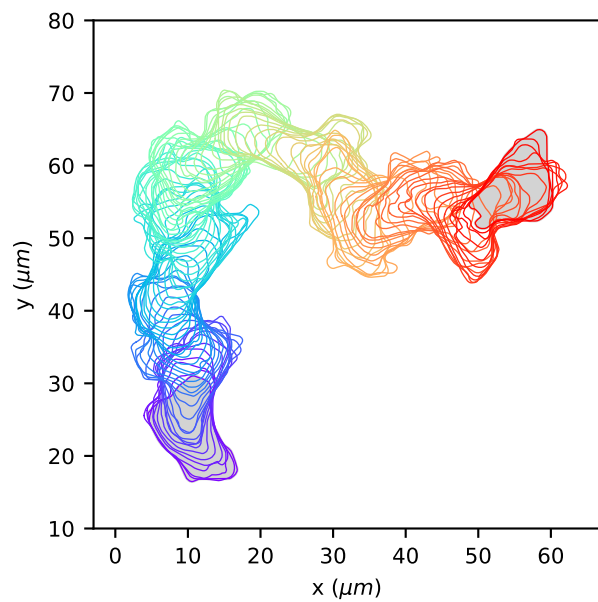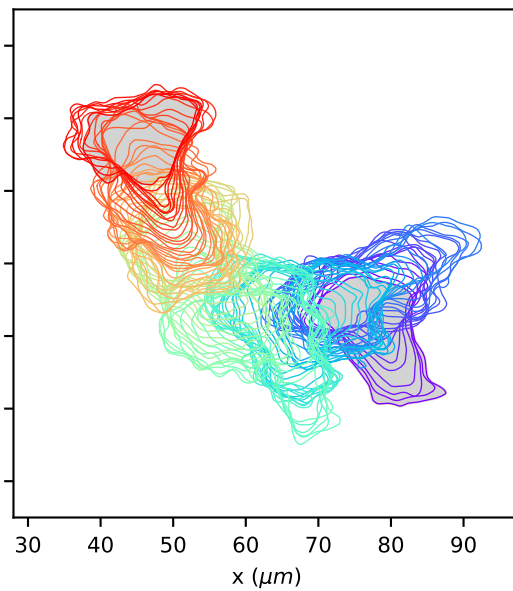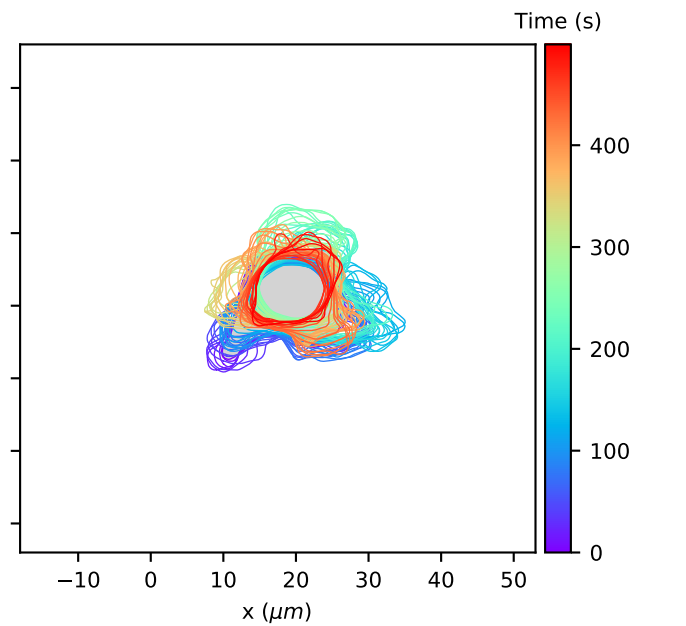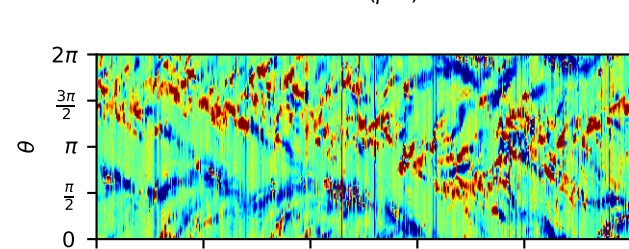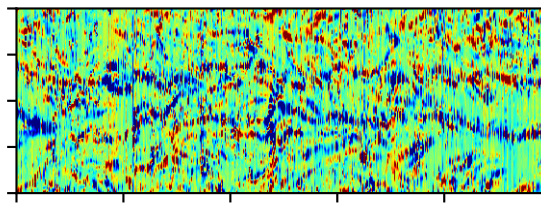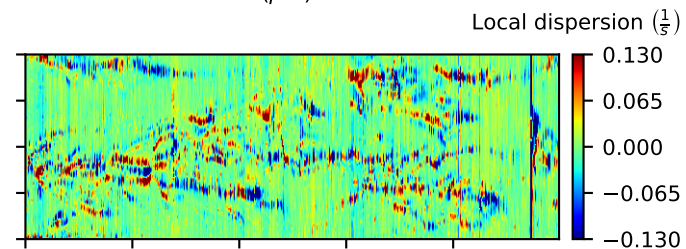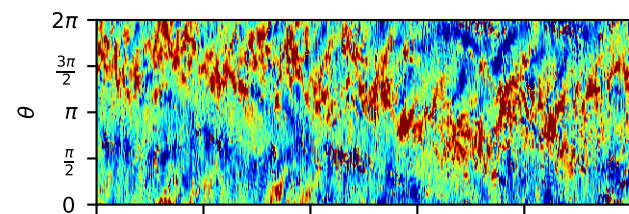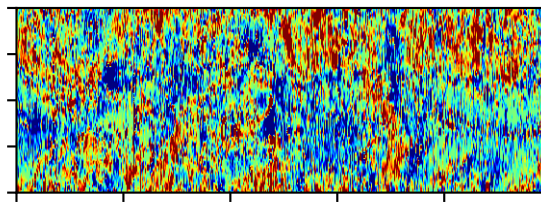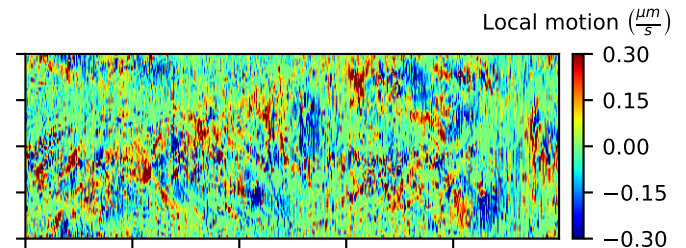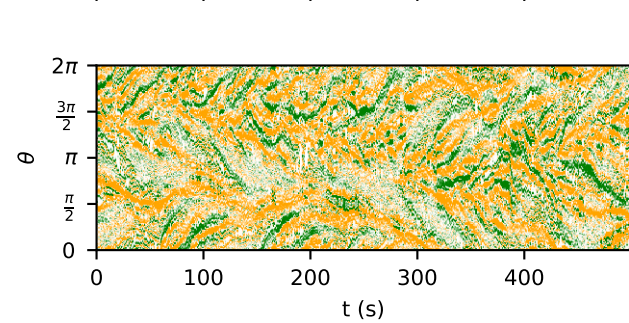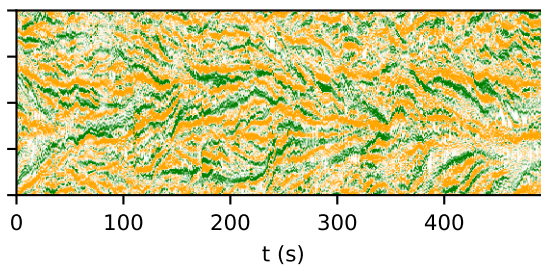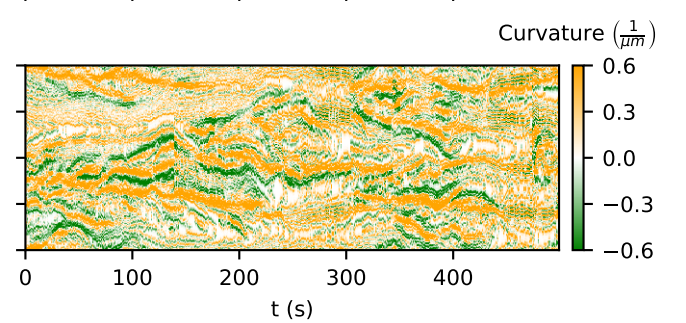

Supplement: S11 Fig — (PDF) [file pcbi.1009268.s012.pdf]

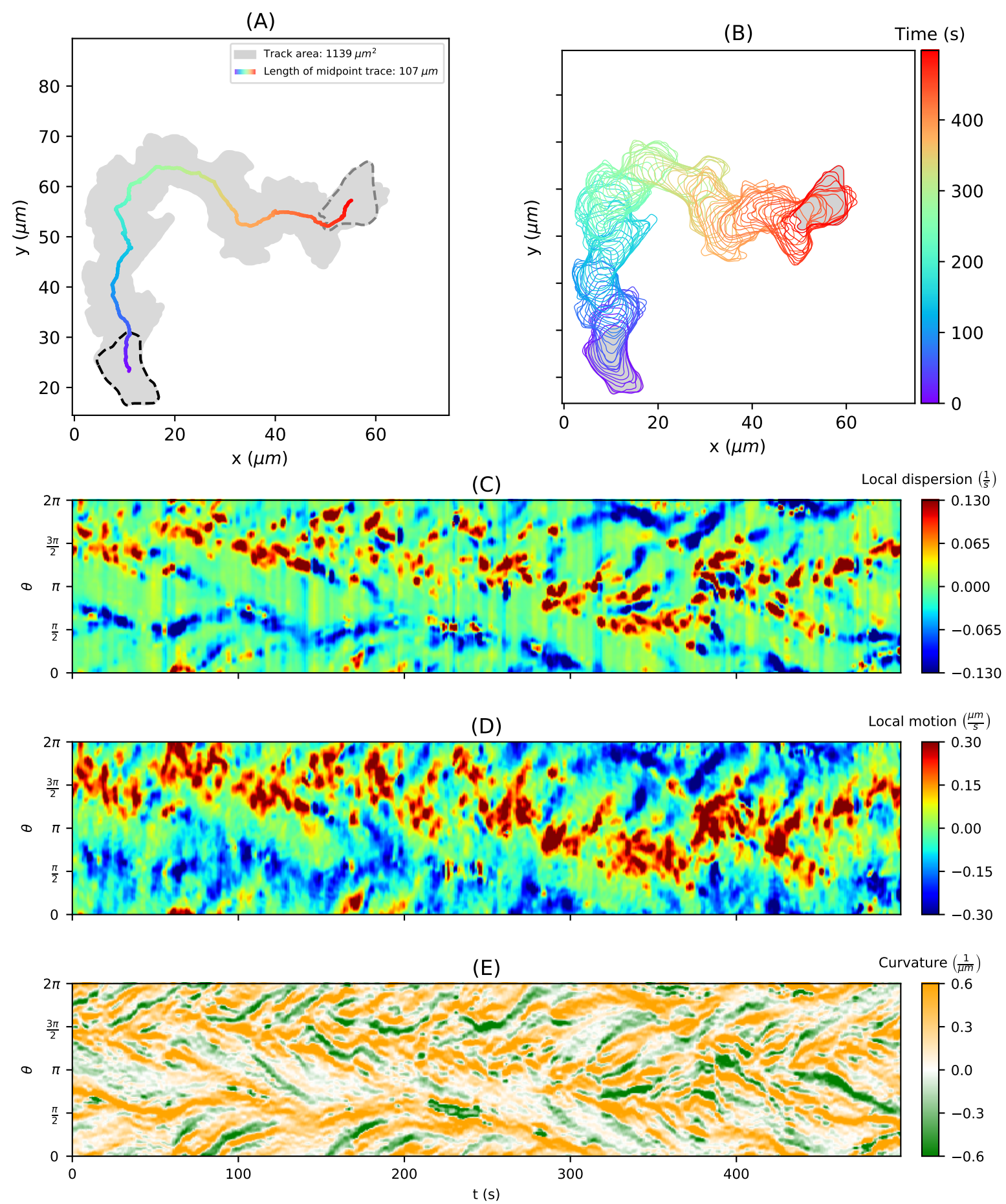

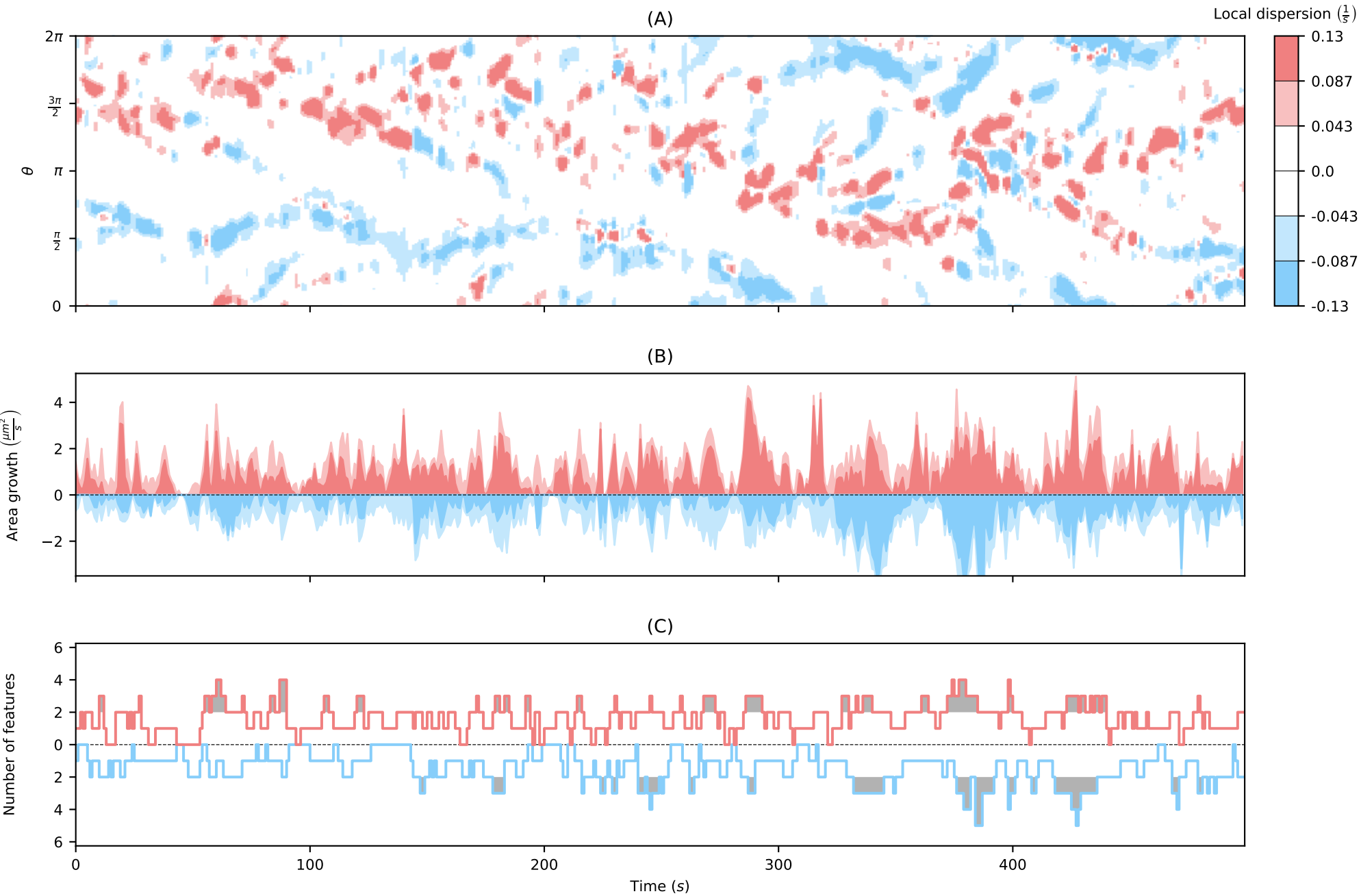

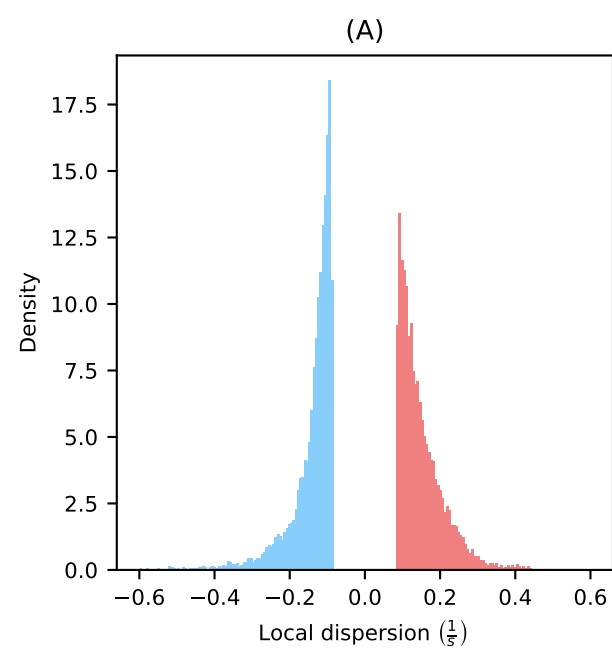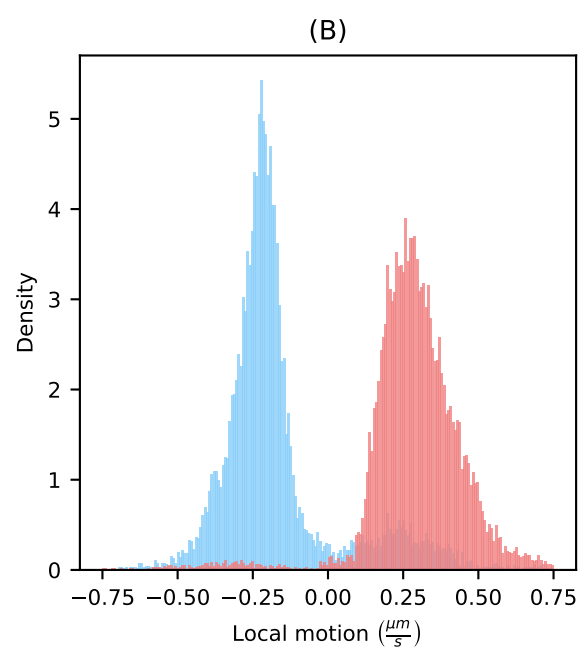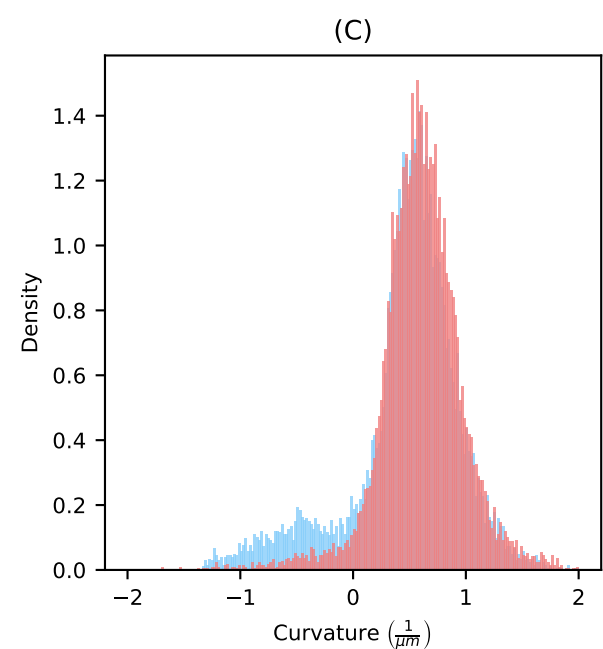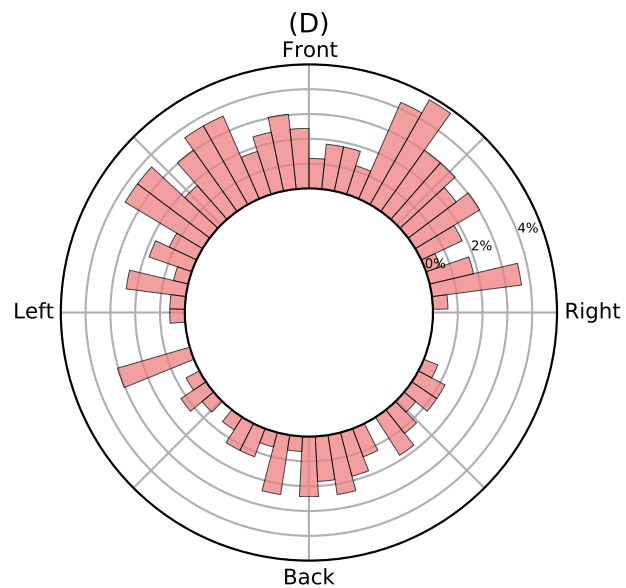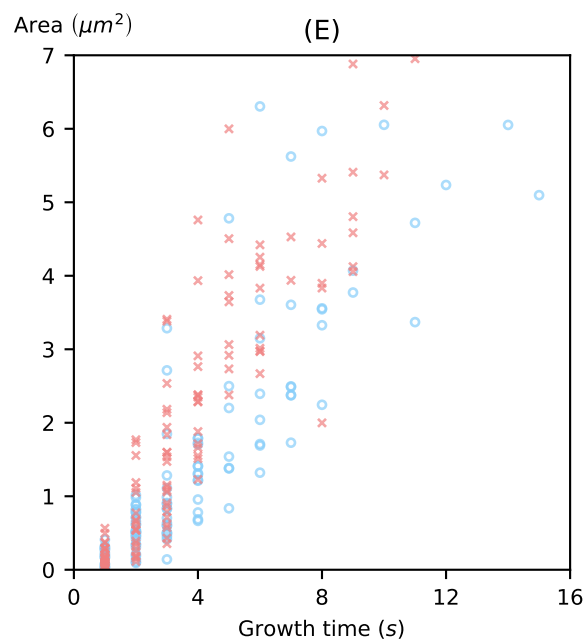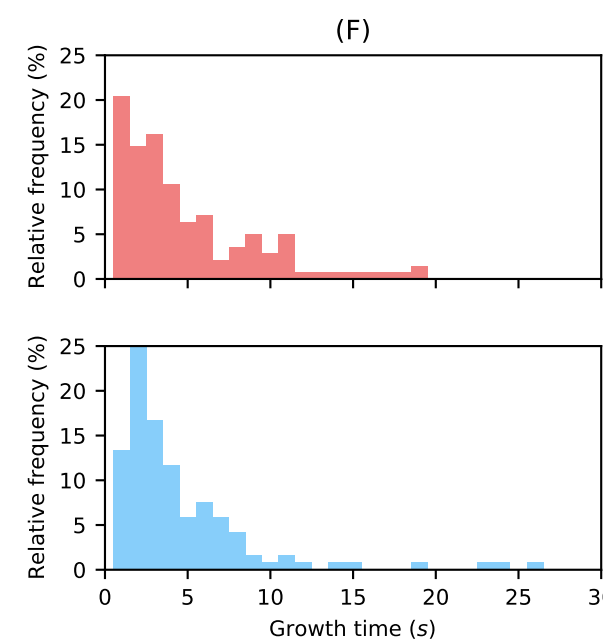

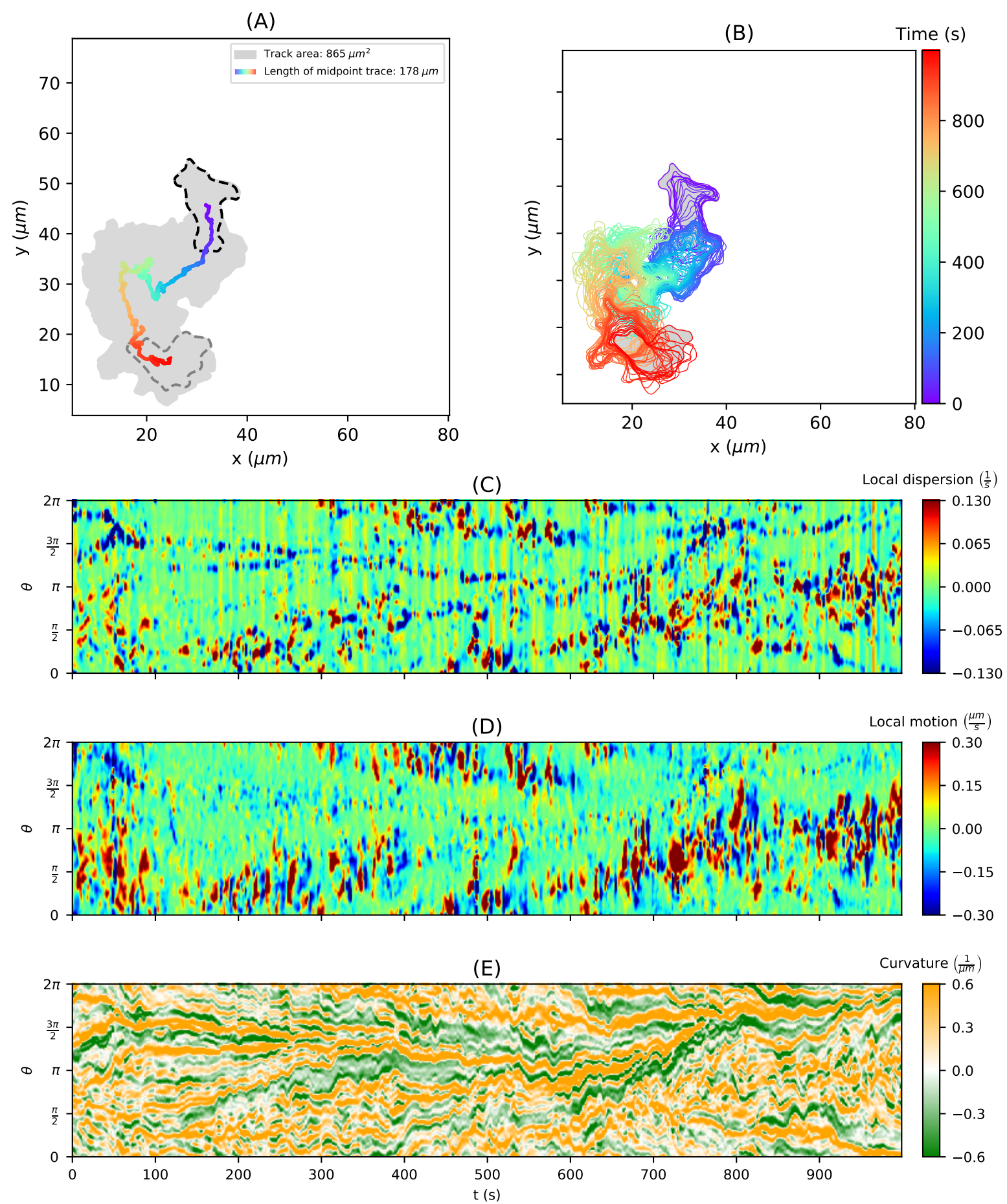

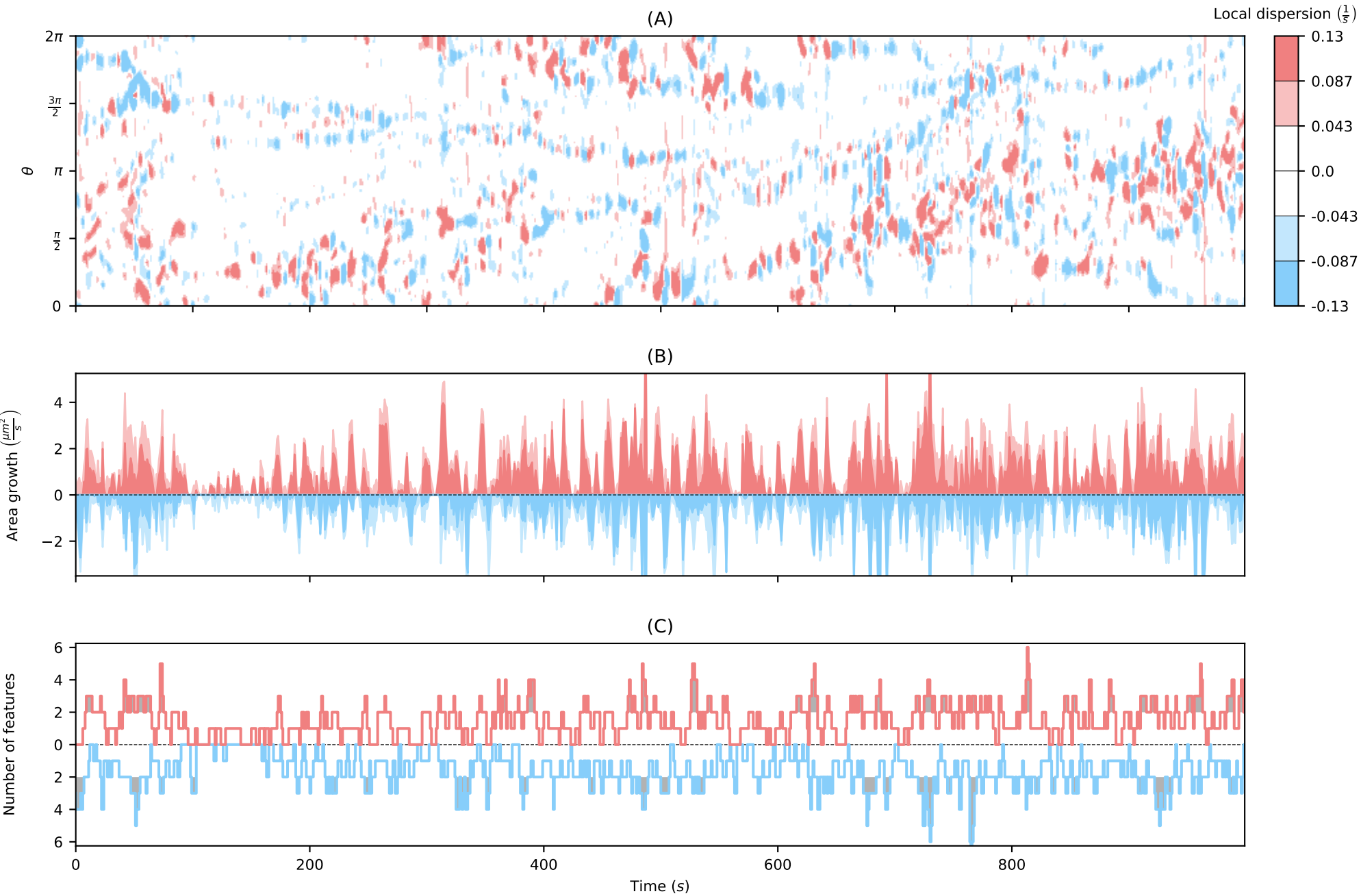

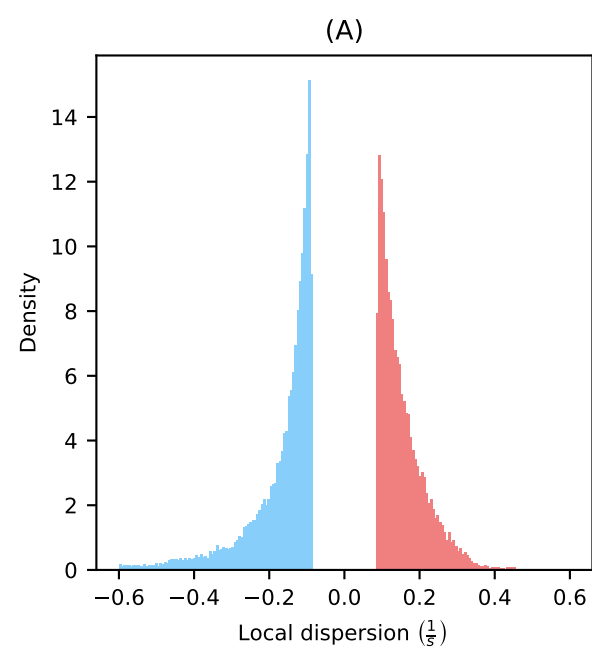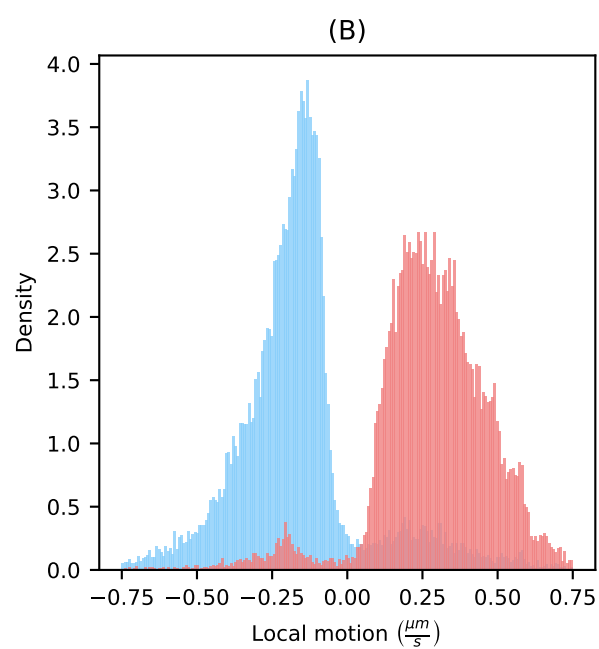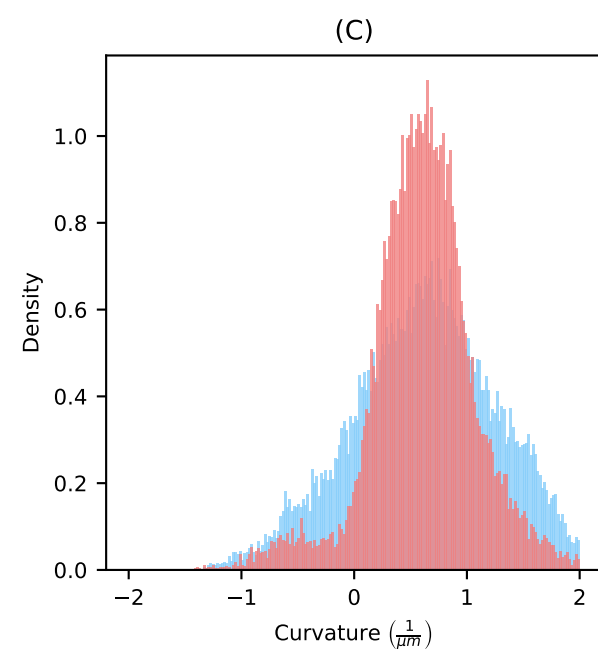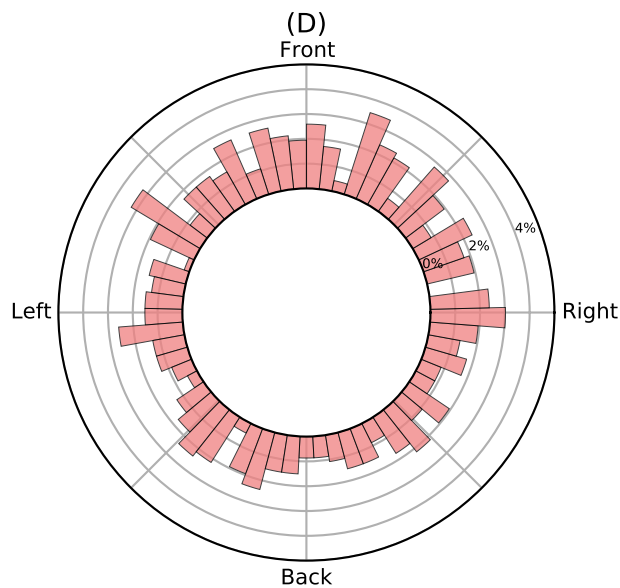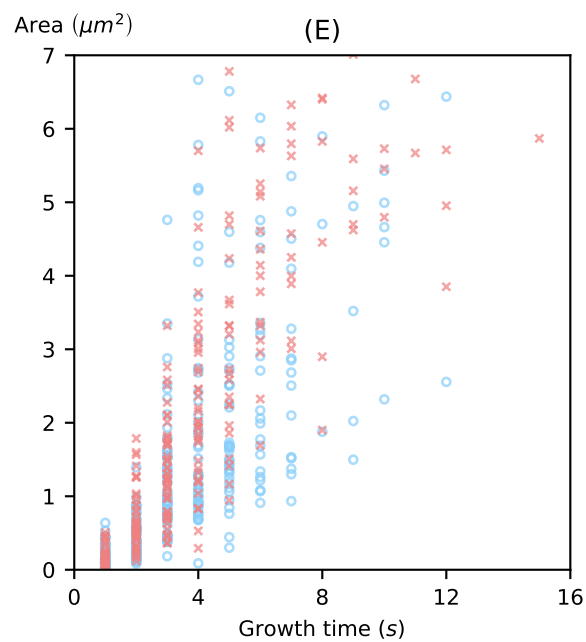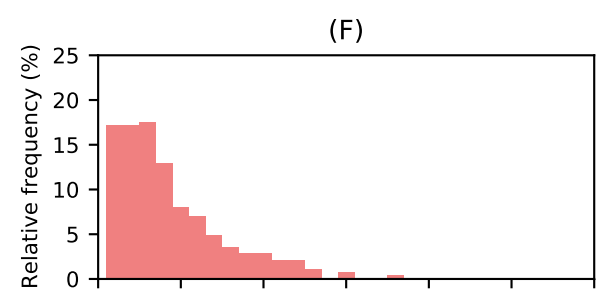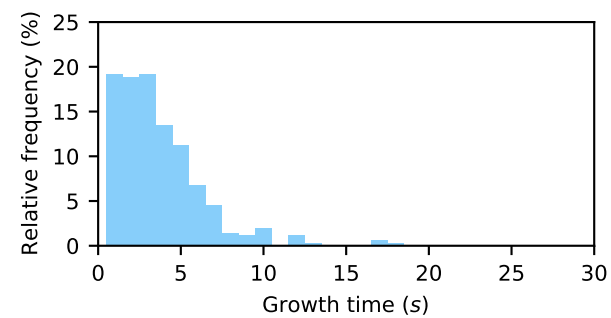

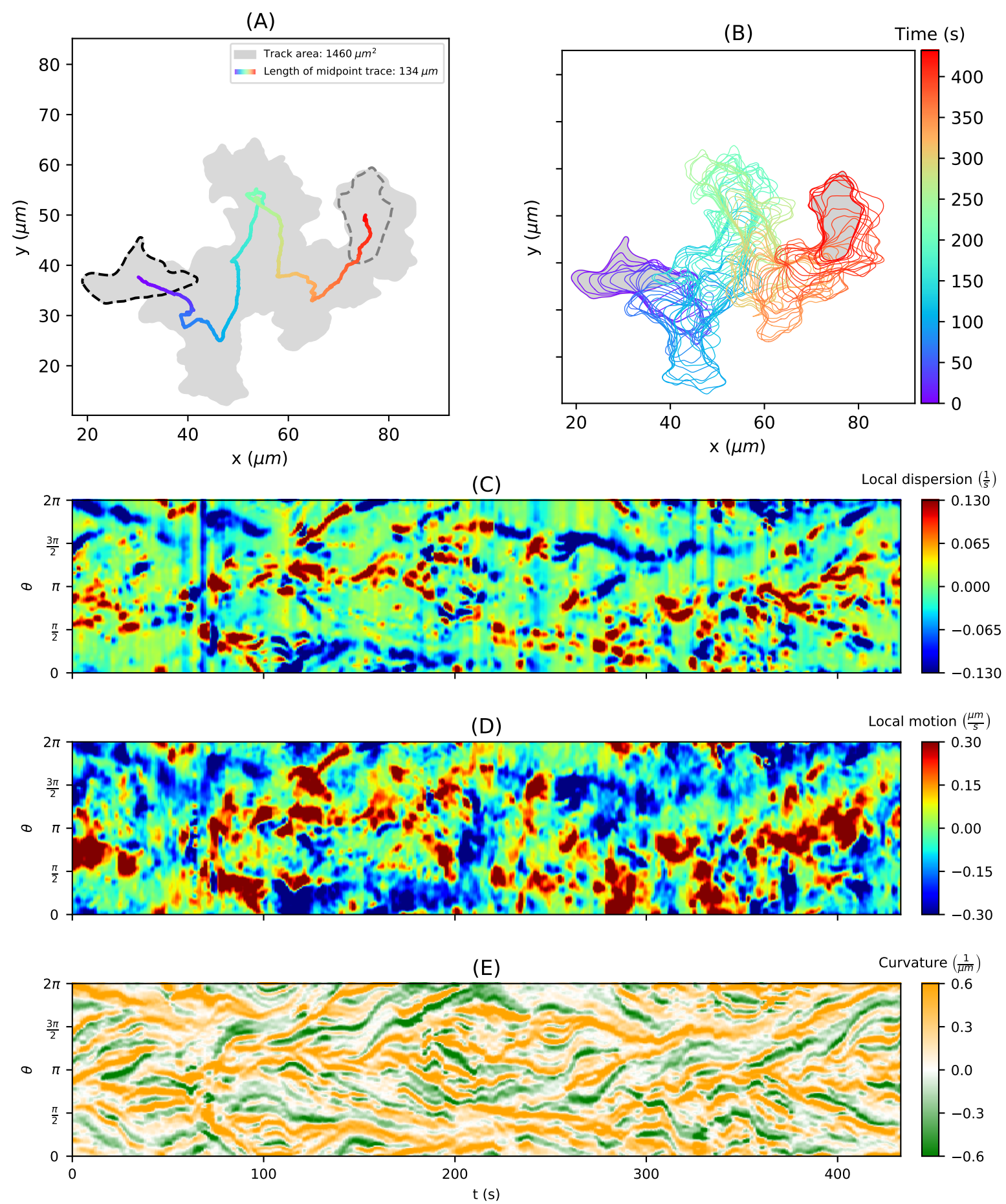

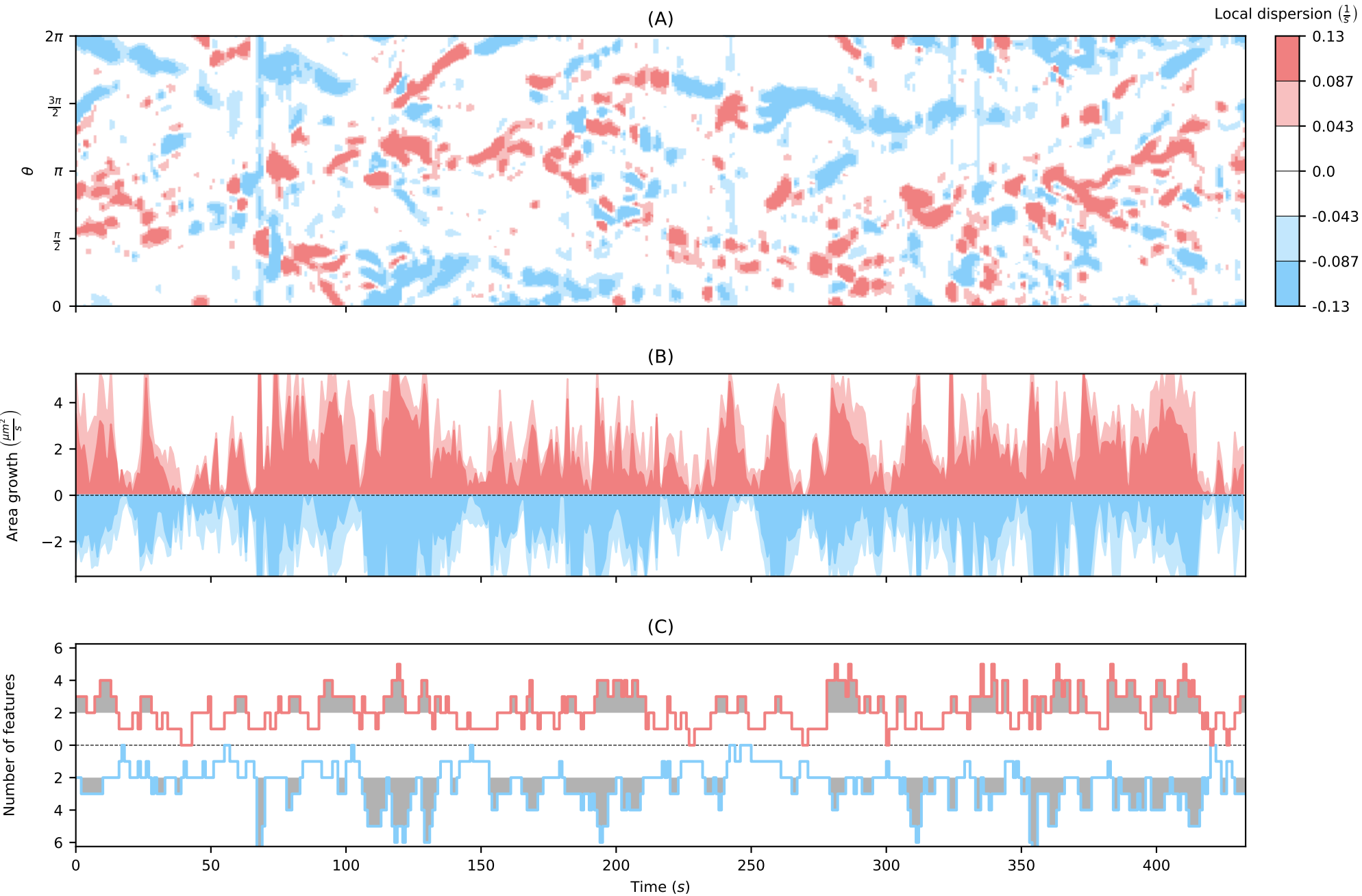

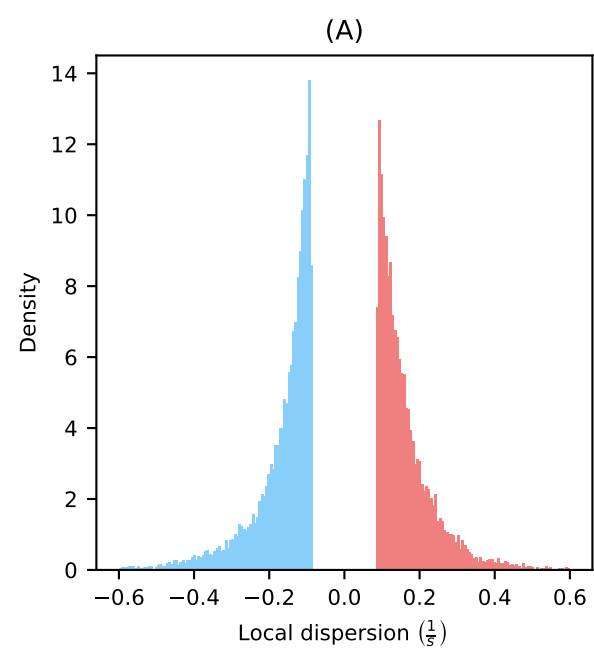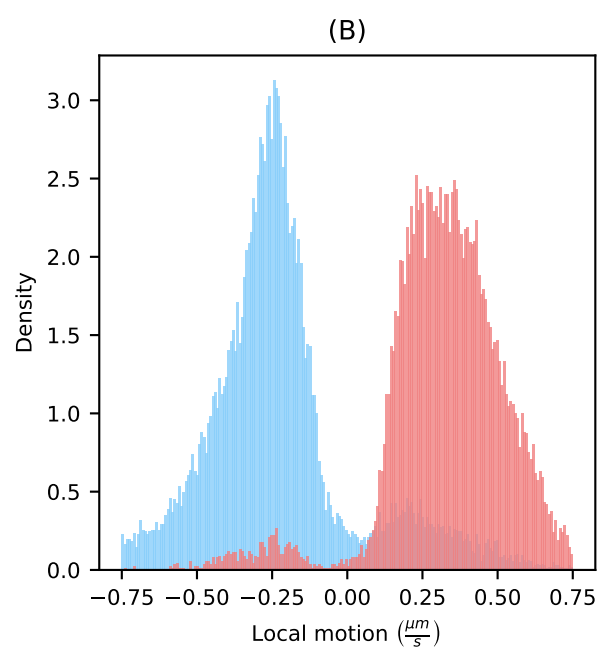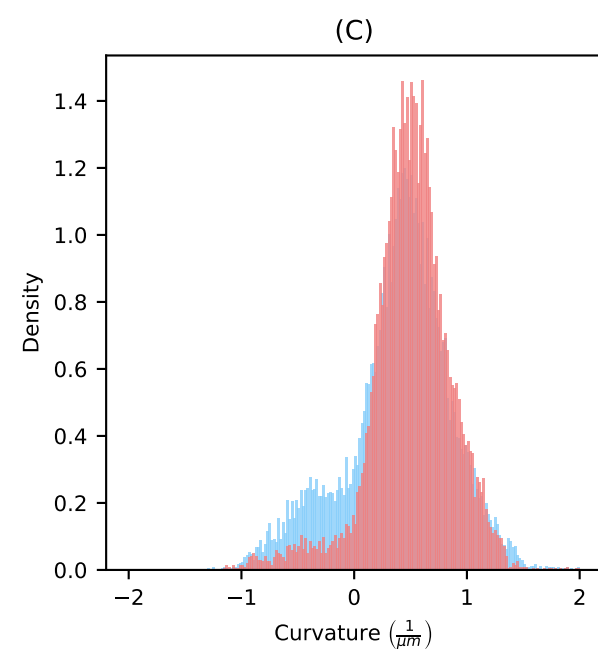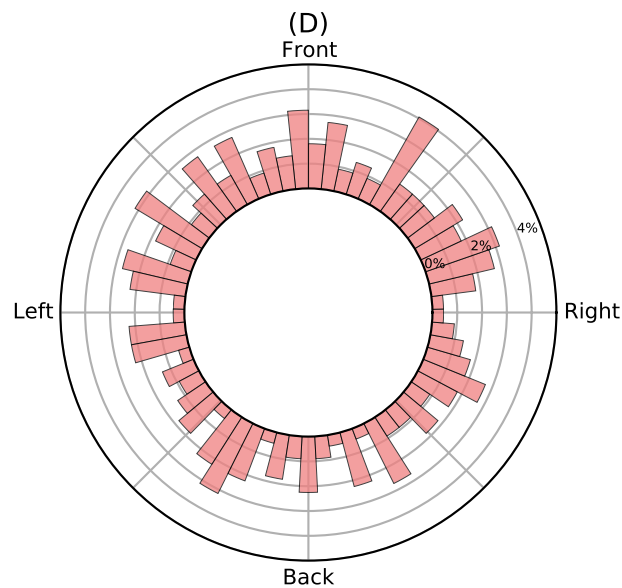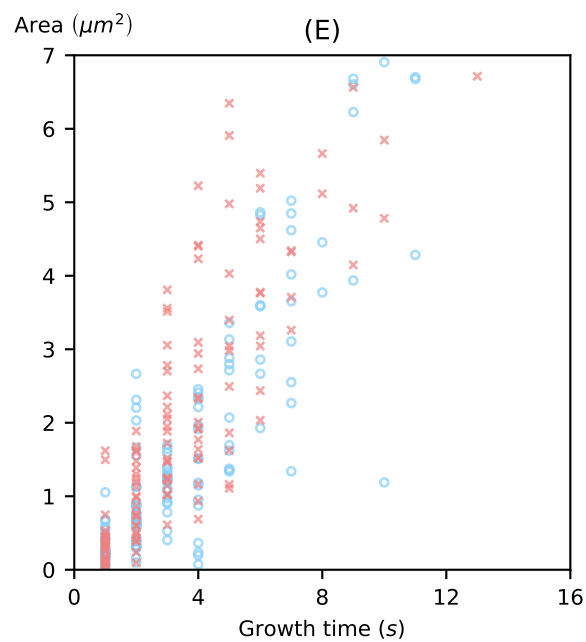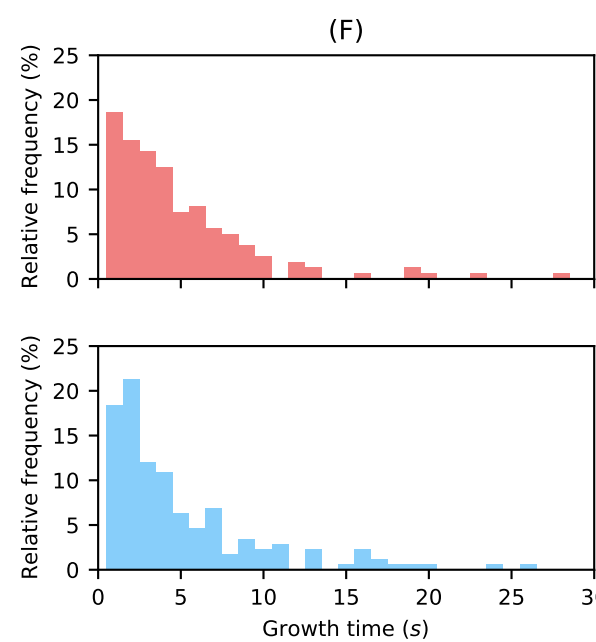

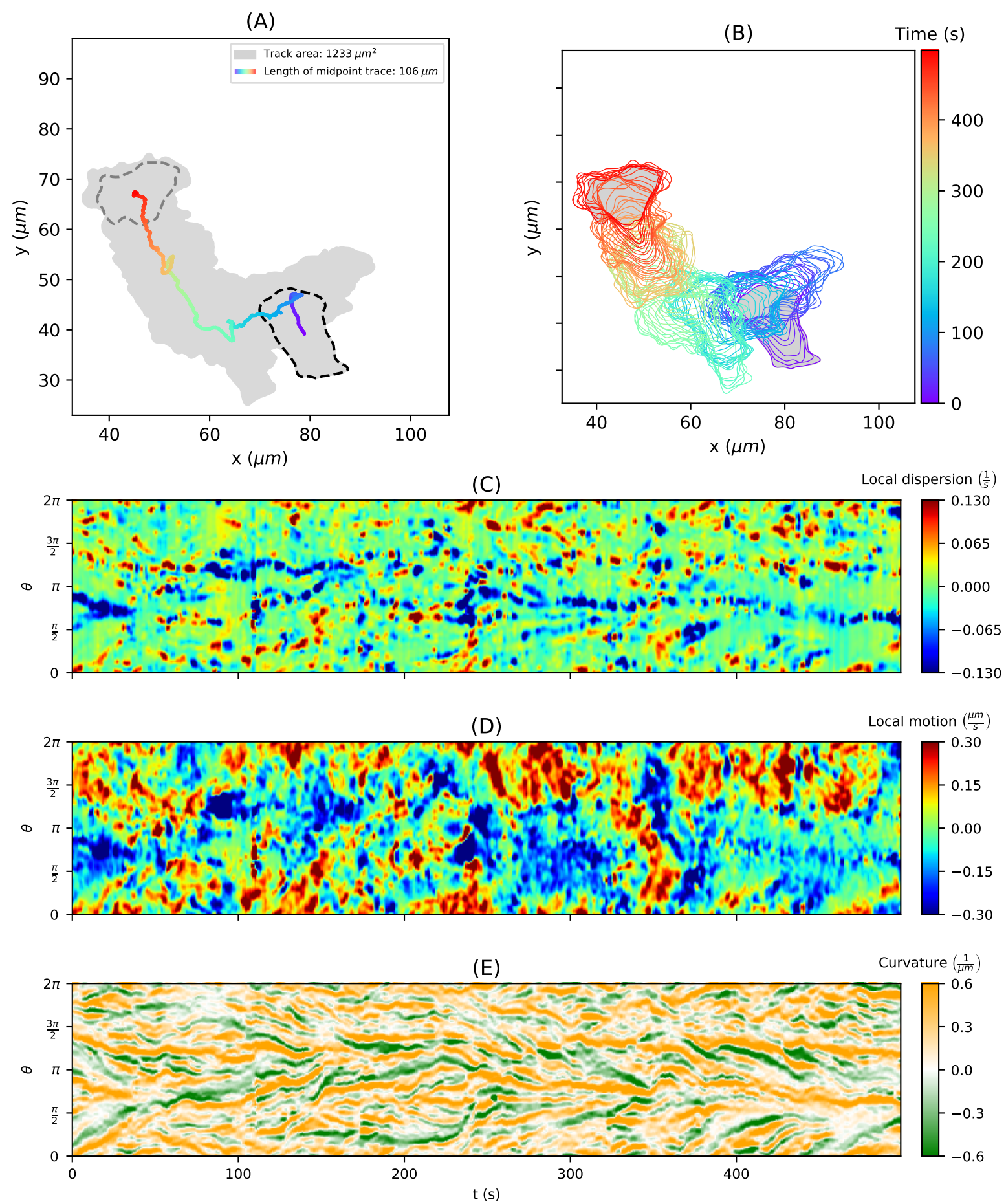

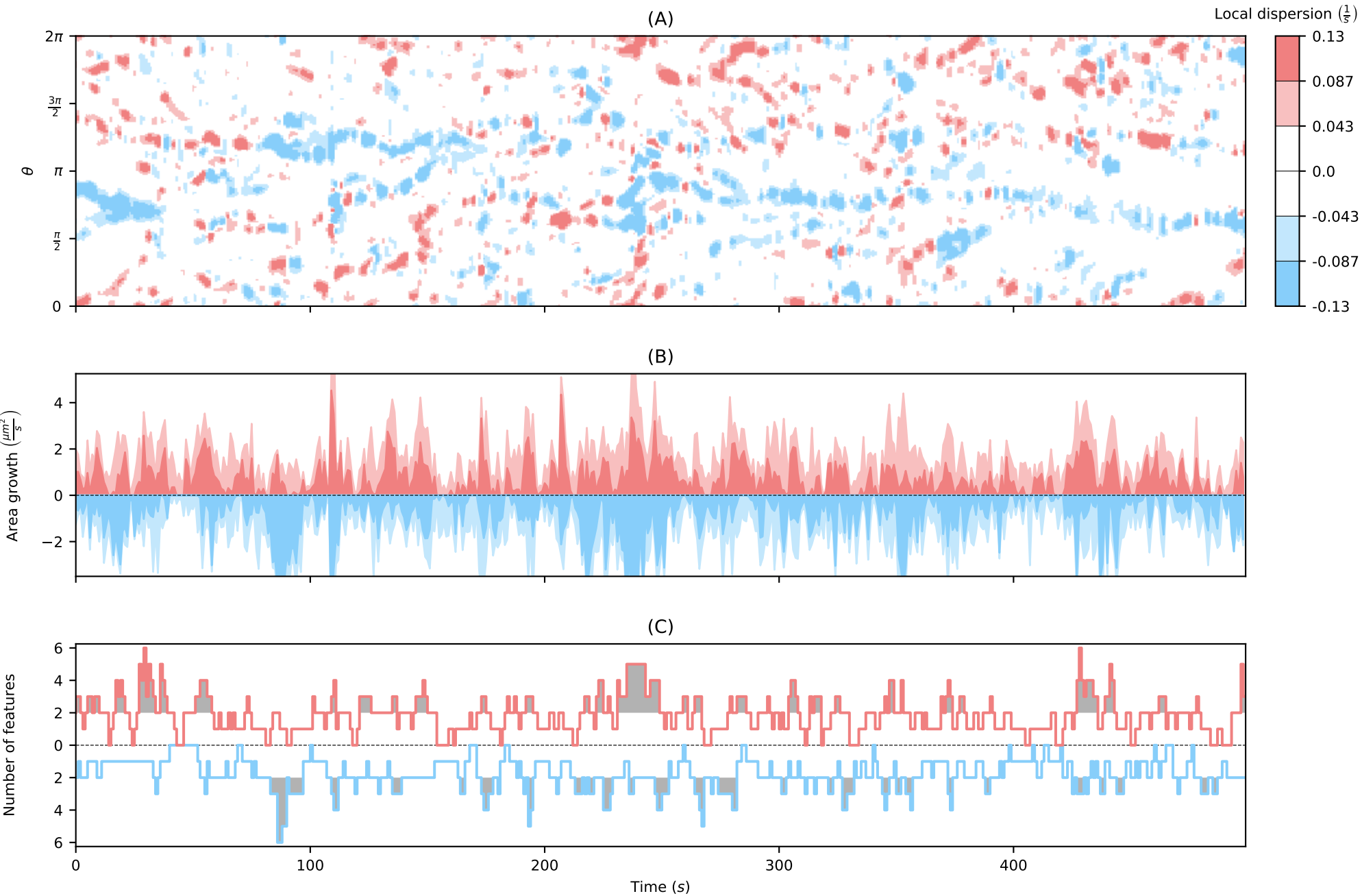

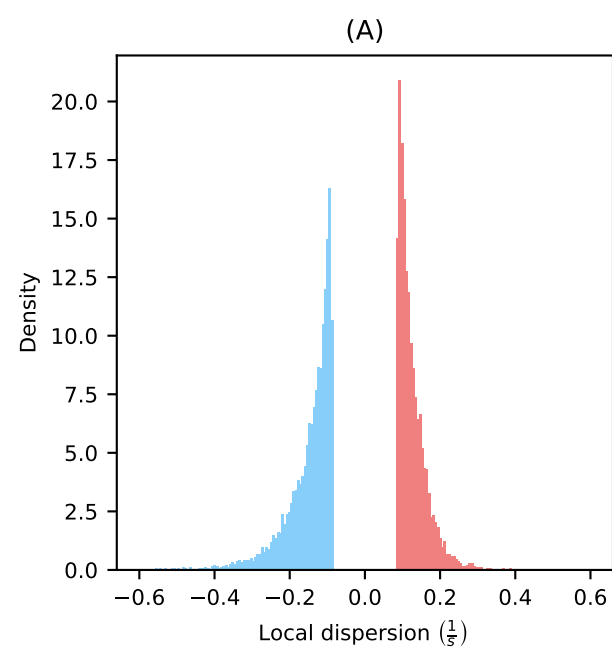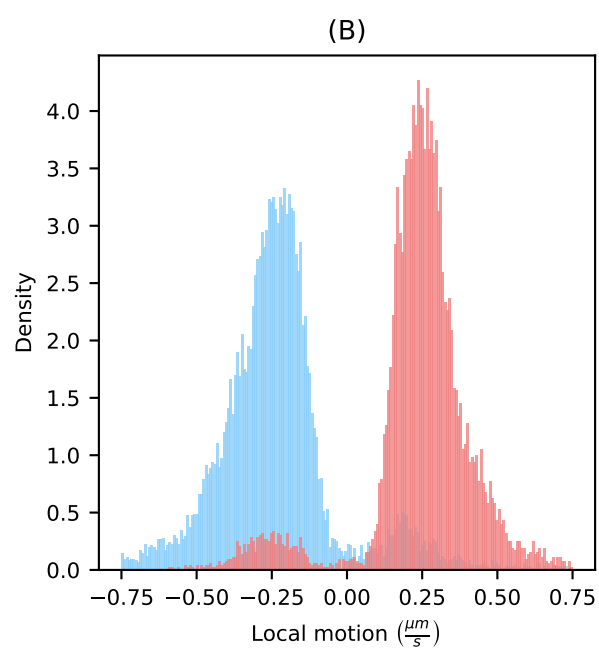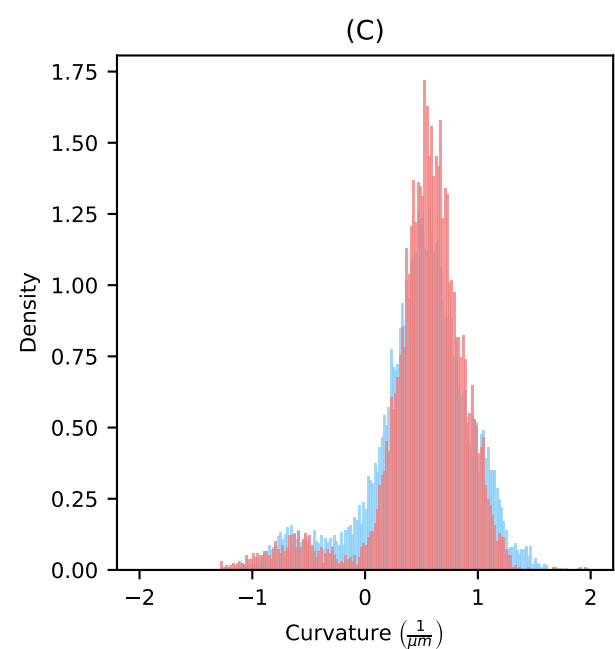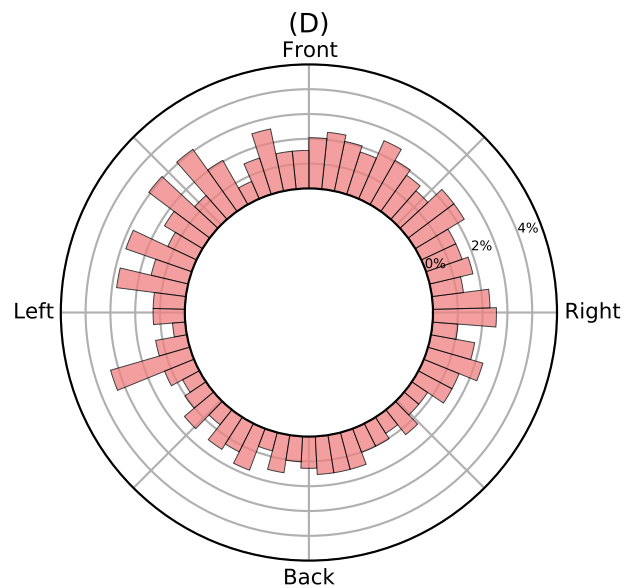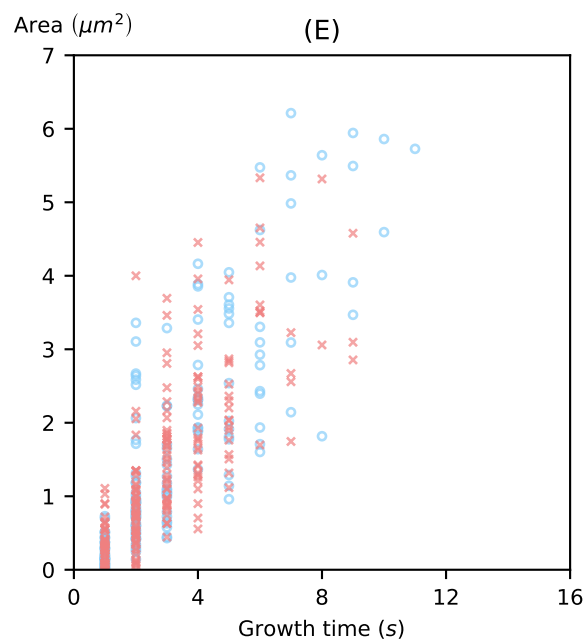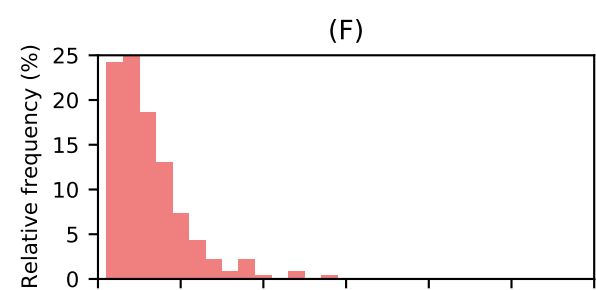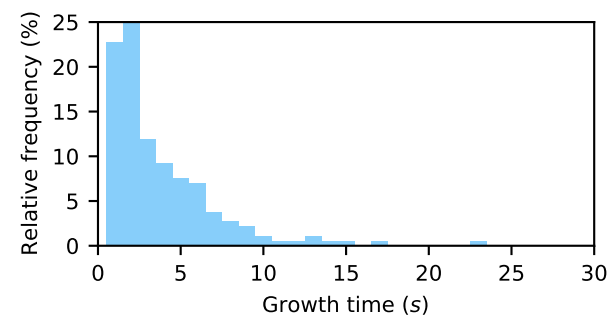

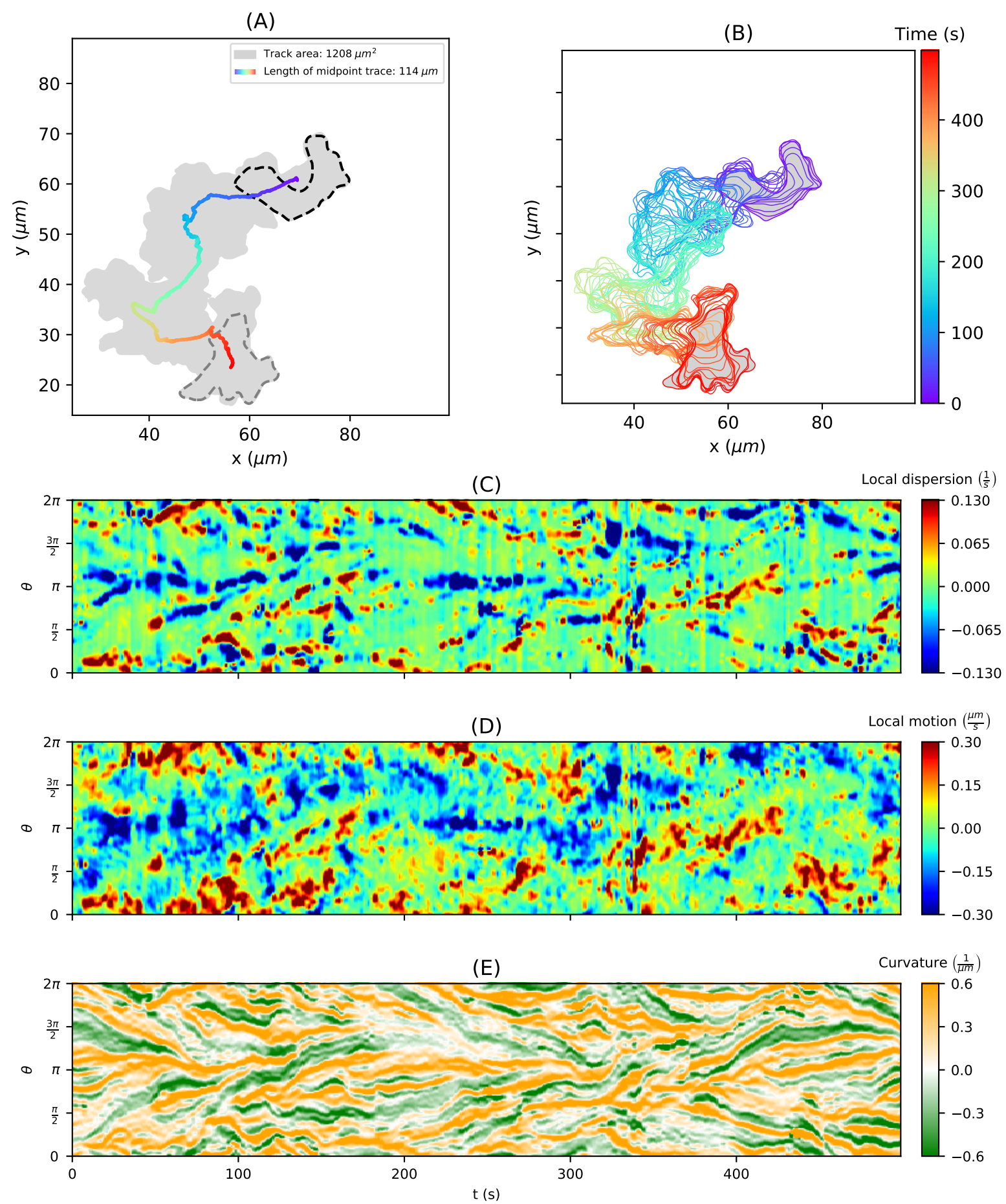

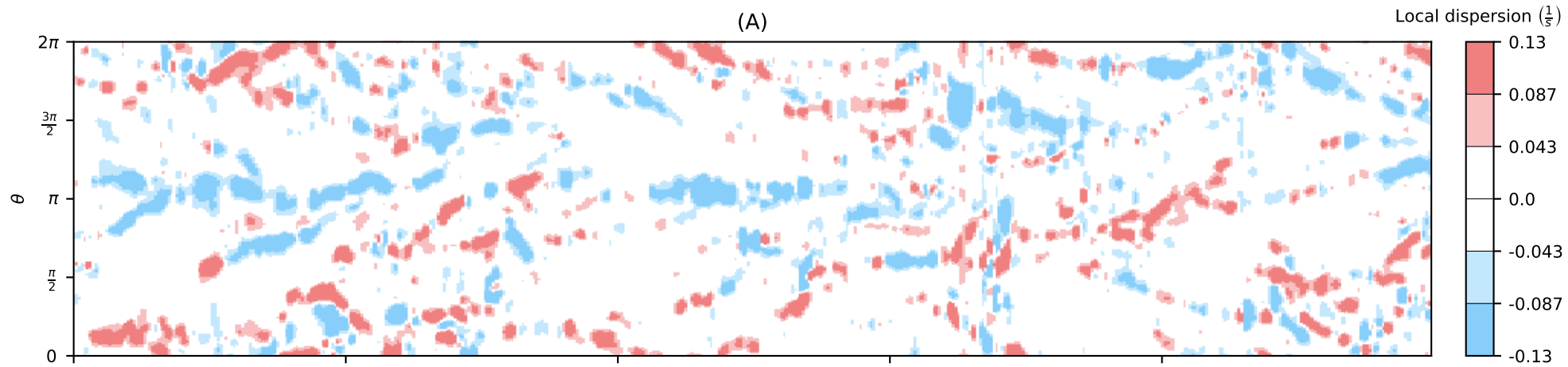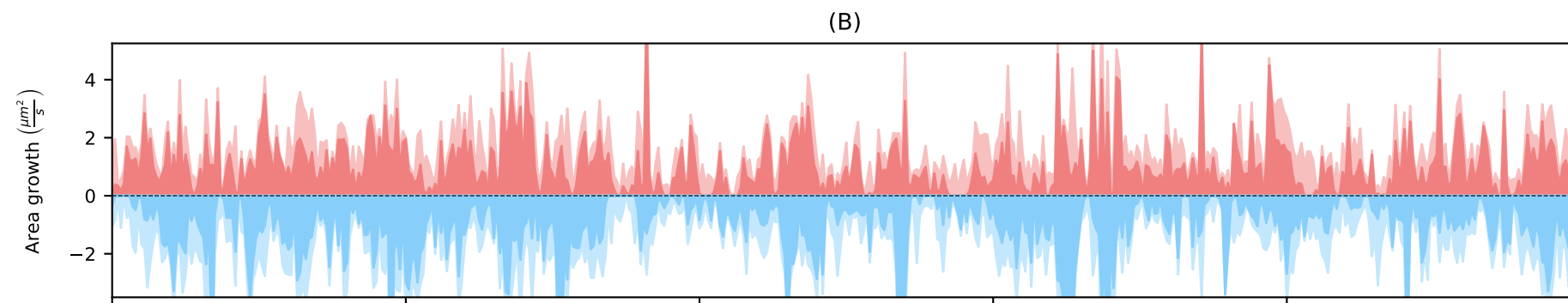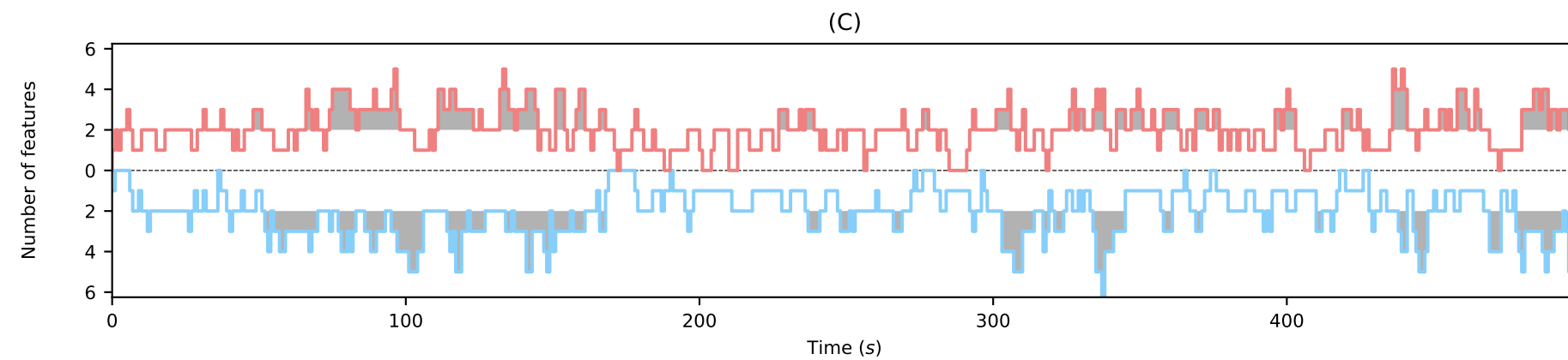

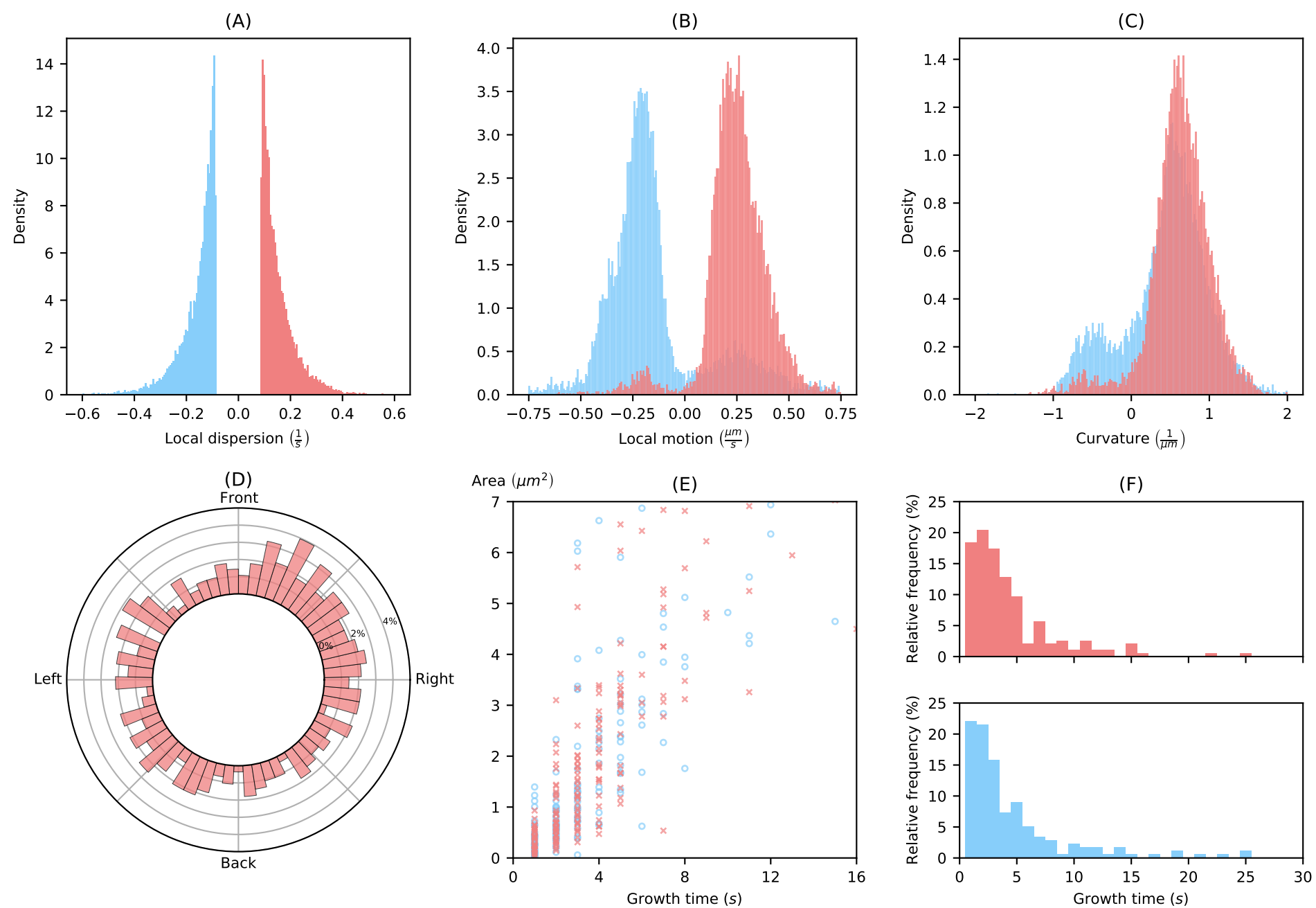

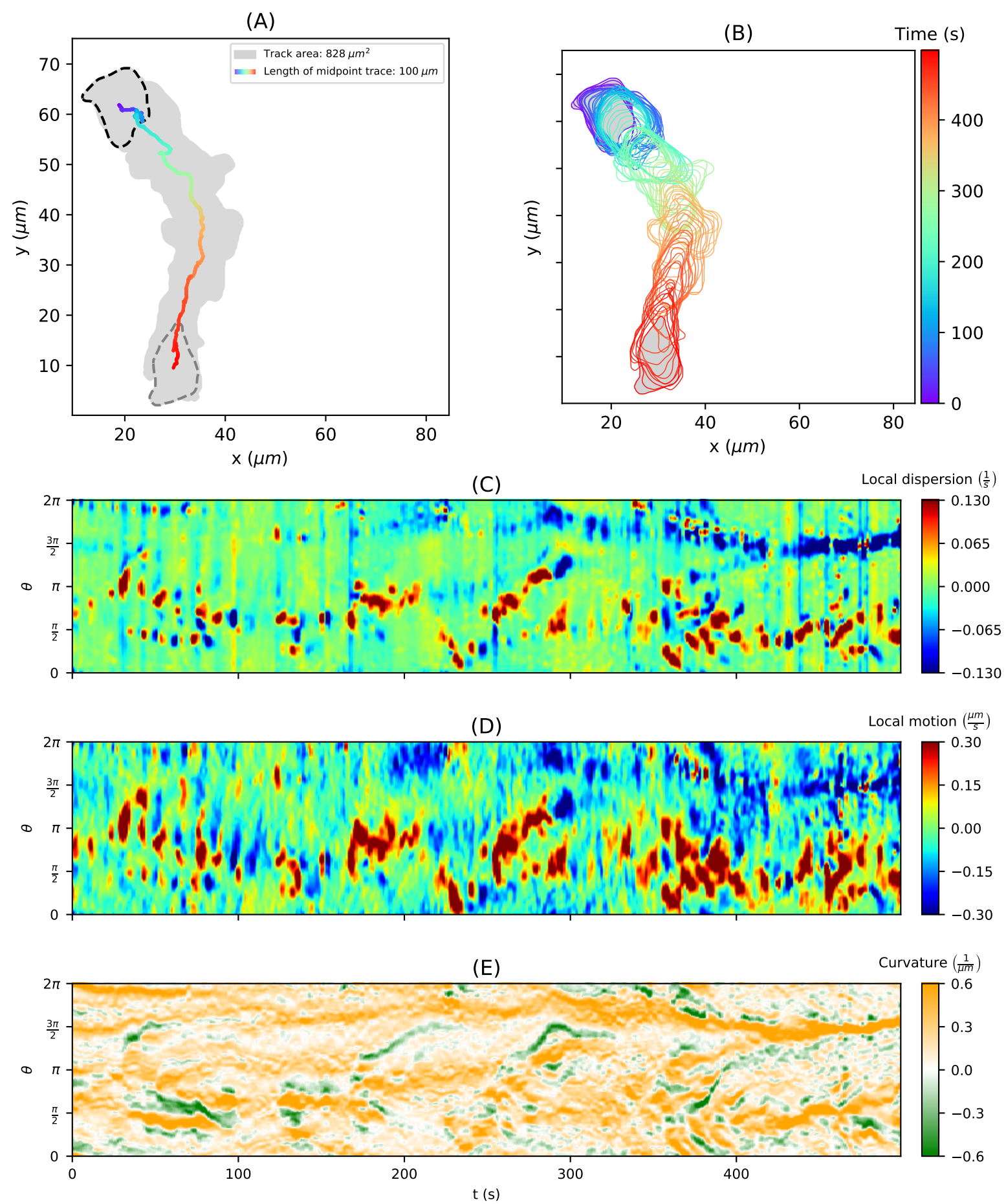

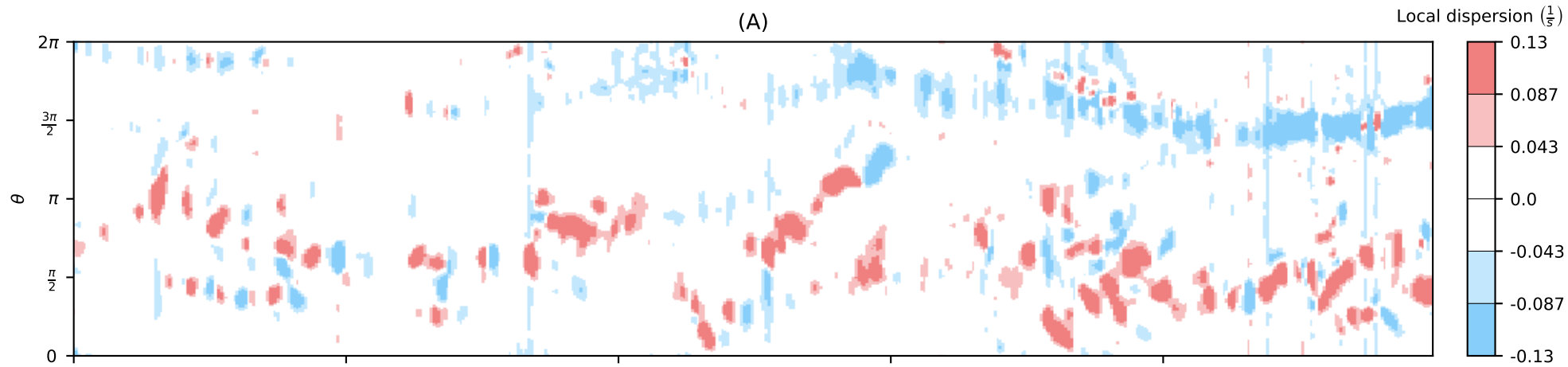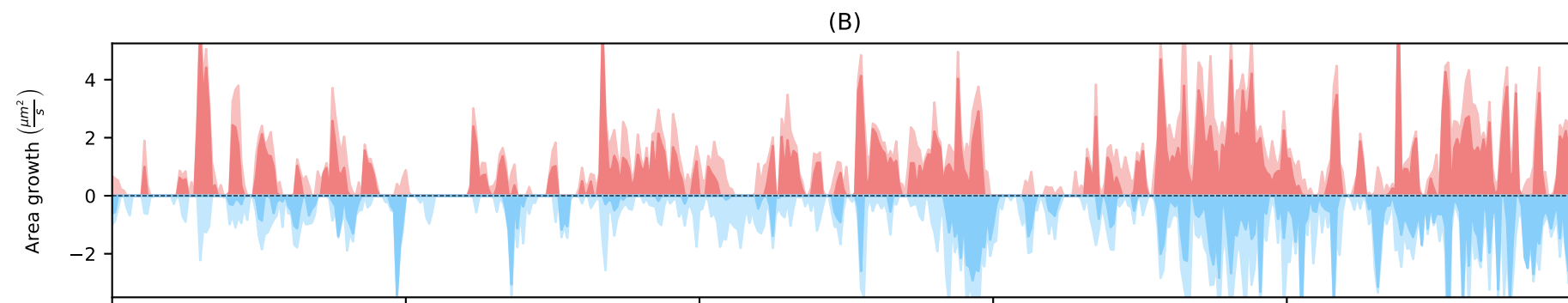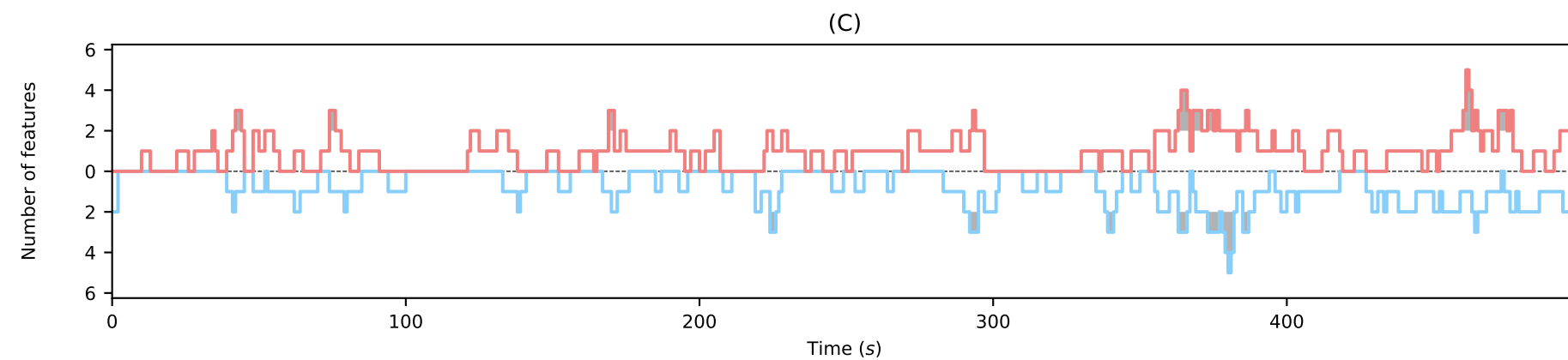

(A)

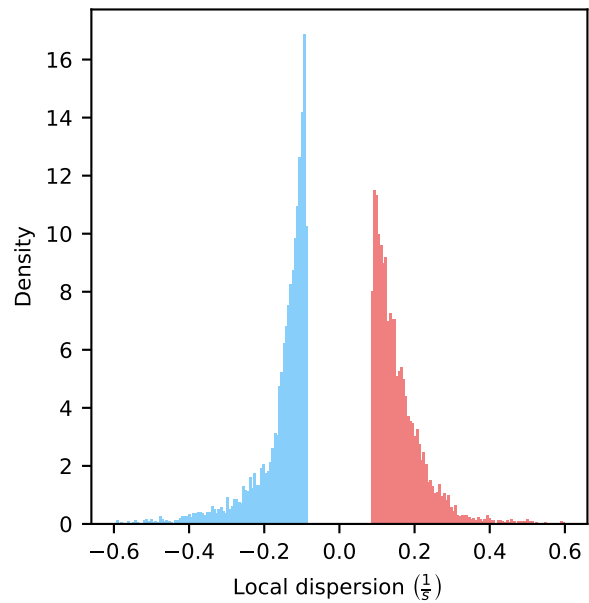

(B)

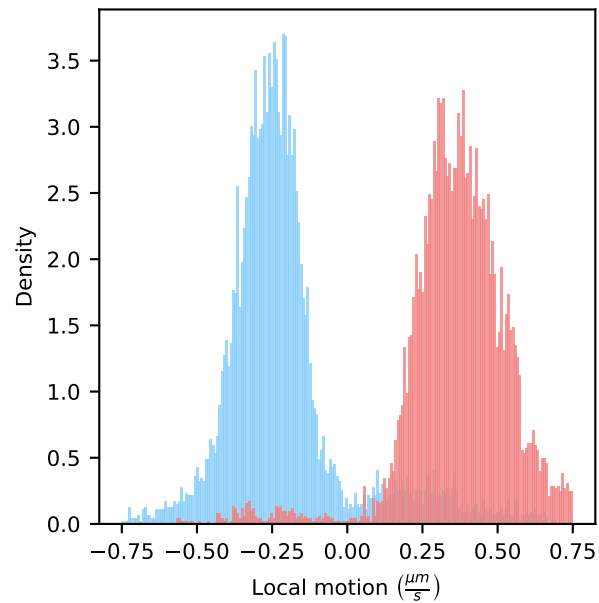

(C)

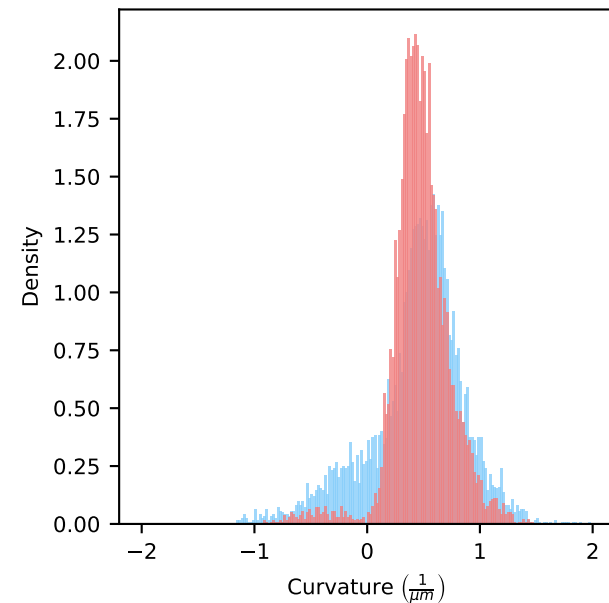

(D)

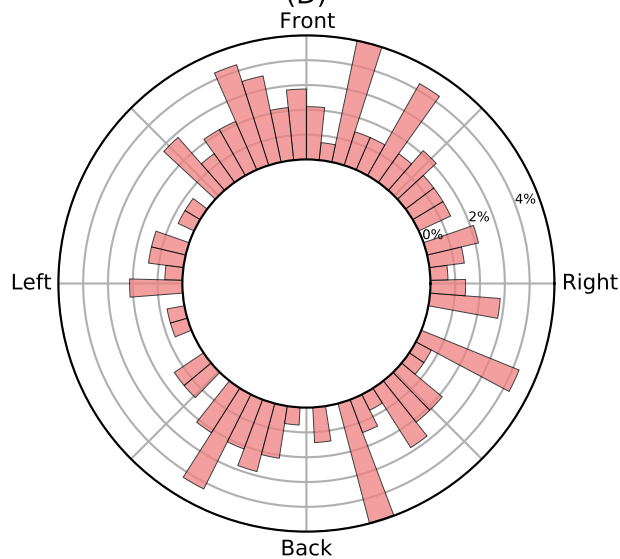

(E)

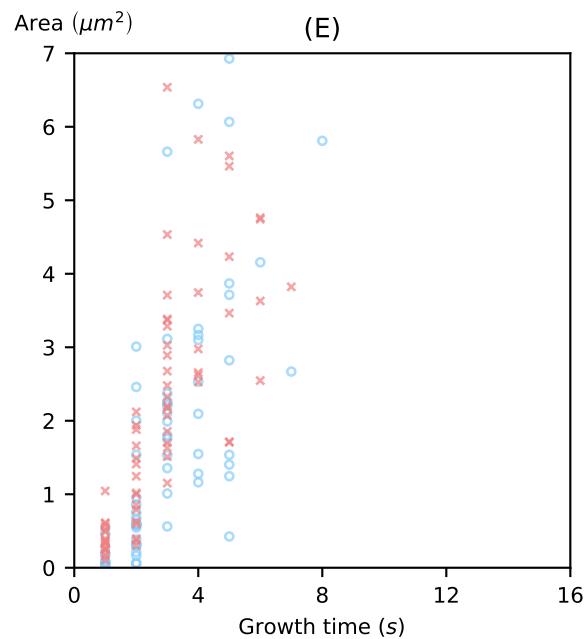

(F)

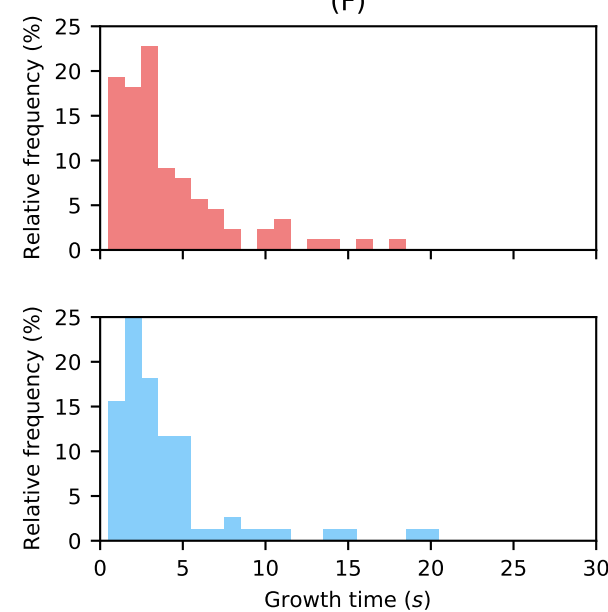

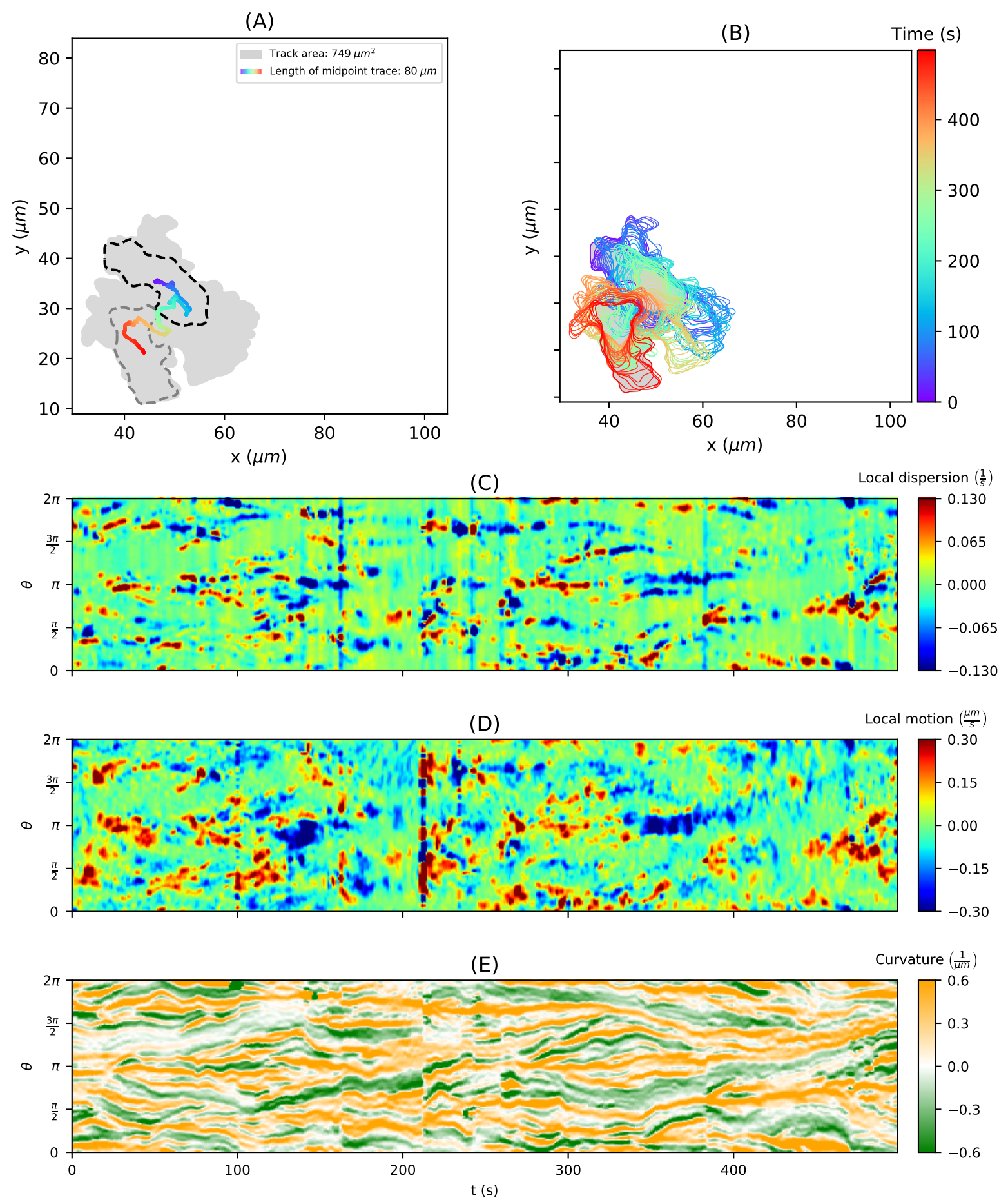

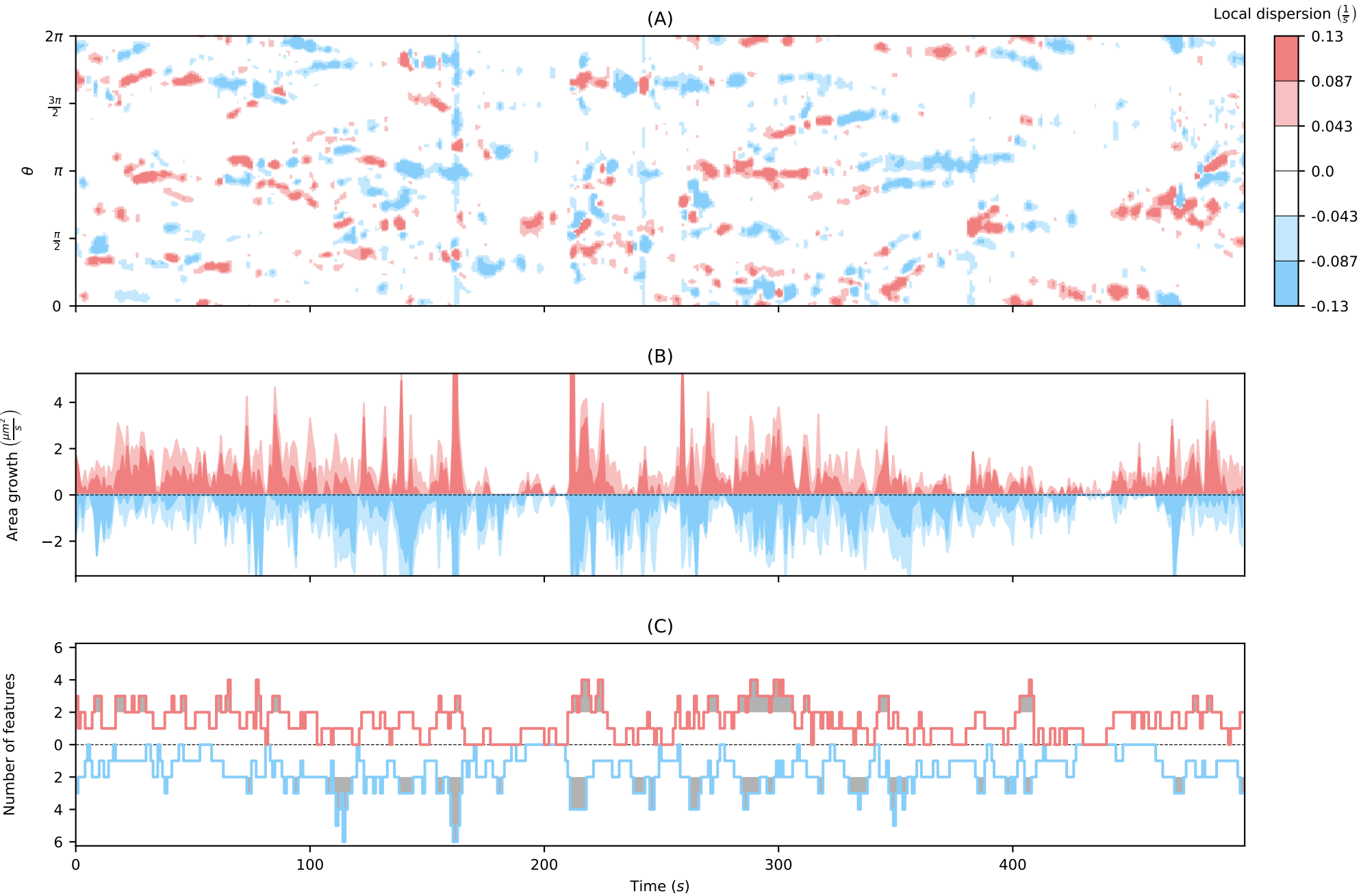

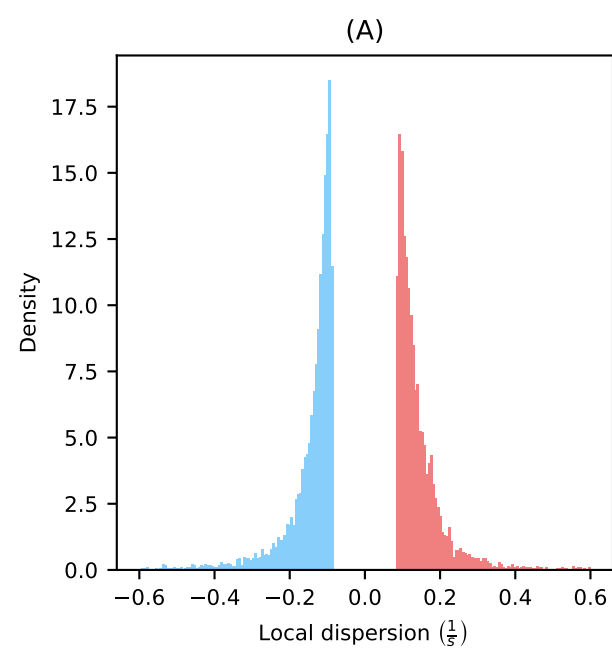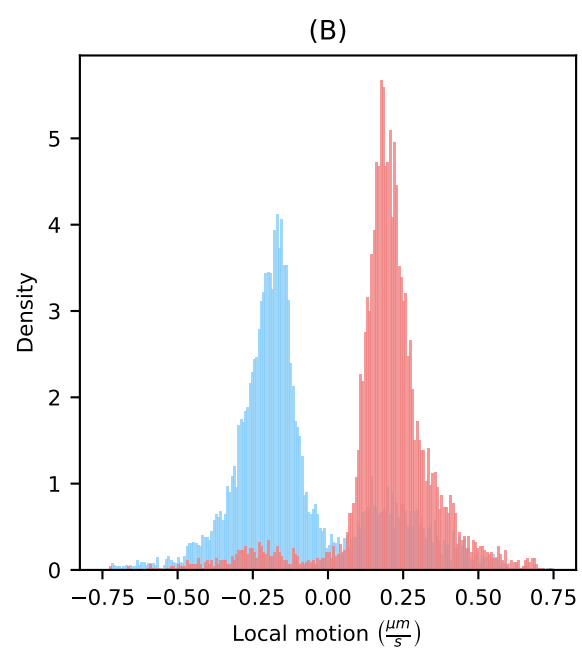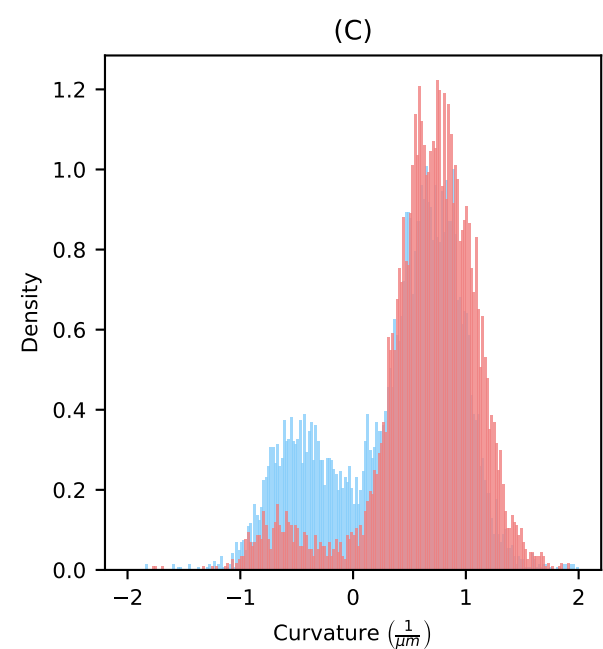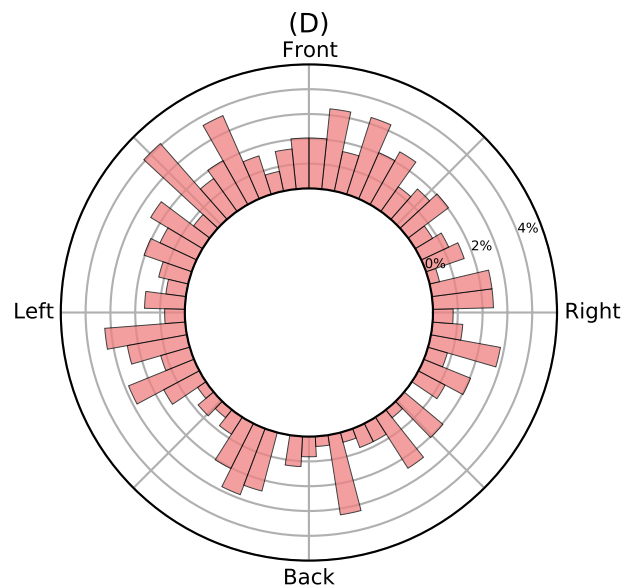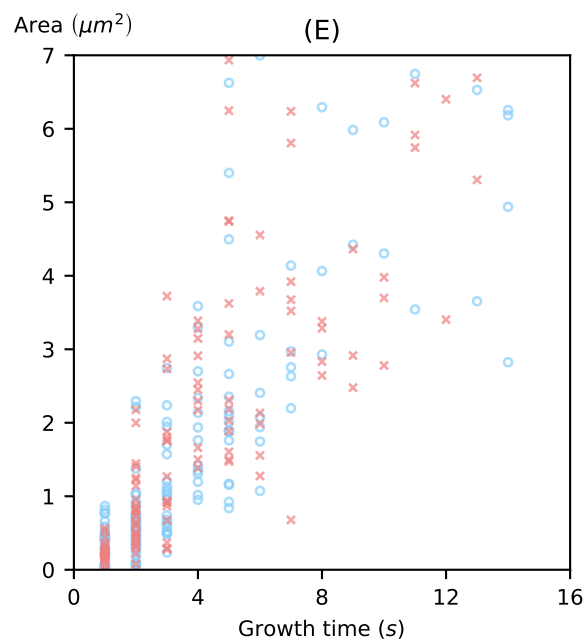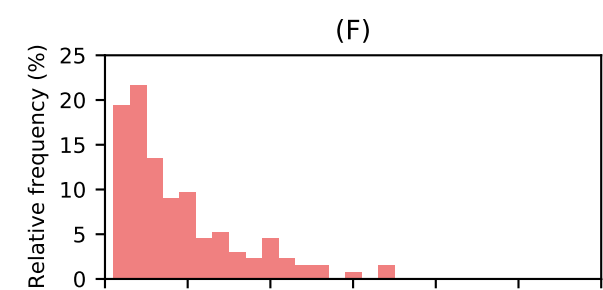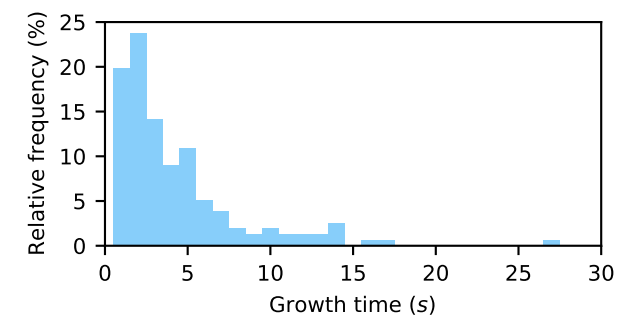

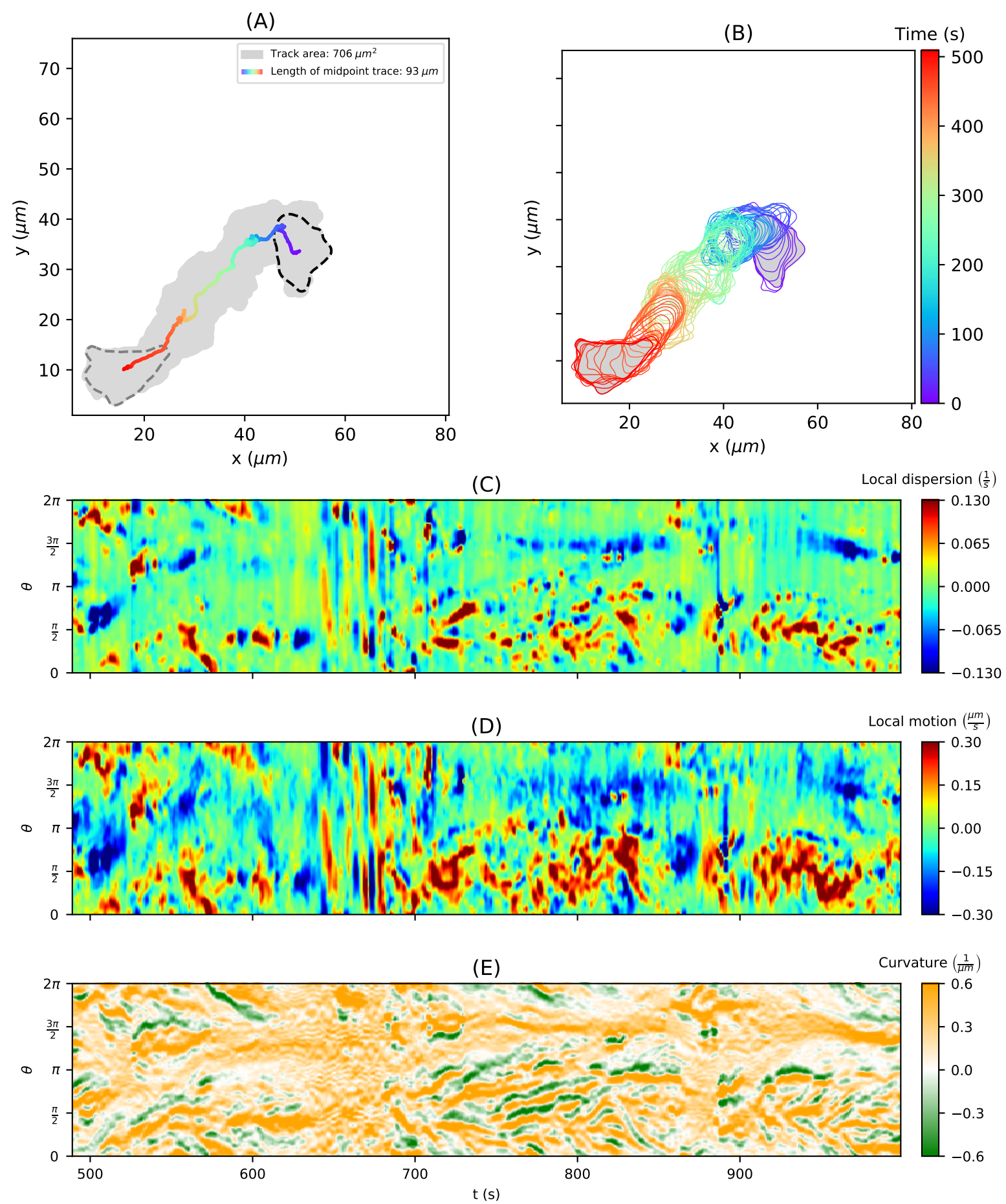

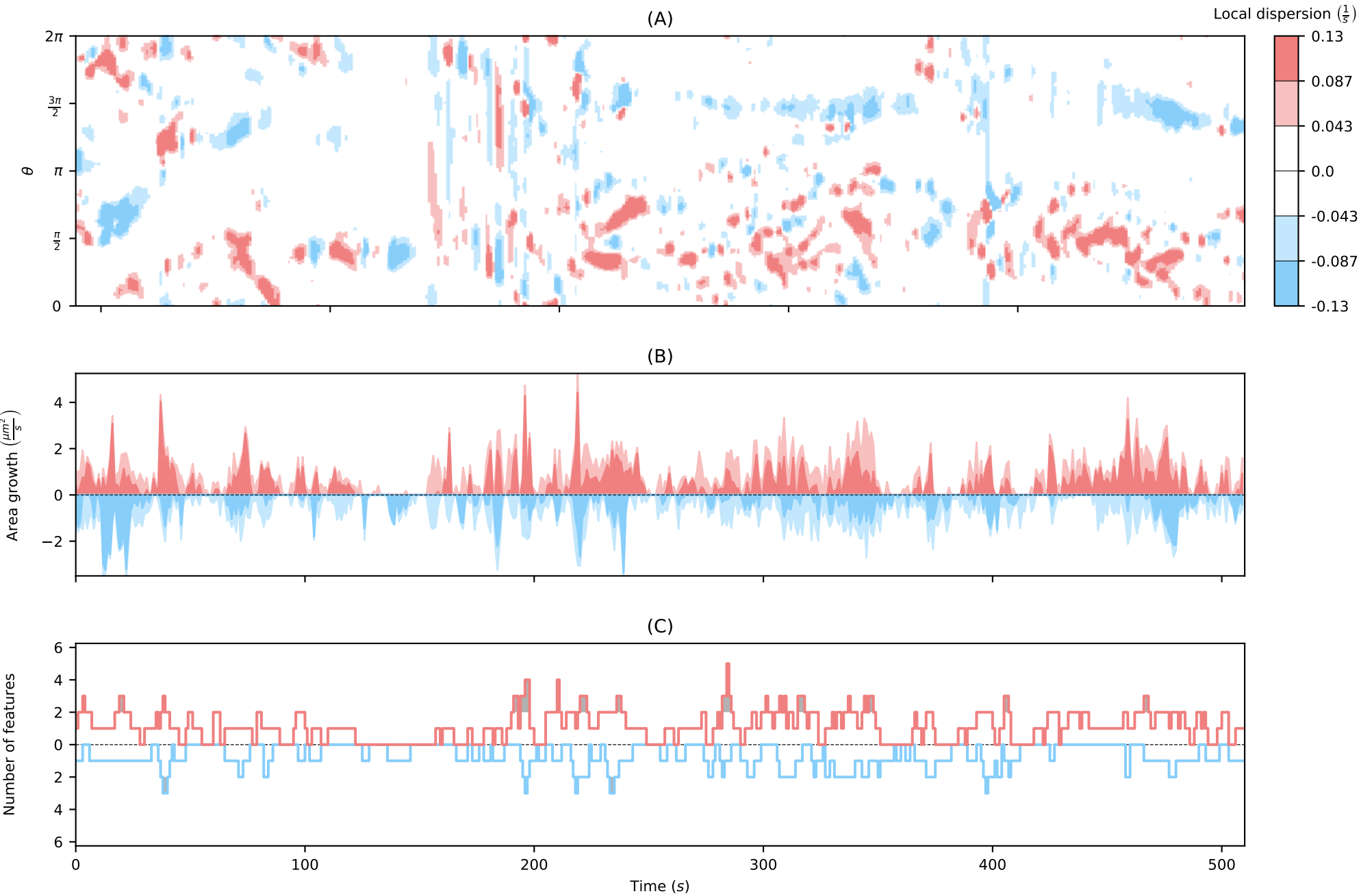

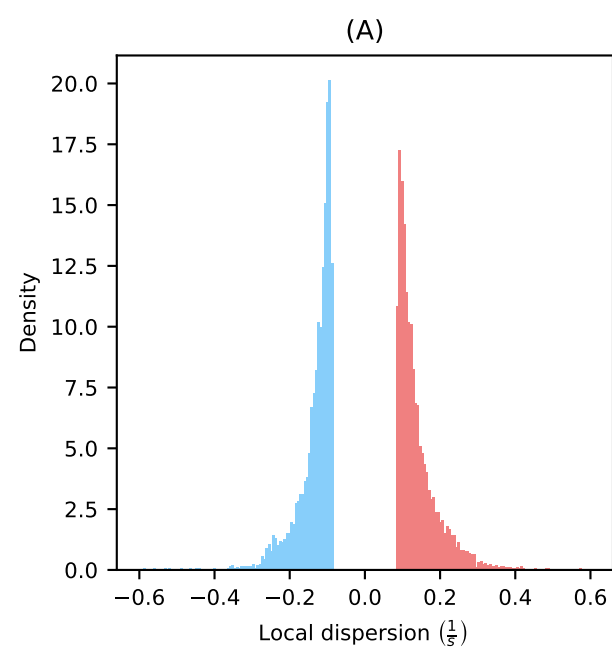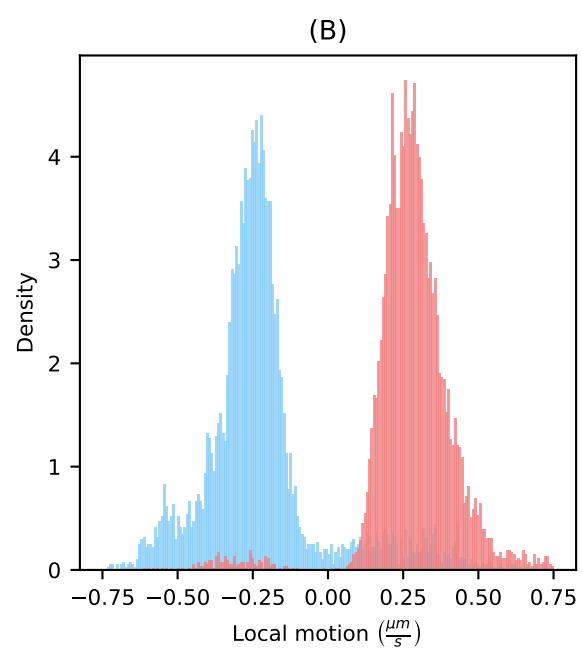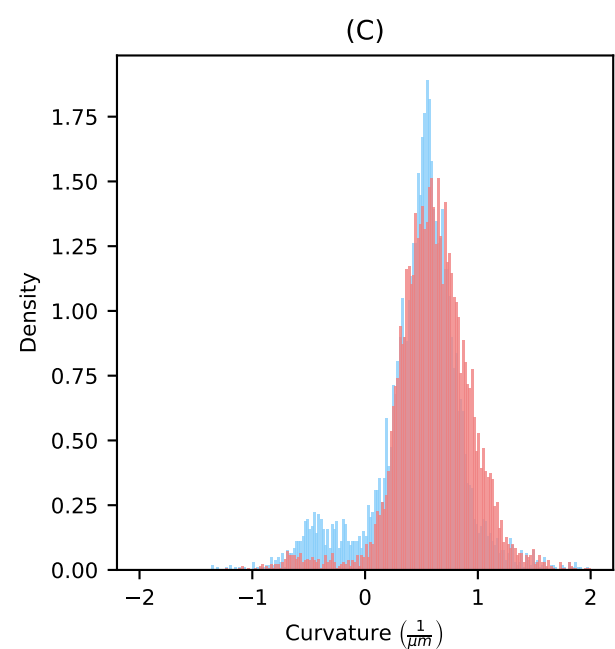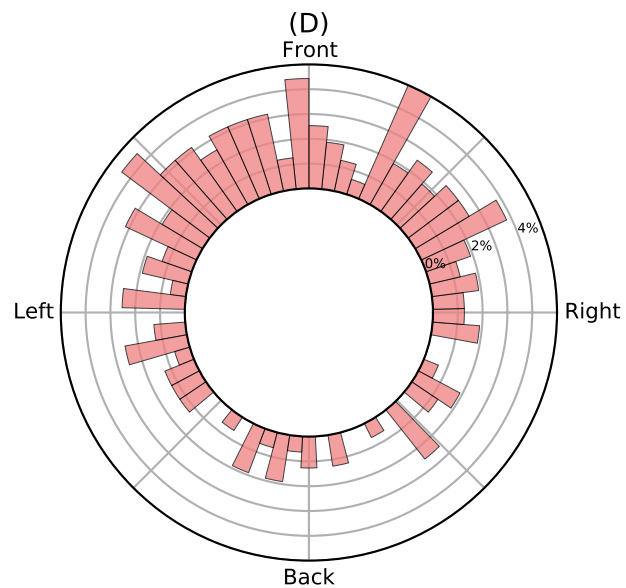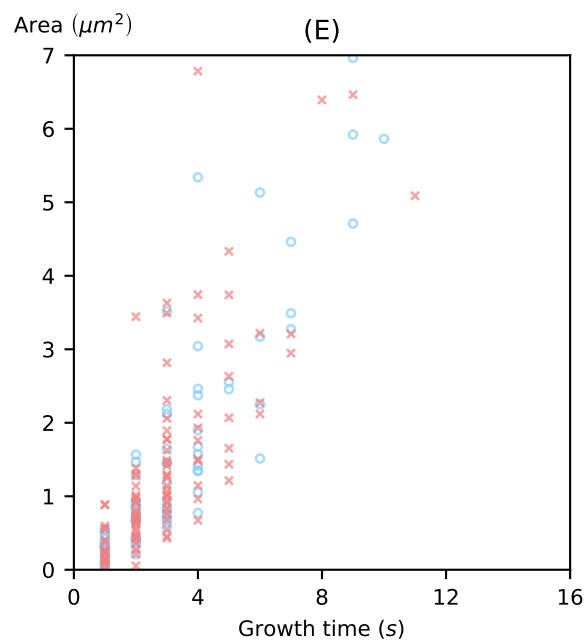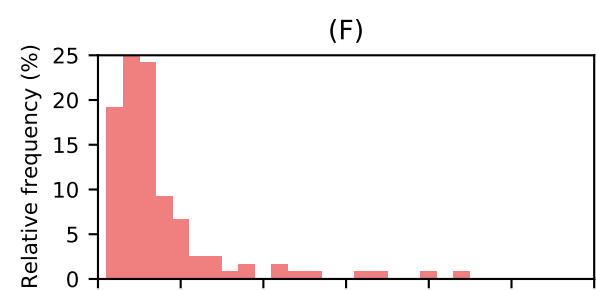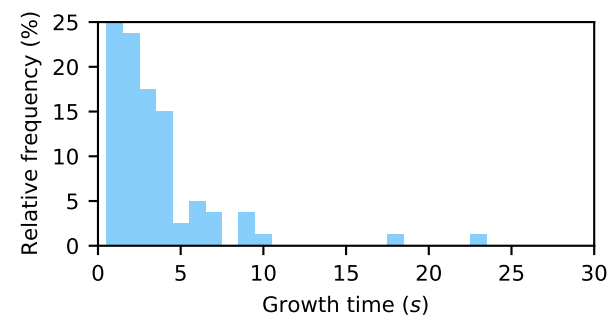

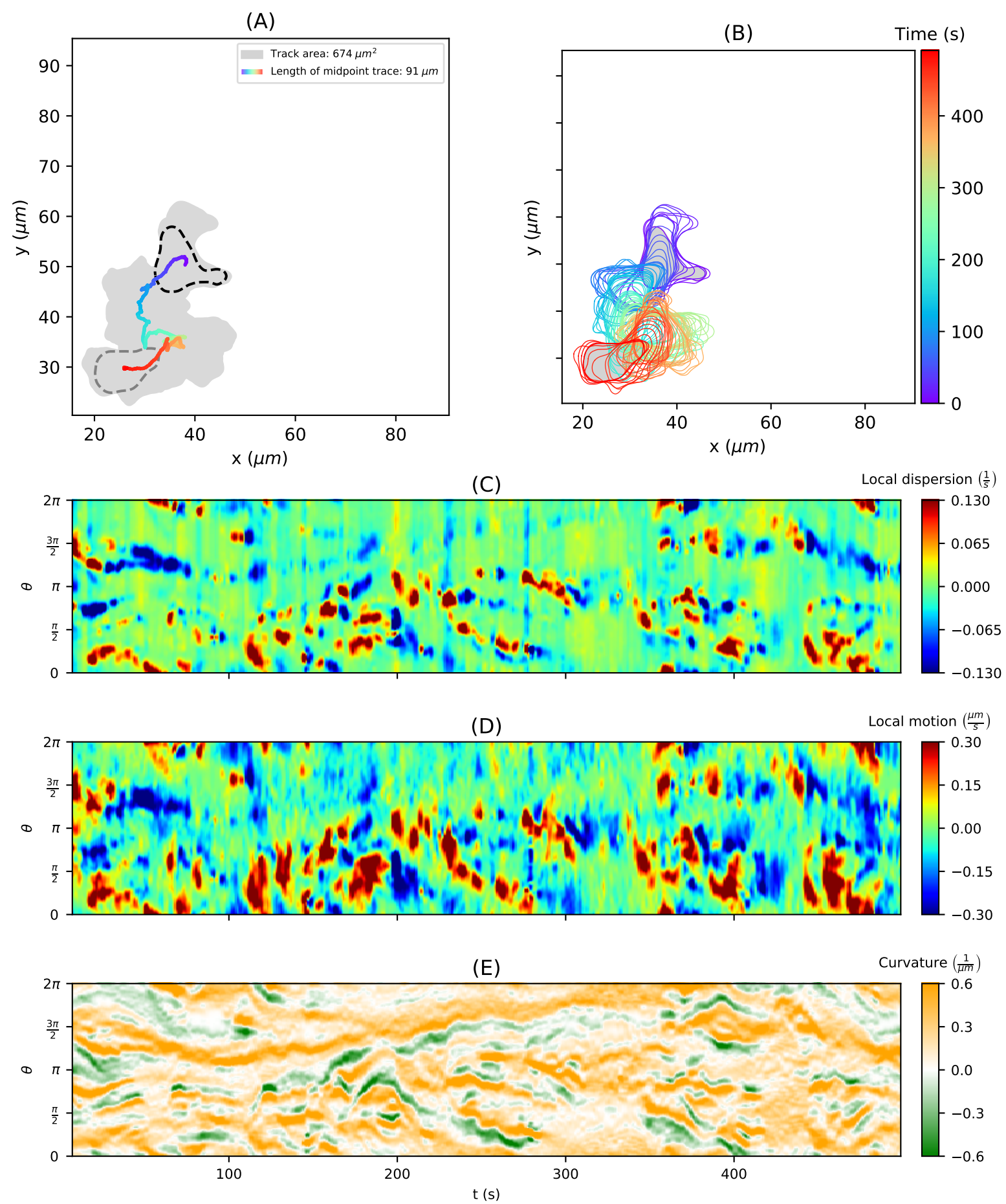

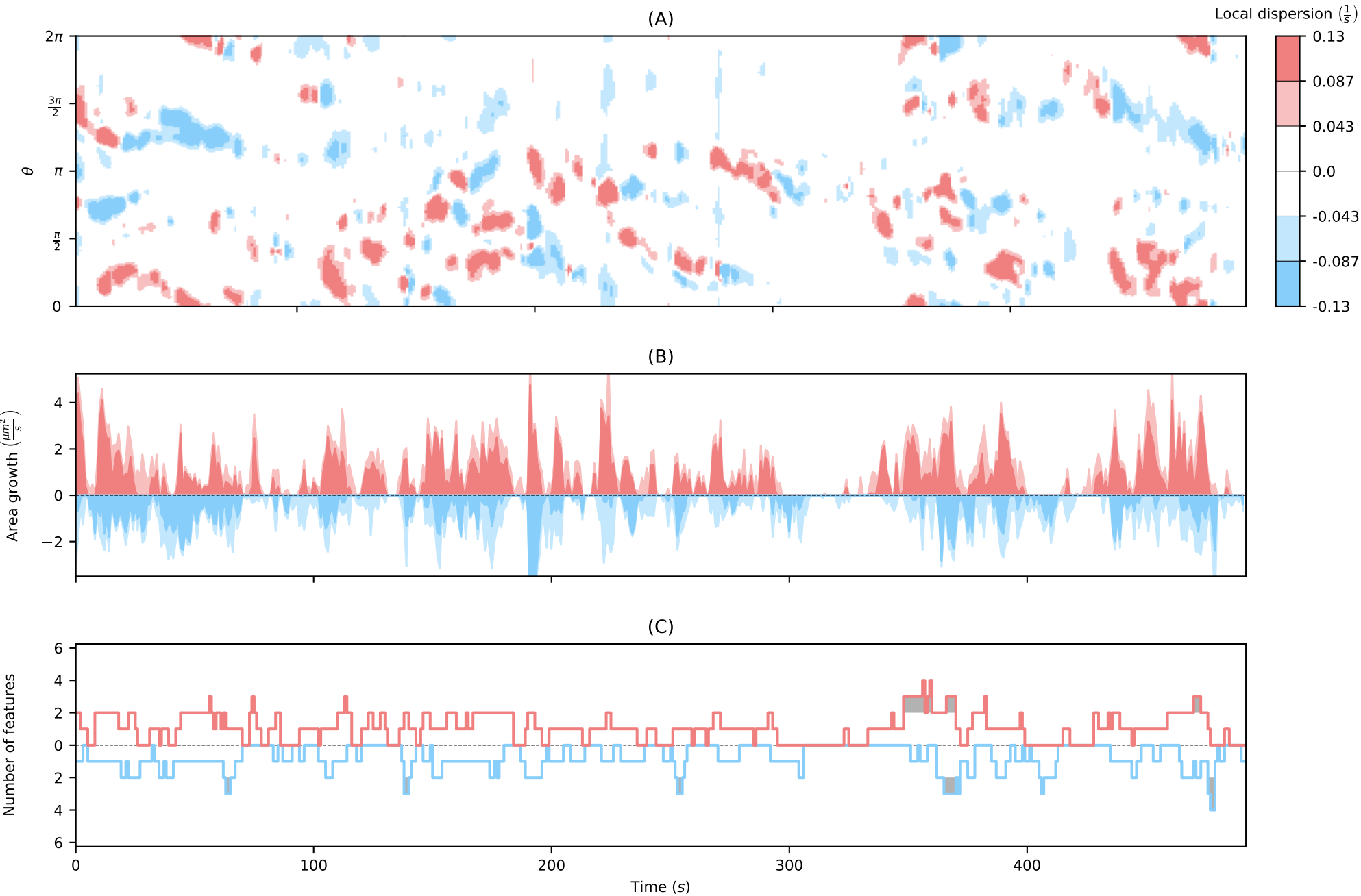

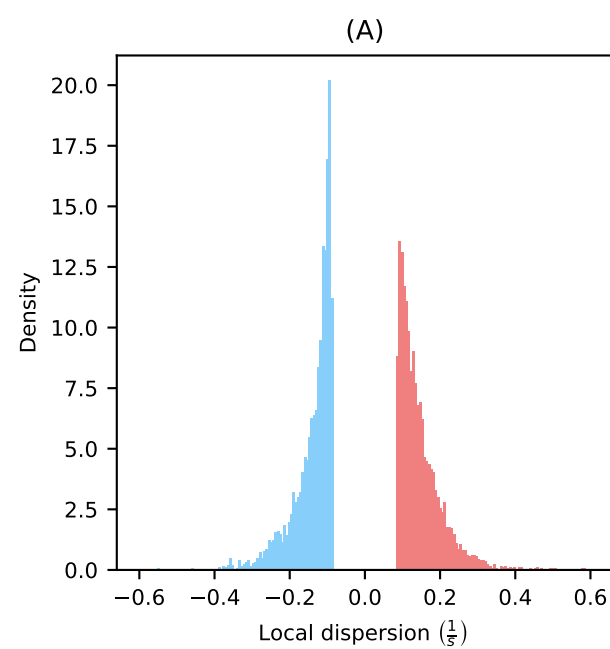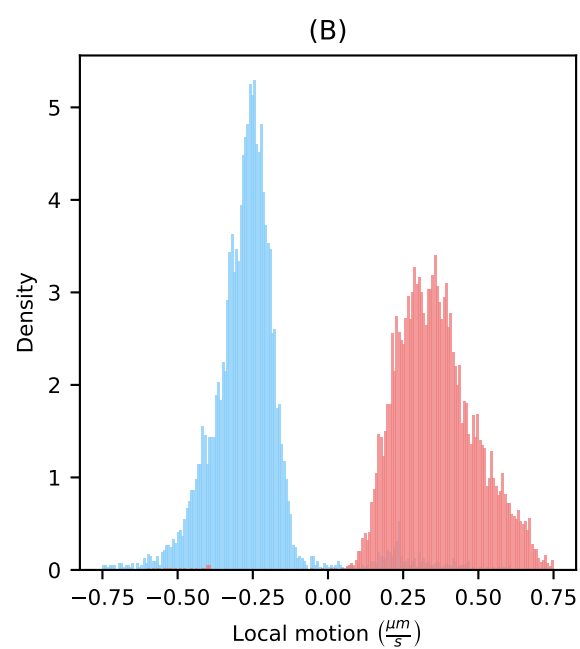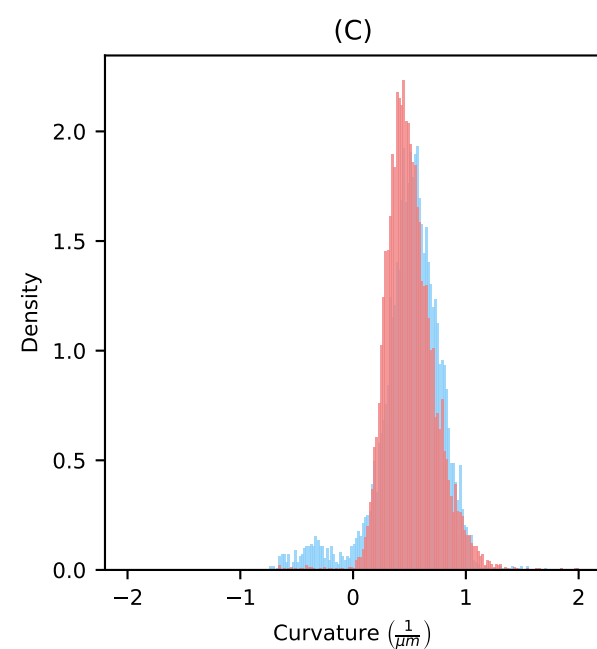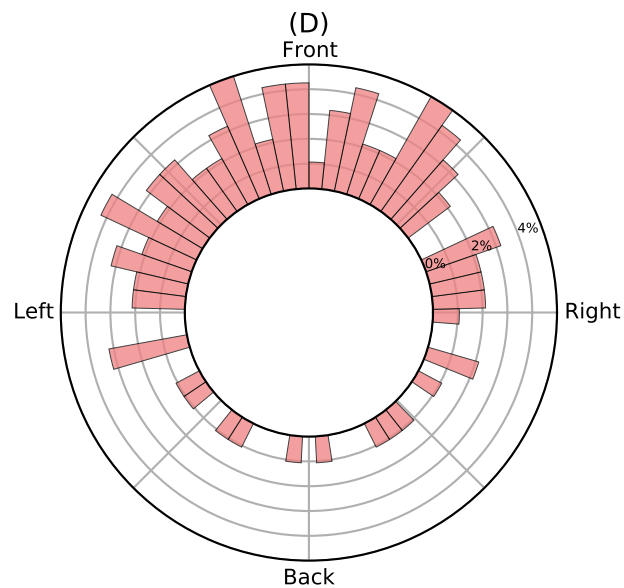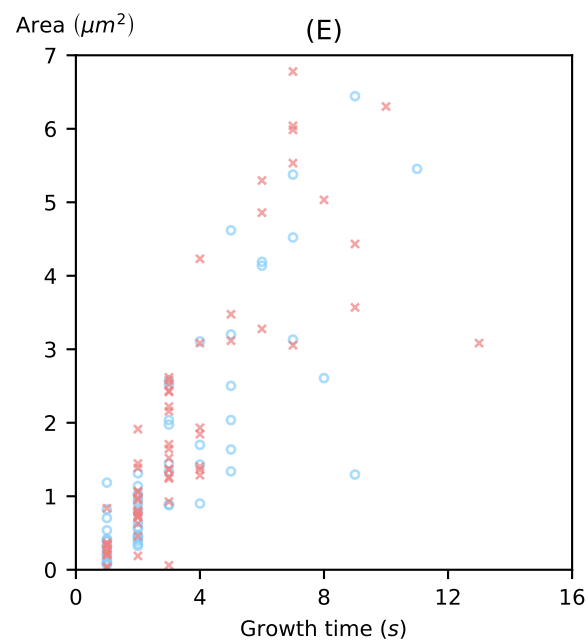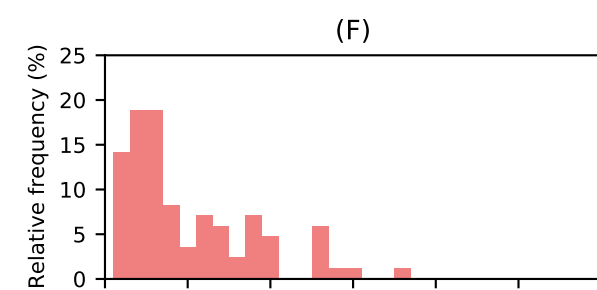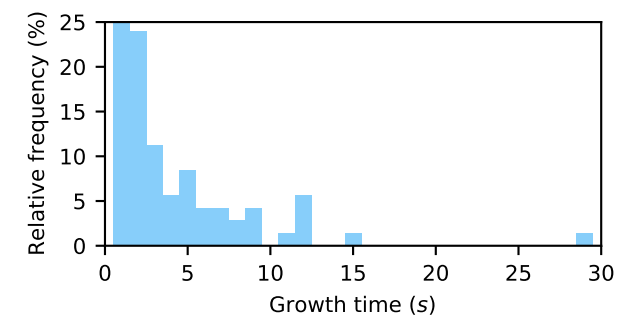

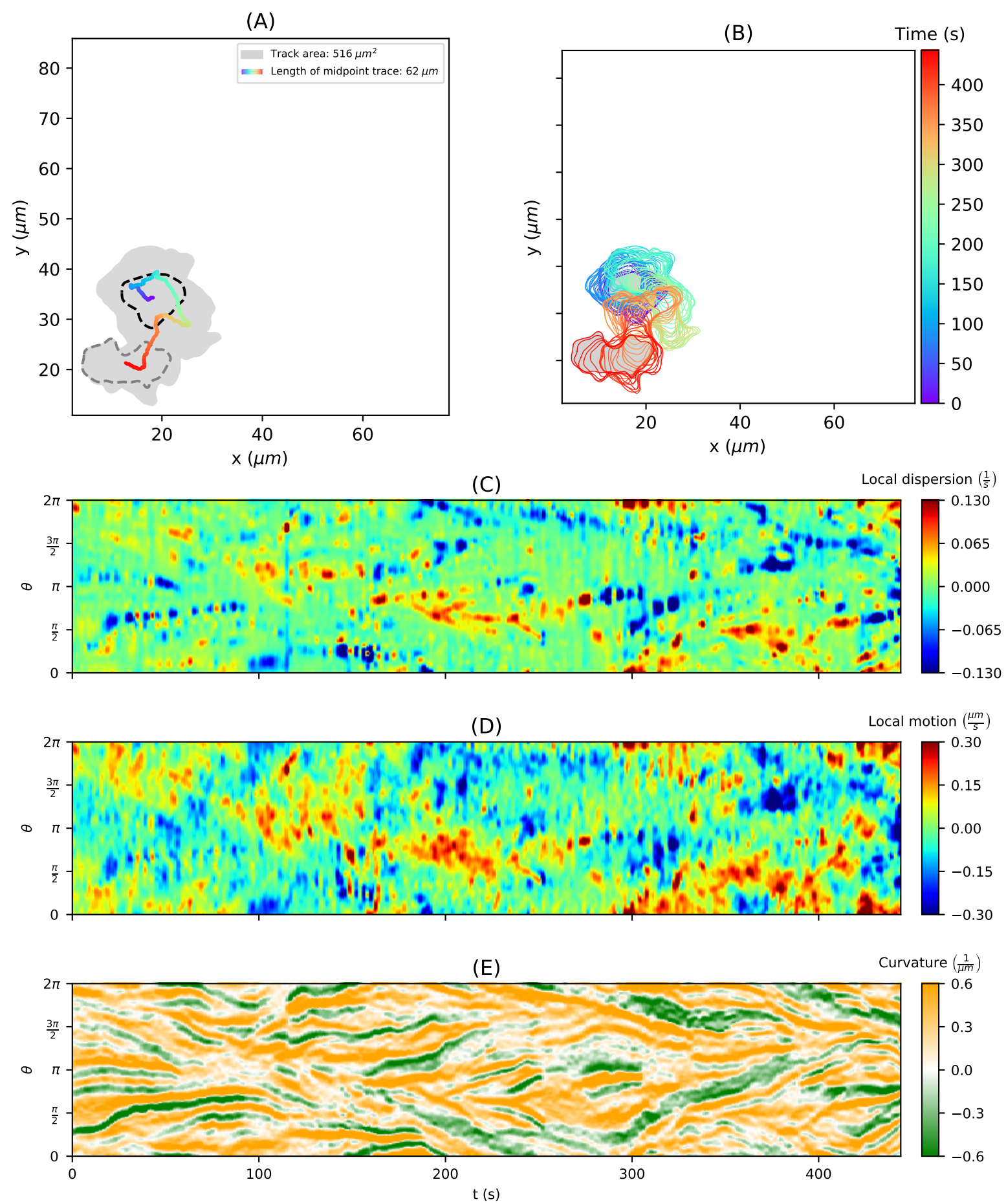

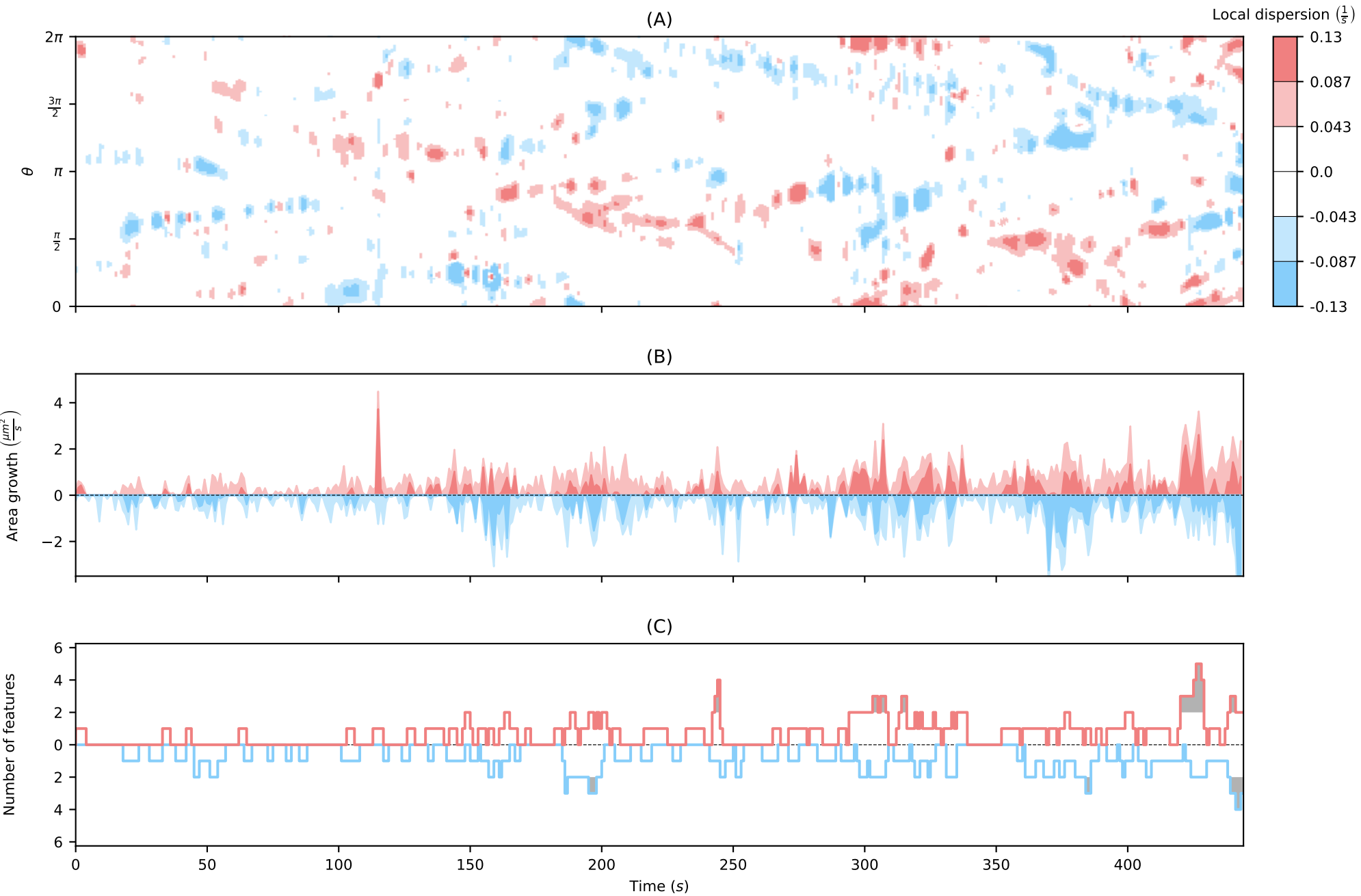

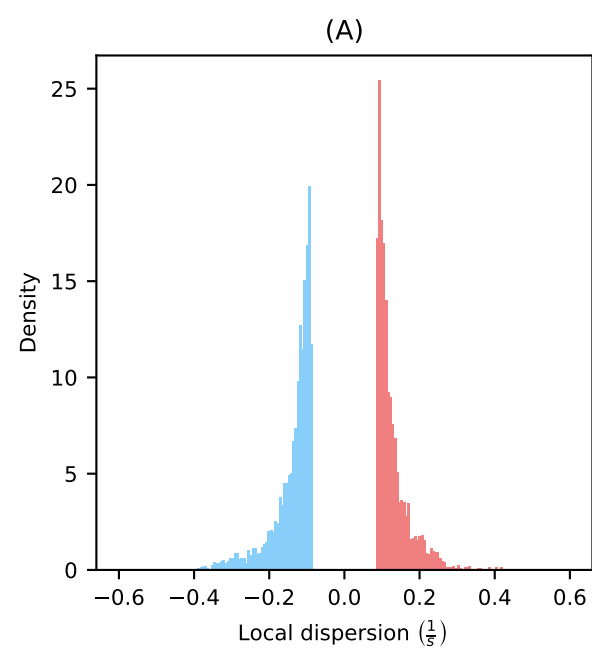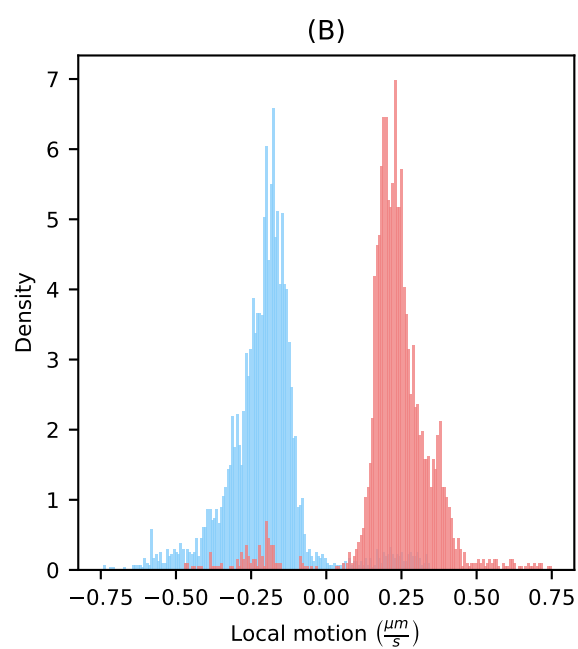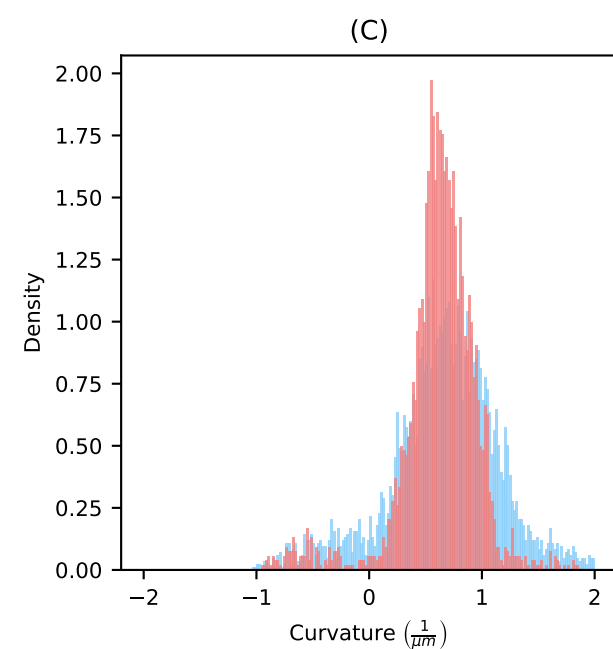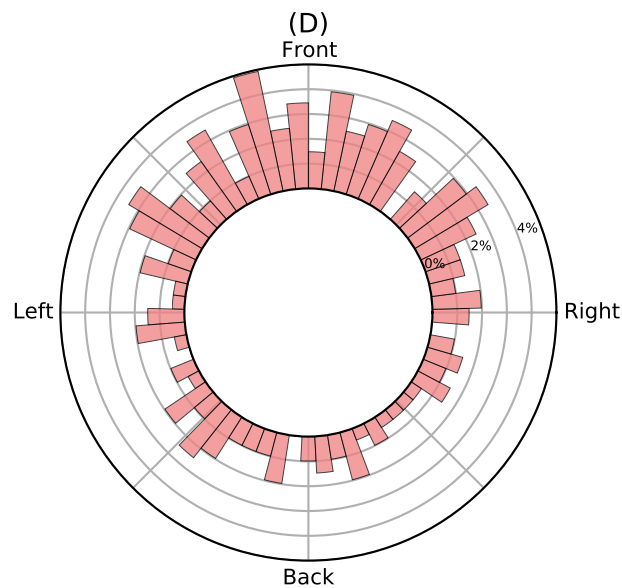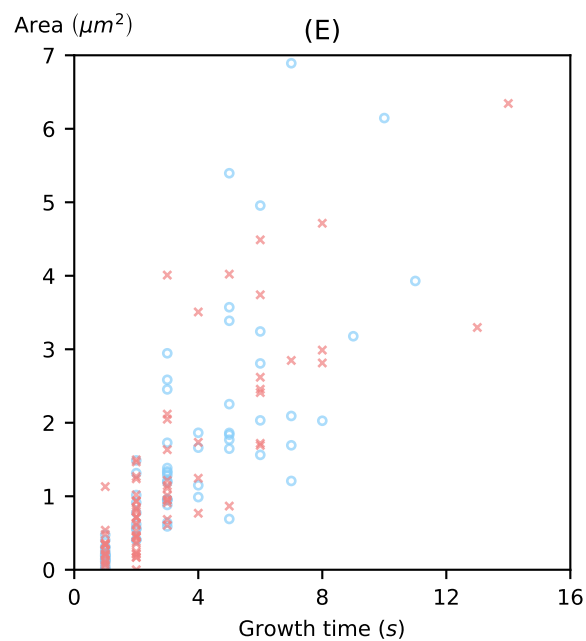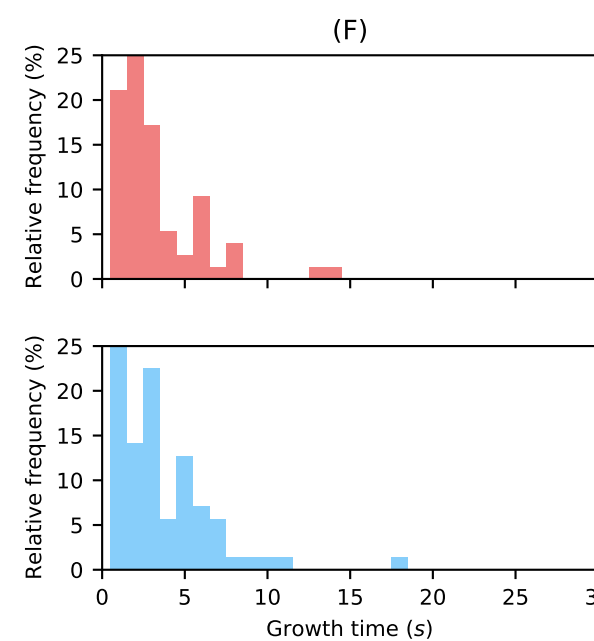

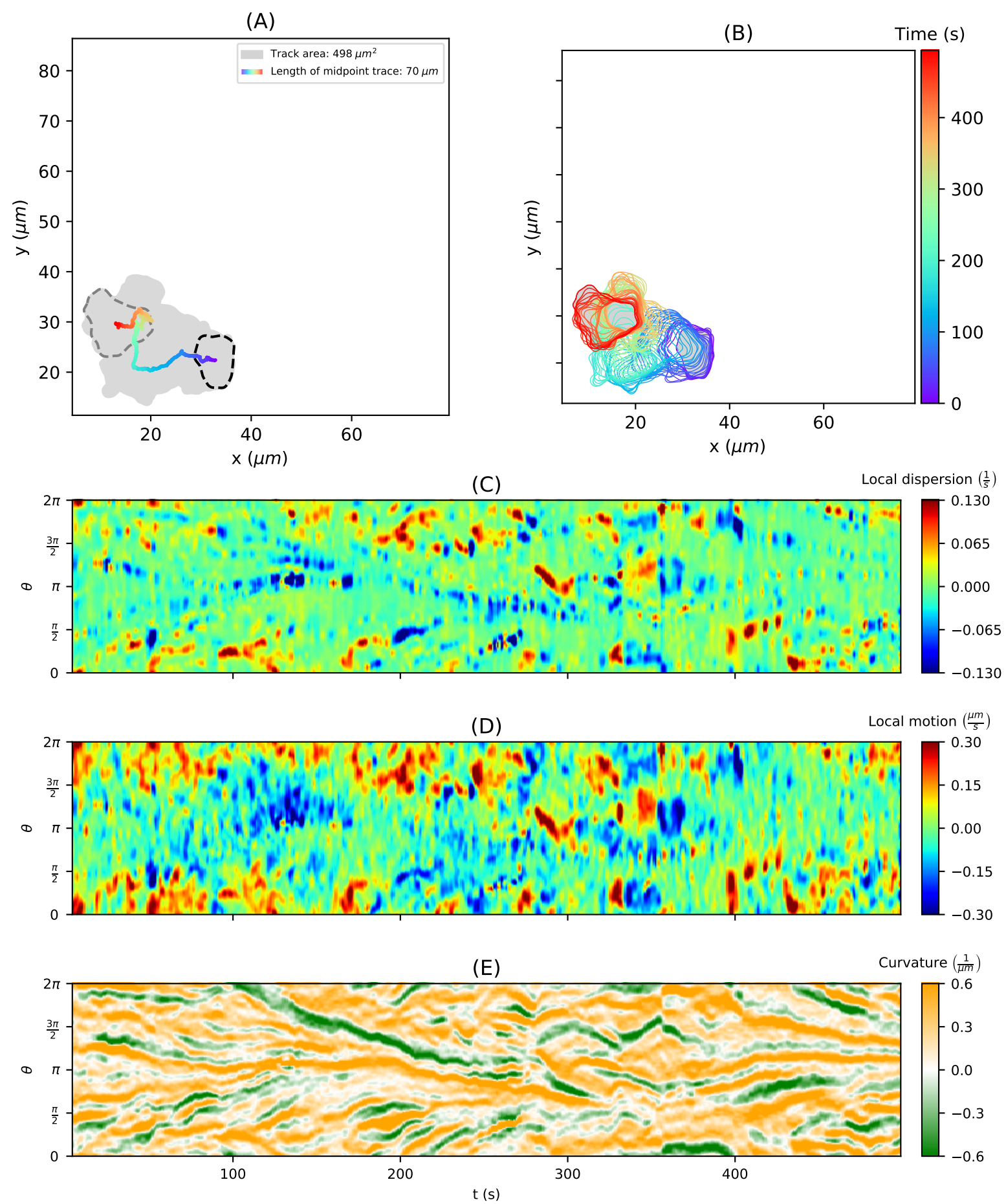

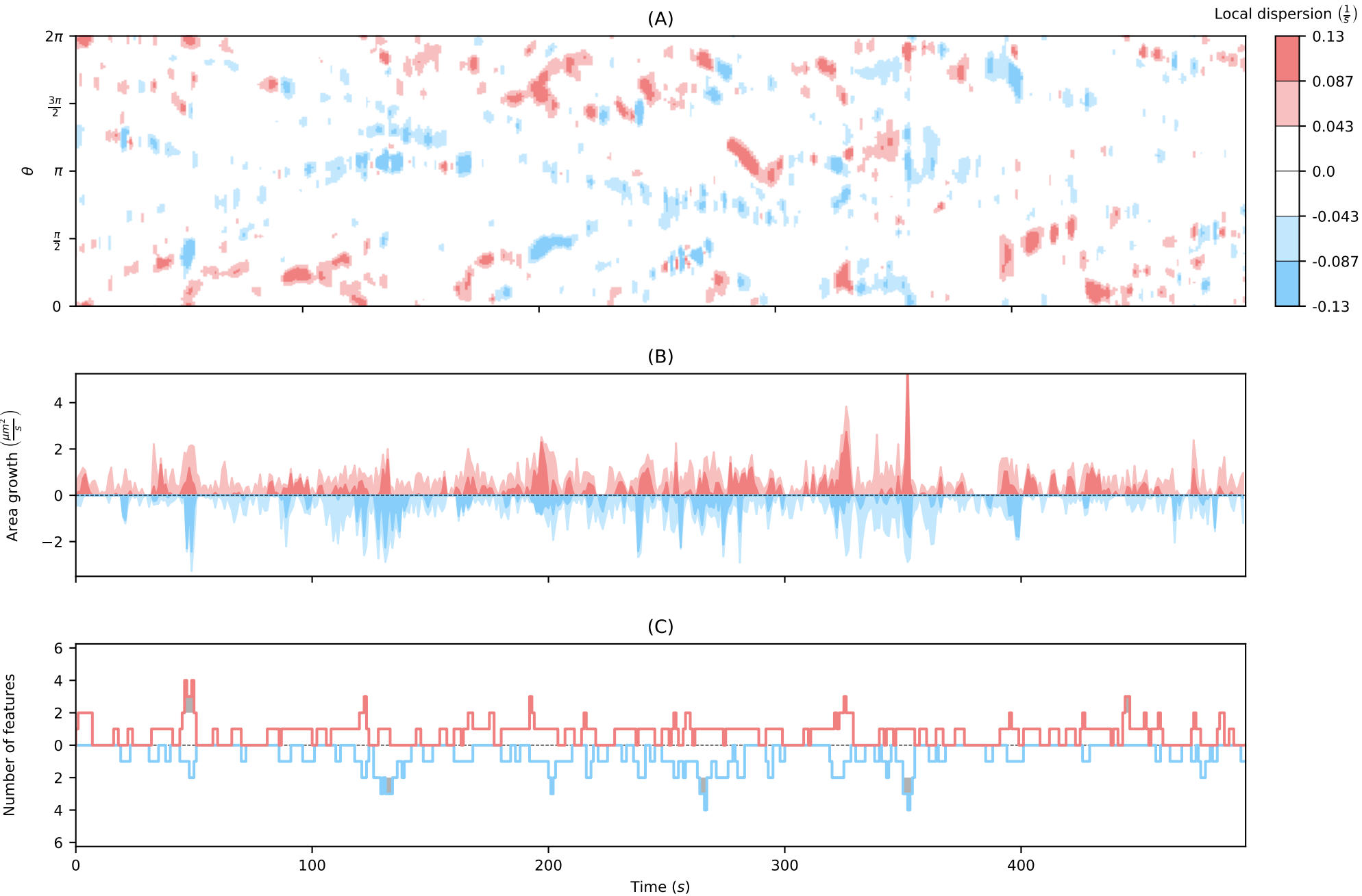

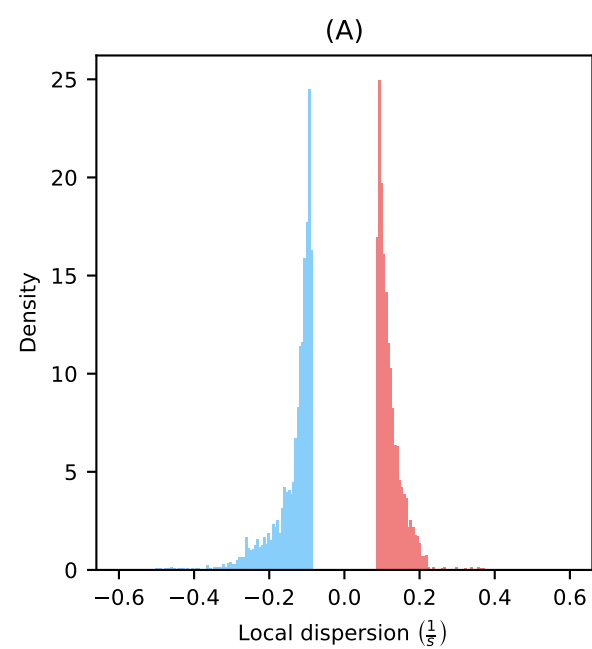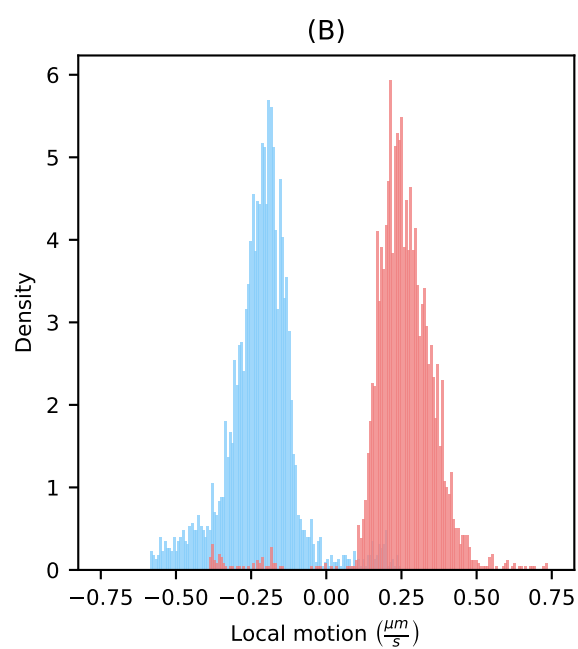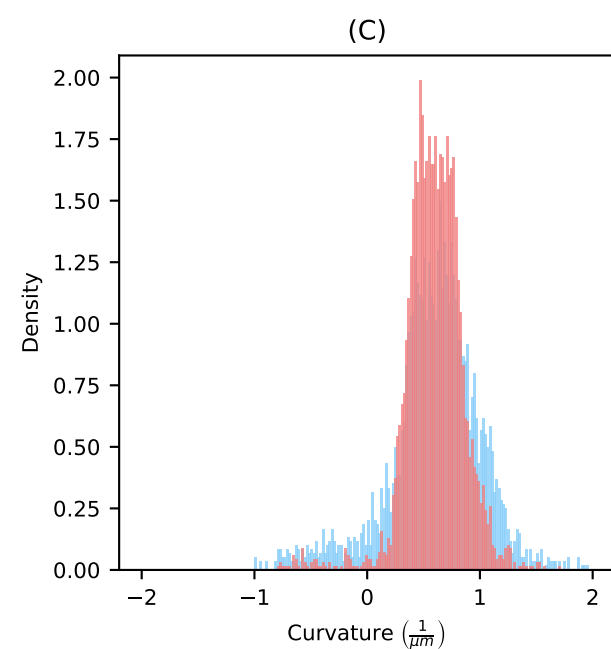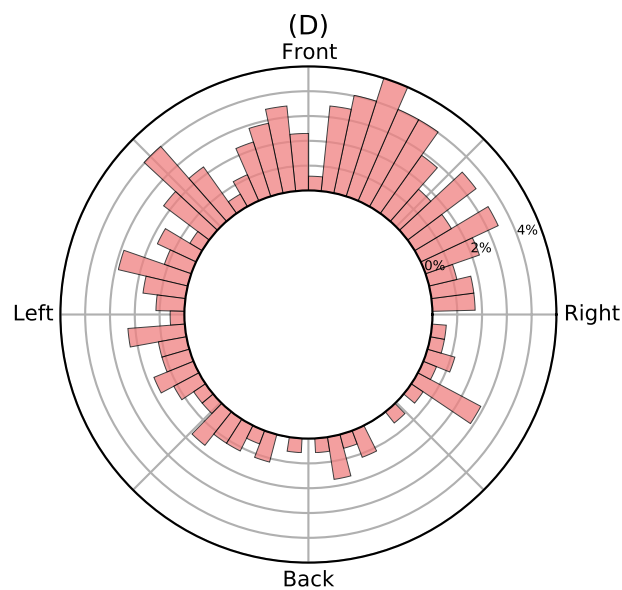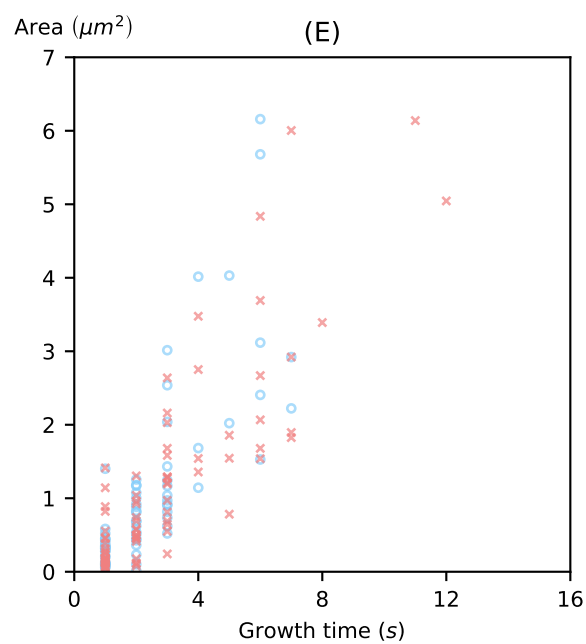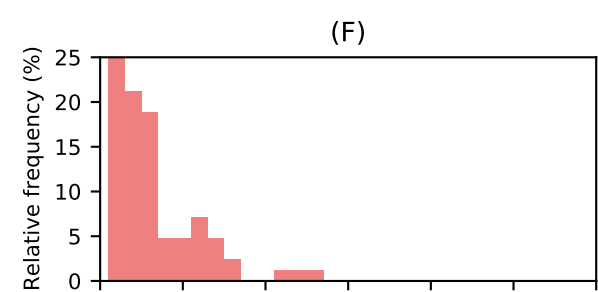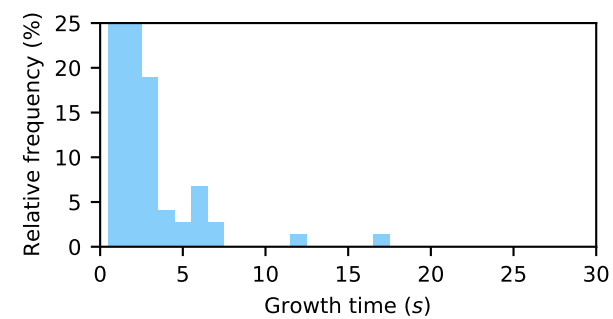

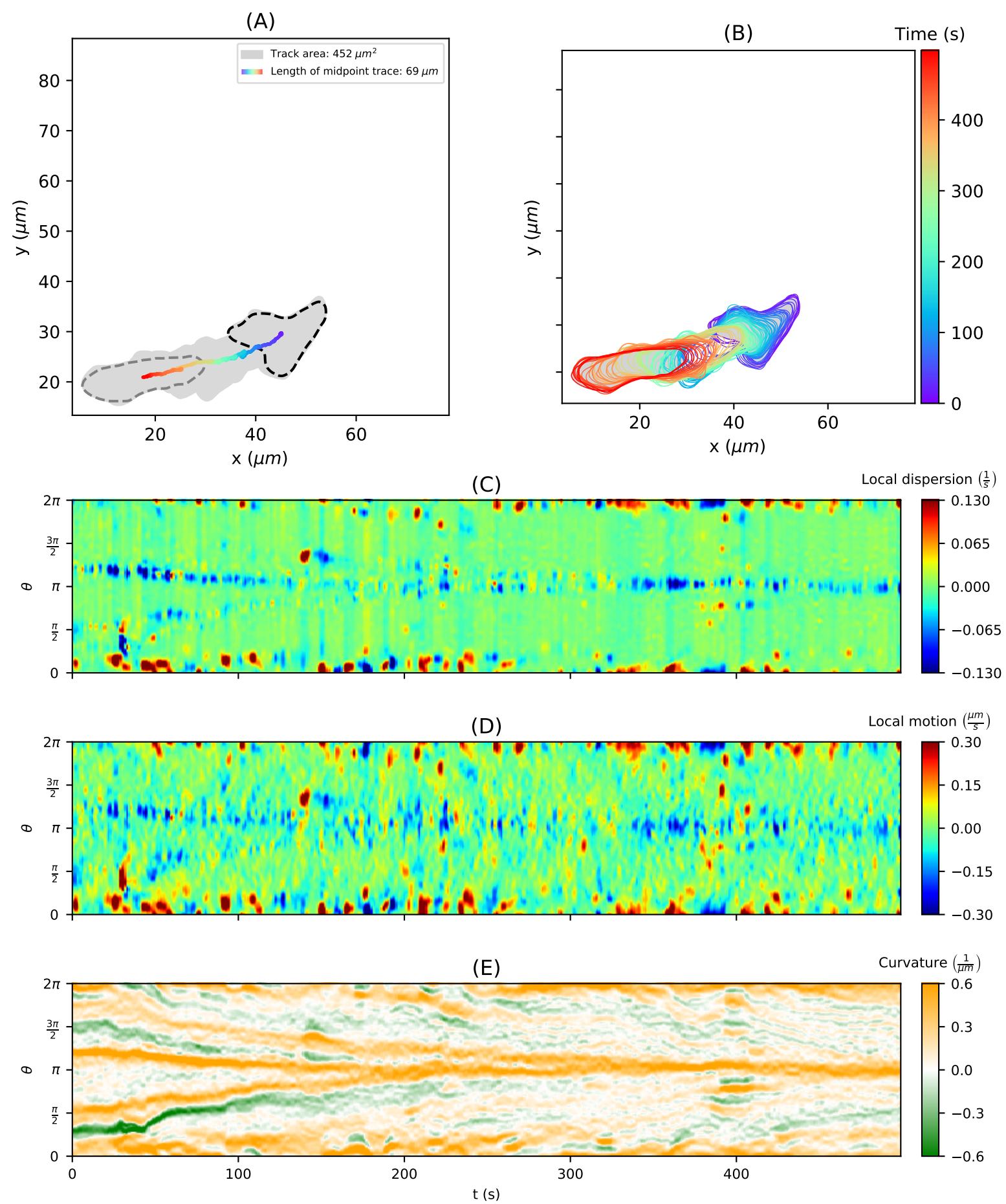

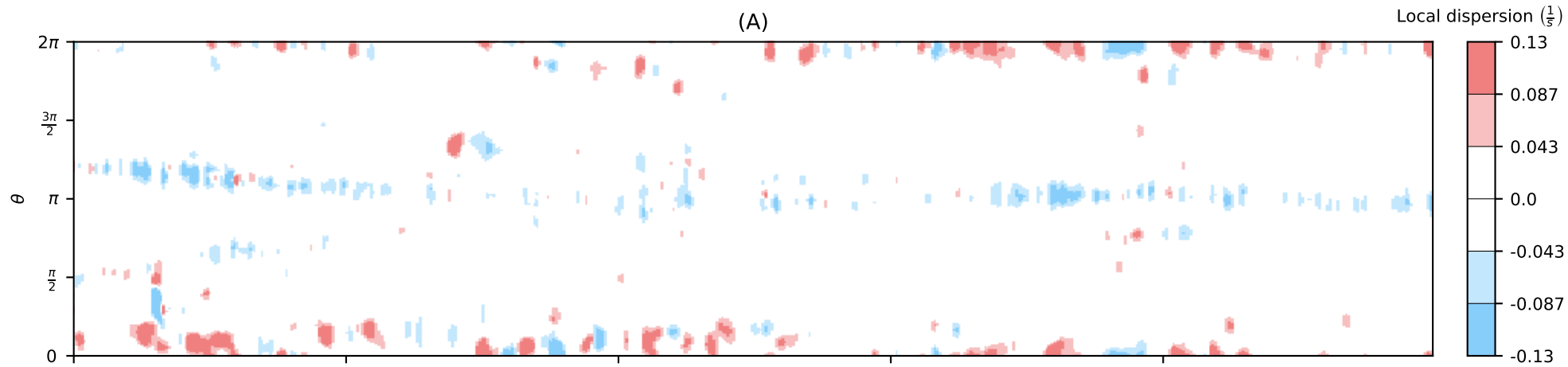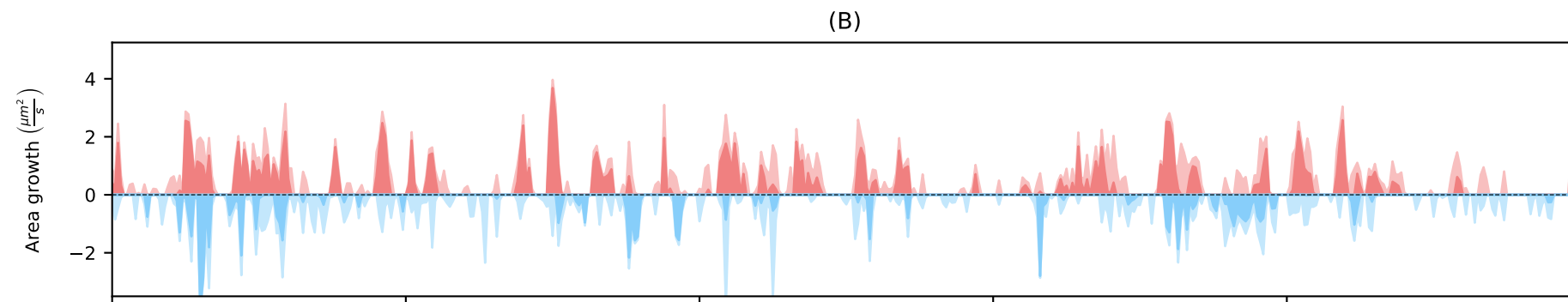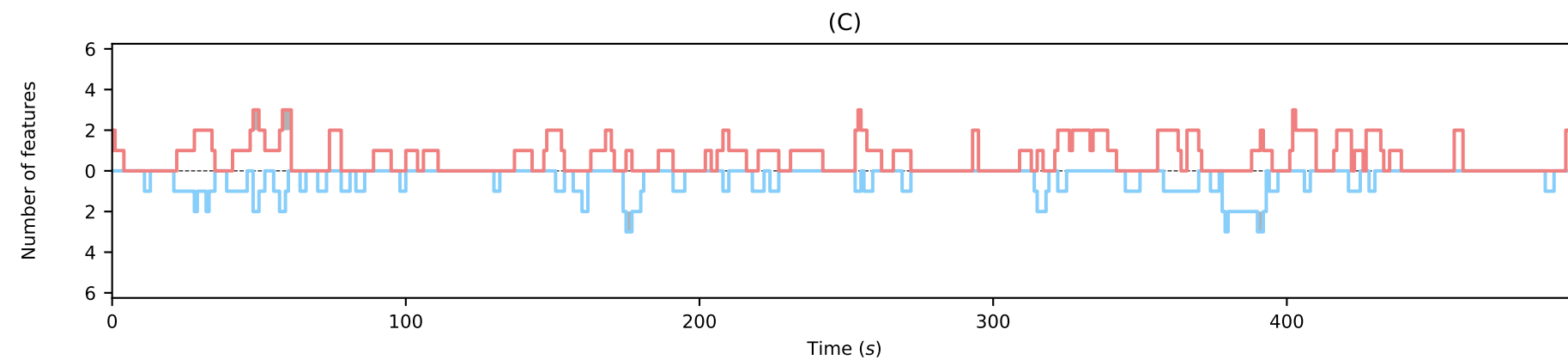

(A)

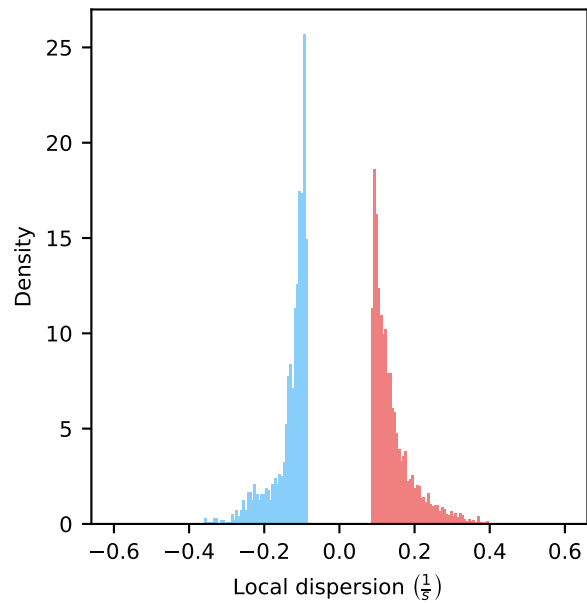

(B)

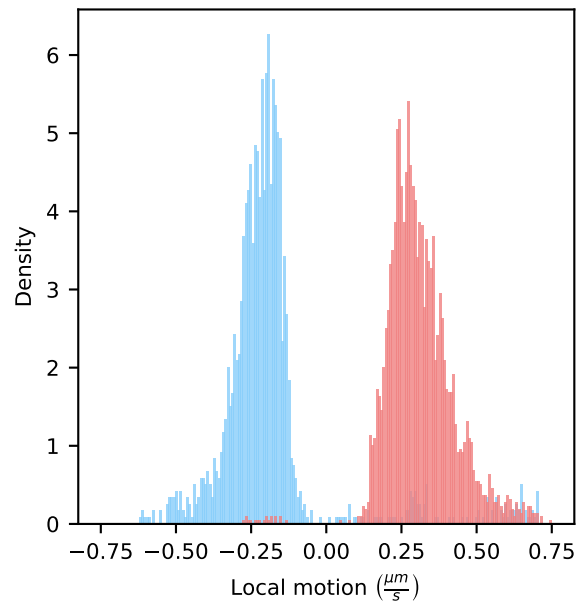

(C)

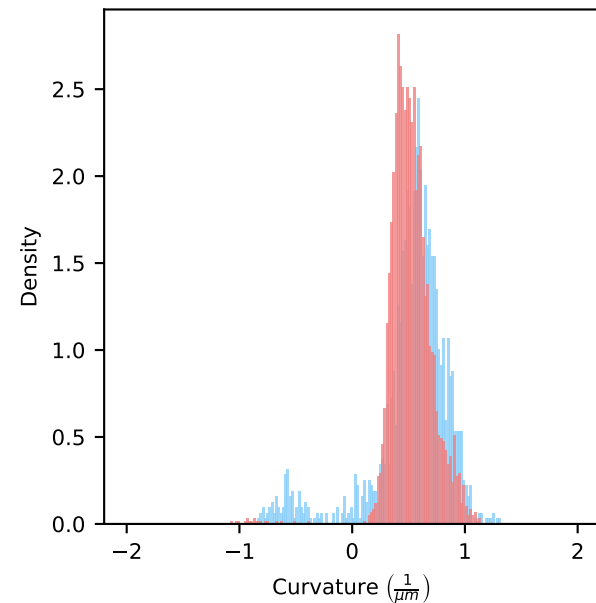

(D)

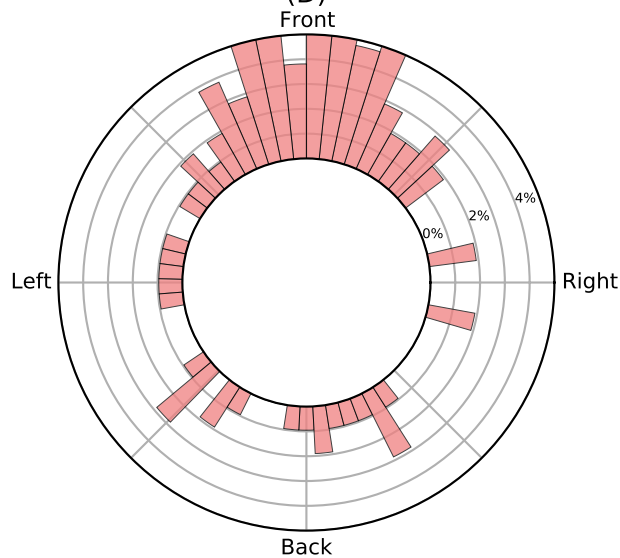

(E)

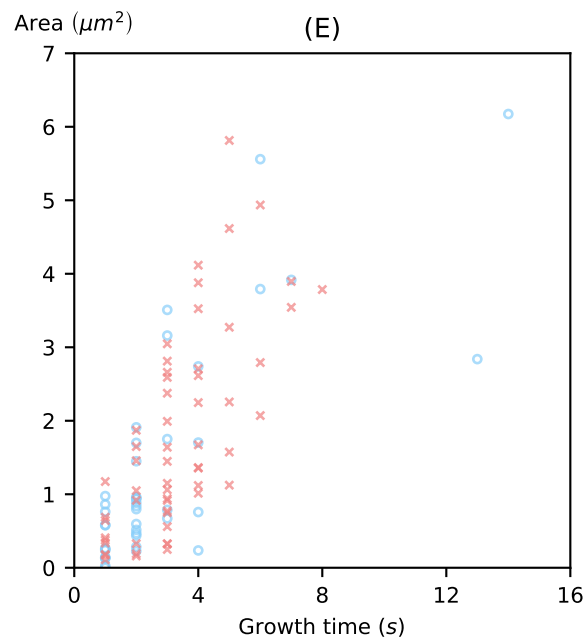

(F)

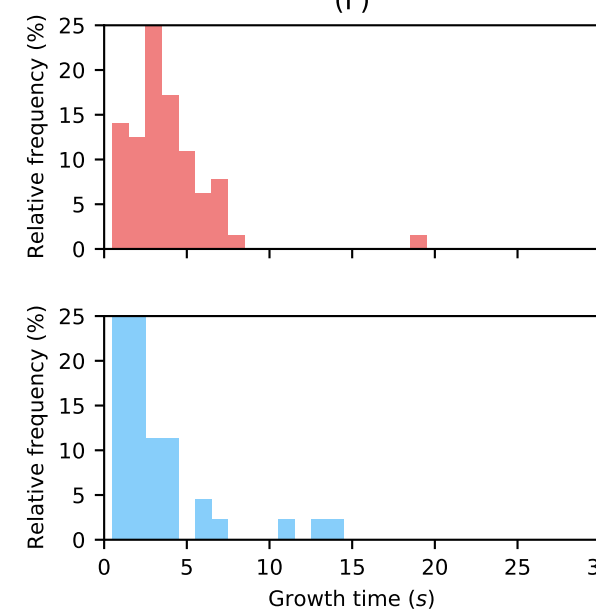

Supplement: S13 Fig — For each track the kymographs of local dispersion, local motion and curvature are shown, followed by plots as in Figs 9 and 10. The cells are sorted in descending order regarding the area of the entire contour track. (PDF) [file pcbi.1009268.s014.pdf]

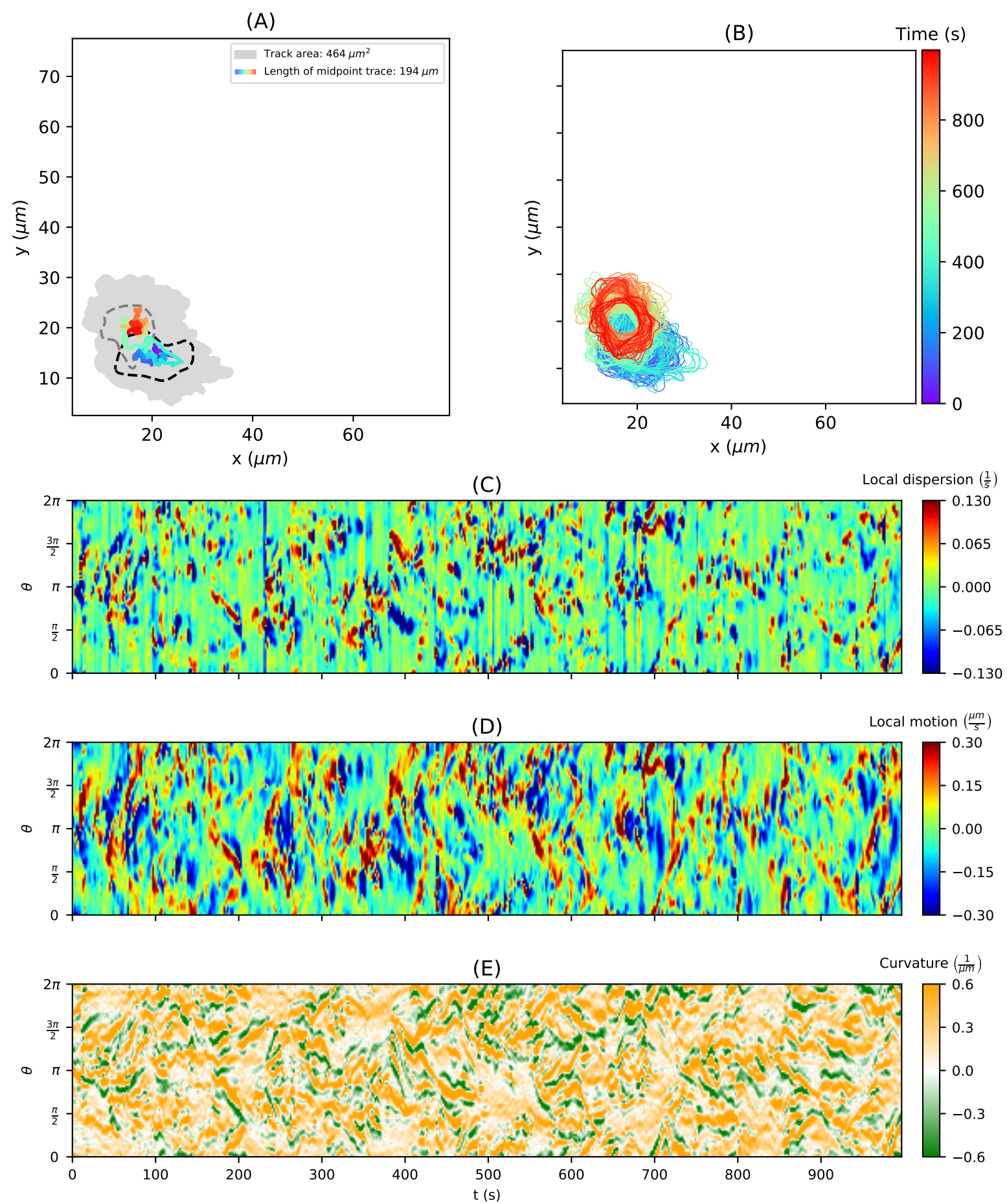

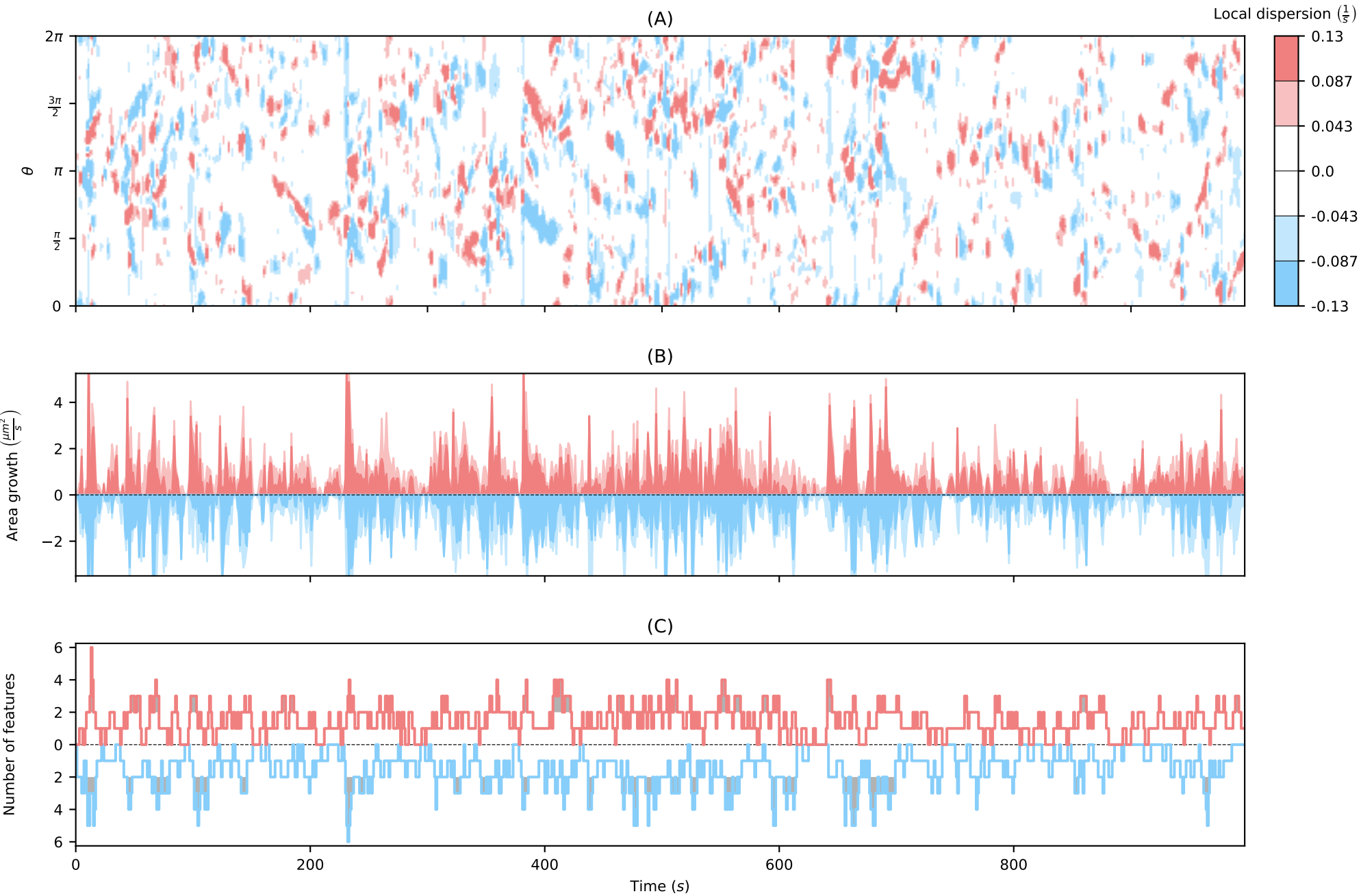

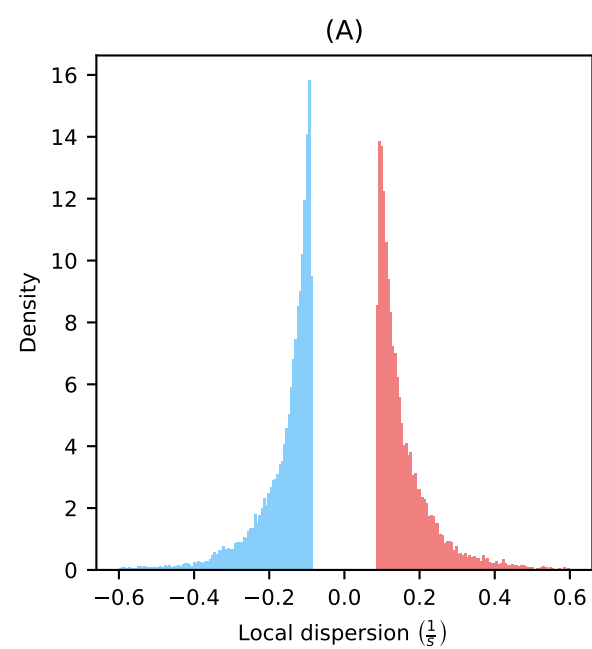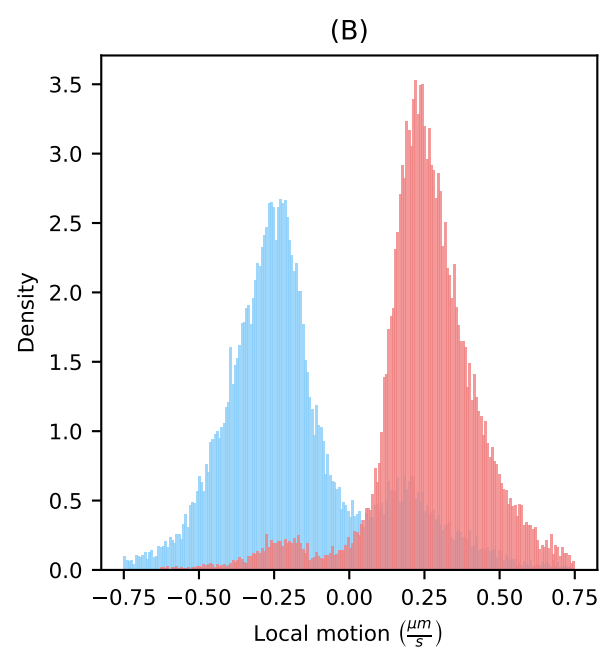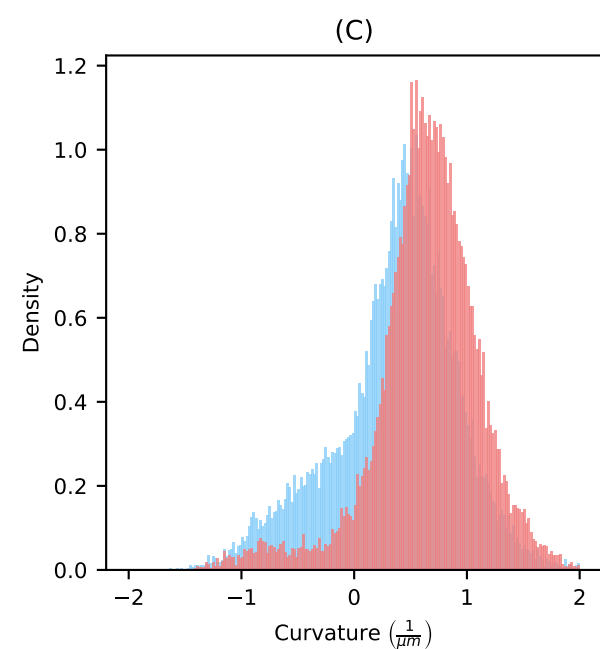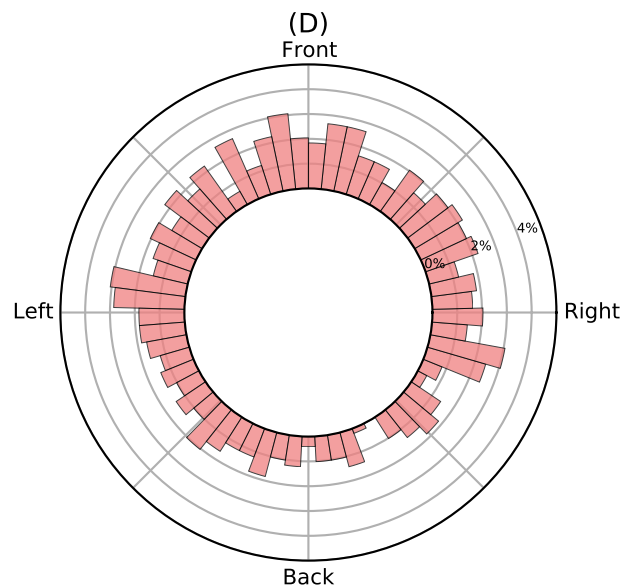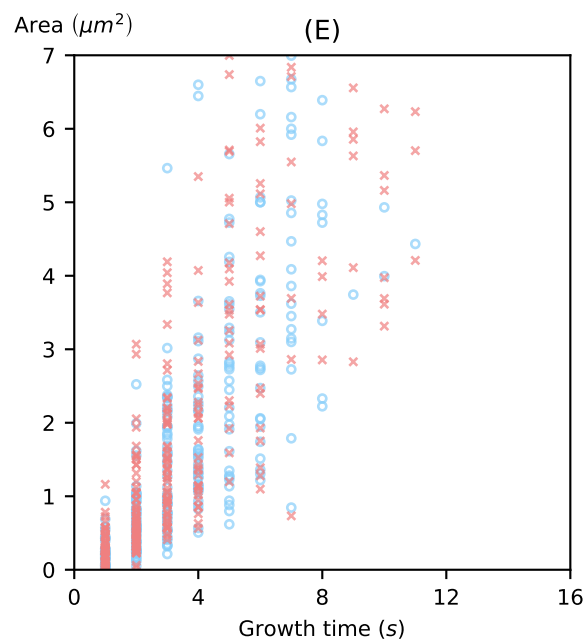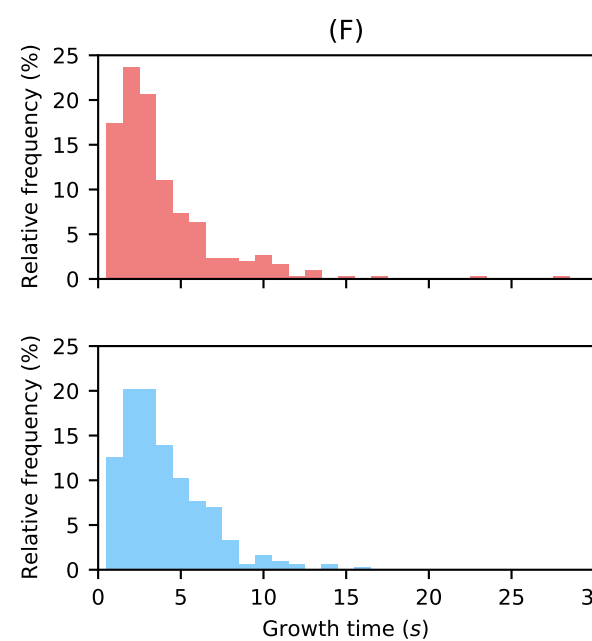

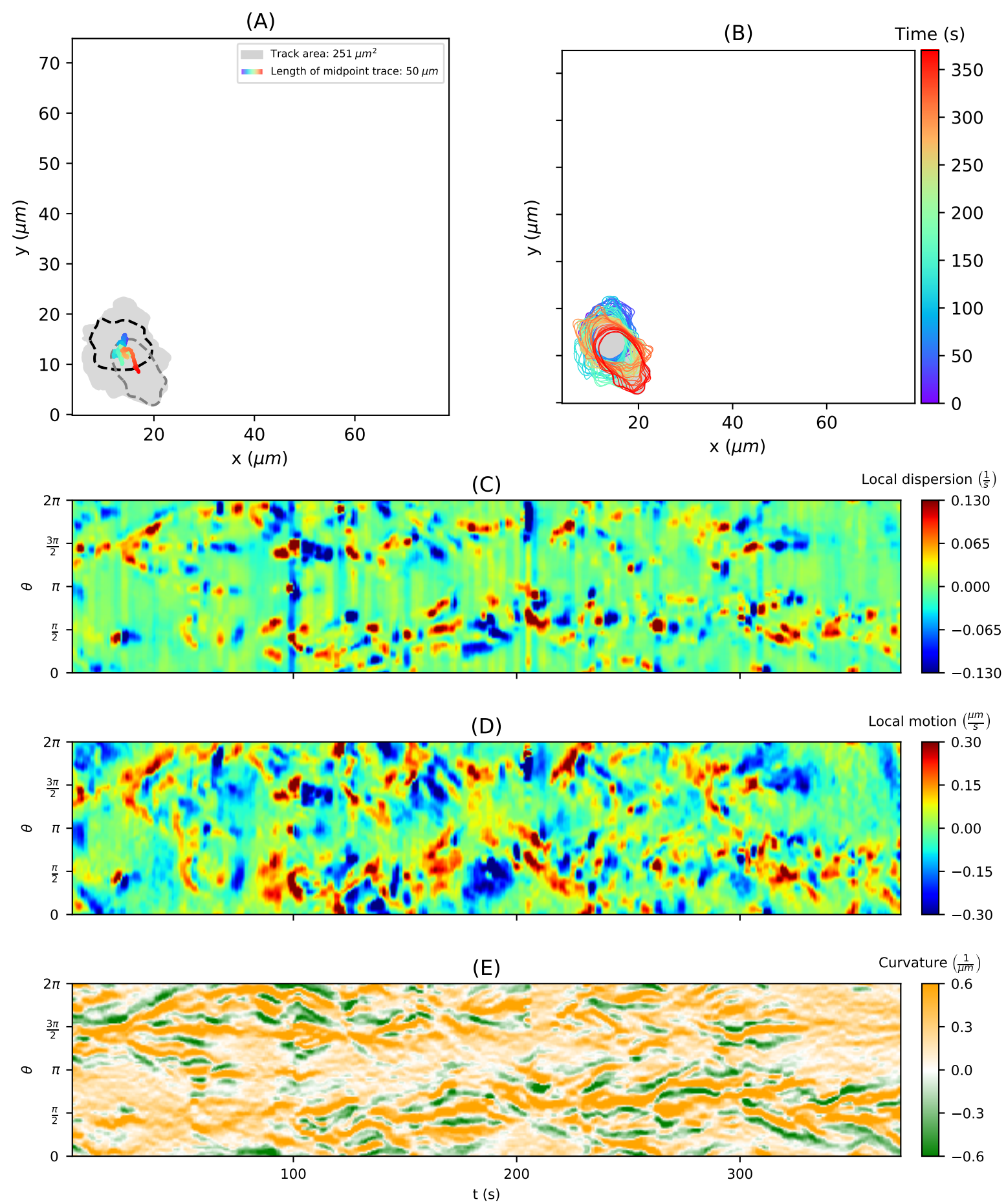

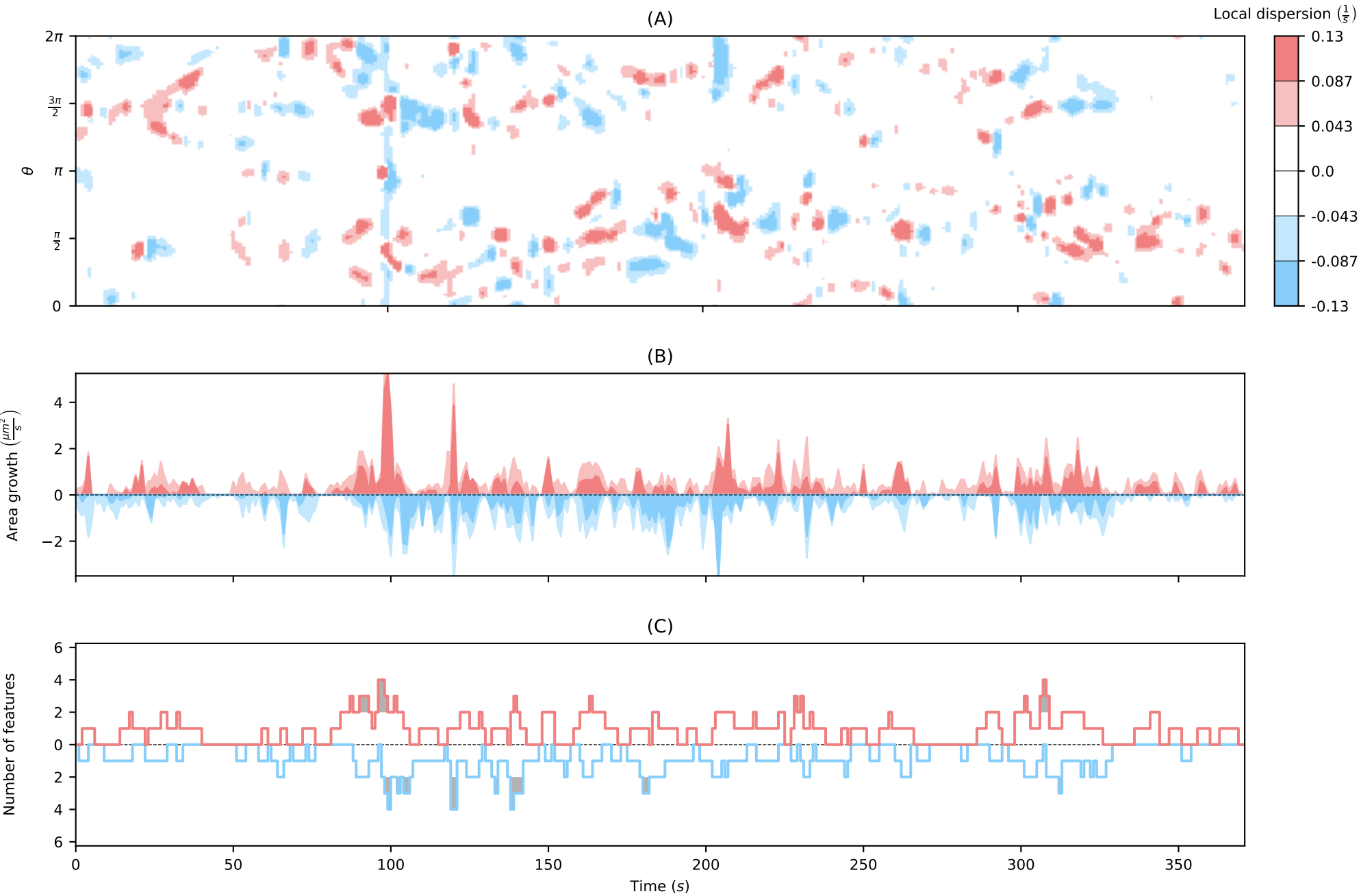

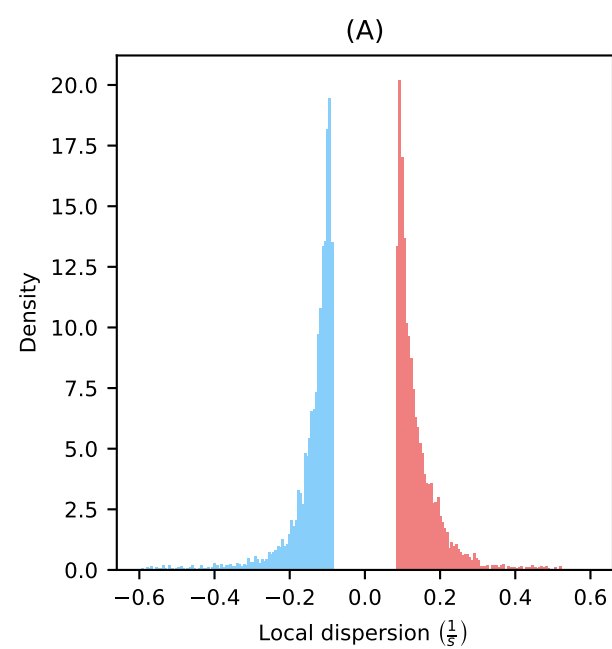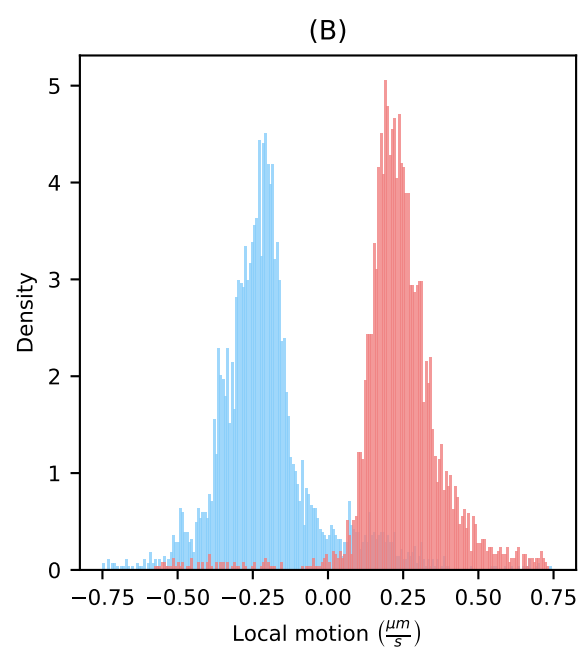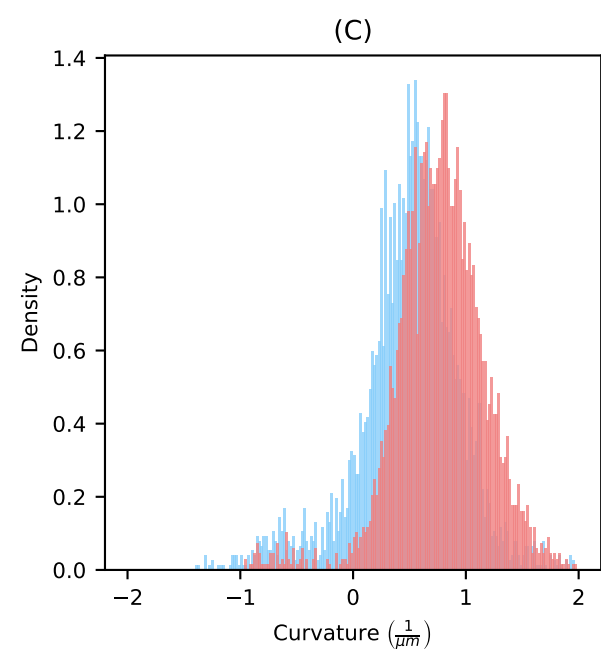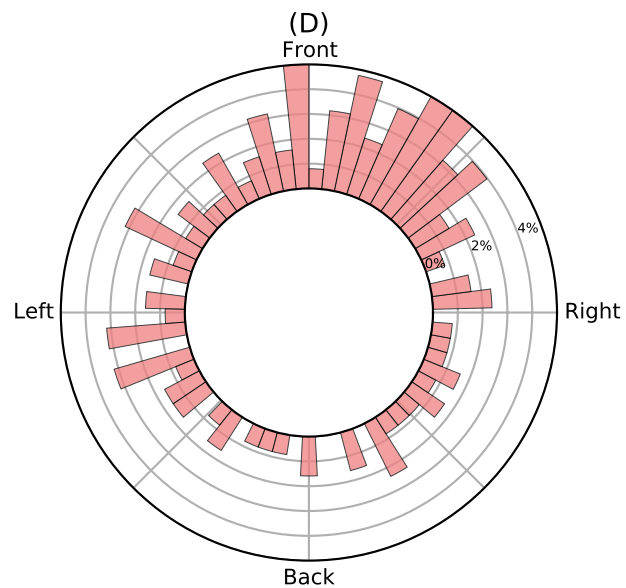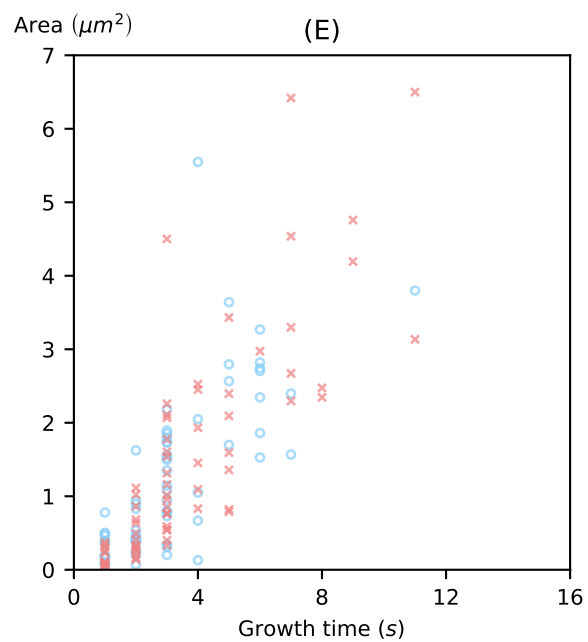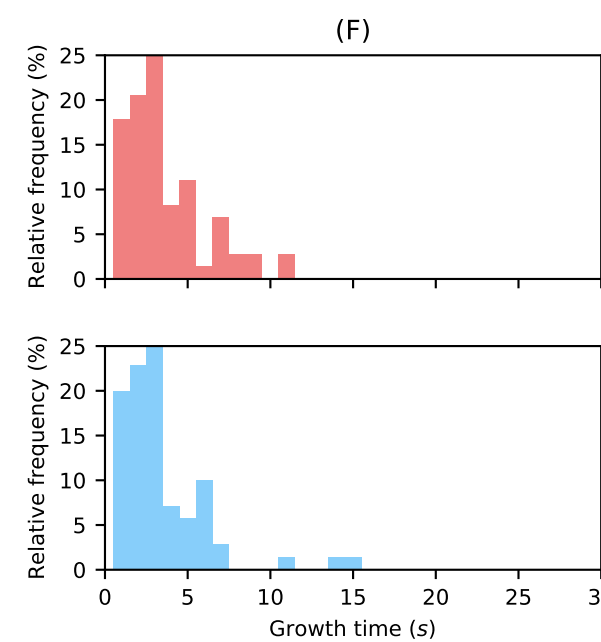

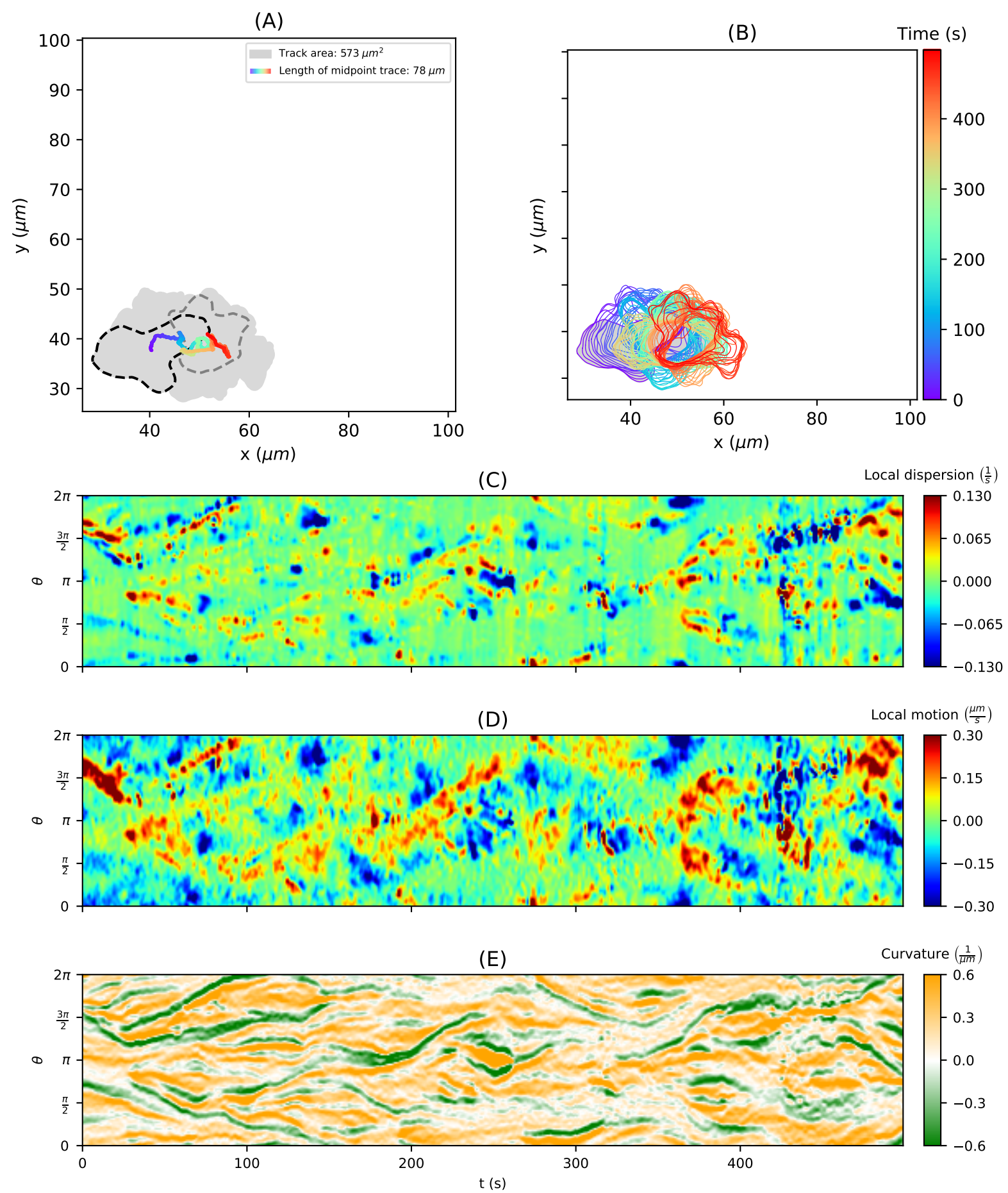

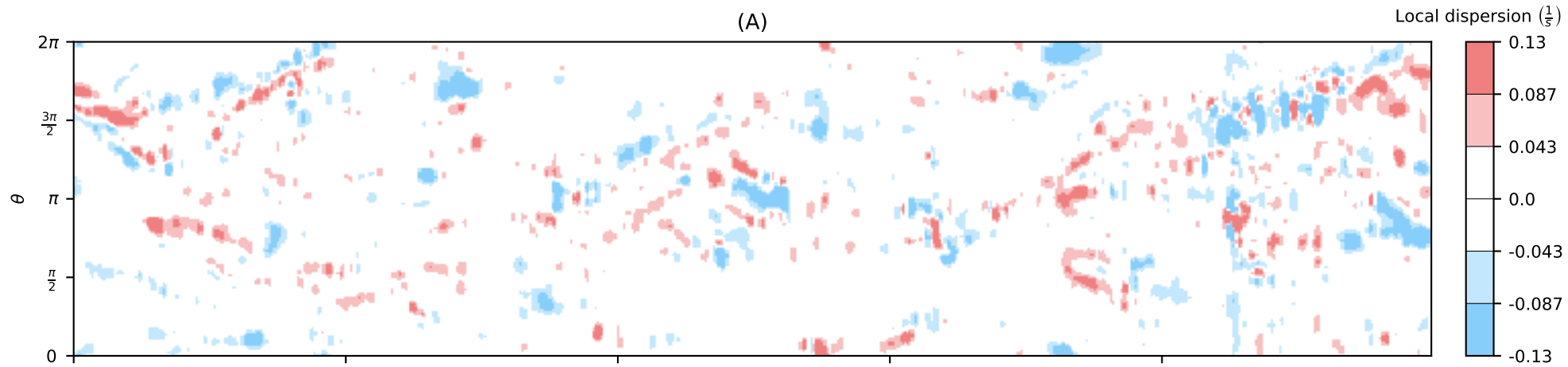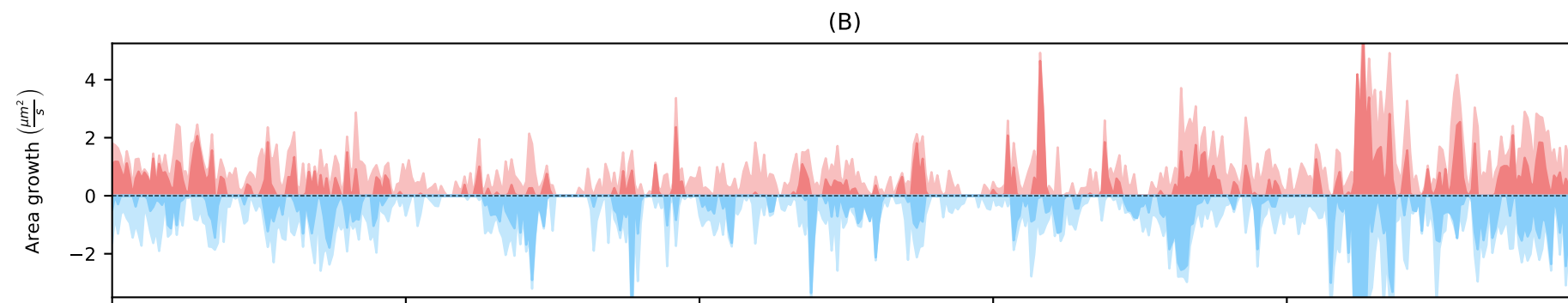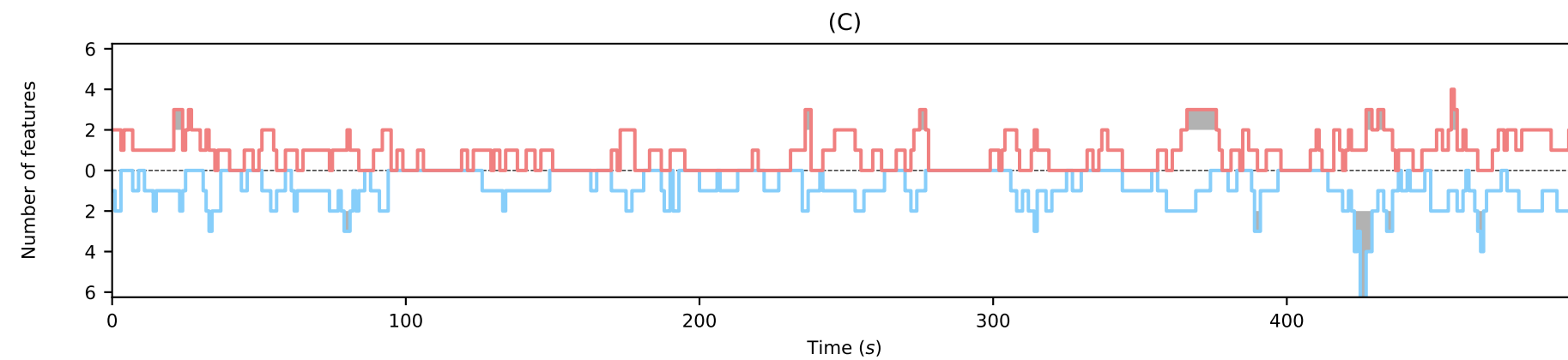

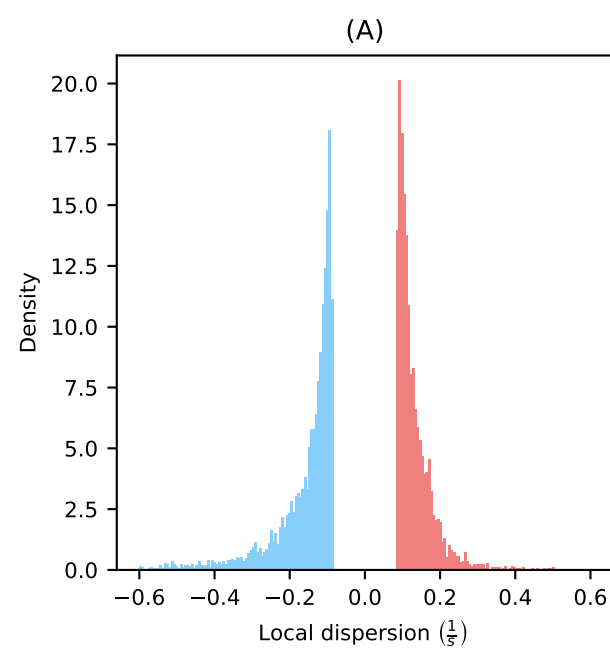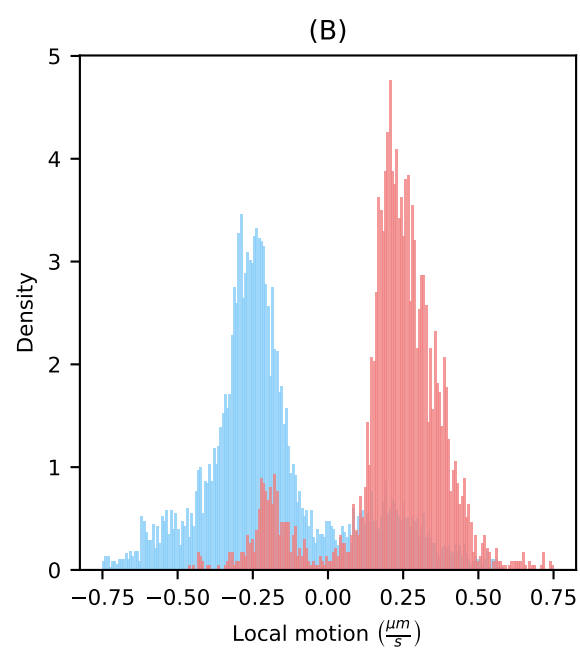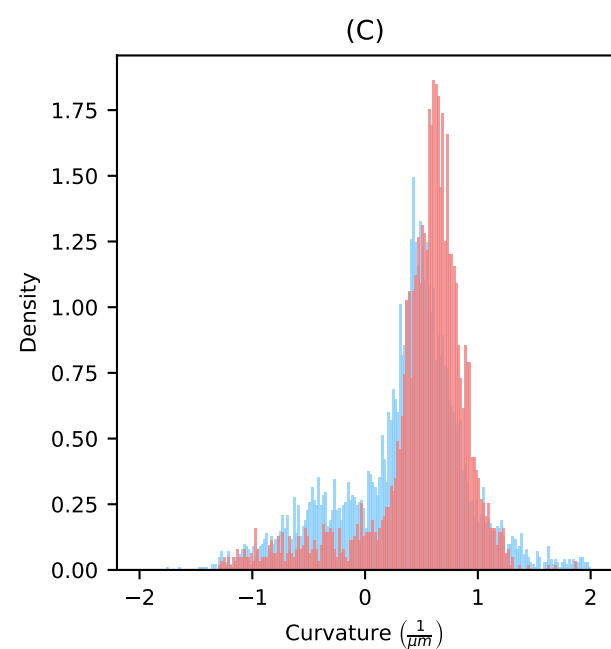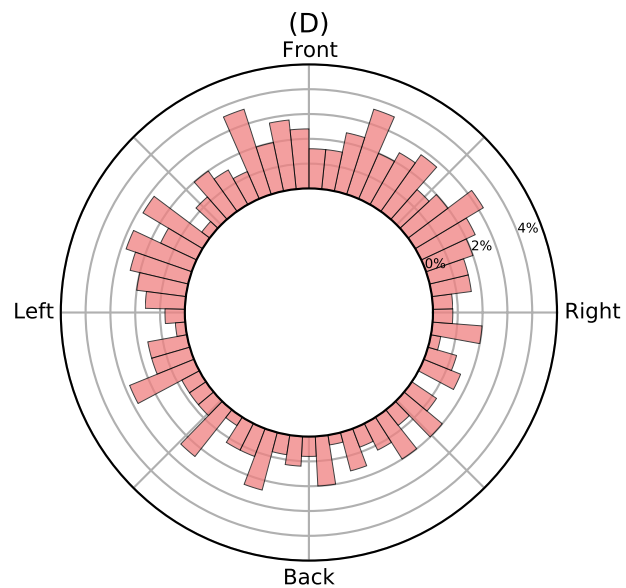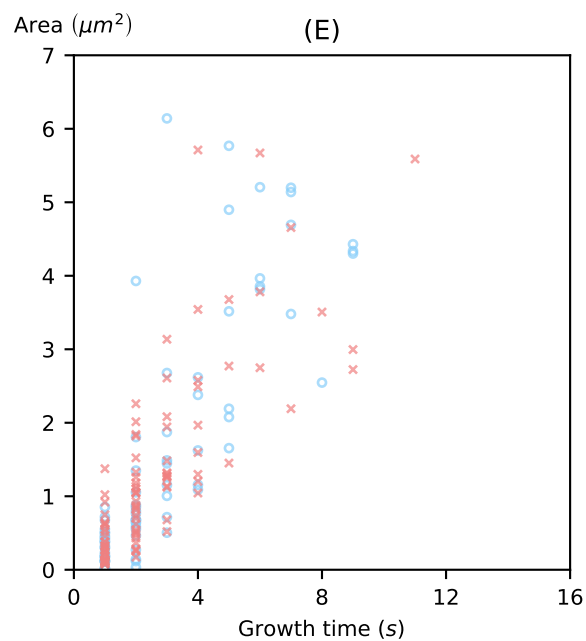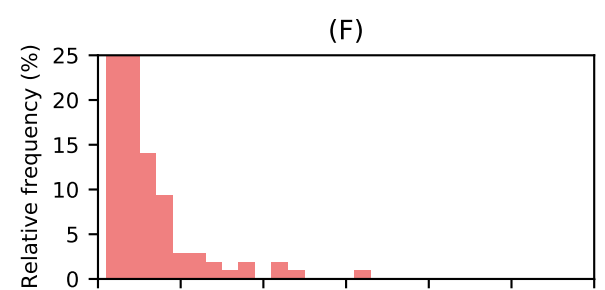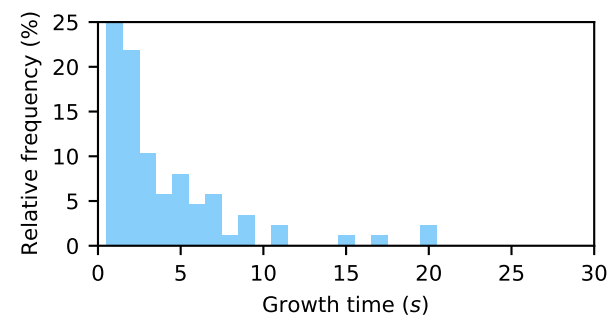

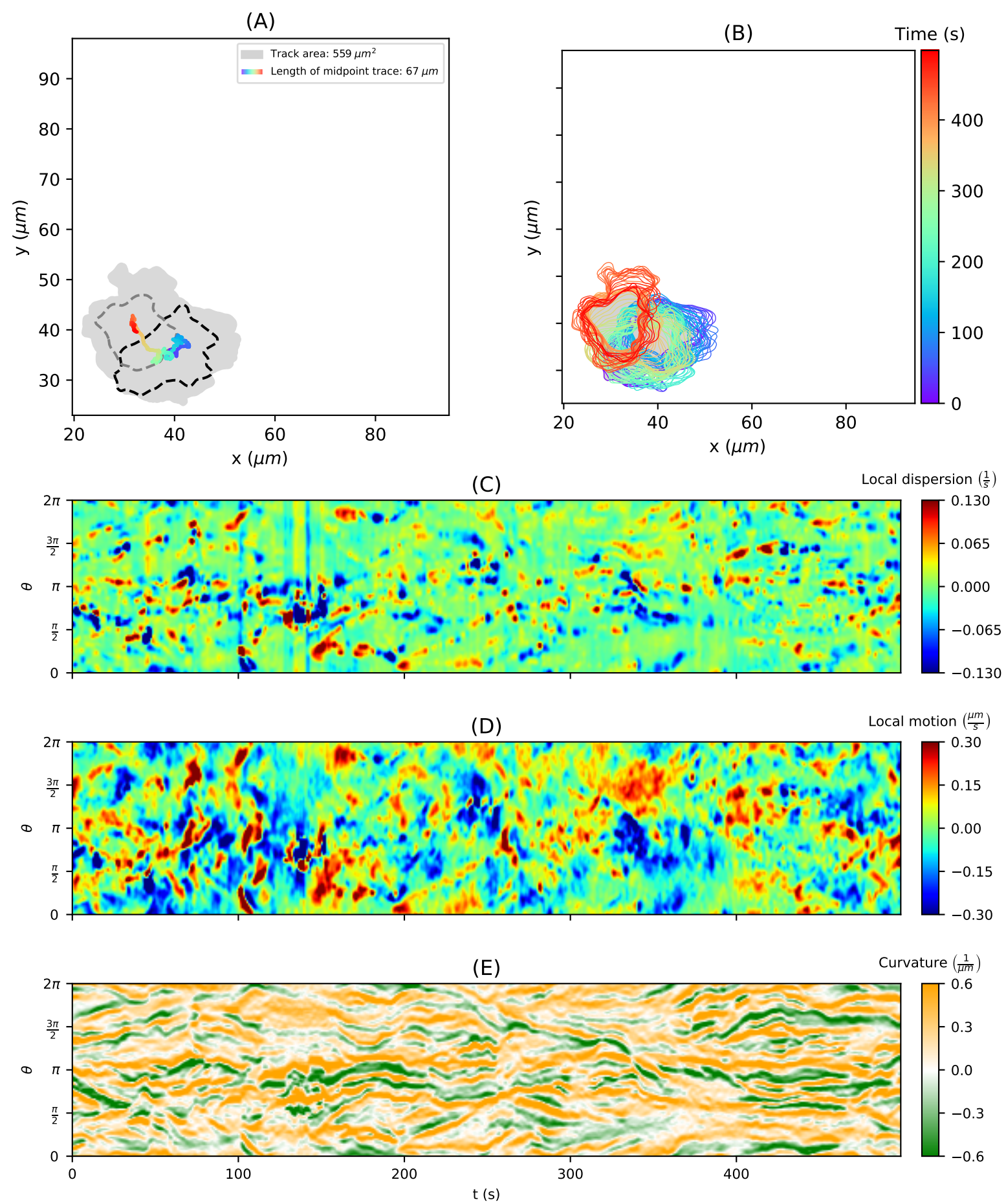

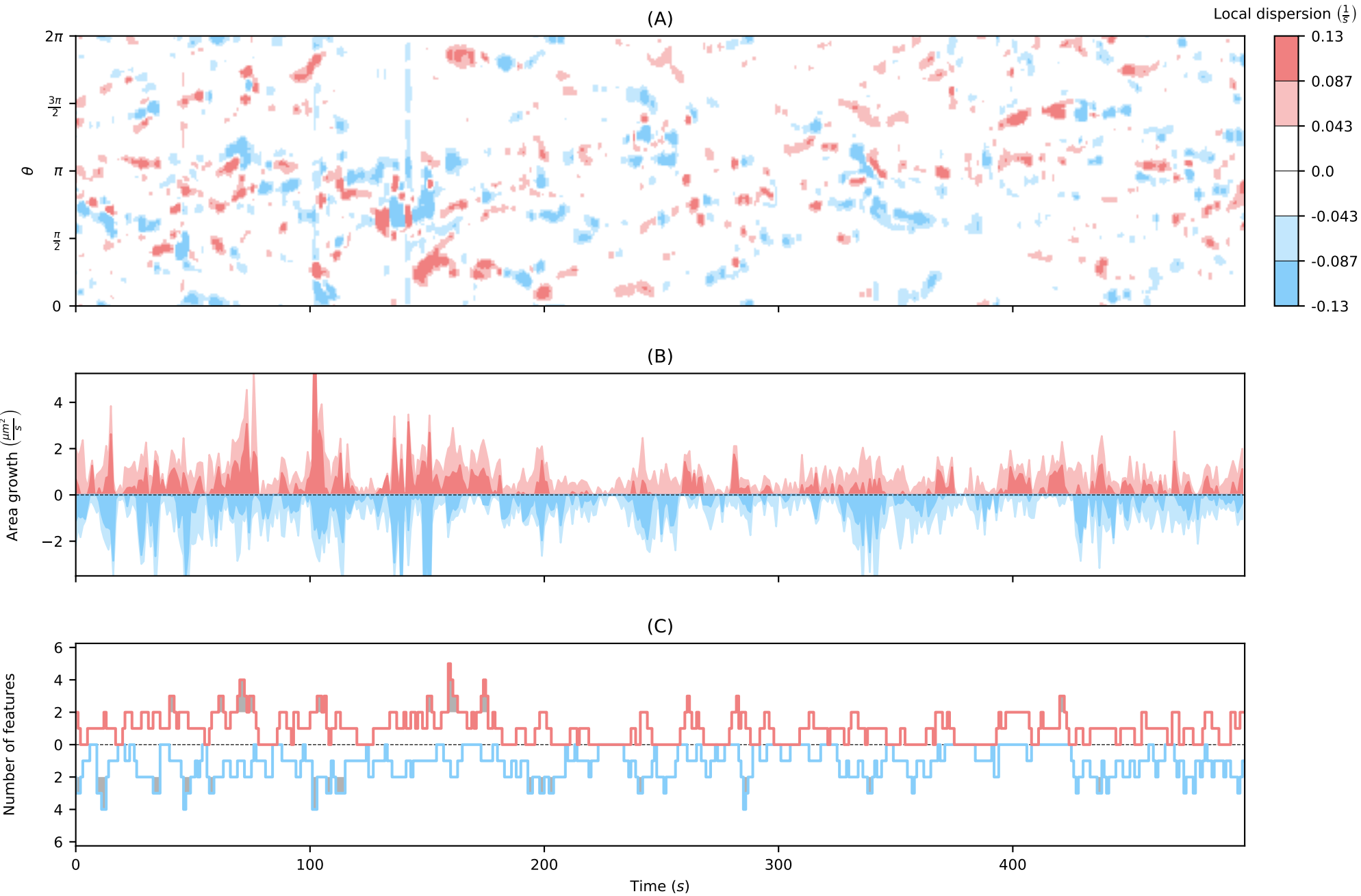

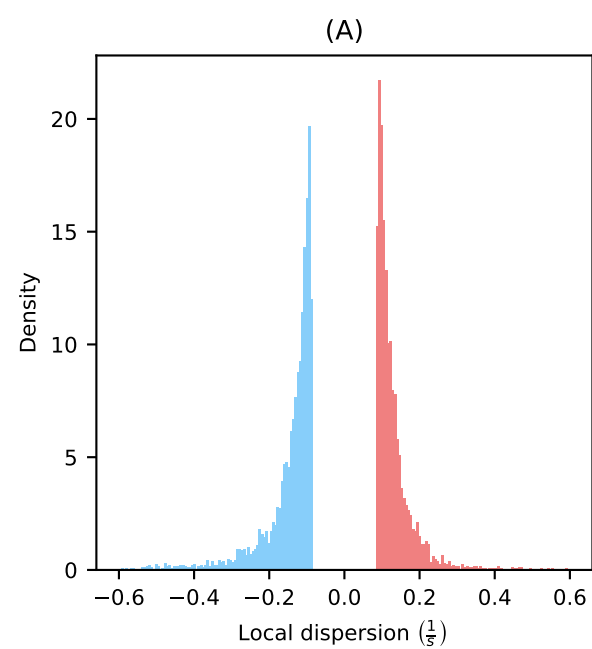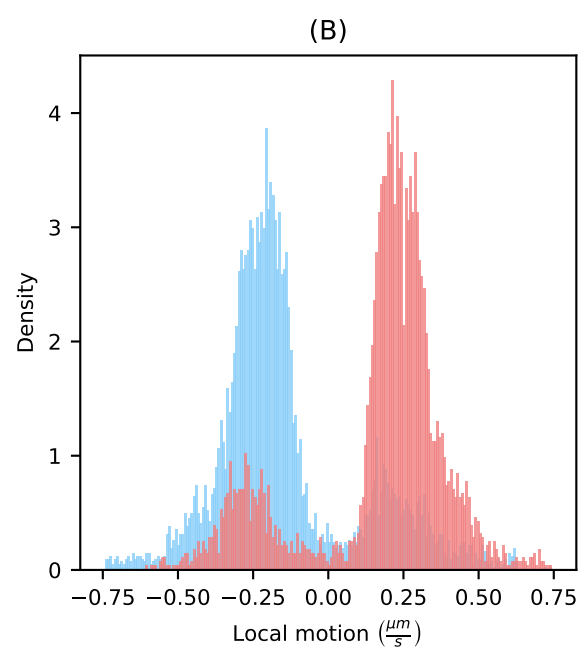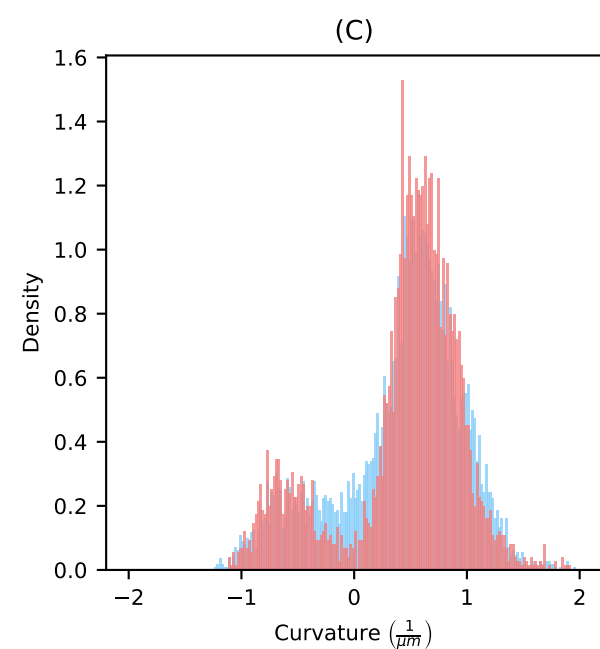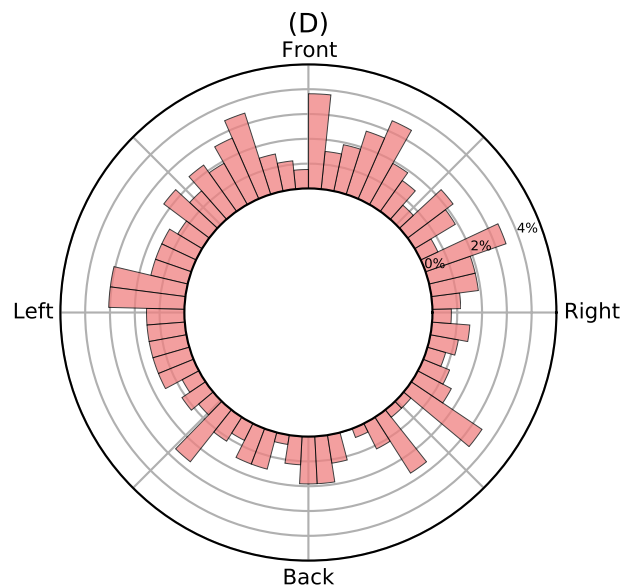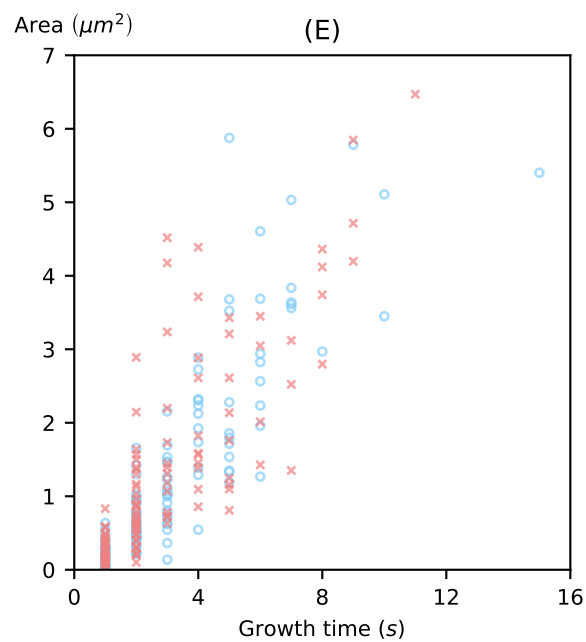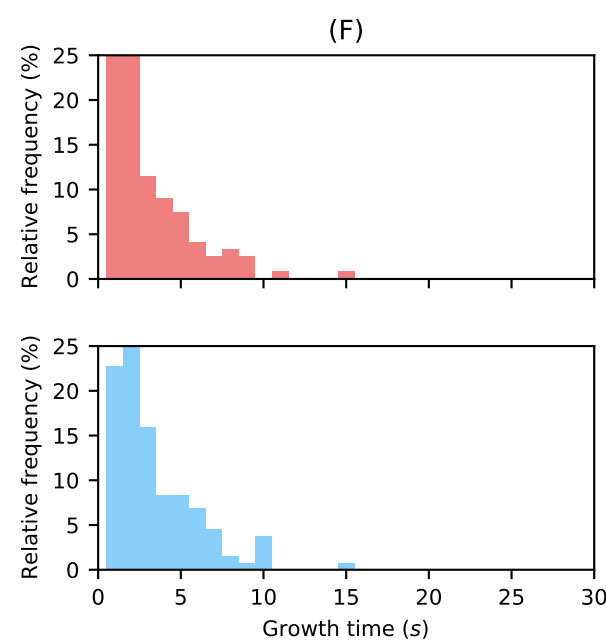

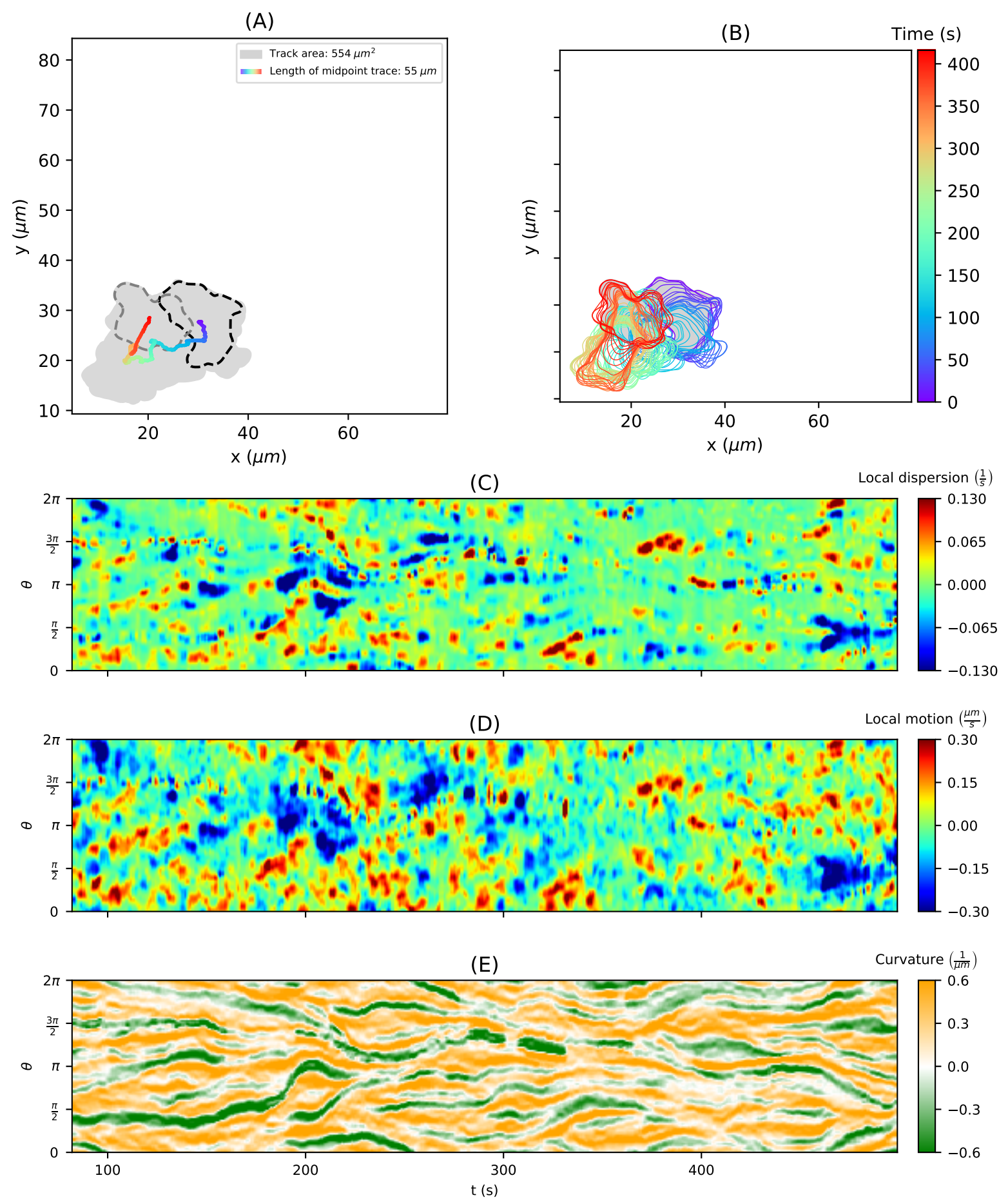

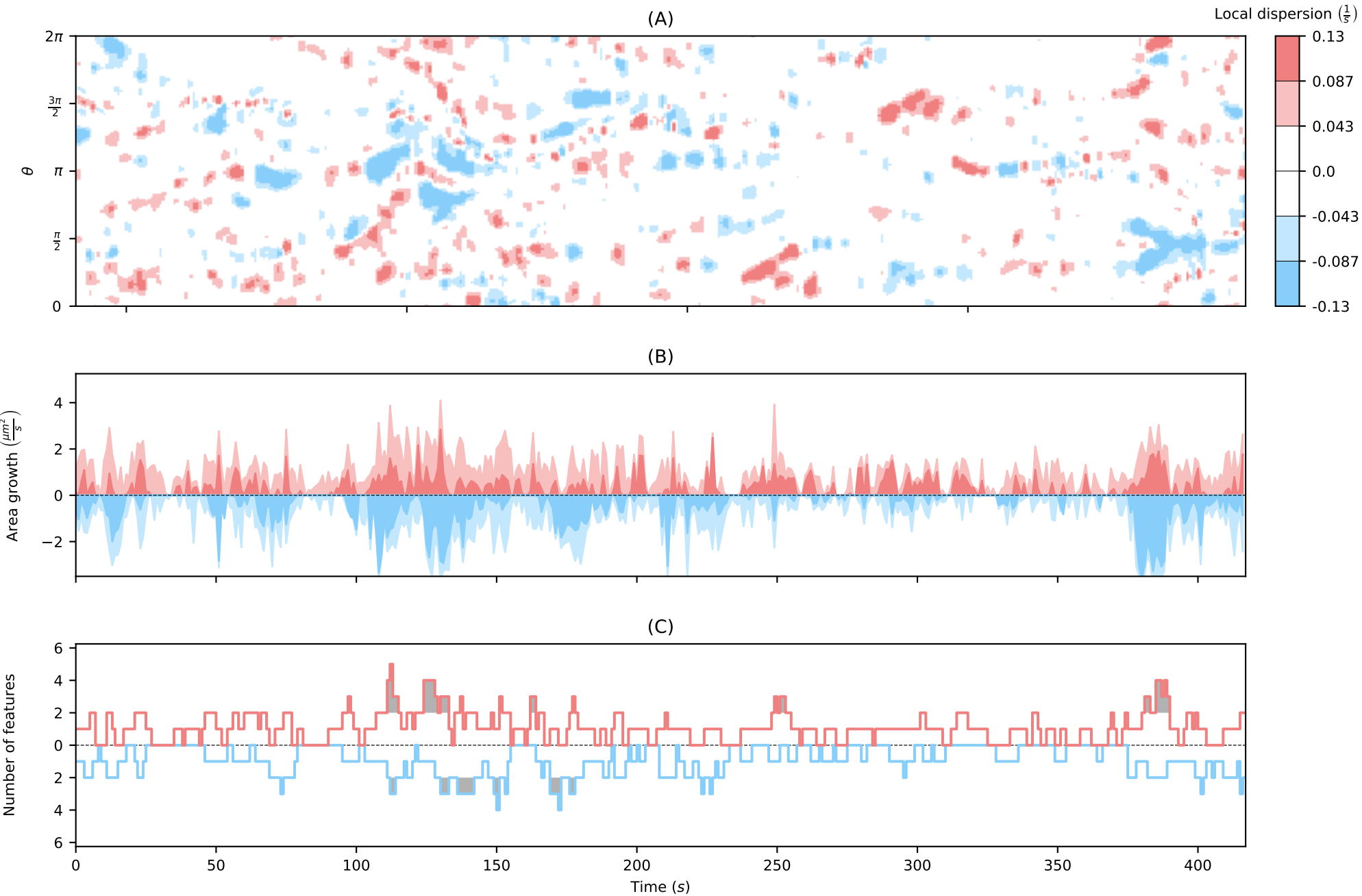

(A)

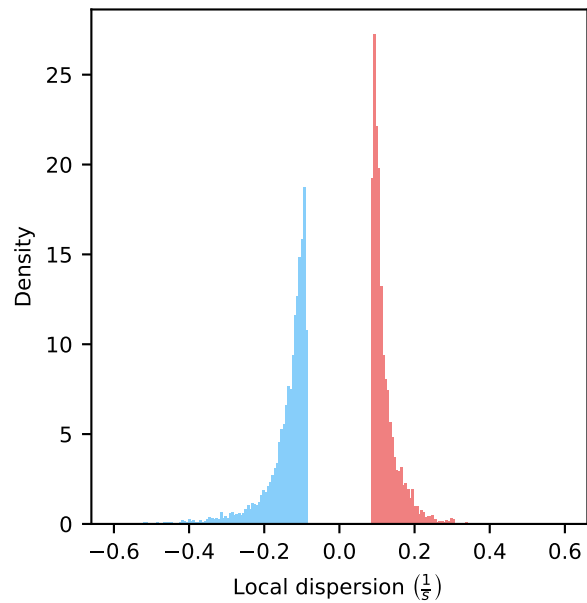

(B)

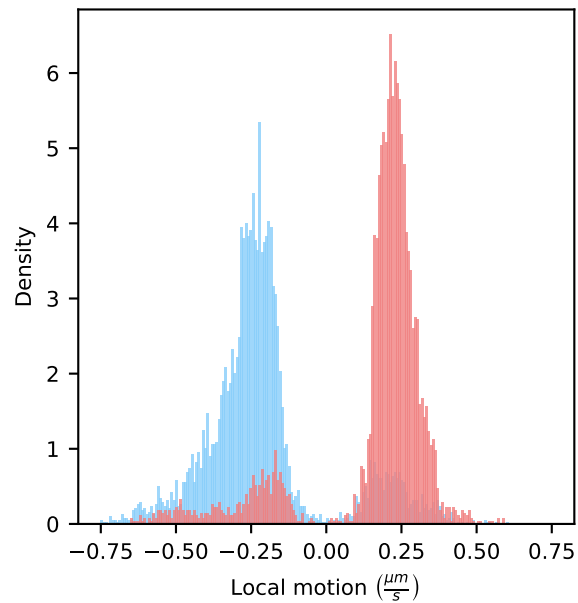

(C)

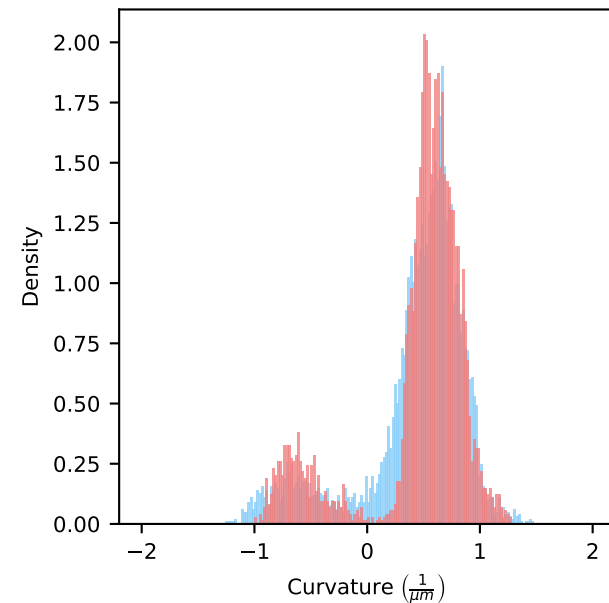

(D)

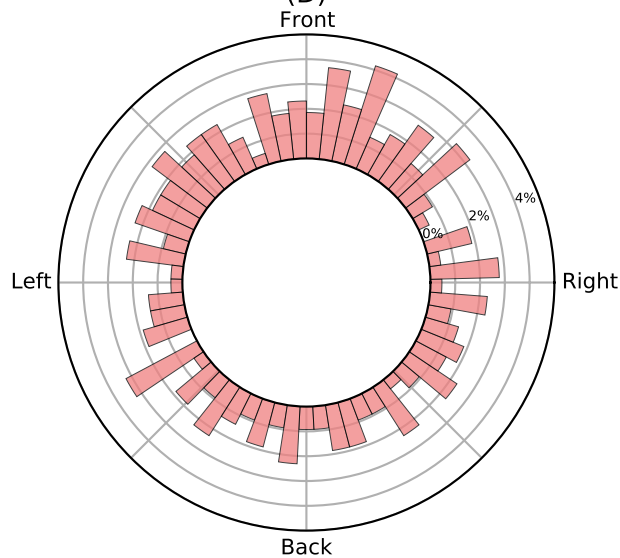

(E)

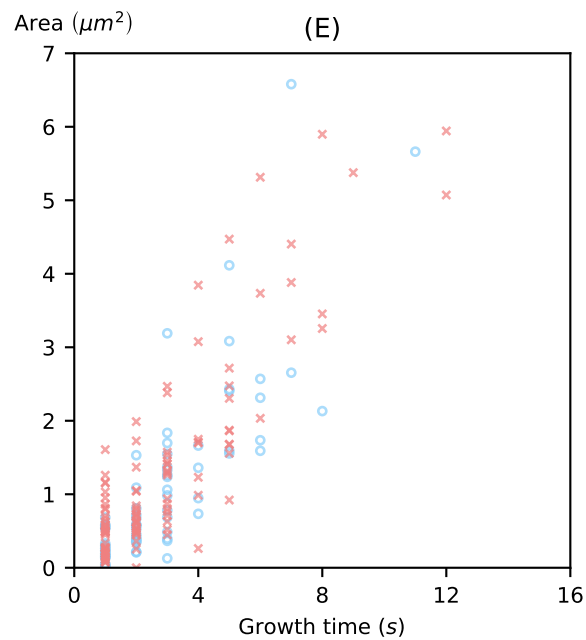

(F)

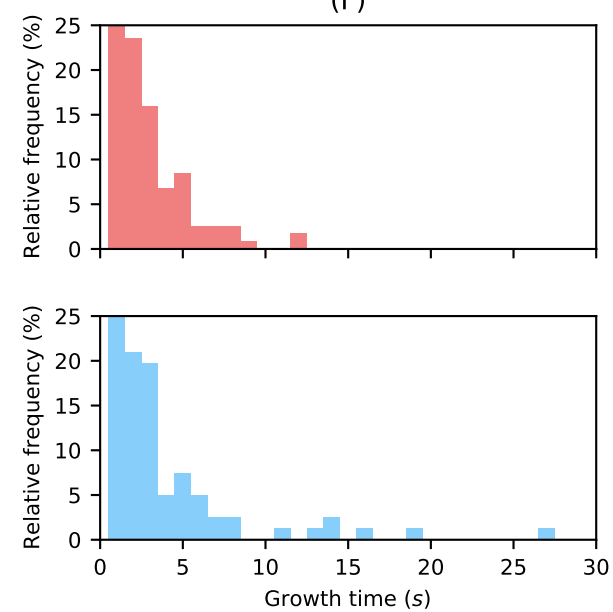

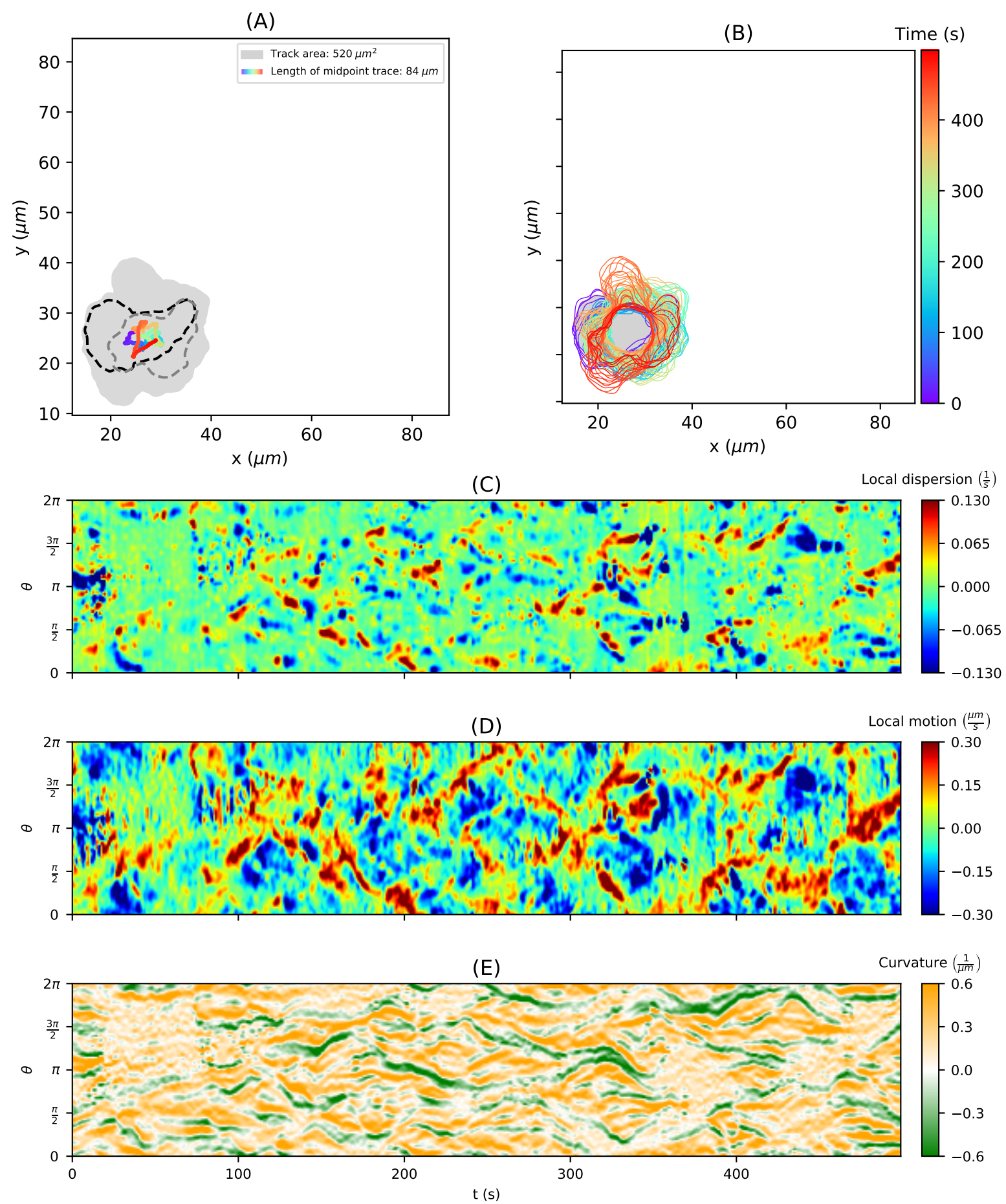

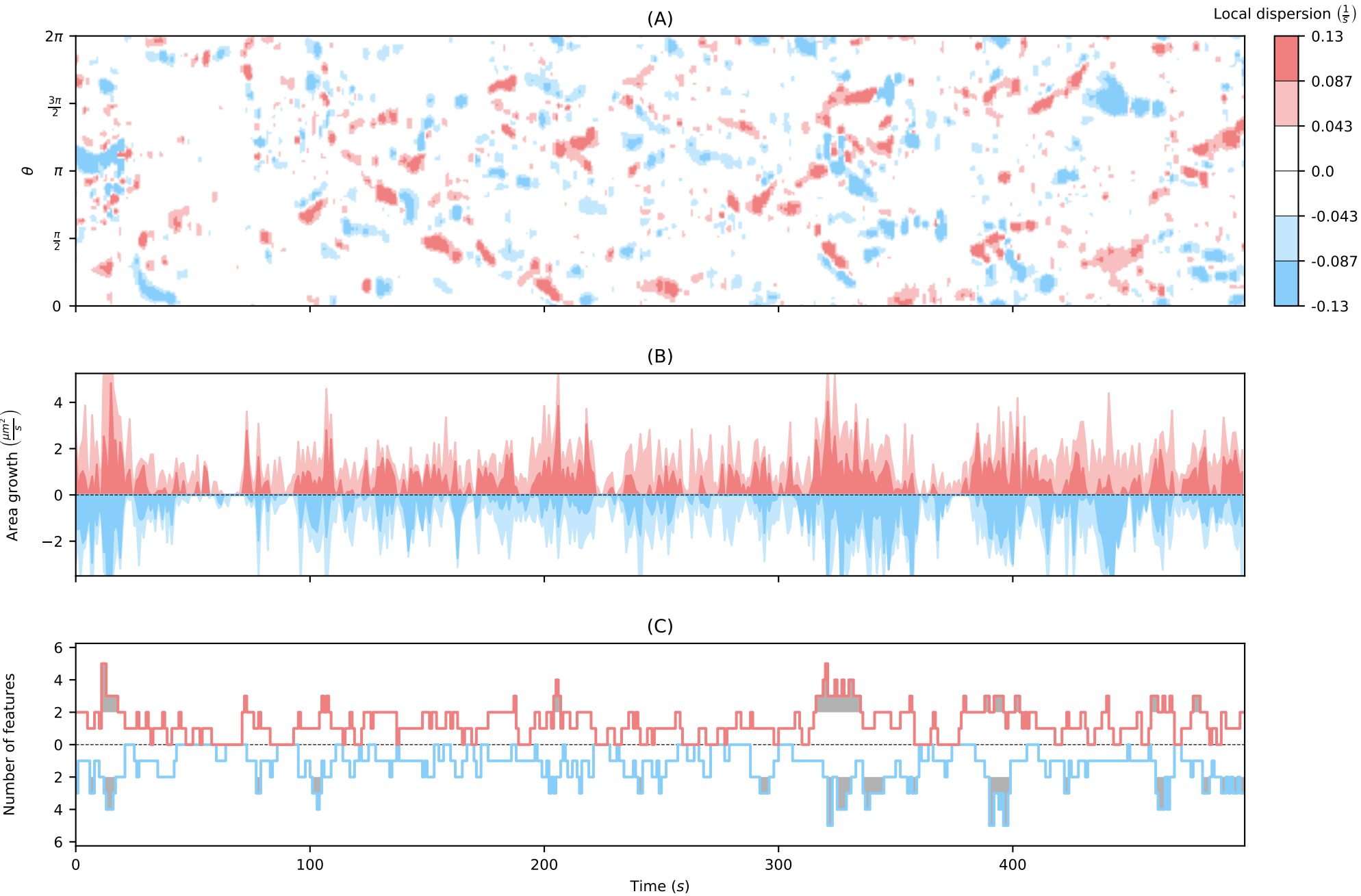

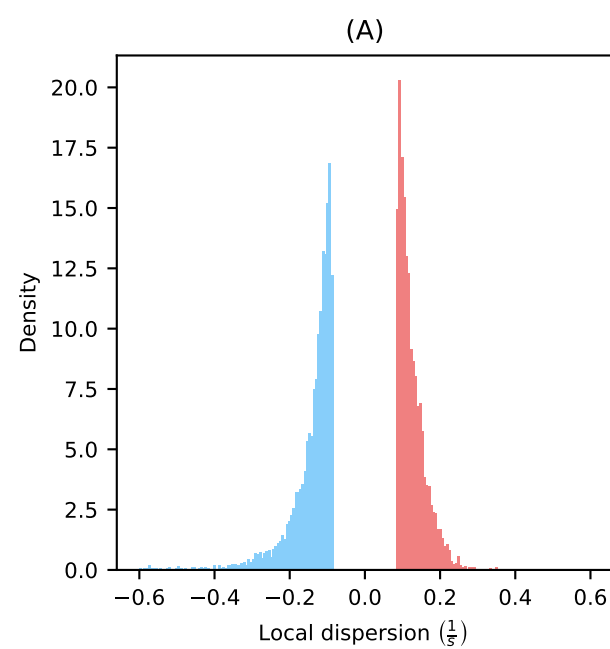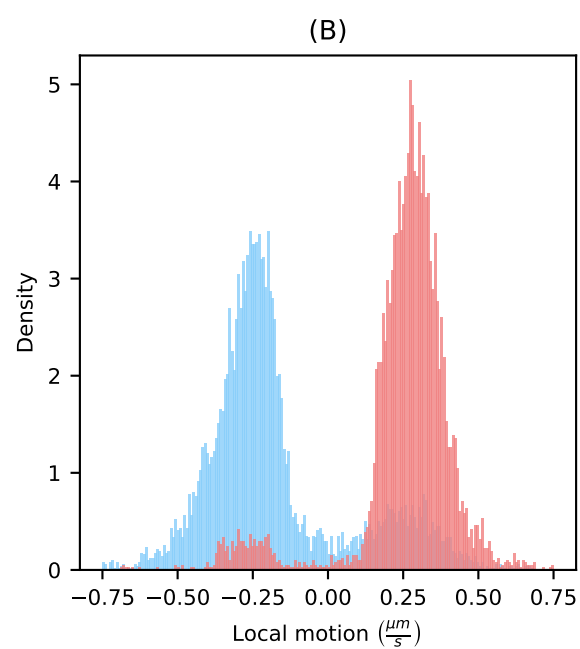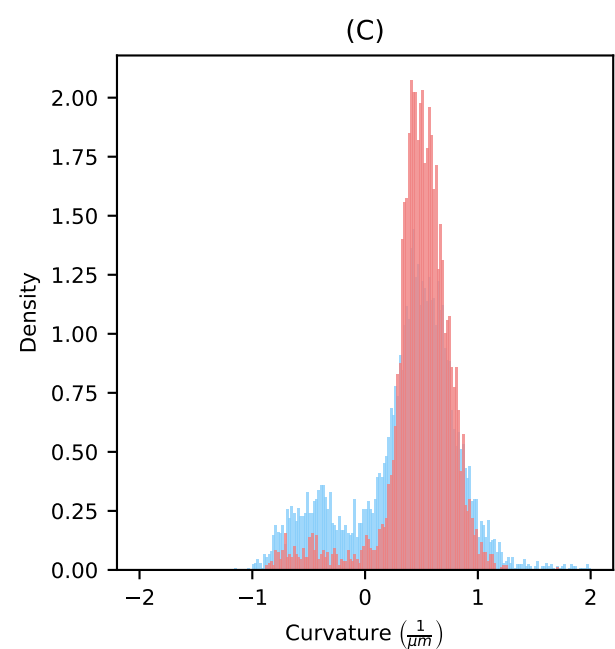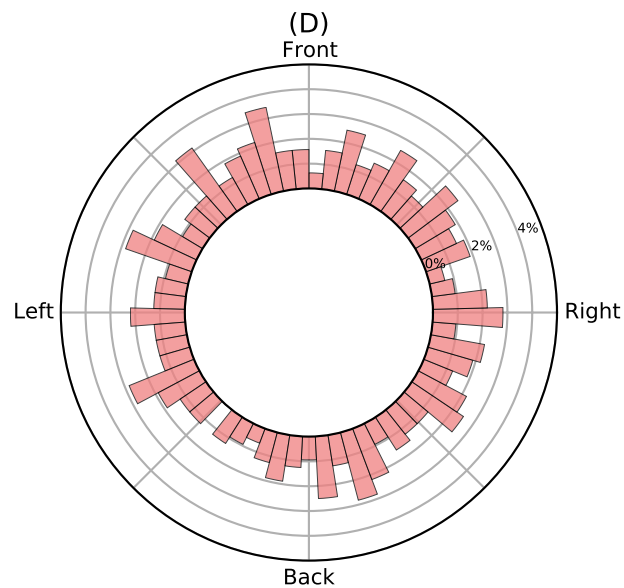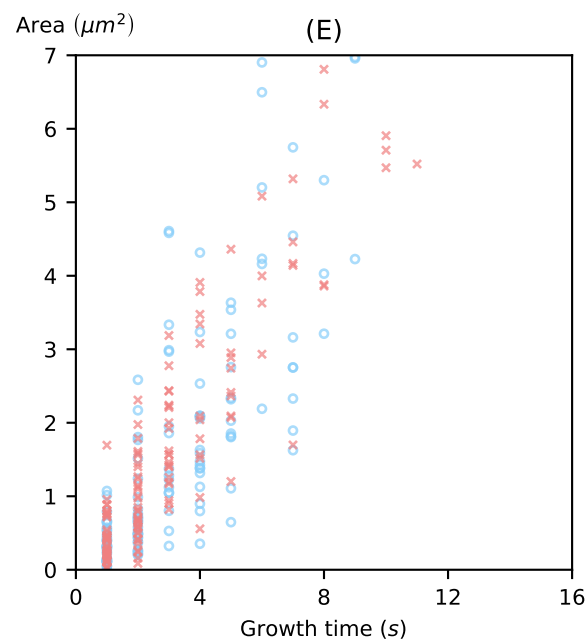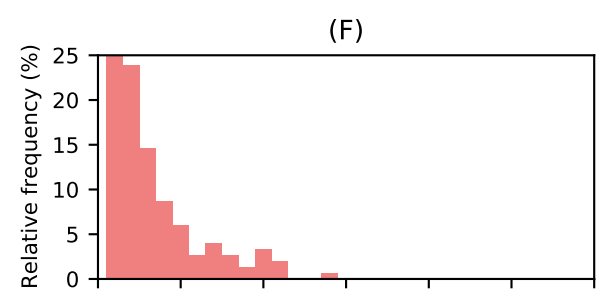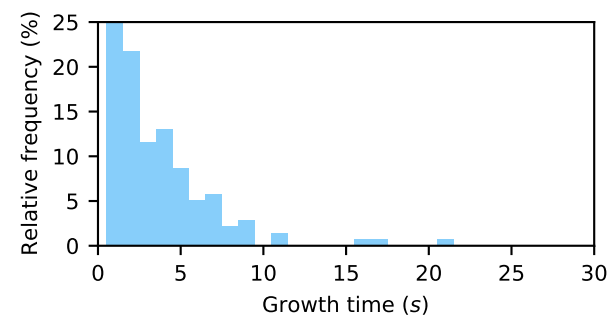

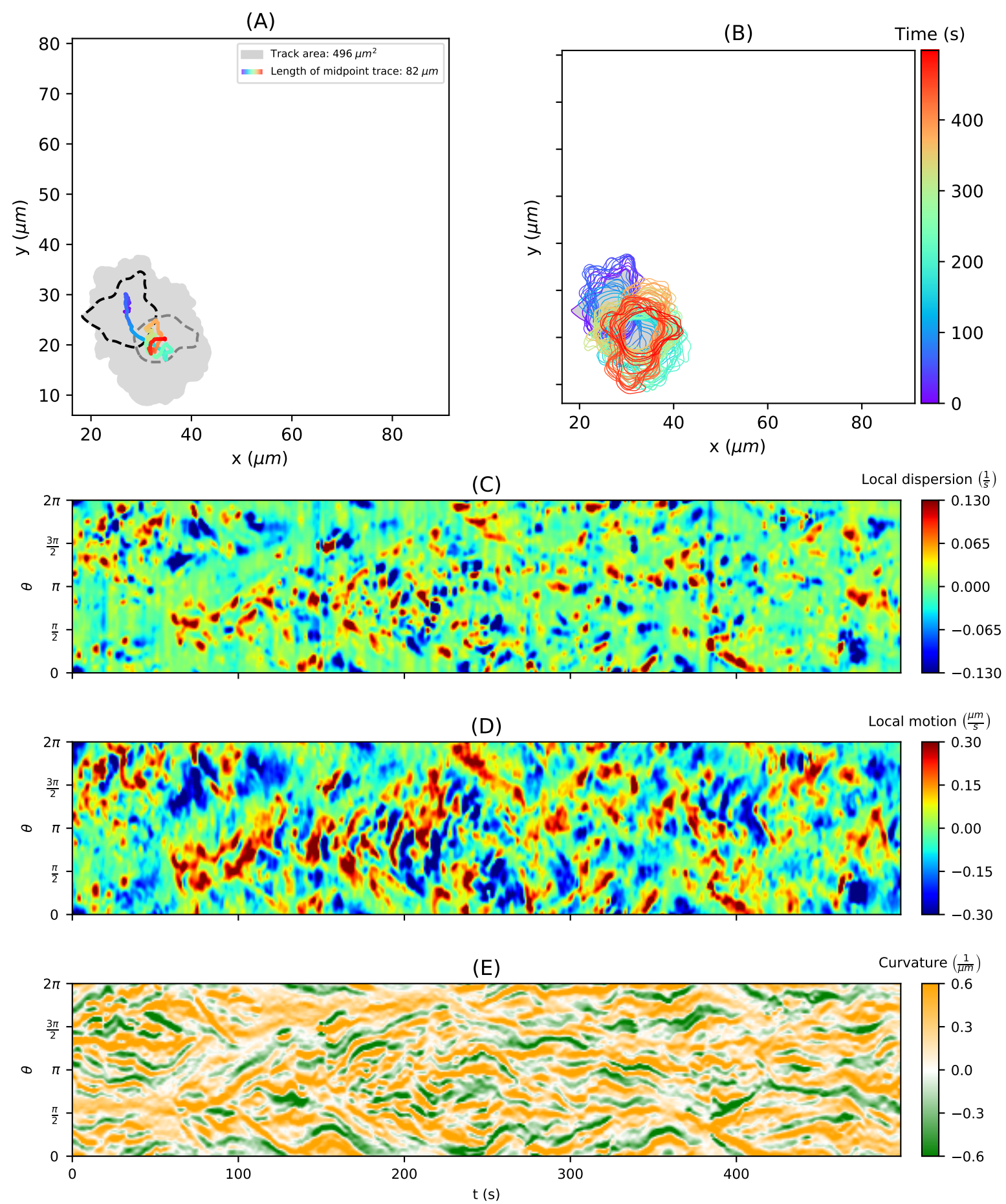

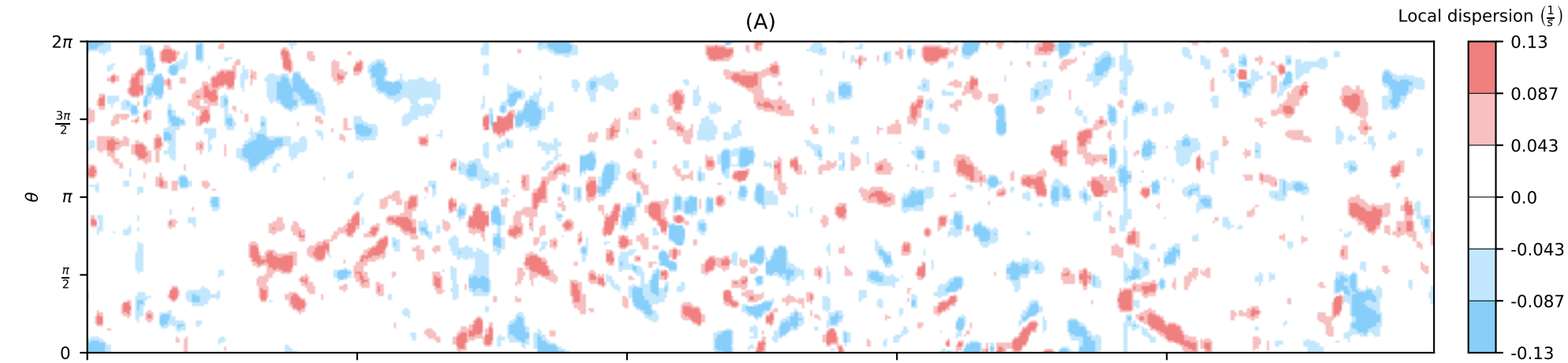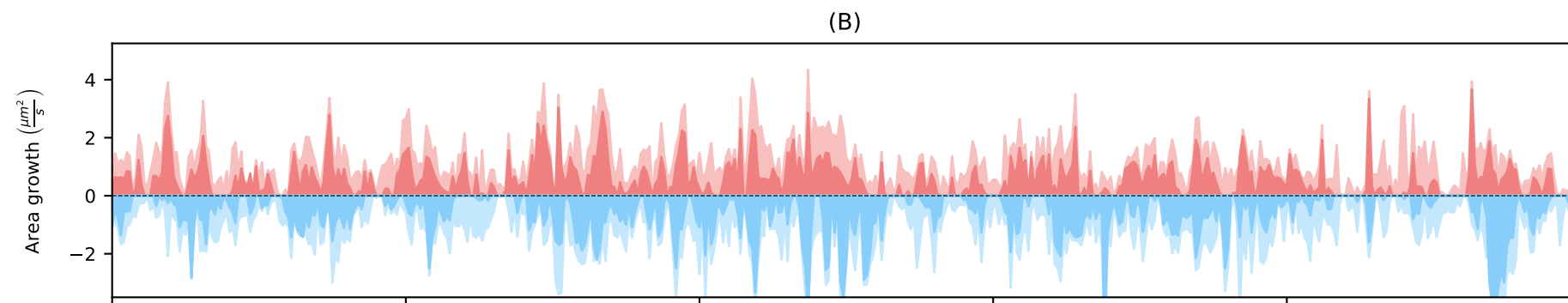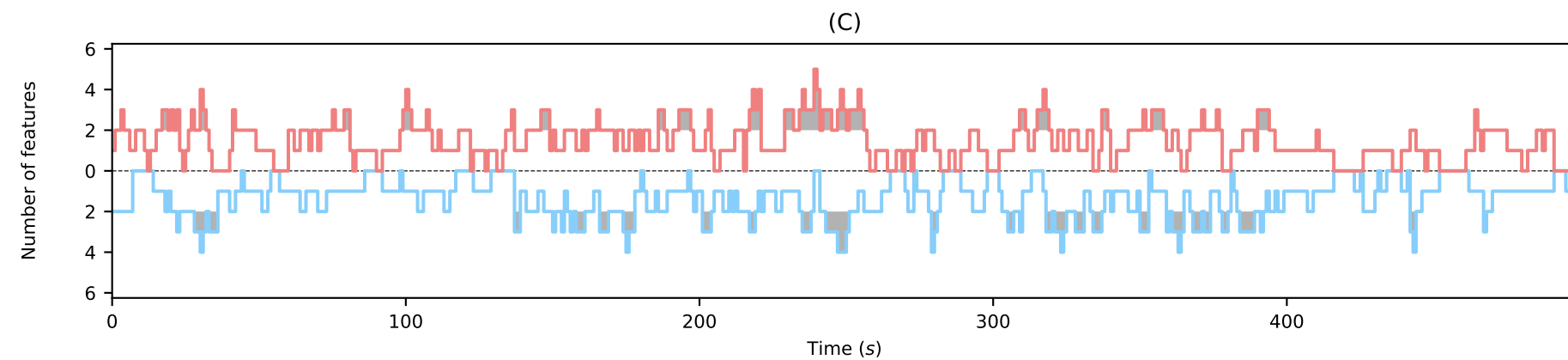

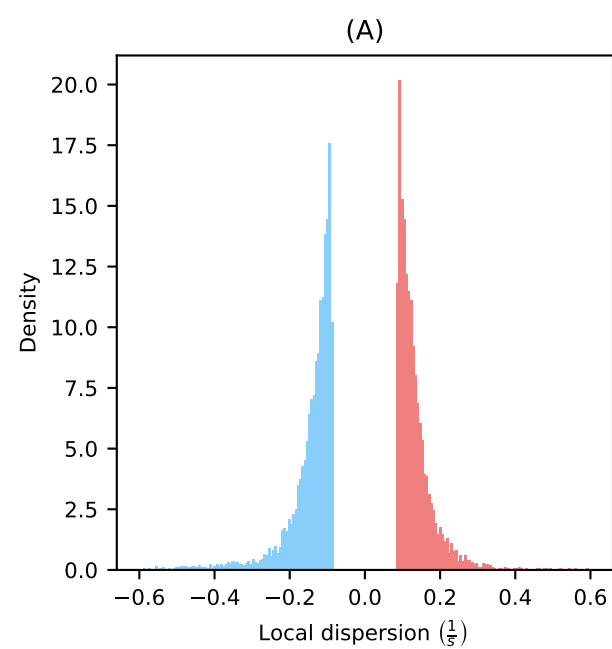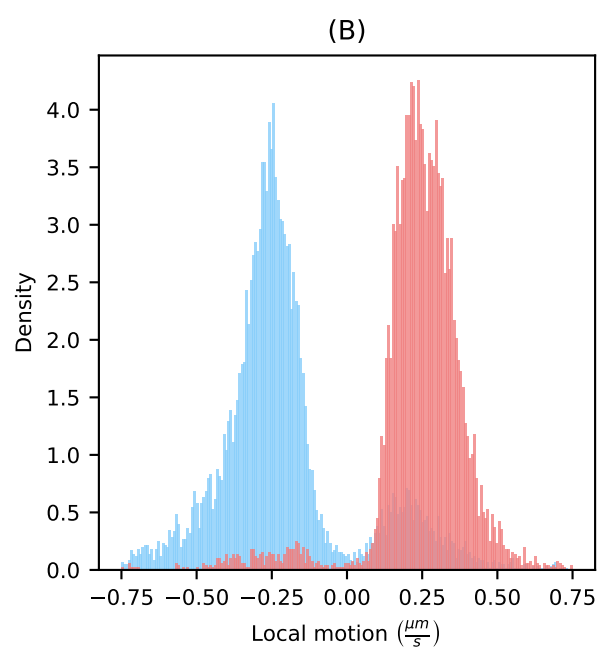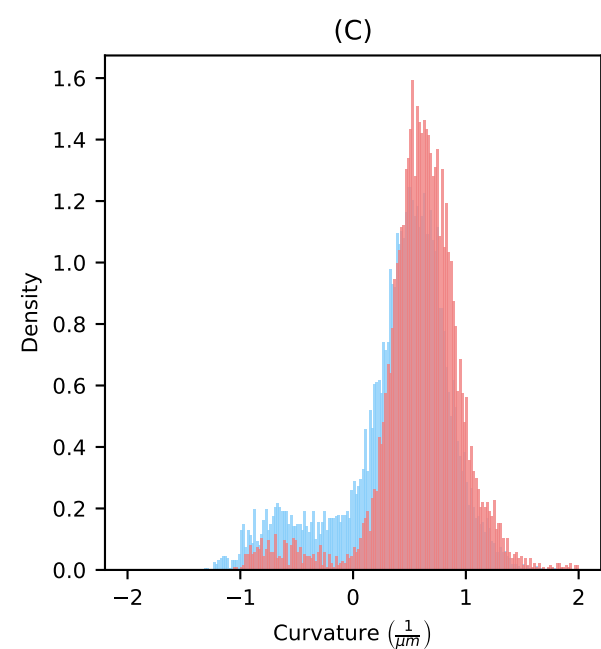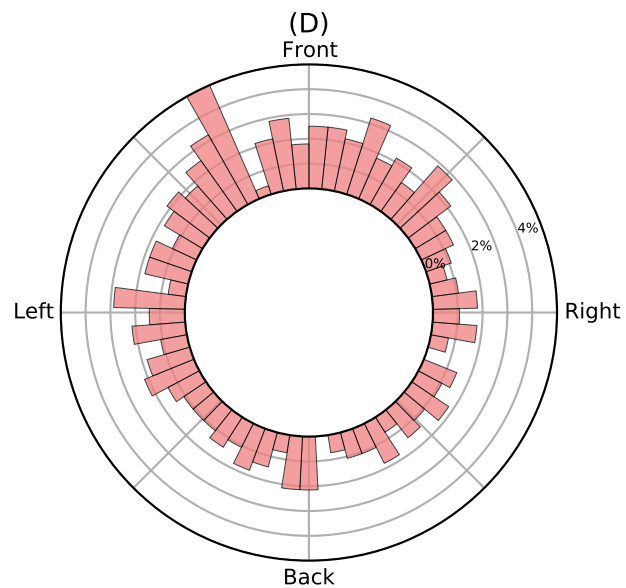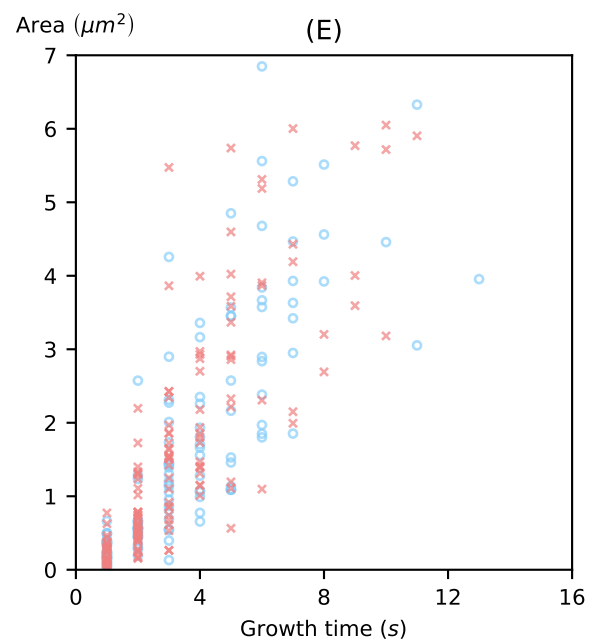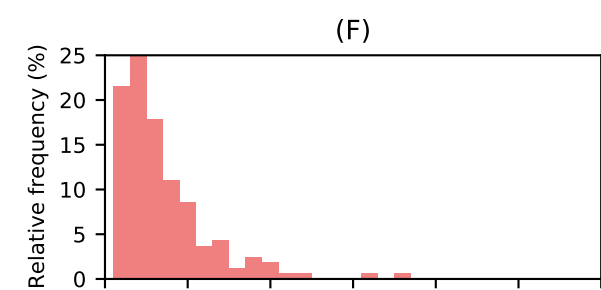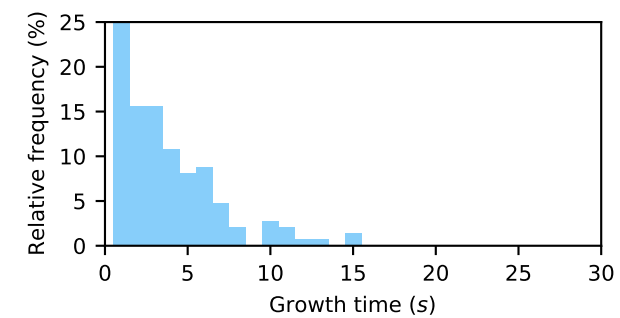

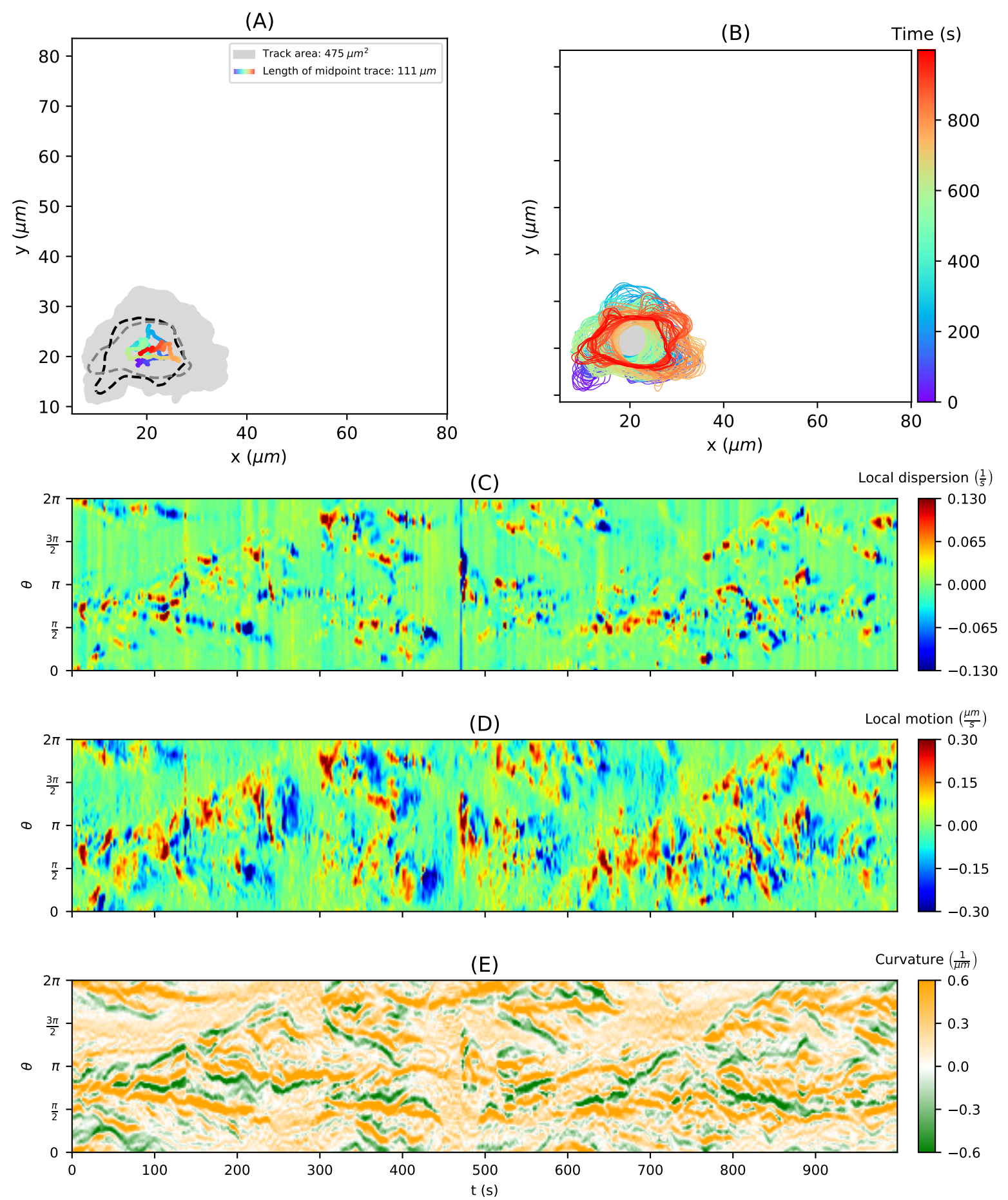

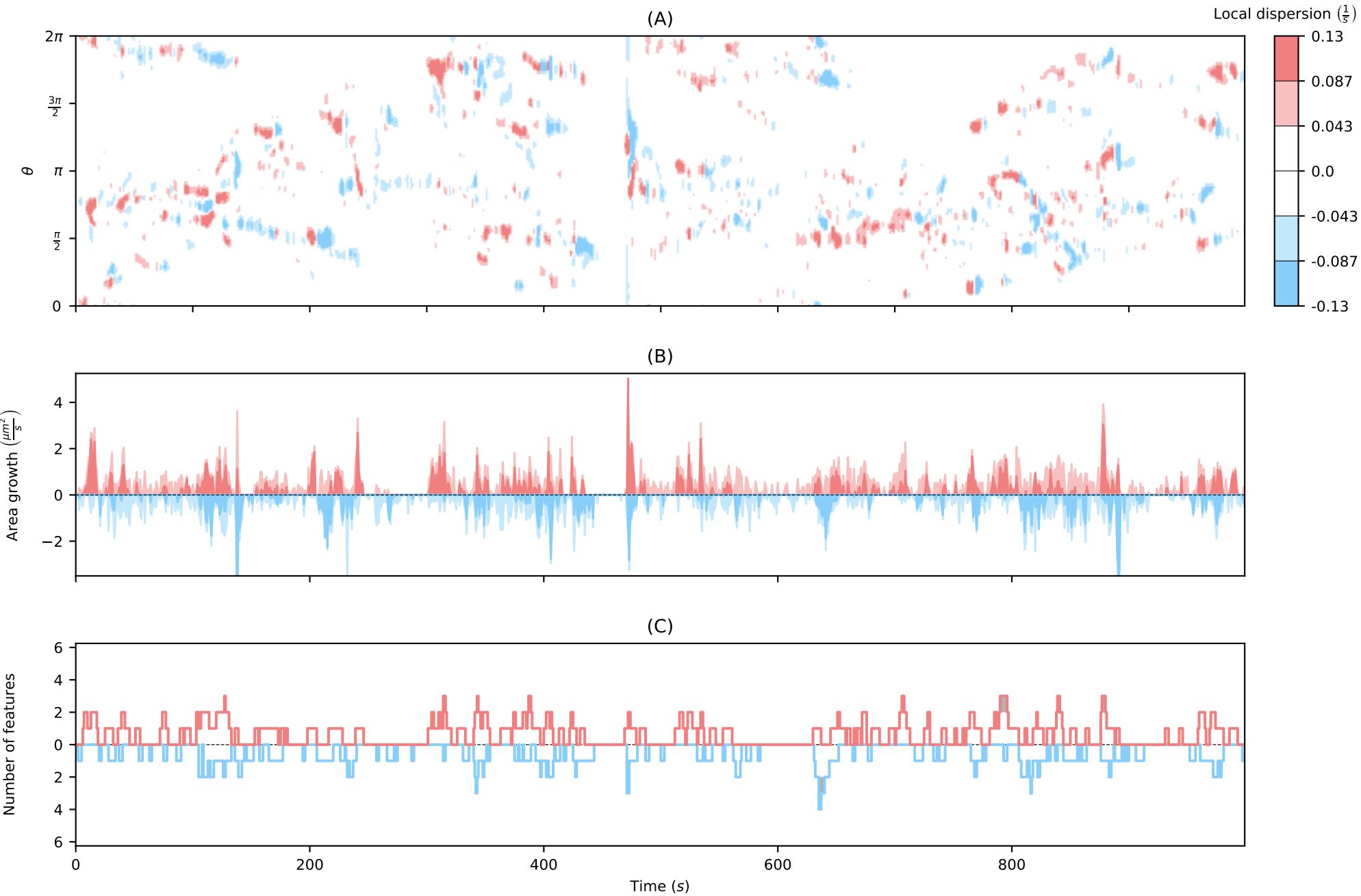

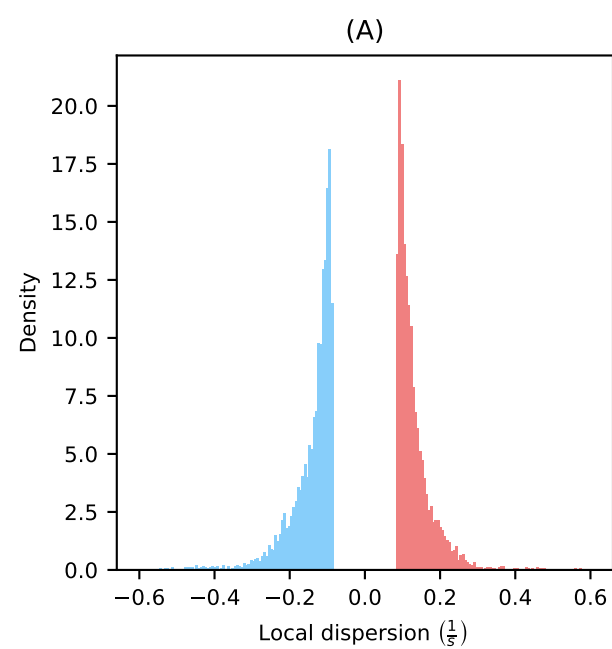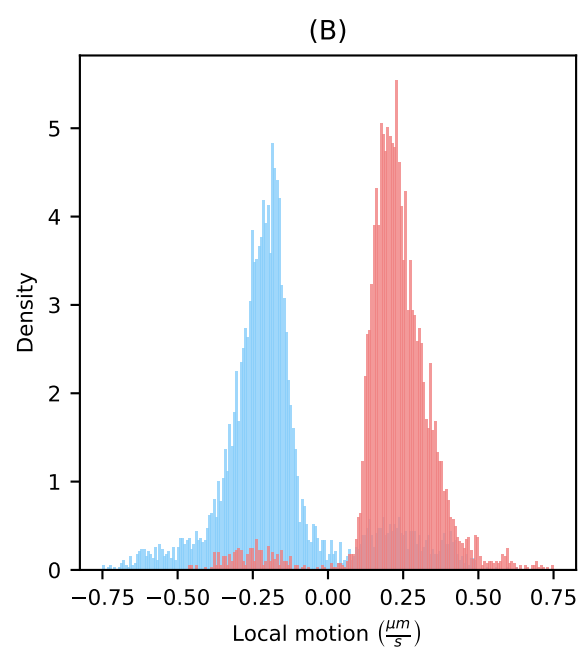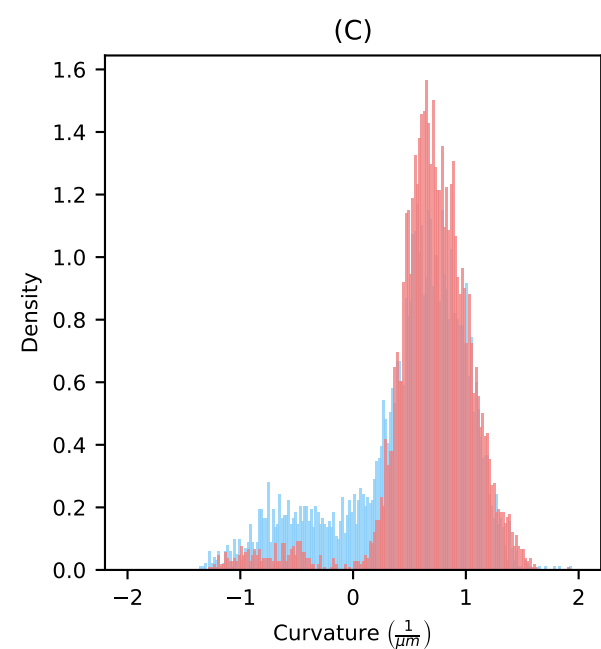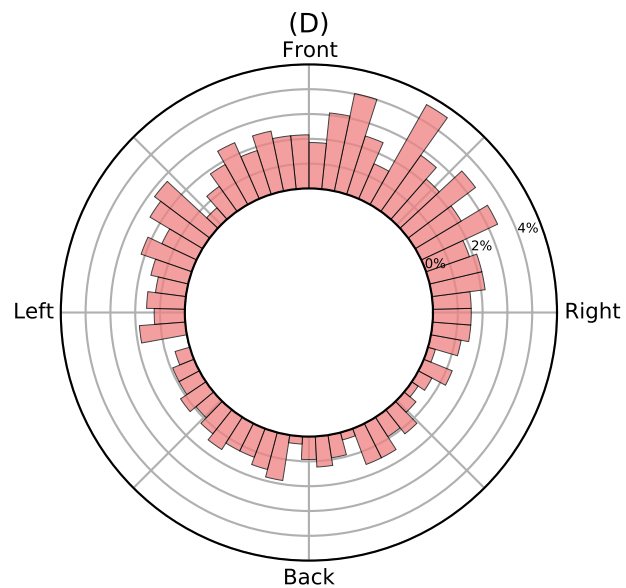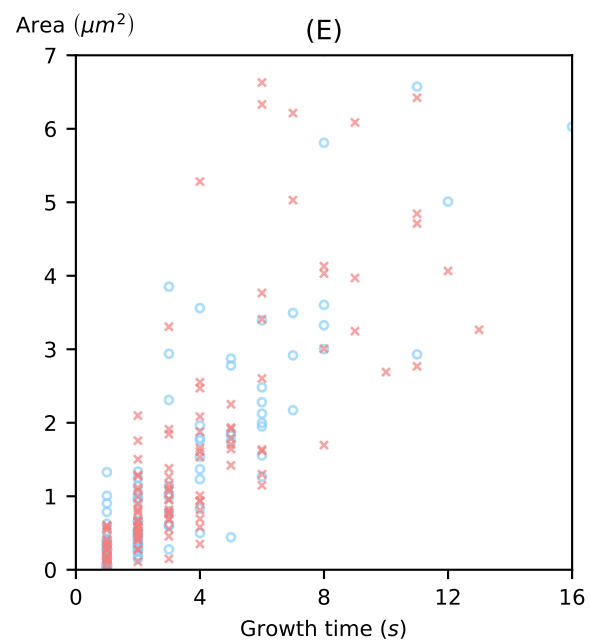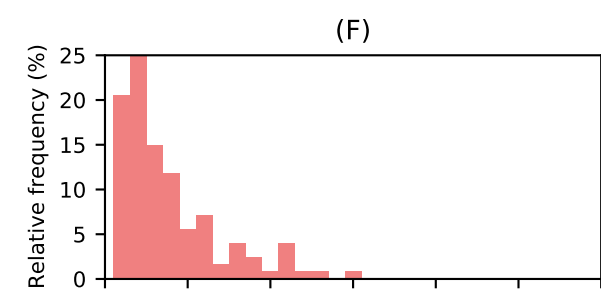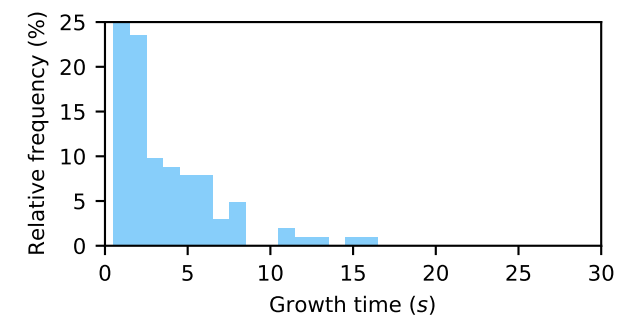

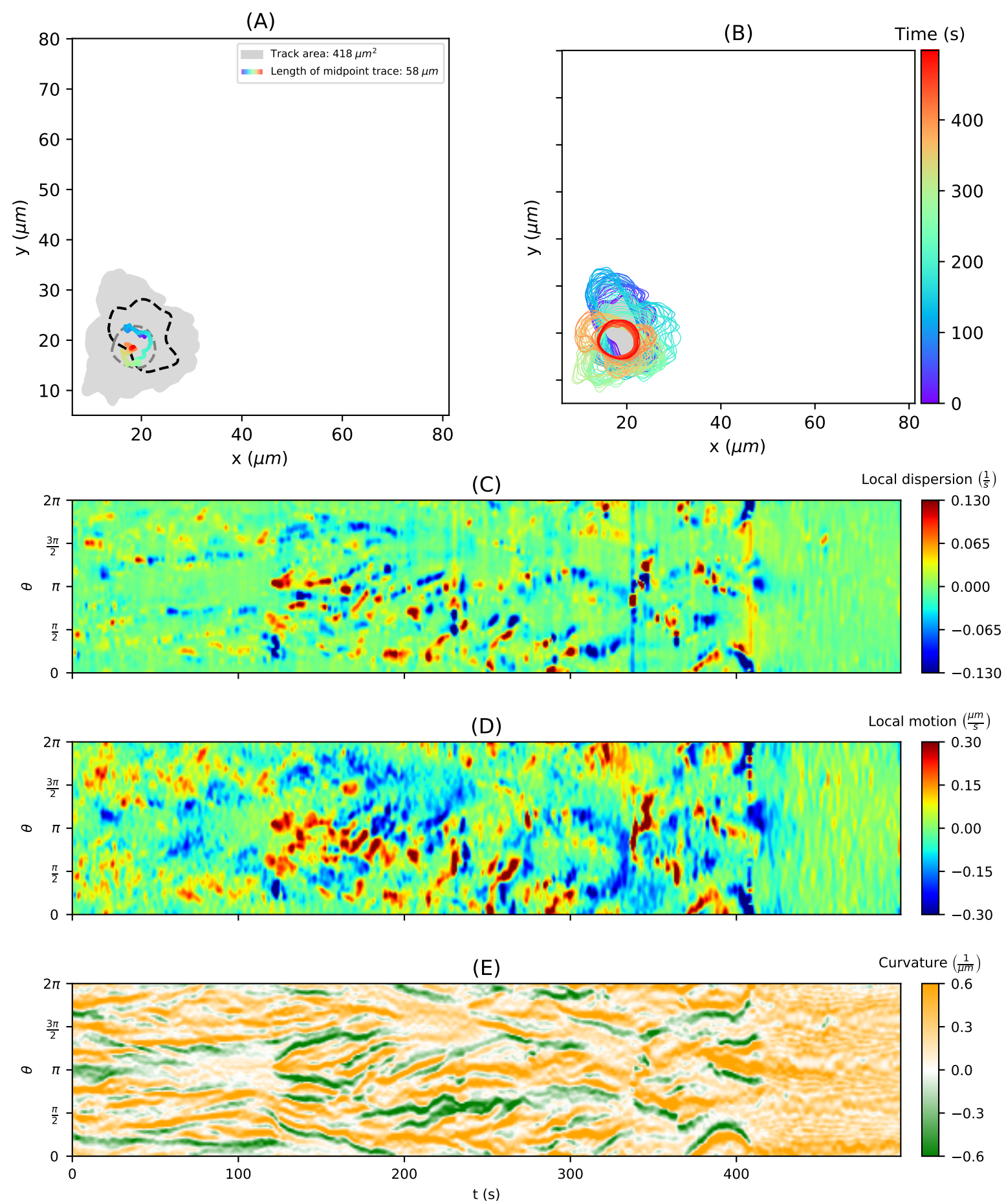

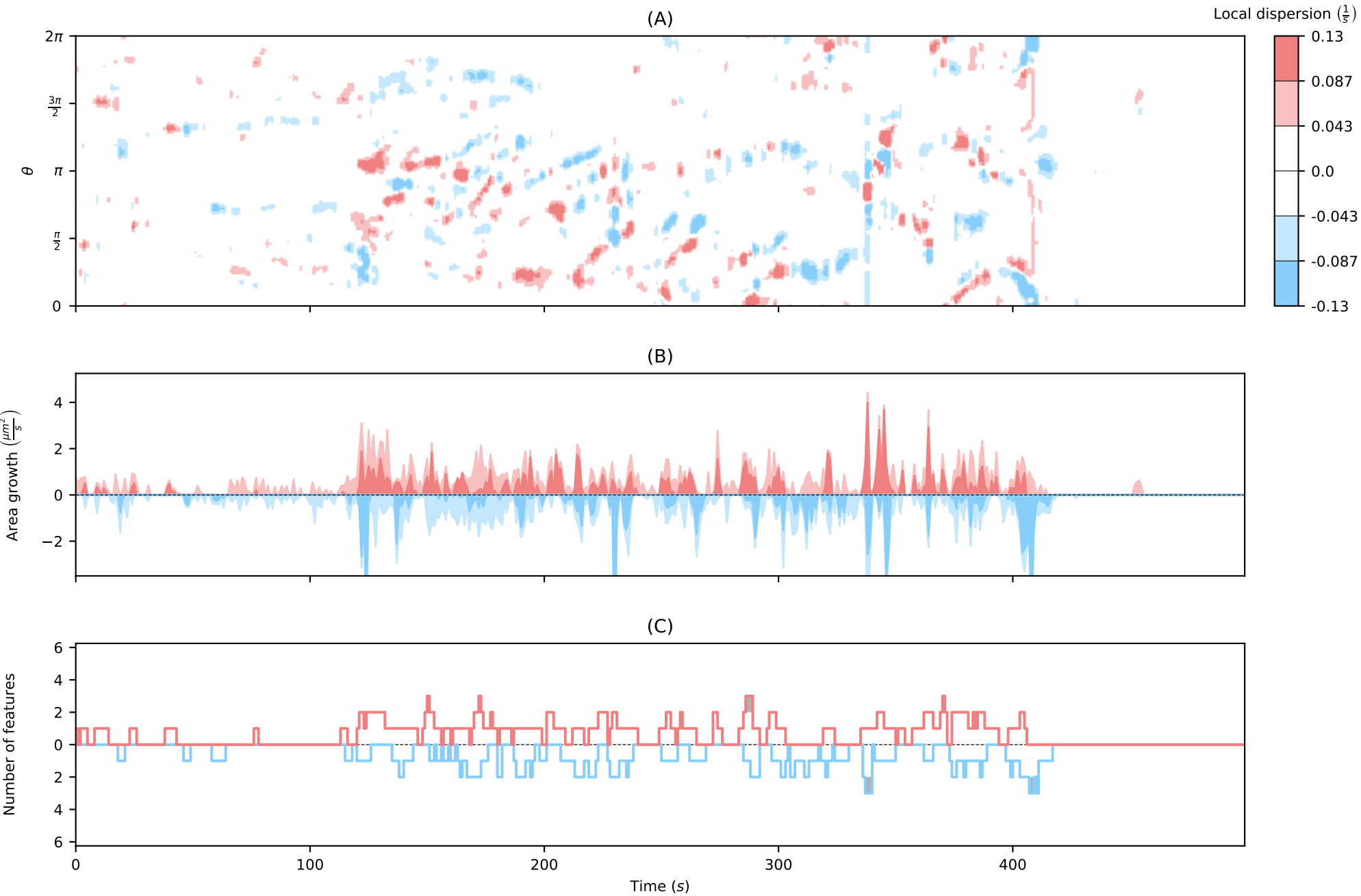

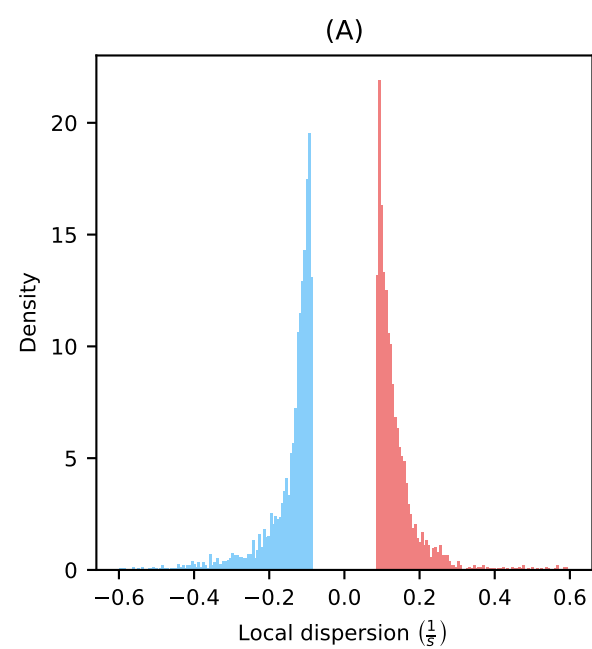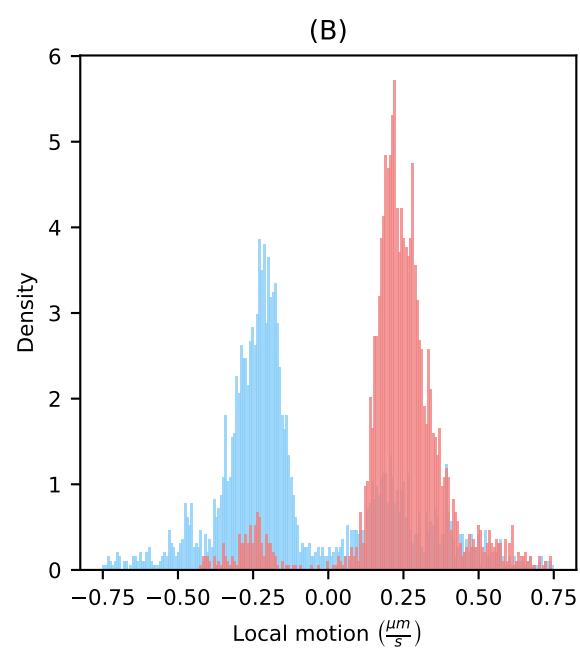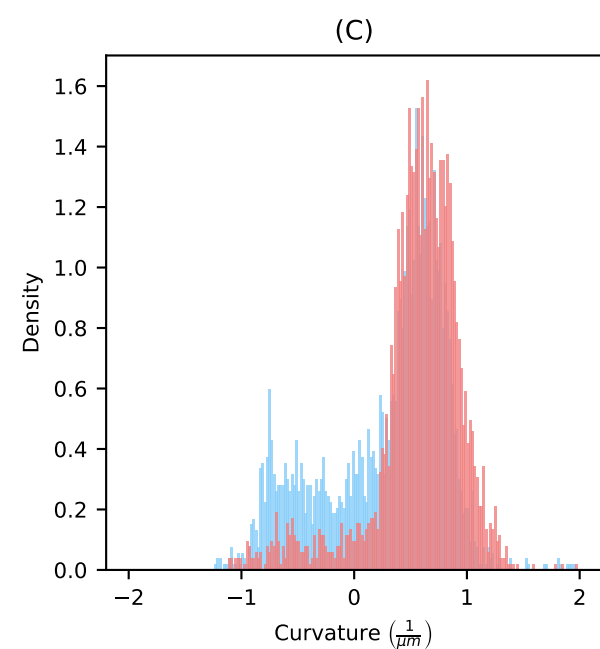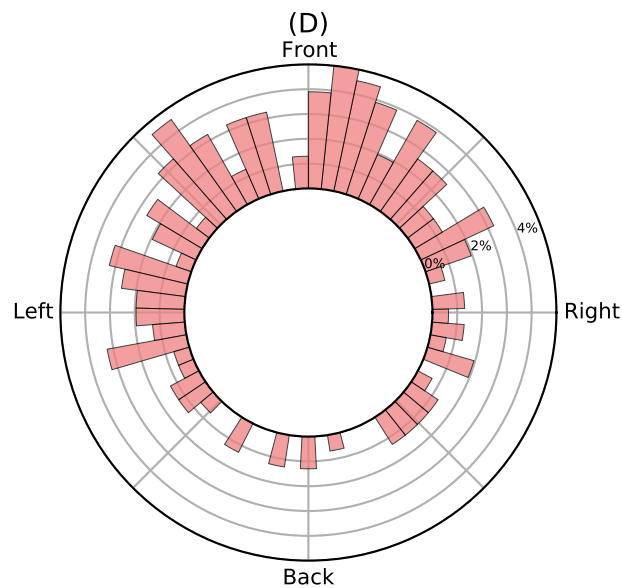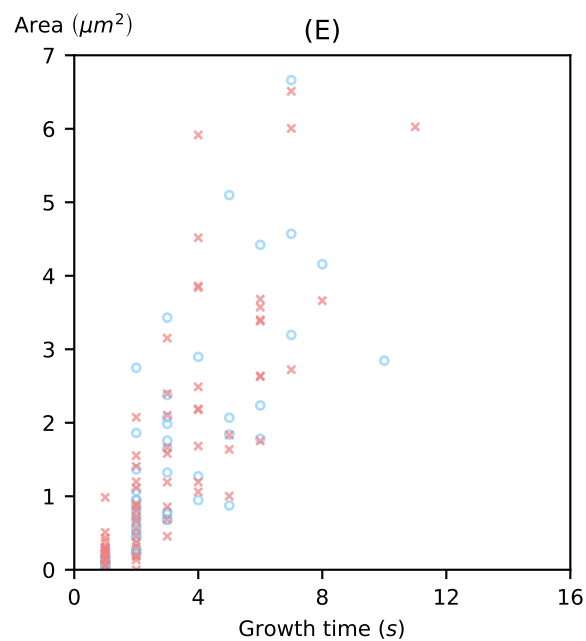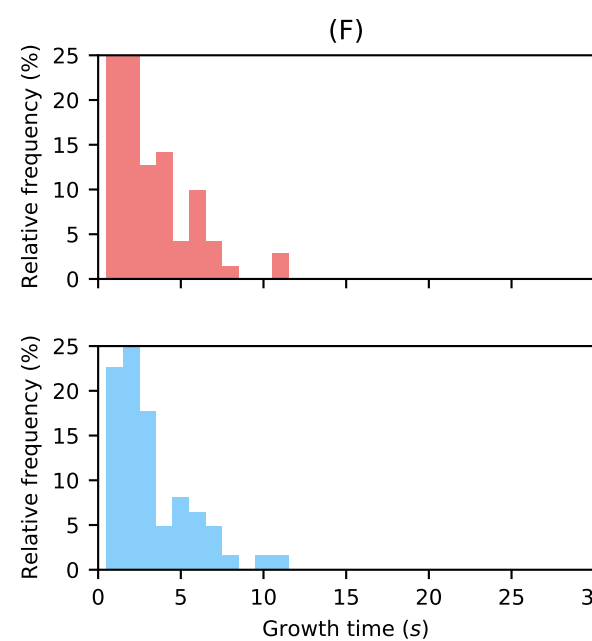

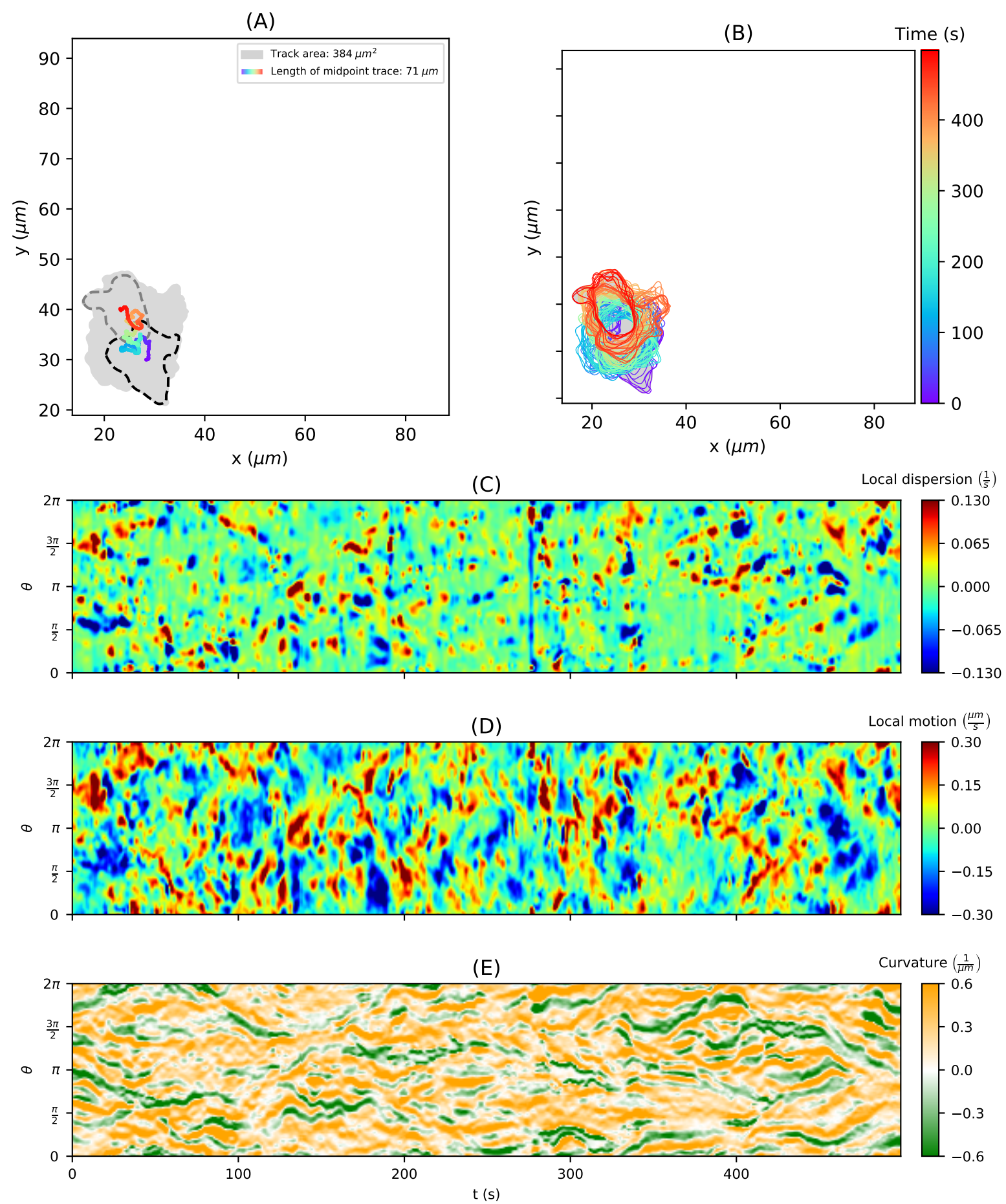

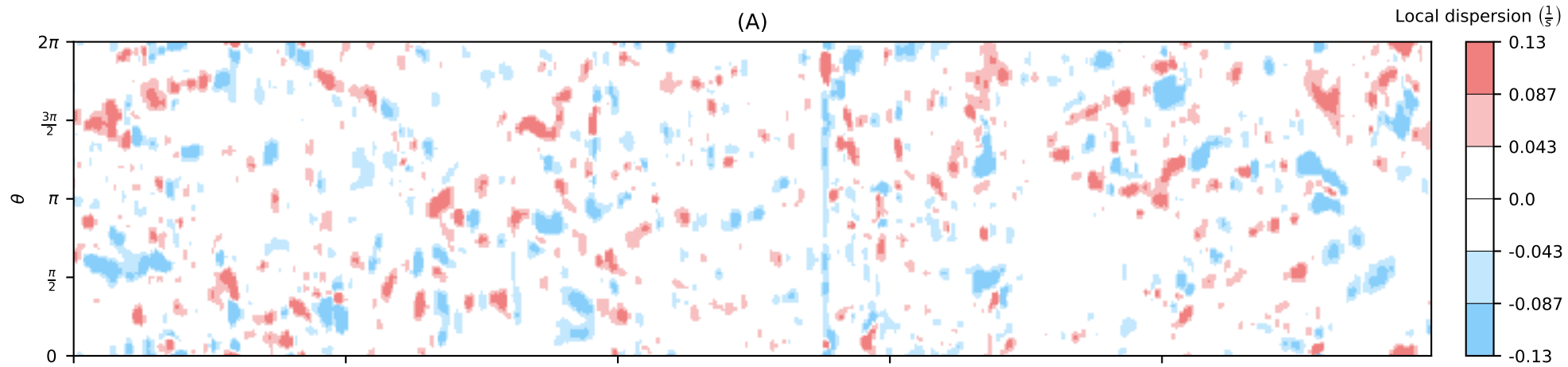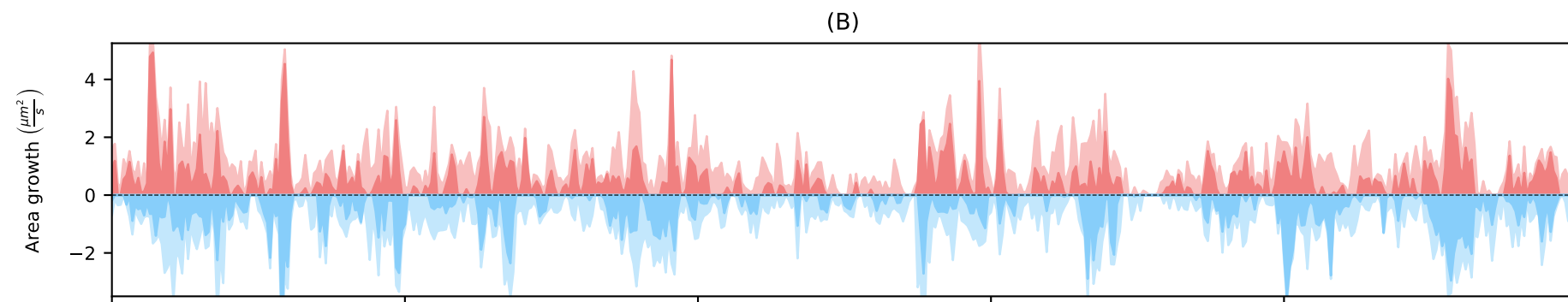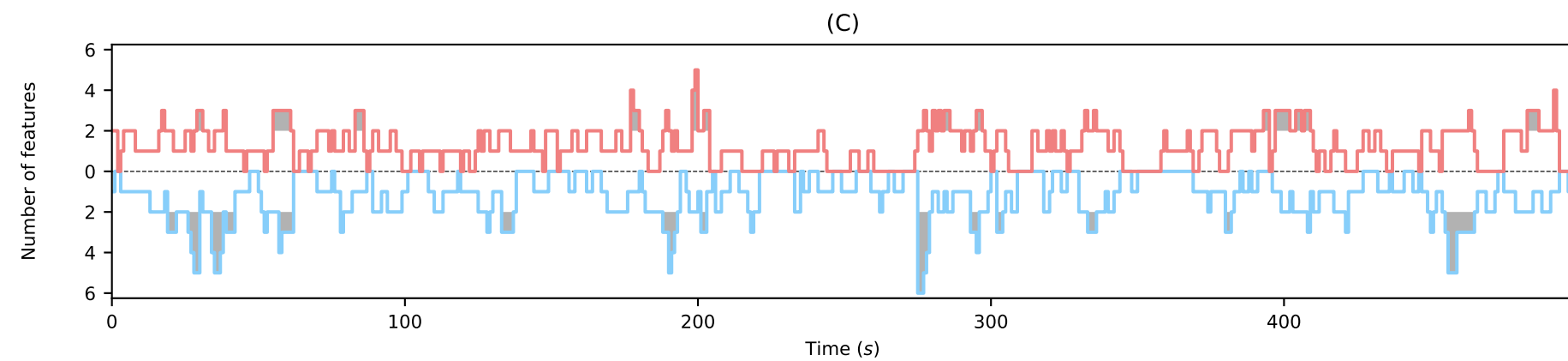

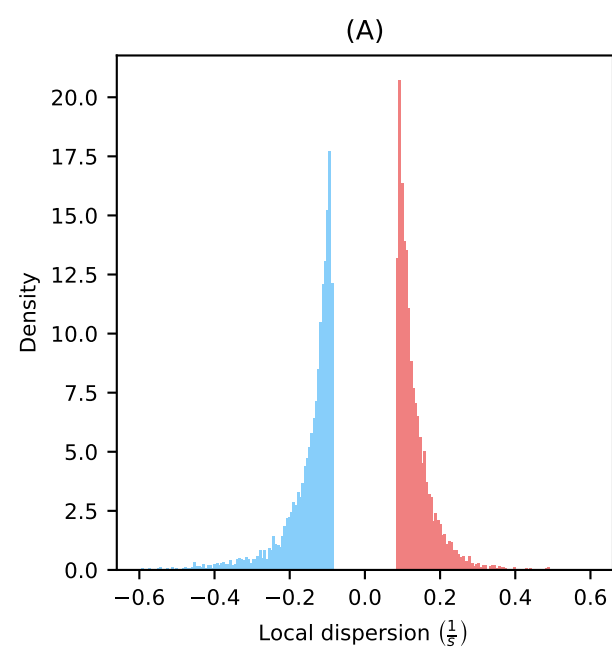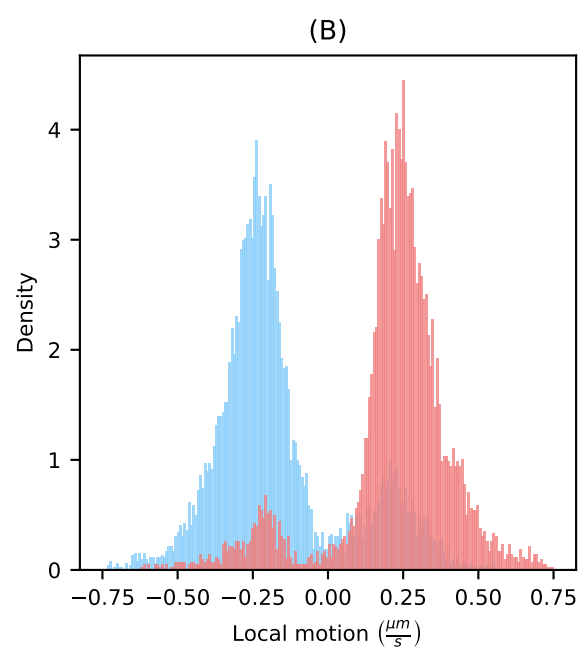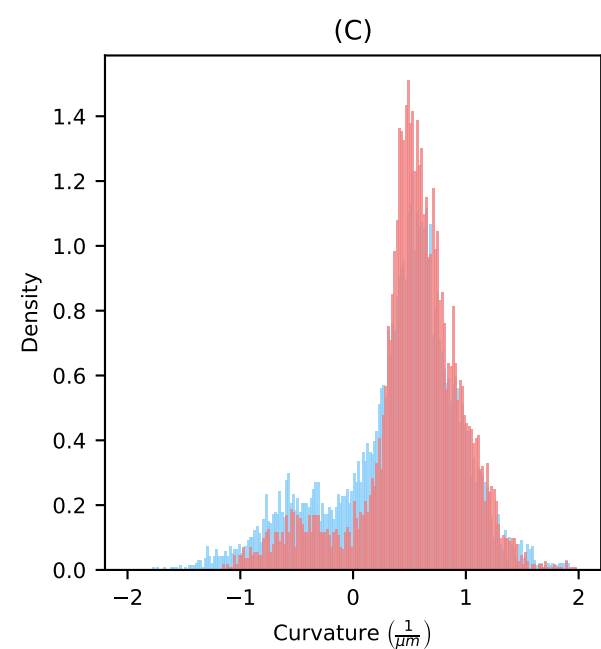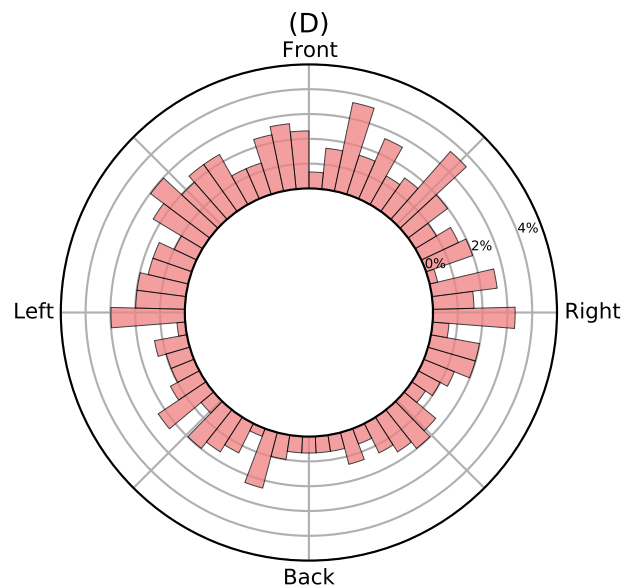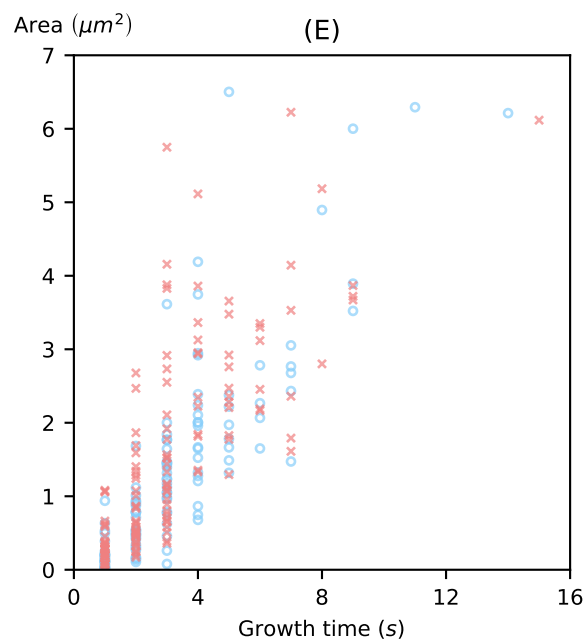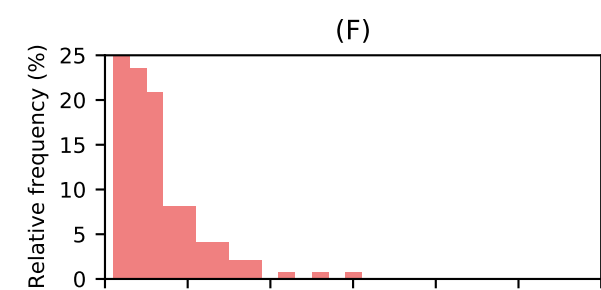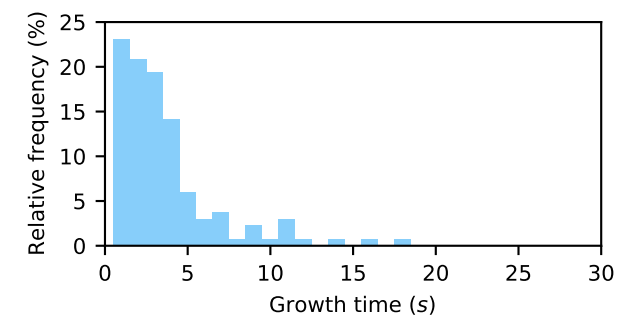

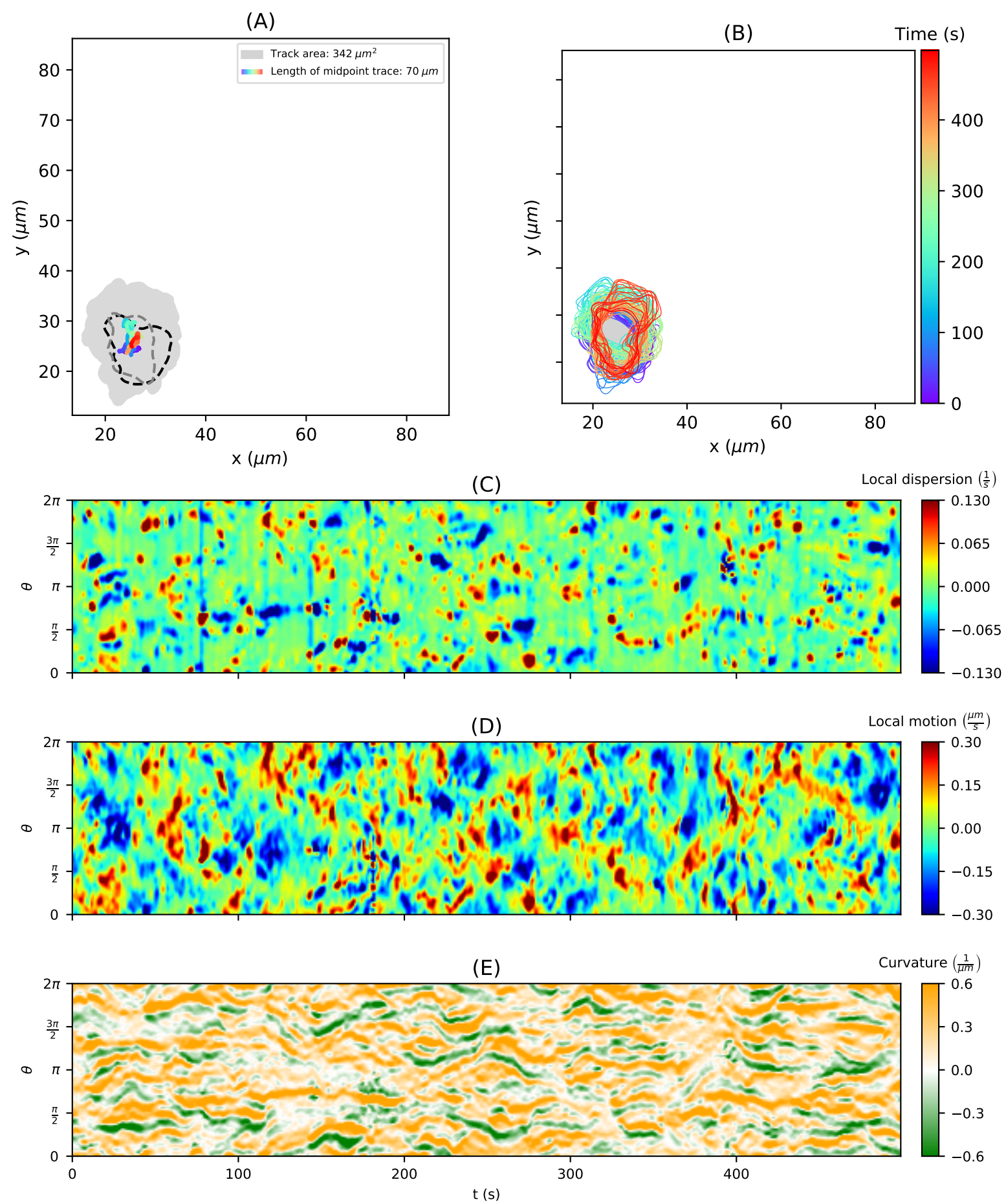

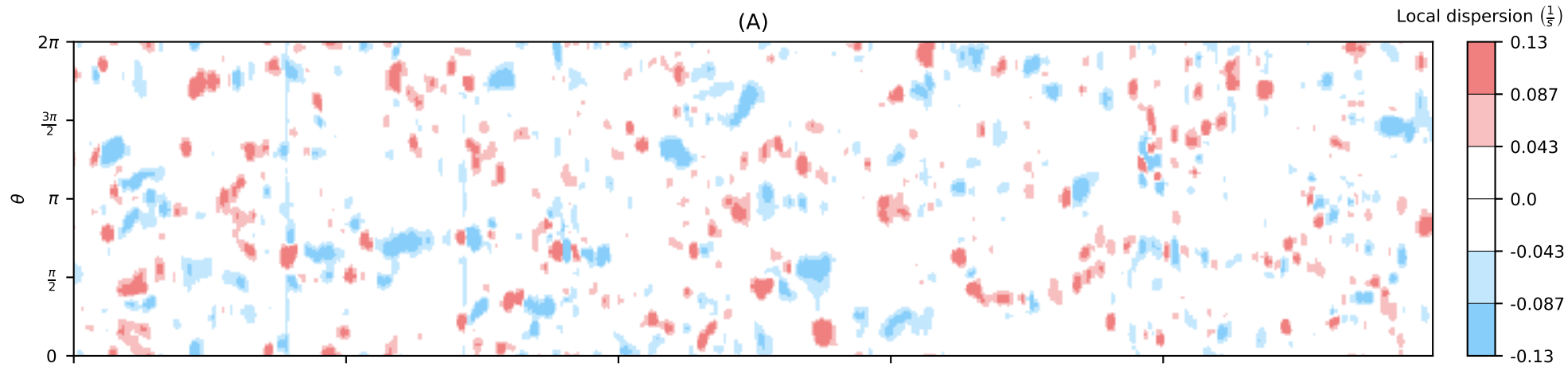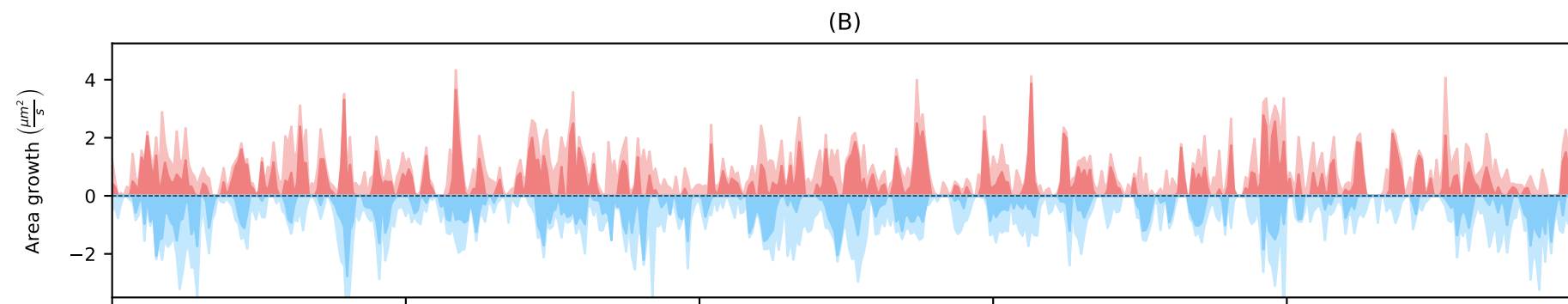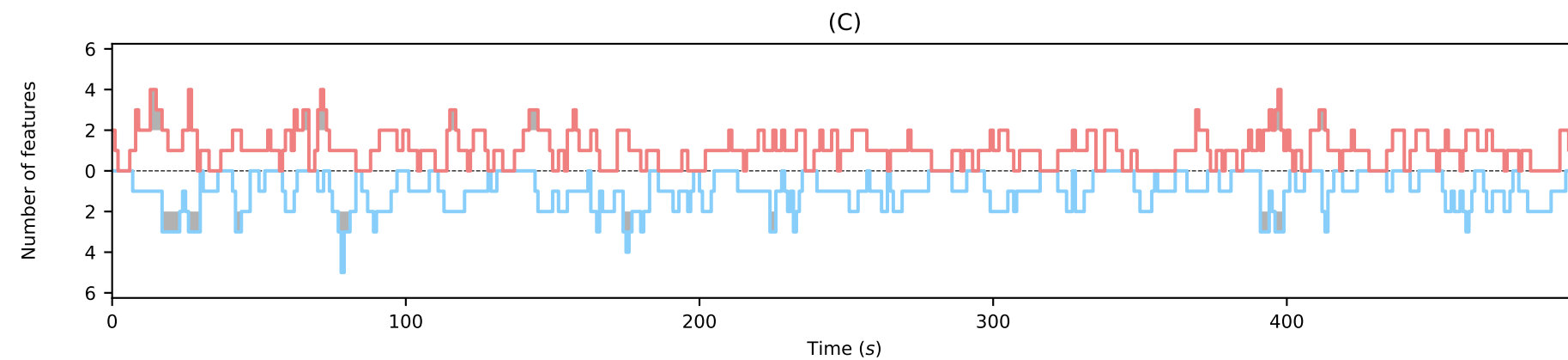

(A)

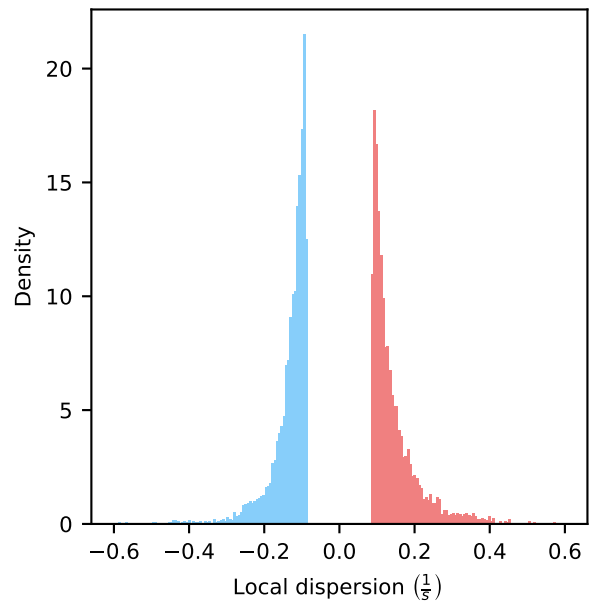

(B)

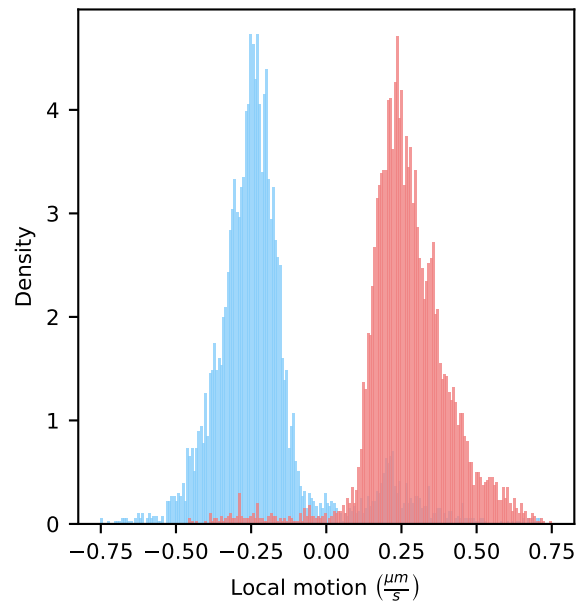

(C)

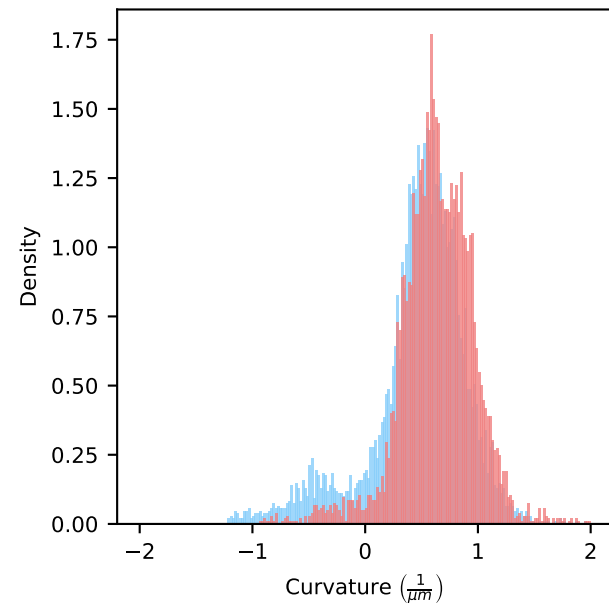

(D)

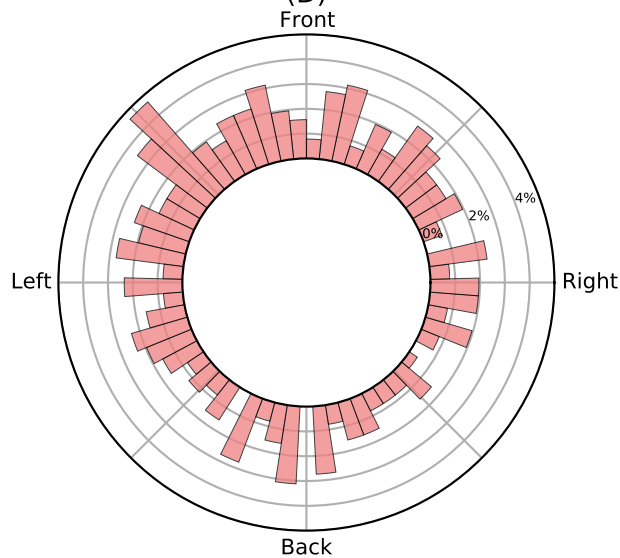

(E)

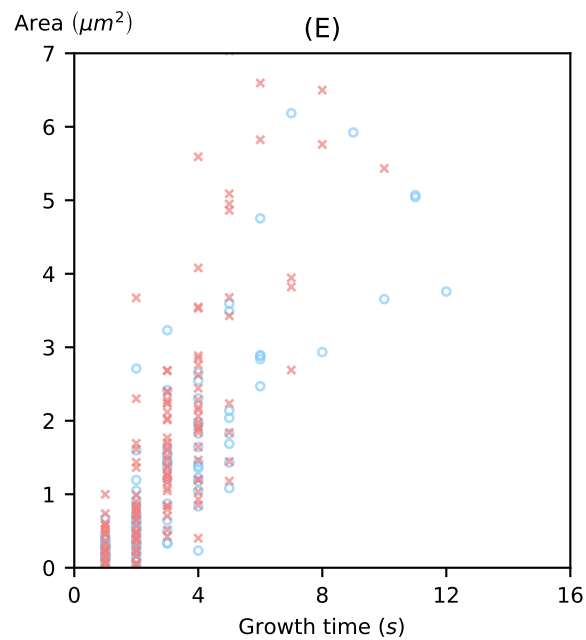

(F)

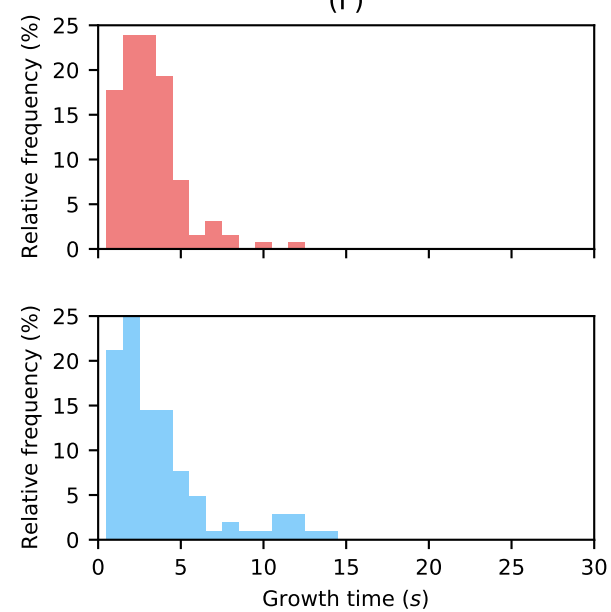

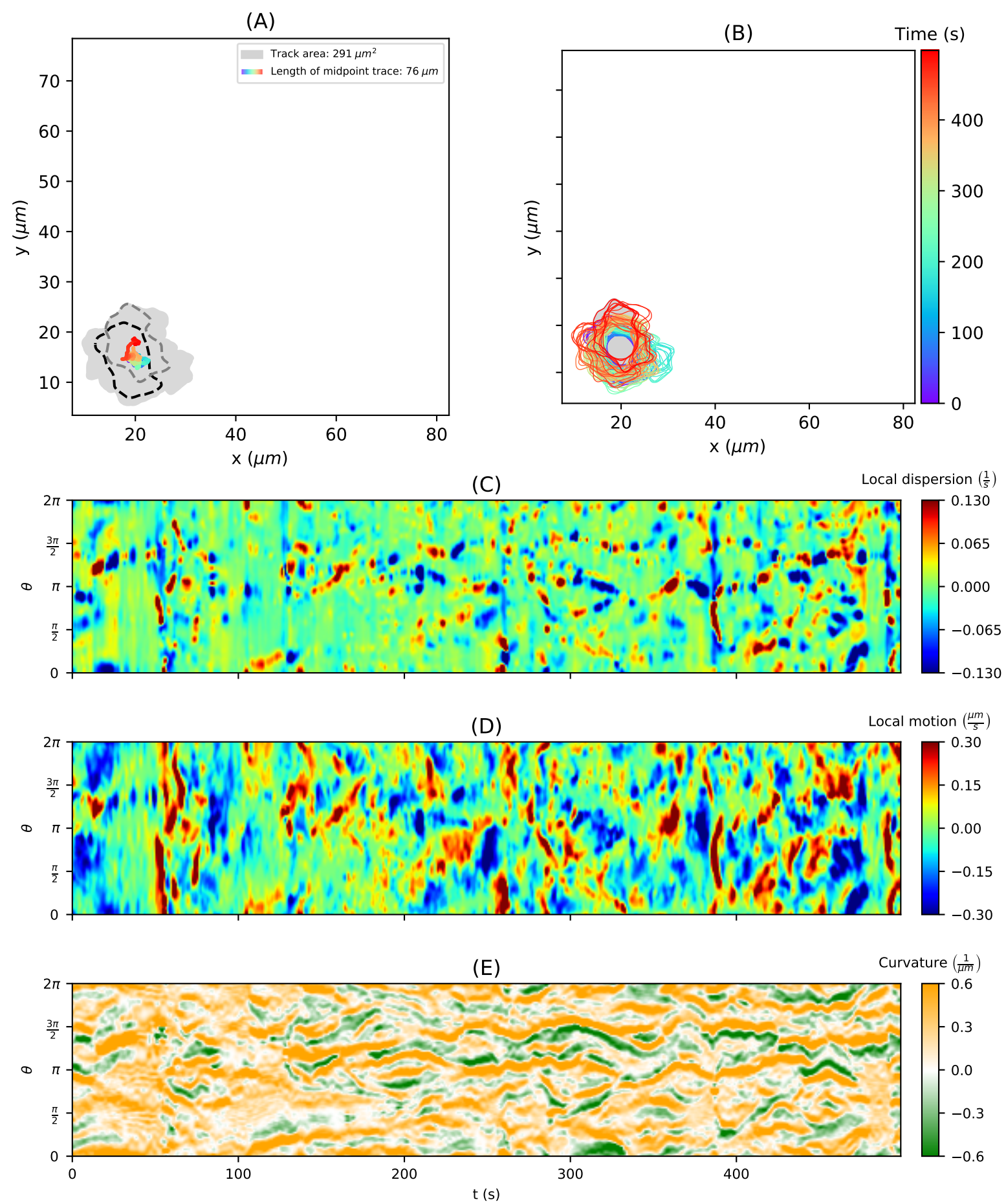

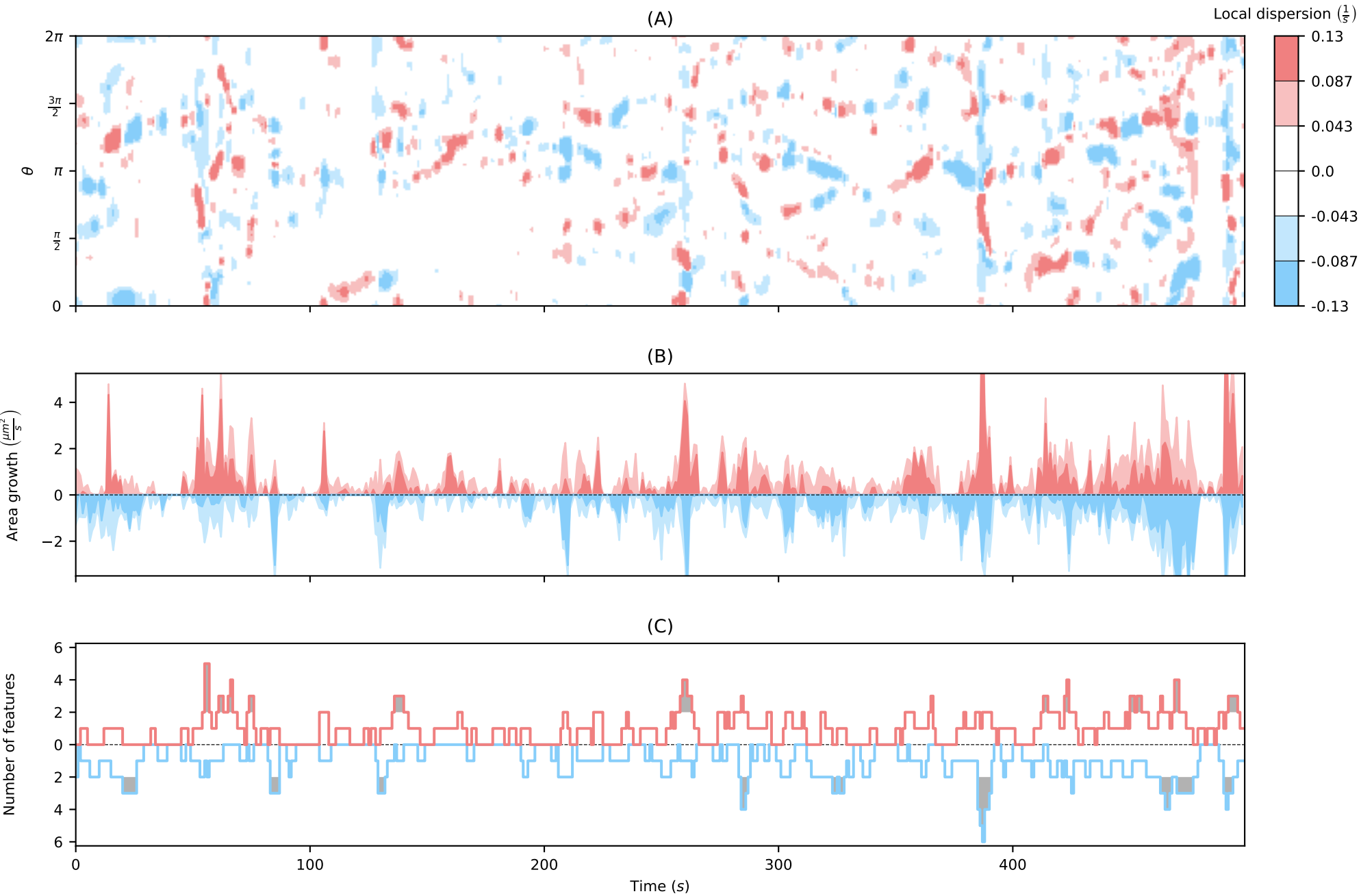

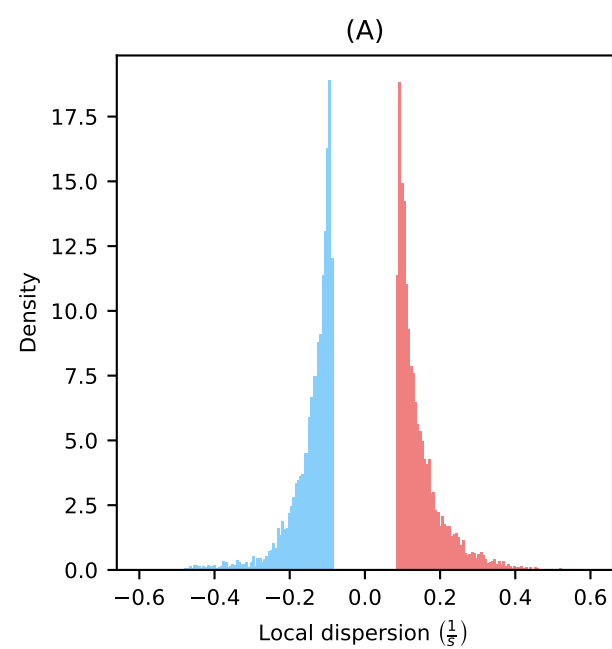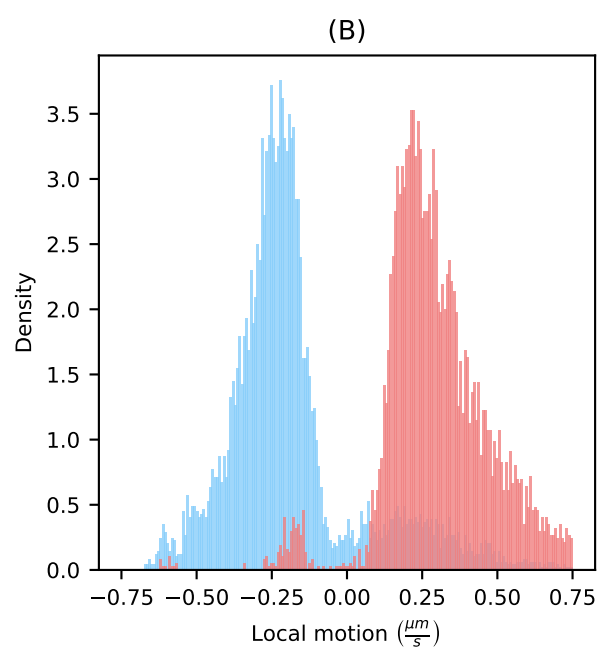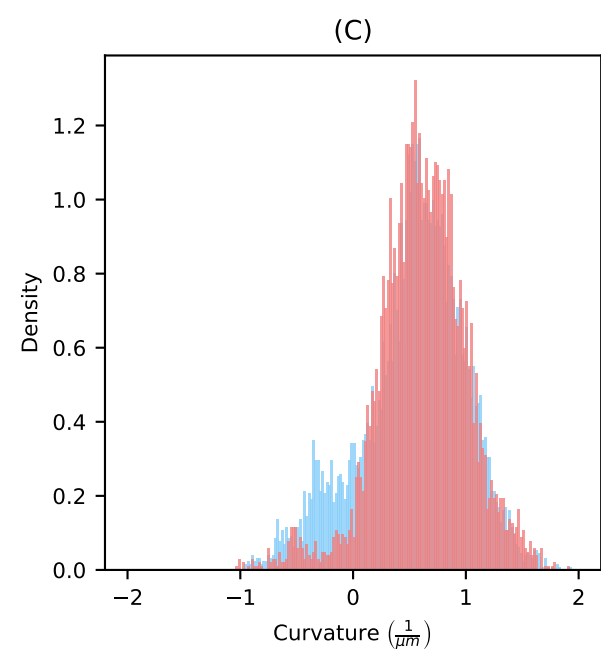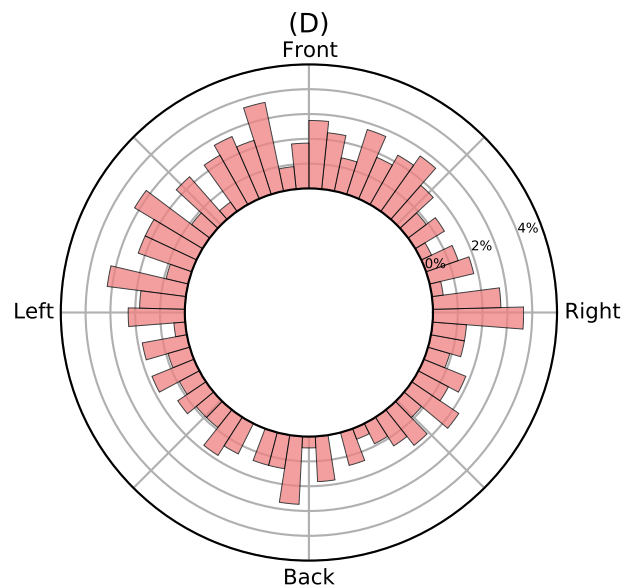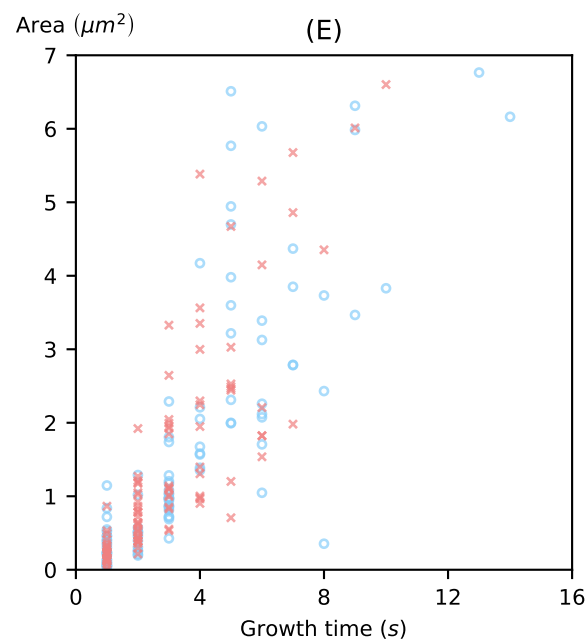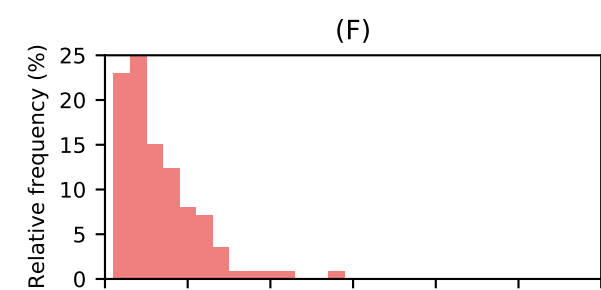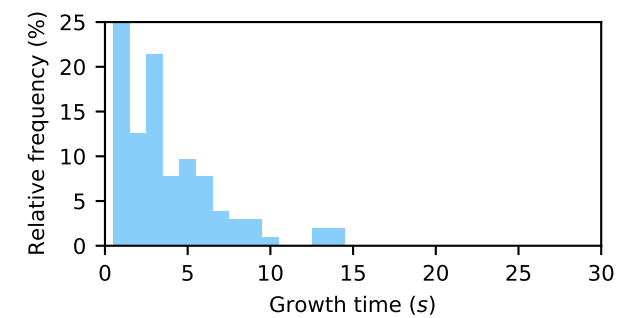

Supplement: S14 Fig — The file is structured as in S13 Fig. (PDF) [file pcbi.1009268.s015.pdf]
