# Supplementary material for: Analysis of protrusion dynamics in amoeboid cell motility by means of regularized contour flows
Source: PLoS Comput Biol. 2021 Aug 23;17(8):e1009268. doi: 10.1371/journal.pcbi.1009268 (PMC8412247; doi:10.1371/journal.pcbi.1009268)
Supplement: S12 Fig — The correlation is shown for the cell track in Fig 6: Persistently motile, weakly motile and almost stationary cell. (PDF) [file pcbi.1009268.s013.pdf]

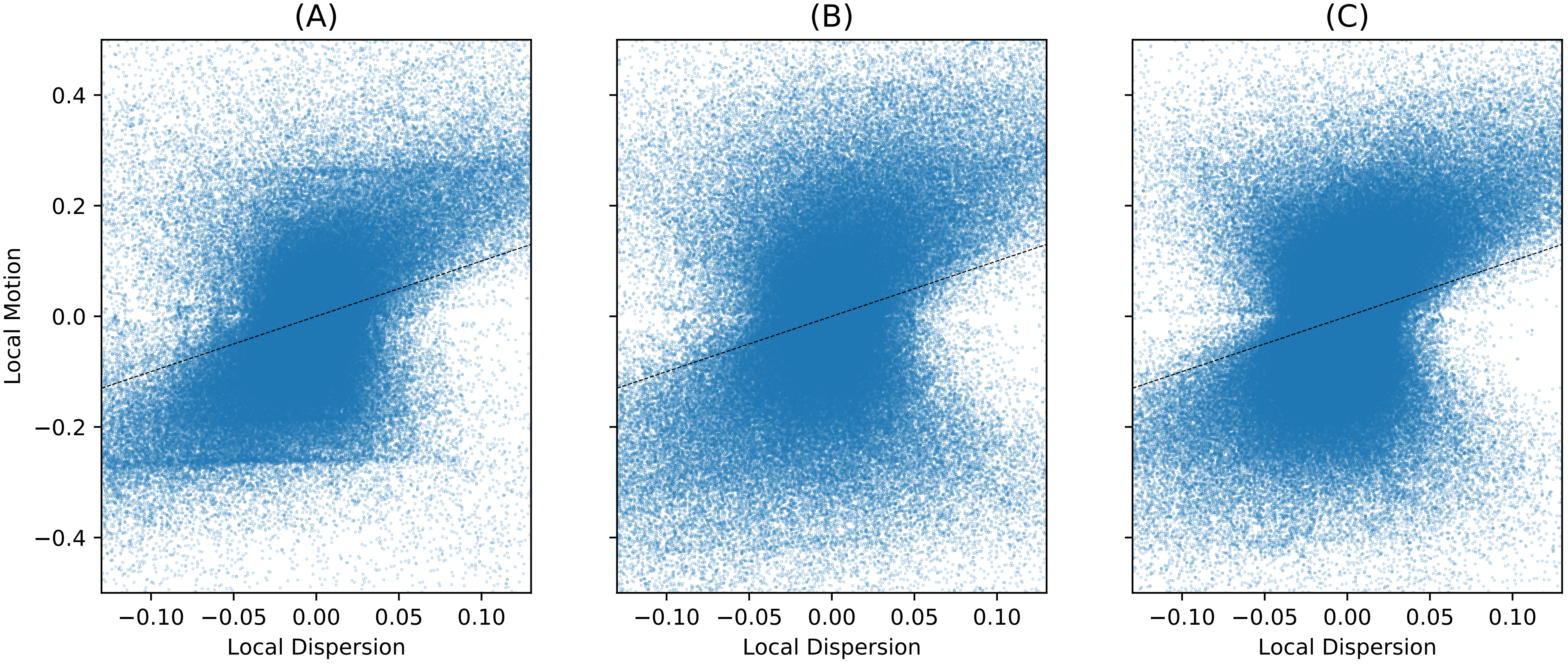

Fig S12. Correlation between local dispersion and local motion. The correlation is shown for different example cell tracks: Persistently motile **(A)**, medium motile **(B)** and rather stationary cell **(C).**
