# Supplementary material for: Analysis of protrusion dynamics in amoeboid cell motility by means of regularized contour flows
Source: PLoS Comput Biol. 2021 Aug 23;17(8):e1009268. doi: 10.1371/journal.pcbi.1009268 (PMC8412247; doi:10.1371/journal.pcbi.1009268)
Supplement: S15 Fig — Only features with minimal persistence length Δt ≥ 3 are shown. (PDF) [file pcbi.1009268.s016.pdf]

Expansion collection, threshold: 0.087

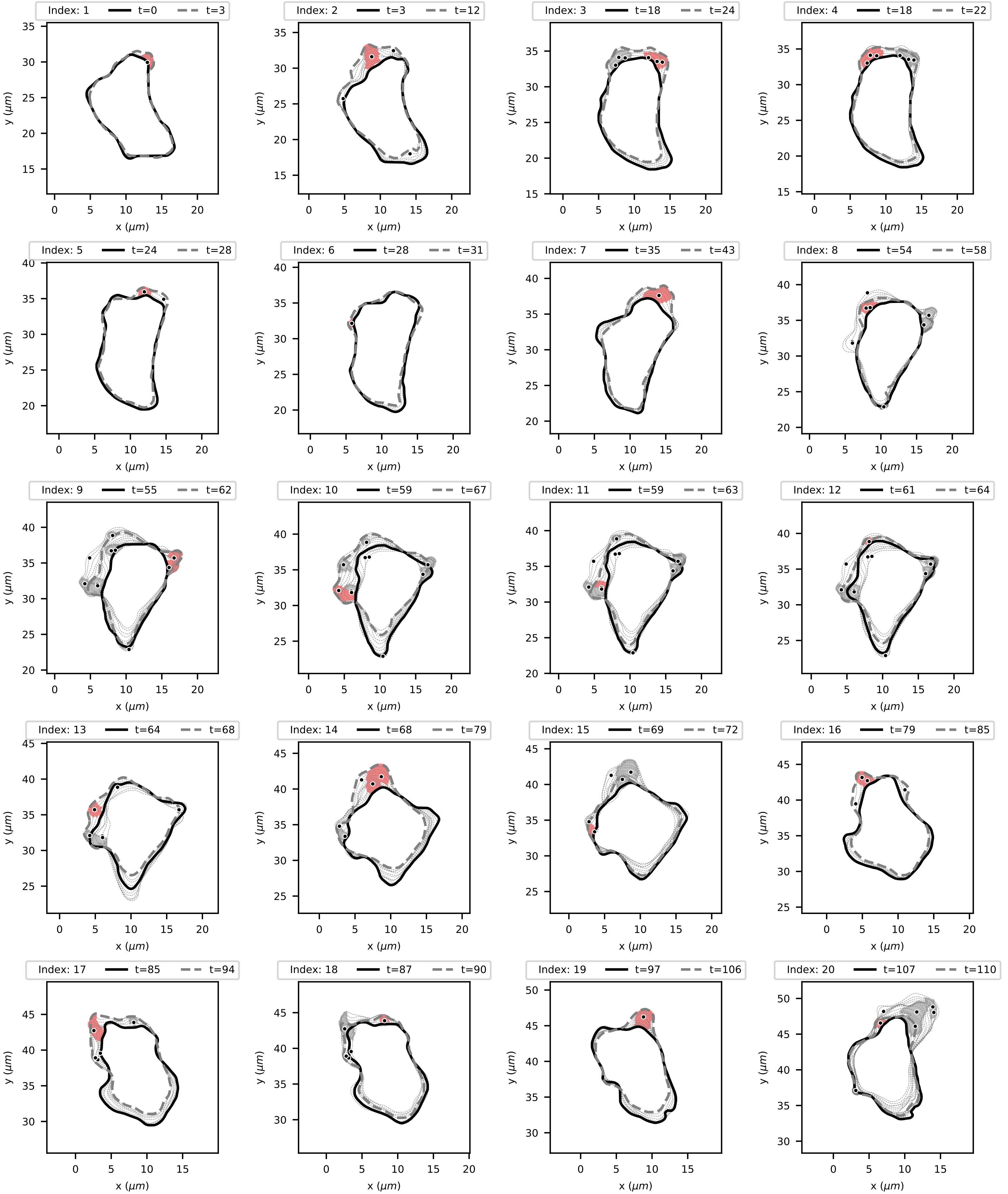

Expansion collection, threshold: 0.087

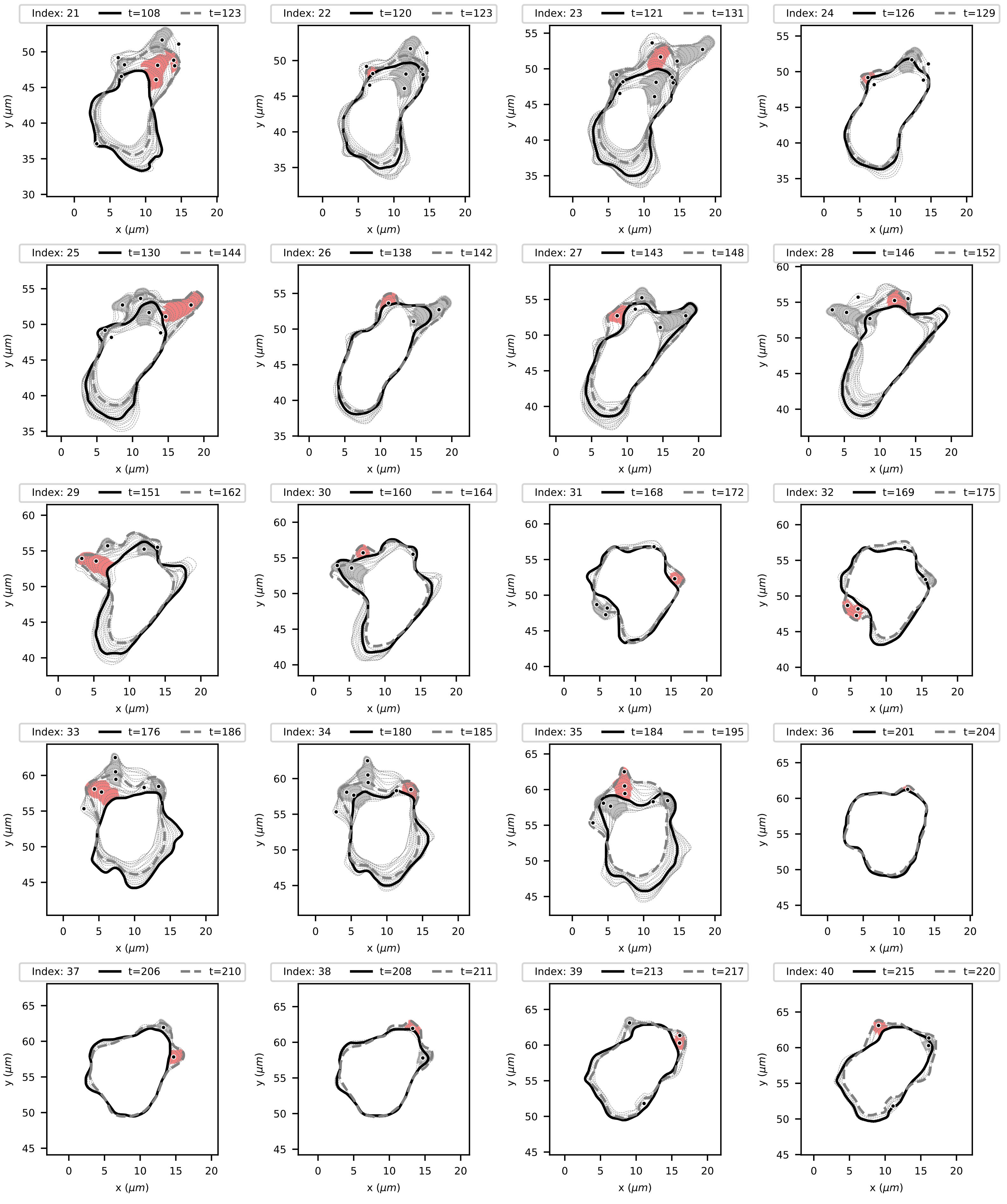

Expansion collection, threshold: 0.087

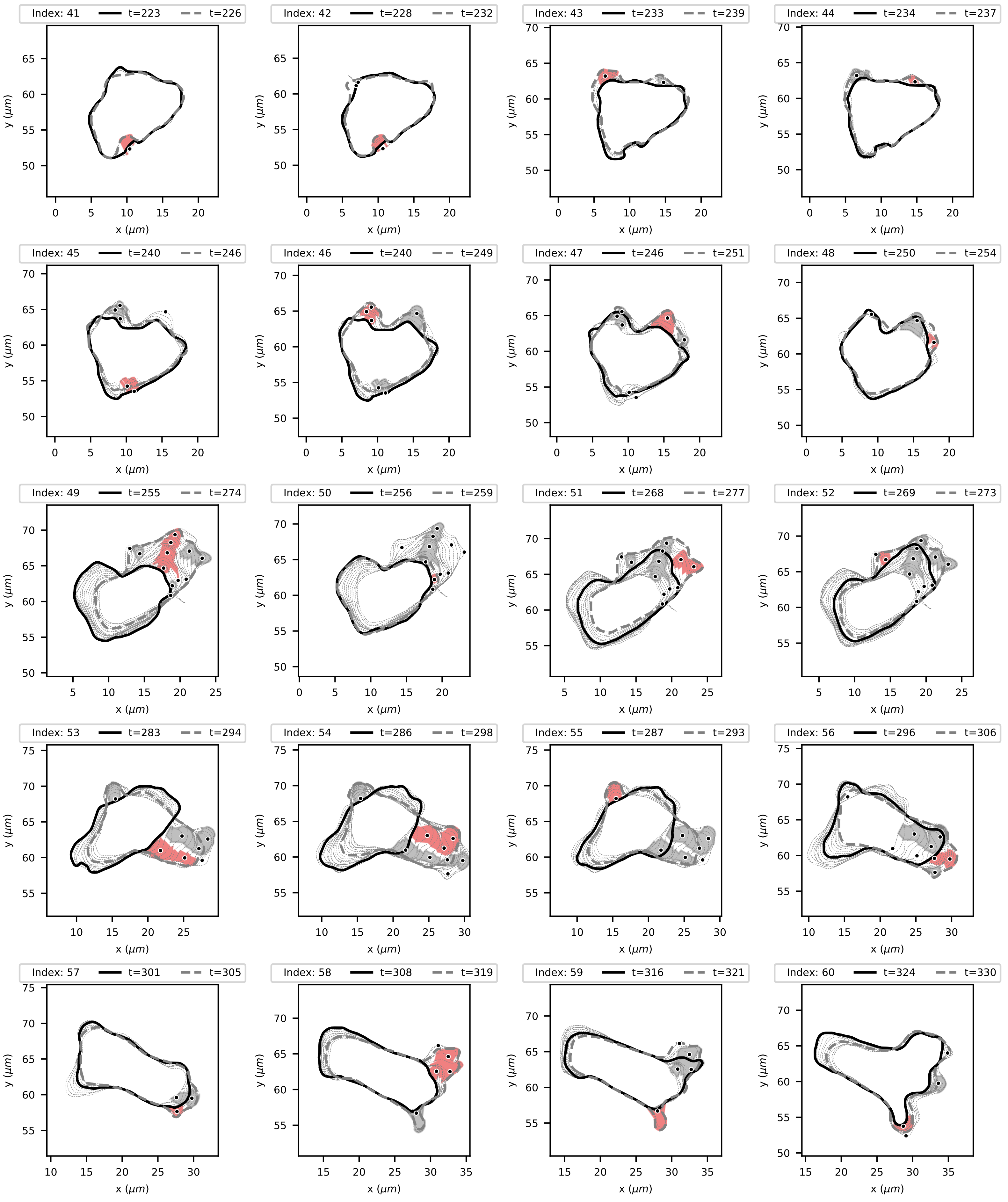

Expansion collection, threshold: 0.087

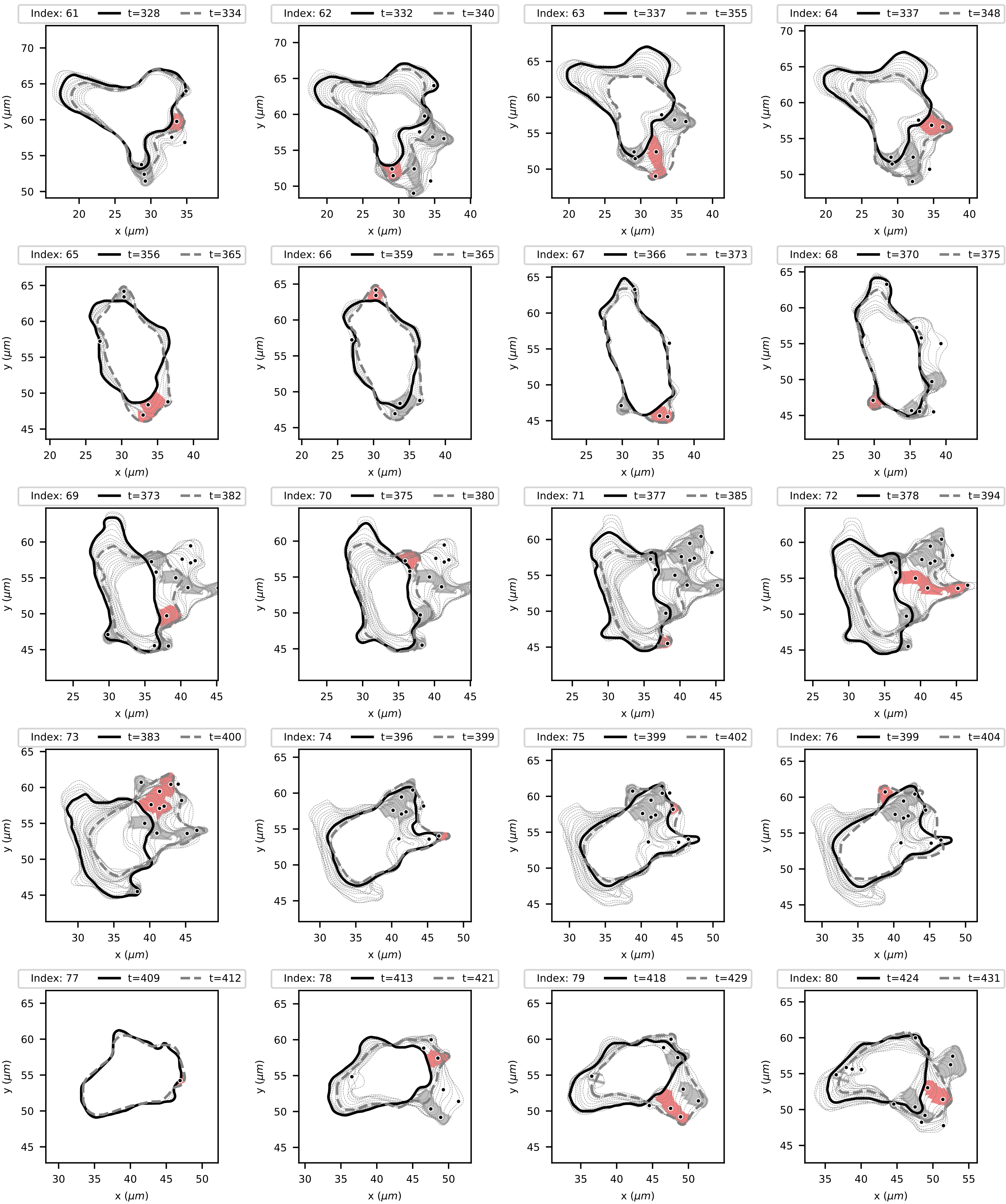

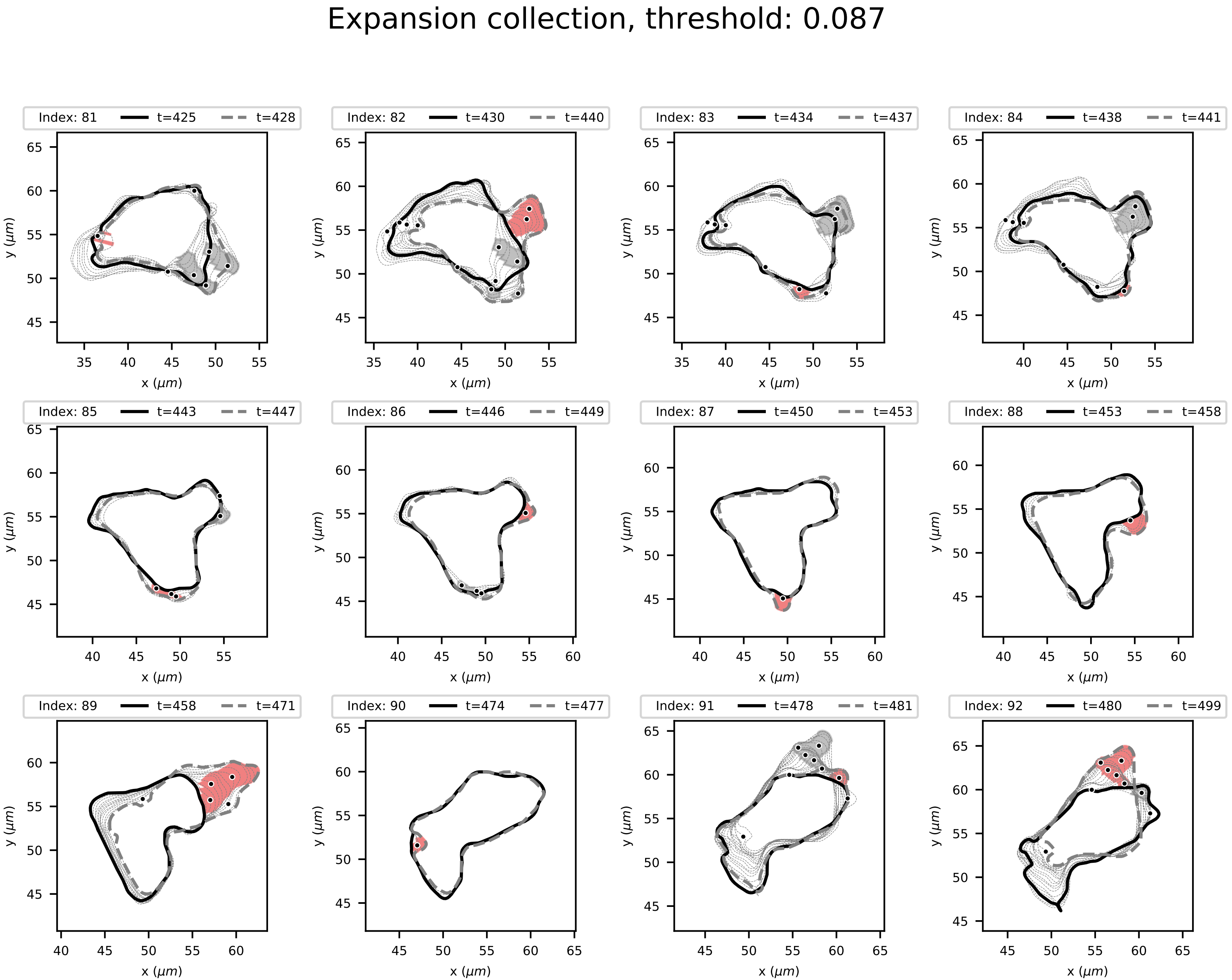
Expansion collection, threshold: 0.087
Index: 81 t=425 t=428
Index: 82 t=430 t=440
Index: 83 t=434 t=437
Index: 84 t=438 t=441
Index: 85 t=443 t=447
Index: 86 t=446 t=449
Index: 87 t=450 t=453
Index: 88 t=453 t=458
Index: 89 t=458 t=471
Index: 90 t=474 t=477
Index: 91 t=478 t=481
Index: 92 t=480 t=499
x (μm)
y (μm)
